# Supplementary material for: Circulating miRNAs as Noninvasive Biomarkers for PDAC Diagnosis and Prognosis in Mexico
Source: Int J Mol Sci. 2023 Oct 14;24(20):15193. doi: 10.3390/ijms242015193 (PMC10607652; doi:10.3390/ijms242015193)
Supplement: Supplementary file 1 [file ijms-24-15193-s001.zip › ijms-2575958-supplementary.pdf]

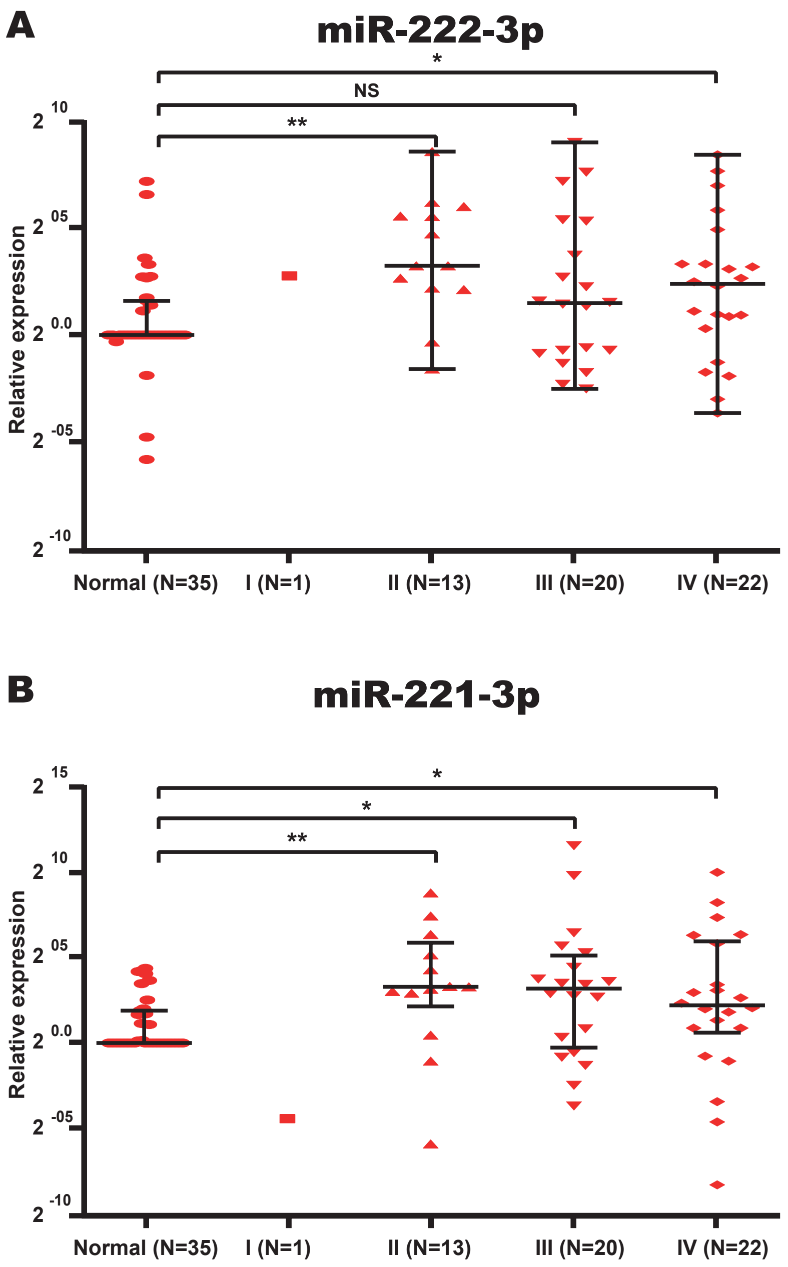

Figure S1. miR-222-3p and miR-221-3p expression according to PDAC stages. **(A)** The miR-222-3p and **(B)** miR-221-3p expression was analyzed by RT-qPCR assays in tissue and plasma samples of PDAC patients and control people that live in Mexico. The differences were determined using the Mann-Whitney test. NS: not significant, **p*-value<0.05 and ***p*-value<0.01.

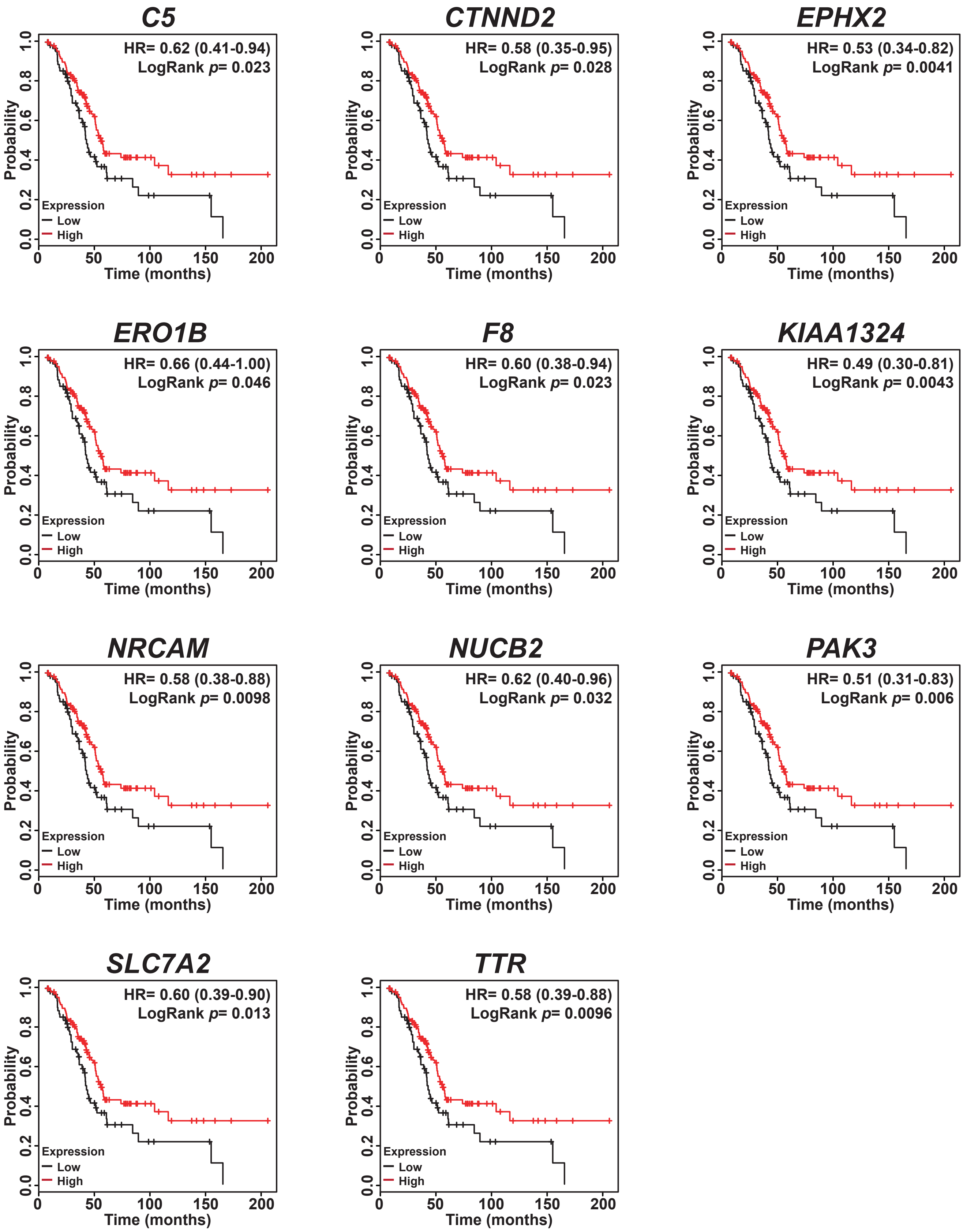

Figure S2. 47 mRNAs were identified as targets of 4 DEmiRNAs and their impact on the prognosis of PDAC. Kaplan-Meier curves of 11 mRNAs were identified as targets of 4 DEmiRNAs identified in this work.

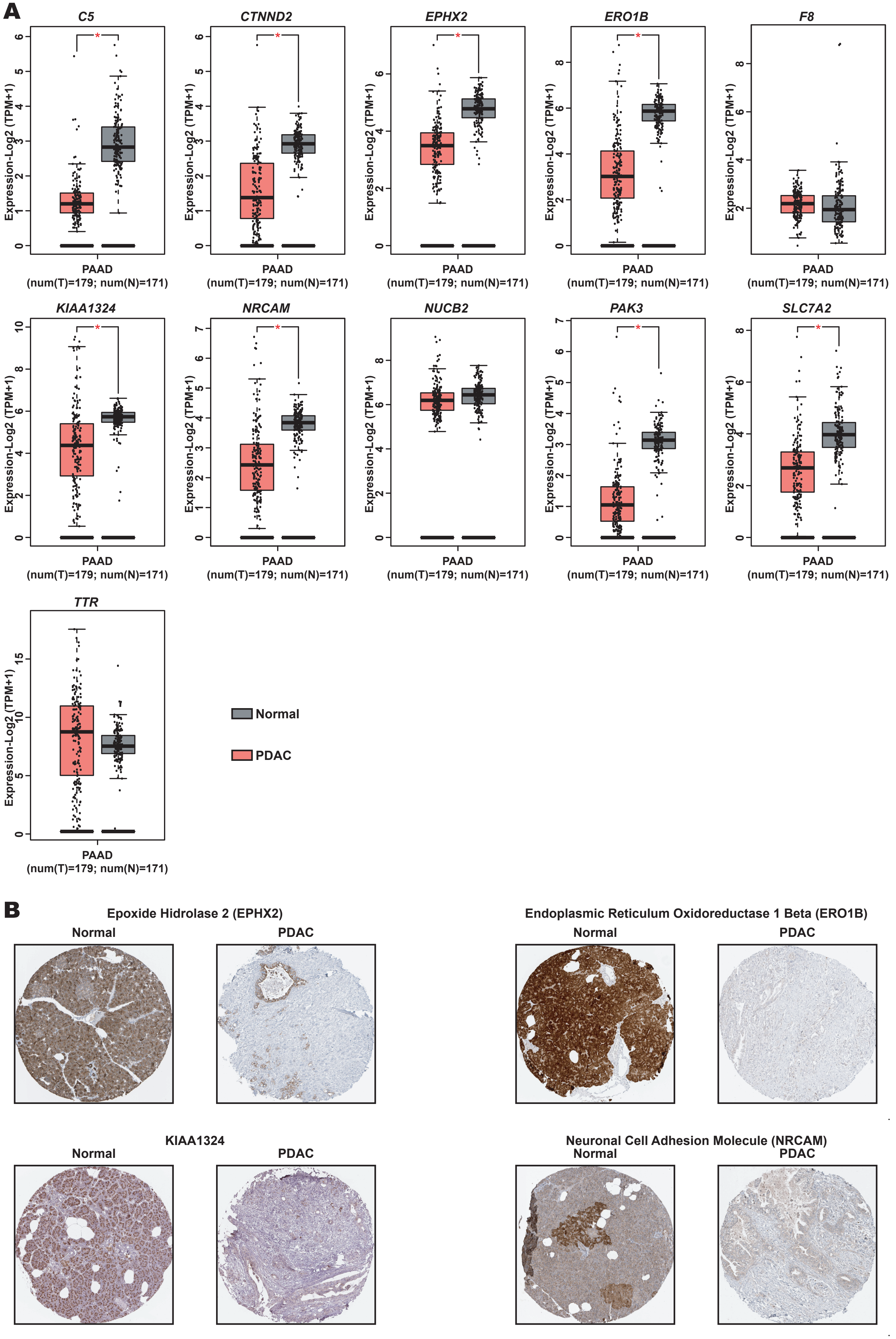
A
C5
CTNND2
EPHX2
ERO1B
F8
KIAA1324
NRCAM
NUCB2
PAK3
SLC7A2
TTR
Expression-Log2 (TPM+1)
PAAD
(num(T)=179; num(N)=171)
Normal
PDAC
B
Epoxide Hidrolase 2 (EPHX2)
Normal
PDAC
Endoplasmic Reticulum Oxidoreductase 1 Beta (ERO1B)
Normal
PDAC
KIAA1324
Normal
PDAC
Neuronal Cell Adhesion Molecule (NRCAM)
Normal
PDAC

Figure S3. Expression of 11 mRNAs in PDAC **(A)** The box plot was downloaded from the GEPIA tool. The gray and red boxes represent normal and cancer tissues, respectively. Tumor, T; Normal, N; and *, *p*-value<0.05, considered as statistically significant. PAAD pancreatic adenocarcinoma. **(B)** Immunohistochemistry of the four target mRNAs of 4 DEmiRNAs found in this work. EPHX2: Normal: staining: high; intensity: strong; quantity: >75%. PDAC: staining: medium; intensity: moderate; quantity: <25%. ERO1B: Normal: staining: high; intensity: strong; quantity: >75%. PDAC: staining: not detected; intensity: weak; quantity: <25%. KIAA1324: Normal: staining: high; intensity: strong; quantity: >75%. PDAC: staining: low; intensity: moderate; quantity: <25%. NRCAM: Normal: staining: high; intensity: strong; quantity: >75%. PDAC: staining: low; intensity: weak; quantity: >75%.

Table S1. mRNAs target of four differentially expressed miRNAs identified in this study.

hsa-miR-222-3p	hsa-miR-345-5p	hsa-miR-100-5p	hsa-miR-221-3p
A1CF	*A1CF*	*ABCA5*	*A1CF*
A4GNT	*A4GNT*	*ABCC5*	*A4GNT*
AADAC	*AADAC*	*ABCF3*	*AADAC*
AADACL2	*AAGAB*	*ABCG2*	*AADACL2*
AAGAB	*AAK1*	*ABHD17A*	*AAGAB*
AAK1	*AAR2*	*ABHD2*	*AAK1*
ABAT	*AARD*	*ABLIM1*	*ABAT*
ABCA1	*AARS2*	*ABLIM2*	*ABCA1*
ABCA3	*AASDH*	*ACAA2*	*ABCA3*
ABCA5	*AASDHPPT*	*ACAD10*	*ABCA5*
ABCA8	*ABAT*	*ACAP2*	*ABCA8*
ABCB11	*ABCA1*	*ACKR3*	*ABCB11*
ABCB5	*ABCA10*	*ACO1*	*ABCB5*
ABCC11	*ABCA4*	*ACOT11*	*ABCC11*
ABCC12	*ABCA5*	*ACOT9*	*ABCC12*
ABCC5	*ABCA6*	*ACSBG1*	*ABCC5*
ABCD2	*ABCA8*	*ACSF3*	*ABCD2*
ABCF1	*ABCA9*	*ACSL3*	*ABCF1*
ABCF3	*ABCB10*	*ACTB*	*ABCF3*
ABCG1	*ABCB11*	*ACTR1B*	*ABCG2*
ABCG2	*ABCB5*	*ACVR2B*	*ABHD12*
ABHD12	*ABCB6*	*ADAM19*	*ABHD13*
ABHD13	*ABCB8*	*ADAMTS5*	*ABHD17A*
ABHD17A	*ABCB9*	*ADAP1*	*ABHD2*
ABHD2	*ABCC12*	*ADARB2*	*ABHD3*
ABHD3	*ABCC5*	*ADAT1*	*ABHD5*
ABHD4	*ABCC6*	*ADAT2*	*ABITRAM*
ABHD5	*ABCD2*	*ADAT3*	*ABL1*
ABI3BP	*ABCD3*	*ADCK1*	*ABL2*
ABL1	*ABCD4*	*ADCY1*	*ABLIM1*
ABLIM1	*ABCE1*	*ADGRE2*	*ABRACL*
ABRAXAS2	*ABCF1*	*ADH1B*	*ABTB2*
ABTB2	*ABCF2*	*AFAP1L1*	*ACAA2*
ACAA2	*ABCF3*	*AGAP1*	*ACAD10*
ACACA	*ABCG1*	*AGBL5*	*ACADL*
ACAD10	*ABCG2*	*AGFG2*	*ACADM*
ACADL	*ABHD10*	*AGO2*	*ACADSB*
ACADM	*ABHD13*	*AGO3*	*ACAP3*
ACAP3	*ABHD14A*	*AGPAT3*	*ACBD5*
ACBD5	*ABHD14A-ACY1*	*AGPAT4*	*ACBD7*
ACBD7	*ABHD15*	*AGTPBP1*	*ACCS*
ACCS	*ABHD17A*	*AHR*	*ACE*

ACE	*ABHD17B*	*AIG1*	*ACER2*
ACER2	*ABHD17C*	*AIPL1*	*ACER3*
ACER3	*ABHD18*	*AK4*	*ACIN1*
ACOD1	*ABHD2*	*AKAP1*	*ACOD1*
ACOT11	*ABHD3*	*AKAP13*	*ACOT11*
ACOT2	*ABHD5*	*AKR7A2*	*ACOT2*
ACOT9	*ABHD6*	*AKT1*	*ACOT9*
ACOX1	*ABI1*	*ALDH3B2*	*ACOX1*
ACP7	*ABI3BP*	*ALDH9A1*	*ACOX3*
ACSF2	*ABITRAM*	*ALG13*	*ACSF2*
ACSL3	*ABL1*	*ALG14*	*ACSL3*
ACSL6	*ABL2*	*ALG3*	*ACSM5*
ACSM5	*ABLIM1*	*ALKBH2*	*ACSS1*
ACSS1	*ABLIM3*	*ALS2*	*ACSS3*
ACSS3	*ABRA*	*ALS2CL*	*ACTB*
ACTB	*ABRACL*	*AMER3*	*ACTC1*
ACTC1	*ABRAXAS1*	*AMMECR1*	*ACTG1*
ACTG1	*ABRAXAS2*	*AMN*	*ACTL7A*
ACTL7A	*ABT1*	*ANAPC1*	*ACTR1A*
ACTR1A	*ABTB2*	*ANAPC11*	*ACTR2*
ACTR2	*ACACB*	*ANGPT2*	*ACTR3*
ACTR3	*ACAD8*	*ANGPT4*	*ACTR3B*
ACVR1B	*ACADL*	*ANK3*	*ACVR1B*
ACVR1C	*ACADM*	*ANKLE1*	*ACVR1C*
ACVR2A	*ACADSB*	*ANKRD28*	*ACVR2A*
ACVR2B	*ACAN*	*ANKRD33B*	*ACVR2B*
ADAD1	*ACAP2*	*ANKRD49*	*ADAD1*
ADAL	*ACBD3*	*ANKRD52*	*ADAL*
ADAM10	*ACBD5*	*ANKRD63*	*ADAM10*
ADAM11	*ACBD7*	*ANKS6*	*ADAM11*
ADAM12	*ACER2*	*ANP32E*	*ADAM12*
ADAM17	*ACER3*	*ANTXR1*	*ADAM17*
ADAM1A	*ACHE*	*AOPEP*	*ADAM18*
ADAM22	*ACKR3*	*AP1AR*	*ADAM1A*
ADAM23	*ACKR4*	*AP1M1*	*ADAM22*
ADAM28	*ACO1*	*AP1S1*	*ADAM23*
ADAM8	*ACO2*	*AP3M1*	*ADAM28*
ADAM9	*ACOT11*	*AP5B1*	*ADAM8*
ADAMTS1	*ACOT13*	*APBA1*	*ADAM9*
ADAMTS14	*ACOT2*	*APBA3*	*ADAMTS1*
ADAMTS17	*ACOT4*	*APC2*	*ADAMTS14*
ADAMTS5	*ACOT9*	*APCDD1*	*ADAMTS16*
ADAMTSL1	*ACOX1*	*APEX1*	*ADAMTS17*
ADARB2	*ACP1*	*API5*	*ADAMTS2*

ADAT1	*ACPP*	*APOA1*	*ADAMTS5*
ADCY1	*ACRV1*	*APOBEC3D*	*ADAMTS6*
ADCY2	*ACSBG1*	*APOBEC3F*	*ADAMTSL1*
ADCY9	*ACSF3*	*APOC3*	*ADAP1*
ADCYAP1	*ACSL3*	*APOL2*	*ADARB2*
ADCYAP1R1	*ACSL4*	*AR*	*ADAT1*
ADD1	*ACSL6*	*ARFGEF1*	*ADCY1*
ADD2	*ACSM2A*	*ARHGEF7*	*ADCY2*
ADD3	*ACSM2B*	*ARID3A*	*ADCY9*
ADGRL3	*ACSM3*	*ARID3B*	*ADCYAP1*
ADH1B	*ACSM4*	*ARPP19*	*ADCYAP1R1*
ADH5	*ACSS3*	*ARRB1*	*ADD1*
ADHFE1	*ACTBL2*	*ARRDC4*	*ADD2*
ADIPOR1	*ACTC1*	*ARSD*	*ADD3*
ADM2	*ACTL6A*	*ART5*	*ADGRG3*
ADNP2	*ACTL7B*	*ASB7*	*ADGRL2*
ADO	*ACTN2*	*ATAT1*	*ADGRL3*
ADRA2C	*ACTR10*	*ATF7IP2*	*ADH1B*
ADSL	*ACTR2*	*ATIC*	*ADH5*
AFDN	*ACTR3*	*ATM*	*ADHFE1*
AFF1	*ACTR3C*	*ATP10B*	*ADI1*
AFF2	*ACTR6*	*ATP11A*	*ADIPOR1*
AFF3	*ACTR8*	*ATP11C*	*ADM2*
AFF4	*ACTRT1*	*ATP1A2*	*ADNP2*
AFG1L	*ACVR1*	*ATP1B3*	*ADO*
AGAP1	*ACVR1B*	*ATP2A2*	*ADSL*
AGBL5	*ACVR1C*	*ATP2B1*	*AFDN*
AGFG1	*ACVR2A*	*ATP2B3*	*AFF1*
AGL	*ACVR2B*	*ATP2B4*	*AFF2*
AGPAT3	*ACVRL1*	*ATP5F1A*	*AFF3*
AGPAT4	*ACY1*	*ATP5MG*	*AFG1L*
AGPAT5	*ACYP2*	*ATP5PB*	*AGAP1*
AGPS	*ADA2*	*ATP6AP1*	*AGBL2*
AGTPBP1	*ADAL*	*ATP6AP1L*	*AGBL5*
AGTRAP	*ADAM10*	*ATP6V1C2*	*AGFG1*
ago-01	*ADAM12*	*ATP7B*	*AGGF1*
ago-02	*ADAM17*	*AUTS2*	*AGL*
ago-03	*ADAM18*	*AVPR1B*	*AGO1*
ago-04	*ADAM2*	*AXIN1*	*AGO2*
AHCTF1	*ADAM22*	*B3GALNT2*	*AGO3*
AHCY	*ADAM23*	*B4GALNT1*	*AGO4*
AHRR	*ADAM28*	*B4GALNT3*	*AGPAT3*
AHSA1	*ADAM7*	*B4GALT7*	*AGPAT4*
AIDA	*ADAM9*	*BAG5*	*AGPAT5*

AIRE	*ADAMTS12*	*BAK1*	*AGPS*
AJAP1	*ADAMTS15*	*BAMBI*	*AGTPBP1*
AK3	*ADAMTS16*	*BAZ1B*	*AGTRAP*
AK4	*ADAMTS17*	*BAZ2A*	*AHCY*
AK9	*ADAMTS2*	*BAZ2B*	*AHRR*
AKAP11	*ADAMTS20*	*BBX*	*AIDA*
AKAP13	*ADAMTS4*	*BCAR1*	*AIRE*
AKAP5	*ADAMTS5*	*BCAS4*	*AJAP1*
AKAP6	*ADAMTS8*	*BCL10*	*AK2*
AKAP9	*ADAMTSL1*	*BCL7C*	*AK3*
AKIRIN1	*ADAMTSL3*	*BECN1*	*AK4*
AKR1C1	*ADARB2*	*BEND2*	*AK9*
AKR7A2	*ADAT1*	*BEND6*	*AKAP13*
AKT2	*ADAT2*	*BID*	*AKAP2*
AKT3	*ADAT3*	*BIRC5*	*AKAP5*
AKTIP	*ADCK2*	*BLOC1S2*	*AKAP6*
ALDH16A1	*ADCY1*	*BLOC1S4*	*AKAP8*
ALDH1A1	*ADCY10*	*BMF*	*AKAP9*
ALDH1A2	*ADCY5*	*BMP2*	*AKIRIN1*
ALDH1B1	*ADCY9*	*BMPR2*	*AKR1C1*
ALDH1L2	*ADCYAP1*	*BRCC3*	*AKR1C2*
ALDH3A2	*ADCYAP1R1*	*BRD4*	*AKR7A2*
ALDH5A1	*ADD1*	*BRF2*	*AKT2*
ALG14	*ADD2*	*BRIX1*	*AKT3*
ALG3	*ADGB*	*BSG*	*AKTIP*
ALG6	*ADGRA3*	*BTBD9*	*ALDH16A1*
ALG8	*ADGRB3*	*BTD*	*ALDH1A1*
ALG9	*ADGRE2*	*BTG3*	*ALDH1A2*
ALKBH1	*ADGRF1*	*C10orf71*	*ALDH1B1*
ALPK3	*ADGRF5*	*C11orf49*	*ALDH1L2*
ALX4	*ADGRG2*	*C12orf45*	*ALG14*
AMACR	*ADGRG3*	*C12orf66*	*ALG6*
AMDHD2	*ADGRG6*	*C14orf28*	*ALG8*
AMER3	*ADGRG7*	*C16orf54*	*ALG9*
AMIGO1	*ADGRL1*	*C19orf38*	*ALKBH1*
AMIGO3	*ADGRL2*	*C1orf109*	*ALKBH8*
AMMECR1	*ADGRL3*	*C1orf115*	*ALPK3*
AMMECR1L	*ADGRL4*	*C20orf194*	*ALX4*
AMOT	*ADH1B*	*C21orf91*	*AMACR*
AMPD2	*ADH5*	*C2CD4C*	*AMD1*
AMPD3	*ADH6*	*C2orf72*	*AMDHD2*
AMPH	*ADIPOR1*	*C3*	*AMER3*
AMZ1	*ADIPOR2*	*C3orf52*	*AMIGO1*
ANAPC10	*ADK*	*C3orf67*	*AMIGO3*

ANAPC16	*ADM2*	*C3orf80*	*AMMECR1*
ANG	*ADNP*	*C5orf22*	*AMMECR1L*
ANGPTL2	*ADNP2*	*C7orf26*	*AMOT*
ANGPTL3	*ADO*	*CACNA1C*	*AMPD2*
ANGPTL8	*ADORA1*	*CACNA1I*	*AMPH*
ANK2	*ADPGK*	*CACNG7*	*AMZ1*
ANKDD1B	*ADPRHL1*	*CACNG8*	*ANAPC10*
ANKFY1	*ADRA1A*	*CACTIN*	*ANAPC16*
ANKHD1	*ADRA2A*	*CALCOCO2*	*ANG*
ANKIB1	*ADRB1*	*CALM2*	*ANGPTL2*
ANKMY1	*ADSL*	*CAMK2N1*	*ANGPTL3*
ANKRD10	*ADSS*	*CAMKK2*	*ANGPTL4*
ANKRD12	*ADTRP*	*CAMTA1*	*ANGPTL8*
ANKRD16	*AEBP2*	*CAND2*	*ANK2*
ANKRD17	*AEN*	*CAPN13*	*ANKDD1B*
ANKRD18A	*AFAP1L1*	*CAPNS1*	*ANKFY1*
ANKRD20A2	*AFAP1L2*	*CAPZB*	*ANKHD1*
ANKRD20A4	*AFDN*	*CARD9*	*ANKIB1*
ANKRD27	*AFF1*	*CASC4*	*ANKMY1*
ANKRD28	*AFF2*	*CASK*	*ANKRD10*
ANKRD33B	*AFF4*	*CASZ1*	*ANKRD12*
ANKRD34C	*AFG1L*	*CATSPER1*	*ANKRD16*
ANKRD36	*AFG3L2*	*CBL*	*ANKRD17*
ANKRD40	*AFMID*	*CBX3*	*ANKRD18A*
ANKRD44	*AFTPH*	*CBX6*	*ANKRD20A2*
ANKRD49	*AGA*	*CBX7*	*ANKRD20A4*
ANKRD50	*AGAP1*	*CCBE1*	*ANKRD27*
ANKRD52	*AGAP4*	*CCDC113*	*ANKRD28*
ANKRD54	*AGAP5*	*CCDC115*	*ANKRD33B*
ANKRD65	*AGAP6*	*CCDC117*	*ANKRD34C*
ANKS1B	*AGAP9*	*CCDC124*	*ANKRD44*
ANO1	*AGBL5*	*CCDC134*	*ANKRD49*
ANO10	*AGFG1*	*CCDC59*	*ANKRD50*
ANO3	*AGFG2*	*CCDC77*	*ANKRD52*
ANO7	*AGK*	*CCN1*	*ANKRD54*
ANOS1	*AGL*	*CCNG1*	*ANKRD55*
ANP32B	*AGMAT*	*CCNYL1*	*ANKRD65*
ANTXR2	*AGMO*	*CCR1*	*ANKS1B*
ANXA1	*AGO1*	*CCR4*	*ANO1*
ANXA11	*AGO2*	*CCZ1B*	*ANO10*
ANXA3	*AGO3*	*CD14*	*ANO3*
ANXA6	*AGO4*	*CD274*	*ANO6*
AP1AR	*AGPAT4*	*CD93*	*ANO7*
AP1B1	*AGPAT5*	*CDC25A*	*ANOS1*

AP1G1	*AGPS*	*CDC42BPG*	*ANP32B*
AP1M1	*AGR3*	*CDC42EP3*	*ANTXR2*
AP1S3	*AGTPBP1*	*CDC42EP4*	*ANXA1*
AP2A1	*AGTR2*	*CDC73*	*ANXA11*
AP3B2	*AGXT*	*CDCA3*	*ANXA3*
AP3M1	*AGXT2*	*CDH6*	*ANXA6*
AP5B1	*AHCTF1*	*CDK19*	*AP1AR*
AP5M1	*AHCY*	*CDK5RAP2*	*AP1G1*
AP5S1	*AHCYL1*	*CDK6*	*AP1M1*
APAF1	*AHCYL2*	*CDS1*	*AP1S1*
APBA1	*AHDC1*	*CDS2*	*AP1S3*
APCDD1L	*AHI1*	*CDYL2*	*AP2A1*
APEX2	*AHNAK*	*CELF1*	*AP3B1*
APLP2	*AHR*	*CELSR1*	*AP3B2*
APOBEC1	*AHRR*	*CENPF*	*AP3M1*
APOBEC3C	*AIDA*	*CEP104*	*AP5B1*
APOBEC3F	*AIF1L*	*CEP170B*	*AP5M1*
APOC3	*AIG1*	*CEP85*	*AP5S1*
APOL2	*AIMP1*	*CEP85L*	*APAF1*
APOL3	*AIPL1*	*CERK*	*APBA1*
APOL4	*AJAP1*	*CFAP20*	*APCDD1*
APOL6	*AJUBA*	*CFAP44*	*APCDD1L*
APOLD1	*AK2*	*CGAS*	*APEX2*
APOOL	*AK3*	*CGN*	*APLP2*
APP	*AK4*	*CHAF1B*	*APOB*
APPBP2	*AK5*	*CHERP*	*APOBEC1*
APPL1	*AK6*	*CHID1*	*APOBEC3C*
APTX	*AK9*	*CHRM2*	*APOBEC3F*
AQP12A	*AKAIN1*	*CHRNB2*	*APOC3*
AQP12B	*AKAP10*	*CHST3*	*APOL2*
AQP3	*AKAP11*	*CHSY1*	*APOL3*
AQP4	*AKAP12*	*CIAO3*	*APOL4*
AR	*AKAP13*	*CKS1B*	*APOL6*
ARAP2	*AKAP17A*	*CLCN6*	*APOLD1*
AREL1	*AKAP2*	*CLCN7*	*APOOL*
ARF1	*AKAP5*	*CLDN11*	*APP*
ARF4	*AKAP6*	*CLDN12*	*APPBP2*
ARF5	*AKAP7*	*CLEC16A*	*APPL1*
ARFGEF1	*AKIRIN1*	*CLEC4C*	*APTX*
ARFGEF3	*AKIRIN2*	*CLIC5*	*AQP12A*
ARFIP1	*AKR1C1*	*CLIP2*	*AQP12B*
ARHGAP11A	*AKR1C2*	*CLK4*	*AQP3*
ARHGAP12	*AKR1D1*	*CLN8*	*AQP4*
ARHGAP19	*AKR7A2*	*CLPX*	*AQR*

ARHGAP20	*AKT2*	*CLU*	*AR*
ARHGAP28	*AKT3*	*CLYBL*	*ARAF*
ARHGAP40	*AKTIP*	*CMBL*	*ARAP2*
ARHGAP42	*ALCAM*	*CMTM6*	*AREL1*
ARHGEF10	*ALDH16A1*	*CNBP*	*ARF1*
ARHGEF12	*ALDH1A2*	*CNDP2*	*ARF3*
ARHGEF17	*ALDH1A3*	*CNTNAP5*	*ARF4*
ARHGEF37	*ALDH1L2*	*COG5*	*ARF5*
ARHGEF38	*ALDH3A2*	*COG6*	*ARF6*
ARHGEF39	*ALDH5A1*	*COL26A1*	*ARFGEF1*
ARHGEF4	*ALDH6A1*	*COL4A1*	*ARFGEF3*
ARHGEF7	*ALDH7A1*	*COMMD2*	*ARFIP1*
ARID1A	*ALDH8A1*	*COMTD1*	*ARGLU1*
ARID2	*ALDH9A1*	*COPS7B*	*ARHGAP11A*
ARIH2	*ALG1*	*COQ2*	*ARHGAP12*
ARL1	*ALG10*	*CORO6*	*ARHGAP19*
ARL10	*ALG10B*	*COX10*	*ARHGAP20*
ARL11	*ALG13*	*COX5A*	*ARHGAP28*
ARL13B	*ALG14*	*CPEB2*	*ARHGAP31*
ARL14EP	*ALG5*	*CPNE5*	*ARHGAP40*
ARL14EPL	*ALG6*	*CPSF2*	*ARHGAP42*
ARL17A	*ALG8*	*CREBBP*	*ARHGAP5*
ARL17B	*ALG9*	*CREG2*	*ARHGEF10*
ARL2BP	*ALKAL2*	*CRIPT*	*ARHGEF12*
ARL4A	*ALKBH1*	*CRLS1*	*ARHGEF17*
ARL4D	*ALKBH8*	*CROCC*	*ARHGEF18*
ARL5A	*ALOX12*	*CRTC1*	*ARHGEF37*
ARL6	*ALOX15*	*CSNK1E*	*ARHGEF38*
ARL6IP1	*ALPG*	*CSTF2*	*ARHGEF39*
ARL6IP5	*ALPK2*	*CTC1*	*ARHGEF4*
ARMC1	*ALPK3*	*CTDSP2*	*ARHGEF7*
ARMC10	*ALPP*	*CTDSPL*	*ARID1A*
ARMC3	*ALS2*	*CTDSPL2*	*ARID2*
ARMCX3	*ALX1*	*CTU1*	*ARID3A*
ARMH3	*ALX4*	*CUEDC1*	*ARIH2*
ARMT1	*AMACR*	*CUL1*	*ARL1*
ARNT	*AMD1*	*CUL2*	*ARL10*
ARNTL2	*AMDHD1*	*CUL5*	*ARL11*
ARPC2	*AMER2*	*CUX1*	*ARL13B*
ARPC4-TTLL3	*AMER3*	*CXCL16*	*ARL14EP*
ARPIN	*AMFR*	*CXCR5*	*ARL14EPL*
ARPP19	*AMIGO2*	*CXorf56*	*ARL17A*
ARRDC2	*AMMECR1*	*CYHR1*	*ARL17B*
ARRDC4	*AMMECR1L*	*CYP1A2*	*ARL2BP*

ARSA	*AMN1*	*CYP20A1*	*ARL3*
ARSB	*AMOT*	*CYP26B1*	*ARL4A*
ARSE	*AMOTL2*	*CYP2W1*	*ARL4D*
ARSG	*AMPD3*	*CYTH1*	*ARL5A*
ARSK	*AMTN*	*CYYR1*	*ARL6*
ASB1	*AMZ1*	*DAPK3*	*ARL6IP1*
ASB4	*ANAPC1*	*DAZAP2*	*ARL6IP5*
ASB5	*ANAPC10*	*DBN1*	*ARMC1*
ASB6	*ANAPC7*	*DBNDD1*	*ARMC10*
ASB7	*ANGEL1*	*DBNL*	*ARMC3*
ASB9	*ANGEL2*	*DBT*	*ARMC6*
ASCC3	*ANGPT1*	*DCAF10*	*ARMC7*
ASCL4	*ANGPT2*	*DCAF5*	*ARMCX3*
ASPA	*ANGPT4*	*DCAF6*	*ARMH3*
ASPH	*ANGPTL1*	*DCBLD1*	*ARMT1*
ASTN1	*ANGPTL2*	*DCUN1D5*	*ARNT*
ASTN2	*ANGPTL3*	*DDAH1*	*ARNTL2*
ASXL1	*ANK1*	*DDIT4L*	*ARPC2*
ASXL3	*ANK3*	*DDRGK1*	*ARPC4-TTLL3*
ATAD2B	*ANKEF1*	*DDX21*	*ARPIN*
ATAT1	*ANKFY1*	*DDX4*	*ARPP19*
ATF2	*ANKH*	*DDX49*	*ARRDC2*
ATF3	*ANKHD1*	*DEAF1*	*ARRDC3*
ATF4	*ANKIB1*	*DELE1*	*ARRDC4*
ATF5	*ANKMY1*	*DENR*	*ARSB*
ATF6	*ANKMY2*	*DESI2*	*ARSD*
ATF7	*ANKRA2*	*DFFB*	*ARSE*
ATF7IP2	*ANKRD11*	*DGCR2*	*ARSG*
ATG12	*ANKRD12*	*DHRS13*	*ARSK*
ATG13	*ANKRD13A*	*DHX33*	*ASAH2*
ATG16L1	*ANKRD13B*	*DHX40*	*ASB4*
ATG2B	*ANKRD13C*	*DIAPH2*	*ASB5*
ATG3	*ANKRD16*	*DIP2A*	*ASB6*
ATG4A	*ANKRD17*	*DIPK2B*	*ASB7*
ATG7	*ANKRD18A*	*DLG5*	*ASB9*
ATIC	*ANKRD18B*	*DLX3*	*ASCC3*
ATL2	*ANKRD20A2*	*DLX4*	*ASCL4*
ATL3	*ANKRD20A4*	*DMRT1*	*ASPA*
ATM	*ANKRD22*	*DNAJB4*	*ASPH*
ATN1	*ANKRD28*	*DNAJB6*	*ASTN1*
ATOH8	*ANKRD29*	*DNAJB7*	*ASTN2*
ATP10D	*ANKRD31*	*DNAJC11*	*ASXL2*
ATP11A	*ANKRD33B*	*DNAJC15*	*ASXL3*
ATP11B	*ANKRD34B*	*DNAJC28*	*ASZ1*

ATP13A2	*ANKRD34C*	*DNAJC5*	*ATAD2B*
ATP1A1	*ANKRD37*	*DNASE2*	*ATAT1*
ATP1B2	*ANKRD42*	*DNM3*	*ATF2*
ATP1B4	*ANKRD44*	*DNMT1*	*ATF3*
ATP2A3	*ANKRD45*	*DOCK8-AS1*	*ATF4*
ATP2B3	*ANKRD46*	*DOLK*	*ATF6*
ATP2B4	*ANKRD49*	*DOT1L*	*ATF7*
ATP4A	*ANKRD50*	*DPH7*	*ATF7IP2*
ATP5MC1	*ANKRD52*	*DPM3*	*ATG12*
ATP5MC3	*ANKRD6*	*DPY19L3*	*ATG16L1*
ATP6AP1	*ANKRD66*	*DPY19L4*	*ATG2B*
ATP6V0A2	*ANKS1B*	*DPYSL2*	*ATG3*
ATP6V0D2	*ANKS3*	*DRG1*	*ATG4A*
ATP6V0E1	*ANKS4B*	*DUSP1*	*ATG7*
ATP6V1E1	*ANKS6*	*DUSP4*	*ATIC*
ATP6V1G2	*ANO3*	*DUSP9*	*ATL2*
ATP7A	*ANO5*	*DUXA*	*ATL3*
ATP7B	*ANO6*	*DVL3*	*ATM*
ATP8A1	*ANO7*	*DYNC1LI2*	*ATOH8*
ATP8B4	*ANO8*	*DYRK1A*	*ATP10D*
ATPAF2	*ANOS1*	*DZIP1L*	*ATP11A*
ATRN	*ANP32A*	*E2F2*	*ATP11B*
ATRNL1	*ANP32E*	*E2F7*	*ATP13A2*
ATRX	*ANTXR1*	*ECEL1*	*ATP1A1*
ATXN1	*ANTXR2*	*EDC3*	*ATP1B2*
ATXN1L	*ANXA11*	*EDEM1*	*ATP1B4*
ATXN7L1	*ANXA4*	*EDRF1*	*ATP2A2*
ATXN7L3	*ANXA5*	*EEF1A1*	*ATP2A3*
ATXN7L3B	*ANXA6*	*EEF2K*	*ATP2B3*
AUTS2	*AOC3*	*EFCAB13*	*ATP2B4*
AVL9	*AOPEP*	*EFCAB2*	*ATP4A*
AXIN2	*AOX1*	*EFNA1*	*ATP5MC1*
AZI2	*AP1AR*	*EFNB2*	*ATP5MC3*
B3GALNT2	*AP1G1*	*EGR2*	*ATP5PB*
B3GALT5	*AP1S1*	*EGR4*	*ATP6AP1*
B3GAT2	*AP1S2*	*EHD1*	*ATP6V0A2*
B3GLCT	*AP1S3*	*EIF2AK2*	*ATP6V0E1*
B3GNT3	*AP3B1*	*EIF2B5*	*ATP6V1E1*
B3GNT4	*AP3B2*	*EIF2S3*	*ATP6V1G2*
B3GNT5	*AP3M1*	*EIF5A*	*ATP7A*
B3GNT7	*AP3S2*	*EIF5AL1*	*ATP8A1*
B4GALNT4	*AP4E1*	*EIF5B*	*ATP8A2*
B4GALT7	*AP4S1*	*ELAC1*	*ATP8B4*
BAALC	*AP5B1*	*ELF4*	*ATPAF2*

BAALC-AS2	*AP5M1*	*ELF5*	*ATRN*
BACE2	*APAF1*	*ELOVL6*	*ATRX*
BAG2	*APBA1*	*ELP2*	*ATXN1*
BAG3	*APBB1IP*	*EML6*	*ATXN1L*
BAG5	*APBB2*	*ENTPD5*	*ATXN7*
BAG6	*APCDD1*	*ENTPD6*	*ATXN7L1*
BAHD1	*APEH*	*EPB41L1*	*ATXN7L3*
BAIAP2	*APH1B*	*EPC2*	*ATXN7L3B*
BAMBI	*API5*	*EPDR1*	*AUTS2*
BARD1	*APLF*	*EPHA7*	*AVL9*
BAZ2B	*APLP1*	*EPHX2*	*AXIN2*
BBC3	*APOA1*	*EPHX3*	*AZI2*
BBS12	*APOB*	*EPN2*	*AZIN1*
BBS4	*APOBEC3C*	*EPSTI1*	*B2M*
BBS7	*APOBEC3F*	*ERC1*	*B3GALNT1*
BBX	*APOBEC3G*	*ERGIC1*	*B3GALNT2*
BCAT1	*APOBEC4*	*ERI1*	*B3GALT5*
BCHE	*APOL3*	*ESF1*	*B3GAT2*
BCL10	*APOL4*	*ESR1*	*B3GLCT*
BCL11A	*APOL6*	*ETFDH*	*B3GNT3*
BCL11B	*APOLD1*	*ETV3*	*B3GNT4*
BCL2L11	*APOOL*	*EVI2B*	*B3GNT5*
BCL2L13	*APP*	*EWSR1*	*B3GNT7*
BCL2L14	*APPBP2*	*EXOC3*	*B4GALNT4*
BCL2L15	*APPL1*	*EXOC3-AS1*	*B4GALT2*
BCL2L2	*APPL2*	*EXOC4*	*B4GALT7*
BCL2L2-PABPN1	*APTX*	*F11R*	*BAALC*
BCL7B	*AQP1*	*FAAP20*	*BAALC-AS2*
BCL7C	*AQP2*	*FABP2*	*BACE2*
BCL9	*AQP4*	*FAHD1*	*BACH1*
BCLAF1	*AQP7*	*FAIM2*	*BACH2*
BDNF	*AQP9*	*FAM102A*	*BAG2*
BDP1	*AQR*	*FAM110D*	*BAG3*
BEAN1	*AR*	*FAM111A*	*BAG5*
BEGAIN	*AREL1*	*FAM118A*	*BAHD1*
BEND4	*ARF1*	*FAM120AOS*	*BAIAP2*
BEND6	*ARF3*	*FAM126B*	*BAMBI*
BEST3	*ARF6*	*FAM155B*	*BARD1*
BFSP2	*ARFGAP1*	*FAM161A*	*BAZ1A*
BHLHE40	*ARFGAP3*	*FAM163A*	*BAZ2B*
BICD1	*ARFGEF1*	*FAM204A*	*BBC3*
BICD2	*ARFGEF2*	*FAM219A*	*BBS12*
BICDL1	*ARFGEF3*	*FAM221A*	*BBS4*
BIVM	*ARFIP1*	*FAM49A*	*BBS7*

BLOC1S3	*ARGLU1*	*FAM53A*	*BBX*
BLOC1S4	*ARHGAP1*	*FAM83B*	*BCAS2*
BLOC1S5	*ARHGAP11A*	*FANCC*	*BCAS3*
BLVRA	*ARHGAP12*	*FARP1*	*BCAT1*
BMERB1	*ARHGAP18*	*FARSB*	*BCHE*
BMF	*ARHGAP19*	*FASN*	*BCL10*
BMI1	*ARHGAP20*	*FBXL15*	*BCL11A*
BMP10	*ARHGAP24*	*FBXL18*	*BCL11B*
BMP2K	*ARHGAP28*	*FBXL20*	*BCL2L11*
BMP3	*ARHGAP29*	*FBXL4*	*BCL2L13*
BMP6	*ARHGAP30*	*FBXO41*	*BCL2L14*
BMP8A	*ARHGAP31*	*FBXW2*	*BCL2L15*
BMP8B	*ARHGAP32*	*FCN2*	*BCL2L2*
BMPR1A	*ARHGAP44*	*FCRL2*	*BCL2L2-PABPN1*
BMPR2	*ARHGAP5*	*FGF11*	*BCL6*
BMT2	*ARHGEF10*	*FGF17*	*BCL7B*
BNC2	*ARHGEF12*	*FGFBP1*	*BCL7C*
BNIP2	*ARHGEF26*	*FGFR3*	*BCL9*
BNIP3	*ARHGEF28*	*FIGN*	*BCLAF1*
BNIP3L	*ARHGEF3*	*FKBP5*	*BCLAF3*
BOD1	*ARHGEF38*	*FKRP*	*BDNF*
BOLL	*ARHGEF39*	*FLT1*	*BDP1*
BORCS5	*ARHGEF4*	*FLVCR2*	*BEAN1*
BORCS6	*ARHGEF7*	*FNBP1*	*BECN1*
BORCS8	*ARID1A*	*FOSL2*	*BEGAIN*
BPNT1	*ARID2*	*FOXA1*	*BEND4*
BPTF	*ARID3A*	*FOXH1*	*BEND6*
BRAP	*ARID3B*	*FOXL2NB*	*BEST3*
BRCA1	*ARID4B*	*FOXN2*	*BFSP2*
BRD8	*ARID5B*	*FOXO3*	*BHLHE40*
BRF1	*ARIH1*	*FOXP1*	*BHLHE41*
BRINP3	*ARIH2*	*FPGT-TNNI3K*	*BICD1*
BRIX1	*ARL1*	*FRA10AC1*	*BICD2*
BRK1	*ARL10*	*FRAT1*	*BICDL1*
BRS3	*ARL11*	*FRMD1*	*BLOC1S4*
BRSK2	*ARL13B*	*FRMD8*	*BLOC1S5*
BRWD1	*ARL14*	*FRRS1*	*BLVRA*
BRWD3	*ARL14EP*	*FSTL4*	*BMERB1*
BSDC1	*ARL14EPL*	*FUBP1*	*BMF*
BSPRY	*ARL17A*	*FUS*	*BMI1*
BTAF1	*ARL17B*	*FUT1*	*BMP10*
BTBD7	*ARL4A*	*FUT5*	*BMP2K*
BTBD9	*ARL4C*	*FUT7*	*BMP3*
BTD	*ARL5A*	*FXN*	*BMP6*

BTF3L4	*ARL5B*	*FXR1*	*BMP8A*
BTLA	*ARL5C*	*FZD1*	*BMP8B*
BTN3A1	*ARL6*	*FZD2*	*BMPER*
BTN3A2	*ARL6IP1*	*FZD5*	*BMPR1A*
BTN3A3	*ARL6IP6*	*FZD7*	*BMPR2*
BTNL8	*ARMC1*	*FZD8*	*BMT2*
BTNL9	*ARMC10*	*G6PC2*	*BNC2*
BTRC	*ARMC12*	*G6PC3*	*BNIP2*
BZW1	*ARMC3*	*GABRA4*	*BNIP3*
C10orf105	*ARMC7*	*GABRP*	*BNIP3L*
C10orf67	*ARMC8*	*GAD2*	*BOD1*
C11orf1	*ARMCX2*	*GAGE1*	*BORCS5*
C11orf54	*ARMH4*	*GALNT11*	*BORCS6*
C11orf74	*ARMT1*	*GAS8*	*BORCS8*
C11orf87	*ARNT*	*GATA6*	*BPNT1*
C11orf98	*ARNT2*	*GATAD2B*	*BRAP*
C12orf40	*ARNTL*	*GBP4*	*BRCA1*
C12orf49	*ARNTL2*	*GBX2*	*BRD1*
C12orf65	*ARPC1A*	*GCSH*	*BRD8*
C12orf74	*ARPC2*	*GDE1*	*BRINP3*
C14orf132	*ARPC4-TTLL3*	*GDF11*	*BRIX1*
C15orf61	*ARPC5*	*GDF6*	*BRK1*
C16orf54	*ARPC5L*	*GDF7*	*BRMS1L*
C16orf72	*ARPIN*	*GFM1*	*BRS3*
C16orf95	*ARPIN-AP3S2*	*GFOD1*	*BRSK2*
C17orf75	*ARPP19*	*GHRHR*	*BRWD1*
C18orf21	*ARPP21*	*GID4*	*BRWD3*
C18orf25	*ARRB1*	*GIMAP6*	*BSDC1*
C18orf54	*ARRDC1-AS1*	*GIPC3*	*BSN*
C19orf12	*ARRDC2*	*GIPR*	*BSPRY*
C1GALT1	*ARRDC3*	*GLB1L*	*BTBD7*
C1orf115	*ARRDC4*	*GLG1*	*BTD*
C1orf116	*ARSA*	*GLIPR2*	*BTF3L4*
C1orf198	*ARSB*	*GMDS*	*BTLA*
C1orf210	*ARSD*	*GMEB2*	*BTN3A1*
C1orf216	*ARSE*	*GMPR*	*BTN3A2*
C1orf226	*ARSG*	*GMPS*	*BTN3A3*
C1orf35	*ARSJ*	*GNA13*	*BTNL8*
C1orf43	*ARSK*	*GNAO1*	*BTRC*
C1orf52	*ART3*	*GNB1L*	*BZW1*
C1orf68	*ART4*	*GNE*	*C10orf105*
C1QTNF1-AS1	*ASAH2*	*GNG4*	*C10orf67*
C1QTNF3	*ASAH2B*	*GNG5*	*C10orf82*
C1RL	*ASAP1*	*GNL3L*	*C11orf1*

C20orf194	ASB1	GNPTG	C11orf21
C21orf58	ASB14	GOLGA3	C11orf54
C21orf91	ASB15	GOLGA7B	C11orf74
C2CD2	ASB4	GOSR1	C11orf87
C2CD2L	ASB5	GP6	C11orf98
C2CD4A	ASB7	GPAT4	C12orf40
C2CD4C	ASB8	GPATCH2L	C12orf49
C2orf49	ASCC1	GPI	C12orf66
C2orf50	ASCC3	GPLD1	C12orf74
C2orf68	ASDURF	GPR12	C14orf132
C2orf72	ASF1B	GPR150	C15orf40
C2orf80	ASNA1	GPR151	C15orf61
C3	ASNSD1	GPR157	C16orf54
C3orf14	ASPA	GPR180	C16orf72
C3orf67	ASPH	GPR26	C16orf87
C3orf70	ASPHD2	GPR55	C16orf95
C4orf33	ASPN	GPR63	C17orf75
C4orf46	ASPSCR1	GPRC5A	C18orf21
C4orf54	ASRGL1	GPRC5B	C18orf54
C5AR2	ASTN1	GRAMD1B	C19orf12
C5orf15	ASTN2	GRAP2	C19orf47
C5orf22	ASXL1	GRB2	C1GALT1
C5orf24	ASXL2	GRHL1	C1GALT1C1
C5orf30	ASXL3	GRIK3	C1orf115
C5orf46	ATAD1	GRM6	C1orf116
C5orf51	ATAD2	GRWD1	C1orf198
C5orf63	ATAD2B	GTDC1	C1orf216
C6	ATAD5	GTF2F1	C1orf226
C6orf118	ATAT1	GTF2H1	C1orf35
C6orf120	ATCAY	GTF3C1	C1orf43
C6orf141	ATE1	GTF3C4	C1orf52
C6orf89	ATF2	GTPBP1	C1QTNF1-AS1
C8A	ATF5	GUCY1A1	C1QTNF3
C8orf31	ATF6	GZMM	C1RL
C8orf33	ATF7	H2AFV	C20orf194
C8orf34-AS1	ATF7IP	H2AFY	C21orf58
C8orf37	ATG10	H3F3A	C21orf91
C8orf44-SGK3	ATG12	HACD3	C2CD2
C9orf135	ATG13	HAS2	C2CD2L
C9orf153	ATG14	HBS1L	C2CD4A
C9orf47	ATG16L1	HCAR1	C2CD4C
C9orf64	ATG2B	HCAR3	C2orf49
C9orf72	ATG3	HDAC2	C2orf50
CA12	ATG5	HDAC3	C2orf68

CA13	*ATG7*	*HDHD2*	*C2orf72*
CA5B	*ATIC*	*HDLBP*	*C2orf80*
CA8	*ATL1*	*HEATR3*	*C3*
CABLES1	*ATL2*	*HECTD1*	*C3orf67*
CABP1	*ATL3*	*HENMT1*	*C3orf70*
CABYR	*ATM*	*HES7*	*C4orf33*
CACNA1C	*ATMIN*	*HEXA*	*C4orf46*
CACNB1	*ATN1*	*HEXB*	*C4orf50*
CACNB3	*ATOH7*	*HEXIM2*	*C4orf54*
CACNB4	*ATOH8*	*HHAT*	*C5AR2*
CACNG8	*ATP10A*	*HHIPL1*	*C5orf15*
CACUL1	*ATP10B*	*HIF3A*	*C5orf22*
CADM1	*ATP10D*	*HIP1*	*C5orf24*
CADM2	*ATP11A*	*HIPK2*	*C5orf30*
CALB1	*ATP11B*	*HIST1H4C*	*C5orf46*
CALB2	*ATP11C*	*HIST2H2AA3*	*C5orf51*
CALCOCO2	*ATP1A2*	*HIST2H4B*	*C5orf63*
CALCRL	*ATP1B4*	*HIST4H4*	*C6*
CALHM3	*ATP2B1*	*HJURP*	*C6orf118*
CALHM4	*ATP2B3*	*HLA-DOA*	*C6orf120*
CALHM5	*ATP2B4*	*HLCS*	*C6orf141*
CALM1	*ATP2C1*	*HLTF*	*C6orf58*
CALM3	*ATP5F1A*	*HMGB1*	*C6orf89*
CALN1	*ATP5F1E*	*HMGB2*	*C8A*
CALU	*ATP5IF1*	*HMGN1*	*C8orf31*
CAMK4	*ATP5MC1*	*HNRNPF*	*C8orf33*
CAMKK1	*ATP5MC3*	*HOMEZ*	*C8orf34-AS1*
CAMKK2	*ATP5MG*	*HOXA1*	*C8orf37*
CAMSAP2	*ATP5MPL*	*HOXA13*	*C8orf44-SGK3*
CAMTA1	*ATP5PB*	*HOXA2*	*C9orf135*
CAND1	*ATP5PF*	*HOXC5*	*C9orf153*
CANX	*ATP6V0A2*	*HOXD13*	*C9orf47*
CAP1	*ATP6V0D1*	*HS1BP3*	*C9orf64*
CAPN11	*ATP6V0D2*	*HS3ST2*	*C9orf72*
CAPN5	*ATP6V0E1*	*HS3ST3B1*	*CA12*
CAPN6	*ATP6V0E2*	*HS6ST1*	*CA13*
CAPN7	*ATP6V1A*	*HSD17B13*	*CA8*
CAPN8	*ATP6V1C2*	*HSP90B1*	*CABLES1*
CAPN9	*ATP6V1E1*	*HSPA12B*	*CABP1*
CAPRIN1	*ATP6V1G1*	*HSPA14*	*CABYR*
CAPRIN2	*ATP7A*	*HSPA6*	*CACNA1C*
CAPS2	*ATP7B*	*HSPBP1*	*CACNB1*
CAPZA2	*ATP8A1*	*IARS*	*CACNB3*
CARD8	*ATP8A2*	*ICMT*	*CACNB4*

CARF	*ATP8B2*	*ICOSLG*	*CACNG8*
CARM1	*ATP8B4*	*ID1*	*CACUL1*
CASC3	*ATP9B*	*IDS*	*CADM1*
CASC4	*ATPAF2*	*IER5*	*CADM2*
CASD1	*ATR*	*IFFO2*	*CADM3*
CASK	*ATRN*	*IFIT2*	*CALB1*
CASKIN1	*ATRNL1*	*IFIT3*	*CALB2*
CASKIN2	*ATRX*	*IFNAR1*	*CALCOCO2*
CASP10	*ATXN1*	*IFNE*	*CALCRL*
CASP3	*ATXN1L*	*IFT22*	*CALHM3*
CASP9	*ATXN7*	*IFT74*	*CALHM4*
CASQ2	*ATXN7L1*	*IGF1R*	*CALHM5*
CASR	*ATXN7L3B*	*IGF2*	*CALM1*
CAST	*AURKC*	*IGF2R*	*CALML4*
CASTOR3	*AUTS2*	*IGFN1*	*CALN1*
CASZ1	*AVL9*	*IKZF4*	*CALR*
CAV2	*AVPI1*	*IL17D*	*CALU*
CAVIN3	*AVPR1A*	*IL17RA*	*CAMK2D*
CAVIN4	*AWAT1*	*IL22RA1*	*CAMK4*
CBFB	*AXL*	*IL3RA*	*CAMKK1*
CBL	*AZI2*	*IL6R*	*CAMKK2*
CBLB	*AZIN1*	*ILRUN*	*CAMSAP2*
CBLL1	*B3GALNT1*	*IMPDH1*	*CAMTA1*
CBLN2	*B3GALNT2*	*IMPG2*	*CAND1*
CBR4	*B3GALT1*	*INHBB*	*CANX*
CBWD1	*B3GALT2*	*INO80E*	*CAP1*
CBWD2	*B3GALT5*	*INPP4A*	*CAPN11*
CBWD3	*B3GALT6*	*INPP5E*	*CAPN5*
CBWD5	*B3GAT2*	*INSIG1*	*CAPN6*
CBWD6	*B3GLCT*	*INSM1*	*CAPN7*
CBX2	*B3GNT2*	*INTS6L*	*CAPN8*
CBX5	*B3GNT5*	*IP6K1*	*CAPN9*
CBY1	*B4GALT1*	*IPPK*	*CAPRIN1*
CC2D1B	*B4GALT5*	*IQCK*	*CAPRIN2*
CCDC113	*B4GALT6*	*IRAK3*	*CAPS2*
CCDC114	*B4GALT7*	*IREB2*	*CAPZA2*
CCDC117	*B9D1*	*IRF2BP2*	*CARD8*
CCDC121	*BAALC*	*IRF8*	*CARF*
CCDC125	*BAAT*	*ISL2*	*CARM1*
CCDC126	*BABAM2*	*ISY1-RAB43*	*CASC4*
CCDC127	*BACE1*	*ITGA3*	*CASD1*
CCDC13	*BACE2*	*ITGA4*	*CASK*
CCDC141	*BACH1*	*ITM2B*	*CASKIN1*
CCDC148	*BACH2*	*ITPK1*	*CASP10*

CCDC150	*BAG2*	*ITPRIP*	*CASP16P*
CCDC155	*BAG3*	*ITSN1*	*CASP3*
CCDC171	*BAG4*	*JAGN1*	*CASP9*
CCDC173	*BAG5*	*JDP2*	*CASQ2*
CCDC185	*BAHCC1*	*JMJD4*	*CASR*
CCDC186	*BAHD1*	*JPH1*	*CAST*
CCDC198	*BAIAP2*	*JPH3*	*CASTOR3*
CCDC33	*BAMBI*	*JPH4*	*CASZ1*
CCDC36	*BANF1*	*JPT2*	*CAV2*
CCDC47	*BARD1*	*KAAG1*	*CAVIN3*
CCDC50	*BATF2*	*KANK1*	*CAVIN4*
CCDC71L	*BAZ1B*	*KARS*	*CBFB*
CCDC80	*BAZ2A*	*KATNBL1*	*CBL*
CCDC85C	*BAZ2B*	*KBTBD11*	*CBLB*
CCDC88A	*BBOF1*	*KBTBD8*	*CBLL1*
CCL22	*BBS10*	*KCMF1*	*CBLN1*
CCL28	*BBS7*	*KCNA7*	*CBLN2*
CCM2L	*BBS9*	*KCNAB2*	*CBR4*
CCN1	*BBX*	*KCNG4*	*CBWD1*
CCN2	*BCAP29*	*KCNJ11*	*CBWD2*
CCN3	*BCAR3*	*KCNJ5*	*CBWD3*
CCN4	*BCAS4*	*KCNV1*	*CBWD5*
CCNB1	*BCAT1*	*KCTD10*	*CBWD6*
CCNC	*BCCIP*	*KCTD12*	*CBX2*
CCND1	*BCDIN3D*	*KCTD15*	*CBX5*
CCND2	*BCHE*	*KDELR2*	*CC2D1B*
CCNG2	*BCKDHB*	*KDM5A*	*CCDC113*
CCNL2	*BCL10*	*KDM6B*	*CCDC114*
CCNT2	*BCL11A*	*KIAA1191*	*CCDC117*
CCNYL1	*BCL11B*	*KIAA1257*	*CCDC121*
CCP110	*BCL2*	*KIAA1549*	*CCDC125*
CCR1	*BCL2L11*	*KIAA1614*	*CCDC126*
CCR6	*BCL2L13*	*KIF1A*	*CCDC127*
CCR7	*BCL2L15*	*KIF1B*	*CCDC13*
CCS	*BCL2L2*	*KIF3C*	*CCDC134*
CCSER1	*BCL6B*	*KLC1*	*CCDC141*
CCSER2	*BCL7A*	*KLF3*	*CCDC142*
CCT4	*BCL7C*	*KLF8*	*CCDC148*
CCT5	*BCLAF1*	*KLHL15*	*CCDC150*
CCT6B	*BCLAF3*	*KLHL36*	*CCDC155*
CCT7	*BCO2*	*KMO*	*CCDC160*
CD164	*BCOR*	*KMT2D*	*CCDC170*
CD180	*BCORL1*	*KPNA2*	*CCDC171*
CD1C	*BDH1*	*KREMEN1*	*CCDC173*

CD226	*BDH2*	*KRTAP10-1*	*CCDC185*
CD28	*BDNF*	*KRTAP4-9*	*CCDC186*
CD2AP	*BDP1*	*L2HGDH*	*CCDC198*
CD2BP2	*BEND2*	*LAMA5*	*CCDC25*
CD300A	*BEND3*	*LDHB*	*CCDC30*
CD300E	*BEND4*	*LEF1*	*CCDC33*
CD36	*BEND6*	*LEPR*	*CCDC36*
CD38	*BEST1*	*LEPROT*	*CCDC47*
CD4	*BEST3*	*LETM1*	*CCDC50*
CD44	*BET1*	*LGALS1*	*CCDC57*
CD47	*BET1L*	*LGR6*	*CCDC71*
CD55	*BHLHE22*	*LHFPL2*	*CCDC71L*
CD68	*BHLHE40*	*LHPP*	*CCDC80*
CD79A	*BHLHE41*	*LHX4*	*CCDC85C*
CD83	*BHMT*	*LIF*	*CCDC88A*
CD84	*BHMT2*	*LIN28B*	*CCDC93*
CD93	*BICC1*	*LINC01124*	*CCL22*
CD99L2	*BICD1*	*LINC02312*	*CCL28*
CDAN1	*BICD2*	*LIX1L*	*CCM2L*
CDC27	*BICRAL*	*LMBR1*	*CCN1*
CDC34	*BID*	*LMLN*	*CCN2*
CDC37L1	*BIRC2*	*LMO3*	*CCN3*
CDC42BPA	*BIRC3*	*LNPEP*	*CCN4*
CDC42EP3	*BIRC5*	*LOC100129215*	*CCNB1*
CDC42EP4	*BIVM*	*LOC105375614*	*CCNC*
CDC42SE2	*BLID*	*LONRF3*	*CCND1*
CDC5L	*BLMH*	*LPIN2*	*CCND2*
CDC6	*BLNK*	*LPP*	*CCNG2*
CDC73	*BLOC1S3*	*LRFN2*	*CCNJ*
CDCA2	*BLOC1S4*	*LRIG2*	*CCNL2*
CDCA7L	*BLOC1S5*	*LRP2*	*CCNT2*
CDH1	*BLOC1S6*	*LRP4*	*CCNY*
CDH19	*BLVRA*	*LRP8*	*CCNYL1*
CDH2	*BMERB1*	*LRPAP1*	*CCP110*
CDH5	*BMI1*	*LRRC20*	*CCR1*
CDH6	*BMP1*	*LRRC31*	*CCR6*
CDHR2	*BMP10*	*LRRC32*	*CCR7*
CDK14	*BMP2*	*LRRC41*	*CCSAP*
CDK15	*BMP2K*	*LRRC8B*	*CCSER1*
CDK17	*BMP3*	*LRRFIP1*	*CCSER2*
CDK18	*BMP5*	*LRRN1*	*CCT3*
CDK19	*BMP6*	*LTBR*	*CCT4*
CDK2	*BMP8A*	*LTO1*	*CCT5*
CDK5R1	*BMPER*	*LUC7L*	*CCT6B*

CDK6	*BMPR1A*	*LURAP1*	*CCT7*
CDK8	*BMPR1B*	*LYRM4*	*CD164*
CDKL4	*BMPR2*	*LYZ*	*CD180*
CDKL5	*BMS1*	*MAATS1*	*CD1C*
CDKN1B	*BMT2*	*MAFG*	*CD1D*
CDKN1C	*BNC1*	*MAK*	*CD226*
CDKN2AIP	*BNC2*	*MAP2K3*	*CD28*
CDKN2B	*BNIP2*	*MAP2K7*	*CD2AP*
CDON	*BNIP3*	*MAP4*	*CD2BP2*
CDPF1	*BNIP3L*	*MAP7D1*	*CD300A*
CDR1	*BOD1*	*MAP9*	*CD300E*
CDR2L	*BOD1L1*	*MAPK13*	*CD36*
CDS1	*BOD1L2*	*MAPK1IP1L*	*CD38*
CDS2	*BOLA3*	*MAPK6*	*CD4*
CDV3	*BOLL*	*MAPKBP1*	*CD44*
CDY1	*BORCS5*	*MAPRE2*	*CD47*
CDY1B	*BORCS7*	*MARC1*	*CD55*
CDYL2	*BORCS8*	*MARCH4*	*CD79A*
CEACAM19	*BPIFB2*	*MARF1*	*CD83*
CEACAM21	*BPTF*	*MASP1*	*CD84*
CEBPD	*BRAP*	*MASTL*	*CD93*
CEBPG	*BRCA1*	*MAU2*	*CD99L2*
CELF1	*BRCC3*	*MBD3*	*CDAN1*
CELF4	*BRD4*	*MBIP*	*CDC14B*
CELF5	*BRD7*	*MBNL1*	*CDC25C*
CEMIP2	*BRI3*	*MBNL3*	*CDC27*
CENPA	*BRI3BP*	*MBOAT2*	*CDC34*
CENPC	*BRINP3*	*MBTD1*	*CDC37L1*
CENPF	*BRIP1*	*MCC*	*CDC42BPA*
CENPI	*BRIX1*	*MCFD2*	*CDC42EP3*
CENPJ	*BRMS1L*	*MCTP2*	*CDC42EP4*
CENPL	*BROX*	*MDH2*	*CDC42SE2*
CENPM	*BRPF1*	*MEAK7*	*CDC5L*
CENPN	*BRPF3*	*MECP2*	*CDC6*
CENPQ	*BRWD1*	*MED12*	*CDCA2*
CEP104	*BRWD3*	*MED7*	*CDCA7L*
CEP126	*BSN*	*MEF2D*	*CDH1*
CEP128	*BTAF1*	*METTL22*	*CDH11*
CEP162	*BTBD1*	*METTL24*	*CDH18*
CEP250	*BTBD10*	*MFAP2*	*CDH19*
CEP295	*BTBD17*	*MFHAS1*	*CDH2*
CEP350	*BTBD19*	*MFNG*	*CDH23*
CEP44	*BTBD7*	*MFSD6*	*CDH6*
CEP55	*BTBD9*	*MGA*	*CDH7*

CEP57	*BTC*	*MGAT1*	*CDHR2*
CEP57L1	*BTD*	*MGMT*	*CDK1*
CEP68	*BTF3*	*MIEF2*	*CDK14*
CEP70	*BTF3L4*	*MINDY2*	*CDK15*
CEP72	*BTG1*	*MIP*	*CDK17*
CEP78	*BTK*	*MIS12*	*CDK19*
CEP83	*BTLA*	*MITD1*	*CDK2*
CEP89	*BTN1A1*	*MKLN1*	*CDK2AP1*
CEP97	*BTN3A1*	*MLKL*	*CDK5R1*
CERCAM	*BTNL8*	*MMP13*	*CDK6*
CERS1	*BTNL9*	*MOCS3*	*CDK8*
CERS2	*BTRC*	*MORF4L2*	*CDKL4*
CERS5	*BUB1*	*MPP3*	*CDKN1B*
CETP	*BUB3*	*MPPED1*	*CDKN1C*
CFAP161	*BVES*	*MRC2*	*CDKN2AIP*
CFAP20	*BYSL*	*MRE11*	*CDKN2B*
CFAP298	*BZW1*	*MRGBP*	*CDO1*
CFAP299	*C10orf105*	*MRGPRF*	*CDON*
CFAP300	*C10orf53*	*MRPS14*	*CDPF1*
CFAP97	*C10orf67*	*MRPS23*	*CDR2L*
CFD	*C10orf88*	*MRPS33*	*CDS1*
CFHR3	*C10orf90*	*MS4A15*	*CDS2*
CFHR4	*C11orf1*	*MSANTD1*	*CDV3*
CFL2	*C11orf49*	*MSANTD3*	*CDY1*
CFLAR	*C11orf54*	*MSX2*	*CDY1B*
CGGBP1	*C11orf58*	*MTA3*	*CDYL2*
CGREF1	*C11orf65*	*MT-CO2*	*CEACAM19*
CHAF1B	*C11orf68*	*MT-CYB*	*CEBPD*
CHD1	*C11orf72*	*MTF1*	*CEBPG*
CHD2	*C11orf74*	*MTG1*	*CELF1*
CHD5	*C11orf87*	*MTMR1*	*CELF4*
CHD6	*C11orf91*	*MTMR3*	*CEMIP2*
CHD7	*C11orf98*	*MTMR8*	*CENPA*
CHD8	*C12orf29*	*MTO1*	*CENPBD1*
CHDH	*C12orf4*	*MTOR*	*CENPC*
CHERP	*C12orf40*	*MXRA8*	*CENPF*
CHGB	*C12orf43*	*MYADM*	*CENPH*
CHIC1	*C12orf49*	*MYCBP*	*CENPI*
CHKA	*C12orf50*	*MYCBP2*	*CENPJ*
CHMP3	*C12orf60*	*MYO9B*	*CENPL*
CHMP4B	*C12orf65*	*MYOM2*	*CENPM*
CHN1	*C12orf66*	*MYOZ3*	*CENPN*
CHORDC1	*C12orf73*	*MYPN*	*CENPP*
CHP1	*C12orf74*	*MZF1*	*CENPQ*

CHRAC1	*C12orf75*	*N4BP2*	*CENPT*
CHRDL1	*C14orf119*	*N4BP3*	*CEP104*
CHRM2	*C14orf28*	*N6AMT1*	*CEP126*
CHRM3	*C14orf39*	*NAA30*	*CEP128*
CHRNA5	*C15orf40*	*NAIP*	*CEP162*
CHRNA6	*C15orf41*	*NARF*	*CEP295*
CHRNE	*C15orf48*	*NCMAP*	*CEP350*
CHST11	*C15orf61*	*NCOR1*	*CEP44*
CHST3	*C15orf65*	*NCOR2*	*CEP55*
CHST7	*C16orf46*	*NCS1*	*CEP57*
CHSY1	*C16orf70*	*NDC1*	*CEP57L1*
CHSY3	*C16orf72*	*NDE1*	*CEP68*
CHTF8	*C16orf87*	*NDRG1*	*CEP70*
CHURC1	*C17orf107*	*NDST1*	*CEP72*
CHURC1-FNTB	*C17orf75*	*NDUFA10*	*CEP78*
CIAO2A	*C18orf21*	*NDUFA2*	*CEP83*
CIAPIN1	*C18orf54*	*NDUFA7*	*CEP89*
CILP	*C18orf63*	*NDUFA9*	*CEP97*
CIP2A	*C19orf12*	*NDUFC2*	*CERCAM*
CIPC	*C19orf47*	*NDUFV3*	*CERS1*
CISD1	*C1D*	*NEBL*	*CERS2*
CISD2	*C1GALT1*	*NEFM*	*CERS5*
CISH	*C1GALT1C1*	*NEIL2*	*CETP*
CIT	*C1orf109*	*NEU1*	*CFAP161*
CITED2	*C1orf112*	*NEU3*	*CFAP20*
CKAP2	*C1orf116*	*NF1*	*CFAP298*
CKAP5	*C1orf122*	*NFATC4*	*CFAP299*
CKS1B	*C1orf131*	*NFE2L3*	*CFAP300*
CLASP1	*C1orf141*	*NFIA*	*CFAP44*
CLASP2	*C1orf162*	*NFIB*	*CFAP97*
CLCC1	*C1orf198*	*NFYA*	*CFD*
CLCN3	*C1orf21*	*NHLRC2*	*CFHR3*
CLCN5	*C1orf210*	*NHSL1*	*CFHR4*
CLDN1	*C1orf35*	*NIBAN1*	*CFL2*
CLDN11	*C1orf50*	*NIFK*	*CFLAR*
CLDN12	*C1orf52*	*NIP7*	*CGGBP1*
CLDN16	*C1QL3*	*NIPAL1*	*CGREF1*
CLDN19	*C1QTNF3*	*NIPAL2*	*CHAF1B*
CLDN22	*C1QTNF7*	*NIPAL3*	*CHCHD2*
CLDN23	*C1QTNF9*	*NIPBL*	*CHD1*
CLDND1	*C1RL*	*NIT1*	*CHD2*
CLEC16A	*C20orf144*	*NKAIN1*	*CHD5*
CLEC17A	*C20orf187*	*NKD1*	*CHD6*
CLEC1A	*C20orf194*	*NKRF*	*CHD7*

CLEC4E	*C20orf202*	*NKTR*	*CHD8*
CLEC4F	*C21orf58*	*NKX6-2*	*CHDH*
CLEC4M	*C21orf91*	*NLRP2*	*CHERP*
CLEC5A	*C22orf23*	*NLRP3*	*CHGB*
CLGN	*C22orf42*	*NME6*	*CHIC1*
CLIC2	*C2CD2*	*NOL4L*	*CHID1*
CLIC4	*C2CD2L*	*NOL9*	*CHKA*
CLIC5	*C2CD3*	*NOP2*	*CHML*
CLINT1	*C2CD4A*	*NOS1*	*CHMP3*
CLIP4	*C2CD6*	*NOTCH1*	*CHMP4B*
CLMN	*C2orf42*	*NOVA2*	*CHN1*
CLN5	*C2orf66*	*NOX4*	*CHORDC1*
CLN8	*C2orf68*	*NPC1*	*CHP1*
CLNK	*C2orf69*	*NPEPPS*	*CHP2*
CLPB	*C2orf72*	*NPHS1*	*CHRAC1*
CLSPN	*C2orf73*	*NPR3*	*CHRDL1*
CLSTN1	*C2orf80*	*NR1D2*	*CHRFAM7A*
CLSTN2	*C2orf88*	*NR6A1*	*CHRM2*
CLU	*C3*	*NRBP2*	*CHRM3*
CLVS2	*C3orf14*	*NRIP3*	*CHRNA4*
CLYBL	*C3orf38*	*NSF*	*CHRNA5*
CMBL	*C3orf67*	*NT5C1A*	*CHRNA6*
CMC1	*C3orf70*	*NT5DC3*	*CHRNE*
CMIP	*C3orf80*	*NTAN1*	*CHST10*
CMSS1	*C4orf19*	*NTN1*	*CHST11*
CMTM3	*C4orf3*	*NTPCR*	*CHST14*
CMTM4	*C4orf33*	*NTRK2*	*CHST3*
CMTM5	*C4orf36*	*NTRK3*	*CHST7*
CMTM6	*C4orf45*	*NTSR1*	*CHSY1*
CMTM8	*C4orf46*	*NUDCD2*	*CHSY3*
CNBP	*C4orf50*	*NUDT15*	*CHTF8*
CNDP2	*C4orf54*	*NUDT16*	*CHURC1*
CNIH3	*C5*	*NUDT16L1*	*CHURC1-FNTB*
CNKSR3	*C5AR1*	*NUDT19*	*CIAO2A*
CNNM2	*C5AR2*	*NUFIP2*	*CIAPIN1*
CNNM3	*C5orf24*	*NUGGC*	*CILP*
CNNM4	*C5orf30*	*NUP188*	*CINP*
CNOT1	*C5orf38*	*NUP62CL*	*CIP2A*
CNOT2	*C5orf46*	*NUTF2*	*CISD1*
CNOT4	*C5orf47*	*NXF1*	*CISD2*
CNOT6	*C5orf49*	*NXN*	*CIT*
CNOT6L	*C5orf51*	*NXPH3*	*CITED2*
CNOT7	*C5orf63*	*ODC1*	*CKAP5*
CNP	*C5orf64*	*ODF2L*	*CKS1B*

CNPY3	*C6orf118*	*ONECUT2*	*CLASP1*
CNPY4	*C6orf120*	*OPA3*	*CLASP2*
CNR1	*C6orf132*	*OPN3*	*CLCC1*
CNR2	*C6orf136*	*OPRM1*	*CLCN3*
CNST	*C6orf141*	*ORC2*	*CLCN5*
CNTD1	*C6orf223*	*ORC5*	*CLDN1*
CNTF	*C6orf58*	*ORC6*	*CLDN11*
CNTLN	*C6orf62*	*ORMDL1*	*CLDN12*
CNTN1	*C6orf89*	*ORMDL2*	*CLDN14*
CNTN2	*C7*	*OSBP2*	*CLDN16*
CNTNAP5	*C7orf25*	*OSBPL2*	*CLDN19*
COA5	*C7orf26*	*OSGEPL1*	*CLDN22*
COA7	*C8A*	*OSTF1*	*CLDND1*
COBL	*C8orf31*	*OTUD3*	*CLEC12B*
COBLL1	*C8orf33*	*OTULIN*	*CLEC16A*
COG2	*C8orf34-AS1*	*OTULINL*	*CLEC17A*
COG5	*C8orf37*	*OXA1L*	*CLEC1A*
COG6	*C8orf44-SGK3*	*OXCT1*	*CLEC4C*
COIL	*C8orf86*	*P2RX1*	*CLEC4E*
COL20A1	*C8orf88*	*P2RX5*	*CLEC4F*
COL21A1	*C9orf116*	*PABPN1*	*CLEC4M*
COL26A1	*C9orf152*	*PADI2*	*CLEC5A*
COL27A1	*C9orf153*	*PAFAH1B1*	*CLGN*
COL28A1	*C9orf40*	*PAFAH1B2*	*CLIC1*
COL4A1	*C9orf47*	*PAG1*	*CLIC2*
COL4A3	*C9orf64*	*PALM*	*CLIC5*
COL4A4	*C9orf72*	*PANK2*	*CLINT1*
COL4A6	*C9orf78*	*PANK3*	*CLMN*
COL5A2	*C9orf85*	*PAPLN*	*CLN8*
COL6A5	*CA1*	*PAPSS1*	*CLNK*
COL9A2	*CA10*	*PARP15*	*CLNS1A*
COLEC11	*CA12*	*PARVG*	*CLPB*
COMMD2	*CA13*	*PATE2*	*CLPTM1*
COMMD3-BMI1	*CA2*	*PATZ1*	*CLSPN*
COMMD5	*CA3*	*PAX2*	*CLSTN1*
COMMD9	*CA8*	*PAX7*	*CLSTN2*
COMTD1	*CAAP1*	*PBX1*	*CLTC*
COPS2	*CAB39*	*PCBP1*	*CLU*
COPS7A	*CAB39L*	*PCBP2*	*CLVS2*
COPS8	*CABCOCO1*	*PCDH1*	*CLYBL*
COPZ1	*CABP7*	*PCDH7*	*CMBL*
COQ7	*CABYR*	*PCID2*	*CMC1*
CORIN	*CACNA1I*	*PCSK7*	*CMIP*
CORO1A	*CACNA2D1*	*PCSK9*	*CMSS1*

CORO1C	*CACNA2D2*	*PCYT1A*	*CMTM3*
CORO7	*CACNB1*	*PDCD11*	*CMTM4*
COX15	*CACNB4*	*PDE2A*	*CMTM6*
COX19	*CACNG2*	*PDE9A*	*CMTM8*
COX7A2L	*CACNG4*	*PDF*	*CNBP*
COX8C	*CACNG7*	*PDIA6*	*CNDP2*
CPD	*CACNG8*	*PDK1*	*CNIH3*
CPE	*CACUL1*	*PDLIM2*	*CNIH4*
CPEB3	*CAD*	*PDLIM4*	*CNKSR3*
CPEB4	*CADM1*	*PDZD8*	*CNNM2*
CPED1	*CADM2*	*PER1*	*CNNM3*
CPLX4	*CALB1*	*PEX11A*	*CNNM4*
CPM	*CALCOCO2*	*PEX19*	*CNOT1*
CPNE8	*CALCR*	*PEX5L*	*CNOT2*
CPPED1	*CALCRL*	*PEX7*	*CNOT4*
CPS1	*CALHM4*	*PFKFB3*	*CNOT6*
CPSF6	*CALHM5*	*PFN2*	*CNOT6L*
CPT1A	*CALM1*	*PGAM5*	*CNOT7*
CPT2	*CALM3*	*PGAP3*	*CNP*
CRABP2	*CALML3*	*PGP*	*CNPY3*
CRB1	*CALML4*	*PGR*	*CNPY4*
CRB2	*CALN1*	*PHAX*	*CNR1*
CRB3	*CALR*	*PHC3*	*CNR2*
CREB1	*CALU*	*PHF24*	*CNRIP1*
CREB5	*CALY*	*PHLDA3*	*CNST*
CREBL2	*CAMK1D*	*PHOX2B*	*CNTD1*
CREG2	*CAMK2A*	*PHTF2*	*CNTF*
CREM	*CAMK2D*	*PI15*	*CNTLN*
CRHBP	*CAMK4*	*PI4K2A*	*CNTN1*
CRHR1	*CAMKK2*	*PIAS2*	*CNTN2*
CRIPT	*CAMKMT*	*PIAS4*	*CNTNAP5*
CRISPLD2	*CAMLG*	*PIDD1*	*COA5*
CRKL	*CAMSAP1*	*PIFO*	*COA7*
CRPPA	*CAMSAP2*	*PIGP*	*COBL*
CRTAP	*CAMTA1*	*PIK3IP1*	*COBLL1*
CRTC1	*CAND1*	*PIK3R3*	*COG2*
CRTC3	*CAND2*	*PIKFYVE*	*COG5*
CRX	*CAP1*	*PIMREG*	*COG6*
CRY1	*CAP2*	*PIP5KL1*	*COL12A1*
CRYBG3	*CAPN13*	*PITPNA*	*COL20A1*
CRYZ	*CAPN5*	*PITPNM2*	*COL21A1*
CSAG1	*CAPN6*	*PKNOX1*	*COL26A1*
CSDC2	*CAPN7*	*PLA2G5*	*COL27A1*
CSDE1	*CAPN8*	*PLAAT3*	*COL28A1*

CSE1L	*CAPNS2*	*PLAGL2*	*COL4A1*
CSF1	*CAPRIN1*	*PLBD2*	*COL4A3*
CSF2RA	*CAPS2*	*PLEKHA7*	*COL4A4*
CSF3	*CAPSL*	*PLEKHB2*	*COL4A5*
CSH2	*CAPZA1*	*PLEKHG4B*	*COL4A6*
CSKMT	*CAPZA2*	*PLEKHH1*	*COL6A5*
CSNK1A1	*CAPZB*	*PLEKHM3*	*COL9A2*
CSNK1A1L	*CARD11*	*PLK1*	*COLEC11*
CSNK1D	*CARD16*	*PLPP7*	*COMMD2*
CSNK1G1	*CARD18*	*PLPPR4*	*COMMD3-BMI1*
CSNK2A1	*CARD8*	*PLXNA1*	*COMMD5*
CSPP1	*CARF*	*PMFBP1*	*COMMD9*
CSRNP1	*CARHSP1*	*PNLIPRP3*	*COMTD1*
CSRNP3	*CARMIL1*	*PNMA8A*	*COPS2*
CST3	*CARNMT1*	*PNO1*	*COPS8*
CSTF2	*CASC1*	*PNPLA3*	*COPZ1*
CSTF2T	*CASC4*	*PNRC1*	*COQ7*
CSTF3	*CASK*	*POLE*	*CORIN*
CT45A5	*CASP1*	*POLM*	*CORO1A*
CTBP2	*CASP10*	*POLR1A*	*CORO1C*
CTBS	*CASP2*	*POM121*	*CORO7*
CTCF	*CASP3*	*POTED*	*COX15*
CTCFL	*CASP4*	*POTEI*	*COX18*
CTDSPL	*CASP5*	*PPARGC1B*	*COX19*
CTDSPL2	*CASP7*	*PPFIA3*	*COX6B1*
CTIF	*CASP9*	*PPIF*	*COX7A2L*
CTNNA2	*CASQ2*	*PPIL6*	*COX8C*
CTNNA3	*CAST*	*PPM1H*	*CPA4*
CTNNB1	*CAT*	*PPP1CB*	*CPD*
CTNNBIP1	*CATSPER2*	*PPP1R3F*	*CPE*
CTNNBL1	*CAV1*	*PPP1R7*	*CPEB3*
CTPS2	*CAV2*	*PPP2R3B*	*CPEB4*
CTSB	*CAVIN2*	*PQLC1*	*CPED1*
CTSC	*CAVIN3*	*PRDM1*	*CPLANE2*
CTSE	*CAVIN4*	*PRDM2*	*CPM*
CTTN	*CBFA2T3*	*PRDM6*	*CPNE8*
CTTNBP2NL	*CBFB*	*PRDX1*	*CPPED1*
CTU1	*CBL*	*PRELID2*	*CPS1*
CUEDC1	*CBLB*	*PRELID3B*	*CPSF6*
CUL3	*CBLL1*	*PREX1*	*CPT1A*
CUL5	*CBLN1*	*PRKAB2*	*CPT2*
CUX1	*CBLN2*	*PRKDC*	*CRABP2*
CUX2	*CBLN4*	*PRMT8*	*CRB1*
CWF19L1	*CBR4*	*PROP1*	*CRB2*

CWF19L2	*CBS*	*PRORP*	*CREB1*
CXADR	*CBWD1*	*PROSER3*	*CREB5*
CXCL11	*CBWD2*	*PRPF3*	*CREBL2*
CXCL12	*CBWD3*	*PRPH2*	*CREBZF*
CXCL14	*CBWD5*	*PRR12*	*CREG2*
CXCL17	*CBWD6*	*PRRC2C*	*CRELD2*
CXCR5	*CBX1*	*PRSS23*	*CREM*
CXorf21	*CBX3*	*PRTG*	*CRHBP*
CXorf38	*CBX4*	*PRX*	*CRHR1*
CXorf56	*CBX5*	*PSMA2*	*CRIPT*
CXXC4	*CBX8*	*PSMA5*	*CRISP3*
CYB561	*CC2D1B*	*PSMD11*	*CRISPLD1*
CYB561D1	*CC2D2B*	*PSMG2*	*CRISPLD2*
CYB5D1	*CCAR1*	*PSMG4*	*CRKL*
CYB5R1	*CCBE1*	*PTCHD3*	*CRNKL1*
CYB5R4	*CCDC110*	*PTDSS1*	*CROT*
CYBA	*CCDC112*	*PTGES*	*CRP*
CYBRD1	*CCDC113*	*PTGR2*	*CRPPA*
CYCS	*CCDC117*	*PTK6*	*CRTAP*
CYHR1	*CCDC121*	*PTP4A1*	*CRTC1*
CYP20A1	*CCDC126*	*PTPN11*	*CRTC3*
CYP24A1	*CCDC127*	*PTPN14*	*CRX*
CYP27C1	*CCDC14*	*PTPN9*	*CRY1*
CYP2A7	*CCDC141*	*PTPRD*	*CRYBG3*
CYP2B6	*CCDC148*	*PTPRN2*	*CRYZ*
CYP2C8	*CCDC149*	*PURG*	*CSDE1*
CYP2F1	*CCDC152*	*PWP2*	*CSE1L*
CYP3A5	*CCDC169-SOHLH2*	*PYGO1*	*CSF1*
CYP4F11	*CCDC170*	*PYROXD1*	*CSF2RA*
CYP4V2	*CCDC171*	*QKI*	*CSF3*
CYP4X1	*CCDC173*	*QSER1*	*CSH2*
CYP7A1	*CCDC174*	*QSOX1*	*CSNK1A1*
CYP7B1	*CCDC175*	*RAB11FIP3*	*CSNK1A1L*
CYSLTR2	*CCDC177*	*RAB15*	*CSNK1G1*
CYSTM1	*CCDC181*	*RAB33B*	*CSNK2A1*
CYTH3	*CCDC186*	*RAB3B*	*CSPP1*
CYTIP	*CCDC191*	*RAB3IP*	*CSRNP1*
DAAM1	*CCDC198*	*RAB43*	*CSRNP3*
DAAM2	*CCDC24*	*RAB5B*	*CST3*
DAB2IP	*CCDC25*	*RAB5C*	*CSTF2T*
DACT1	*CCDC3*	*RAB6B*	*CSTF3*
DAG1	*CCDC30*	*RABL6*	*CT45A5*
DAP	*CCDC32*	*RAC1*	*CTBP2*
DARS2	*CCDC34*	*RAD23A*	*CTBS*

DAXX	*CCDC40*	*RAD51C*	*CTCF*
DAZAP1	*CCDC43*	*RALGAPA2*	*CTCFL*
DBN1	*CCDC47*	*RANBP10*	*CTDSP1*
DBNL	*CCDC50*	*RAP1B*	*CTDSP2*
DBT	*CCDC57*	*RAP2B*	*CTDSPL*
DCAF11	*CCDC58*	*RAP2C*	*CTDSPL2*
DCAF12	*CCDC59*	*RAPH1*	*CTIF*
DCAF5	*CCDC6*	*RARS*	*CTNNA2*
DCAF7	*CCDC68*	*RASA2*	*CTNNA3*
DCAKD	*CCDC7*	*RASA3*	*CTNNB1*
DCANP1	*CCDC71L*	*RASGEF1A*	*CTNNBIP1*
DCBLD2	*CCDC74A*	*RASGRP3*	*CTNNBL1*
DCDC1	*CCDC74B*	*RASSF4*	*CTPS2*
DCLK1	*CCDC77*	*RASSF6*	*CTR9*
DCLRE1A	*CCDC80*	*RAVER2*	*CTSB*
DCLRE1C	*CCDC85A*	*RB1*	*CTSC*
DCP2	*CCDC85C*	*RBFA*	*CTSE*
DCTN5	*CCDC88A*	*RBFOX1*	*CTTN*
DCTN6	*CCDC88C*	*RBFOX2*	*CTU1*
DCUN1D1	*CCDC90B*	*RBM19*	*CUEDC1*
DCUN1D3	*CCDC91*	*RBM22*	*CUL3*
DCUN1D4	*CCDC93*	*RBM28*	*CUL5*
DCUN1D5	*CCDC96*	*RBM48*	*CUX1*
DDA1	*CCL1*	*RBM7*	*CUX2*
DDB2	*CCL22*	*RBMX2*	*CWF19L1*
DDHD1	*CCL28*	*RBSN*	*CWF19L2*
DDHD2	*CCL7*	*RCBTB1*	*CXADR*
DDI2	*CCL8*	*RCL1*	*CXCL11*
DDIT4	*CCN1*	*RCN2*	*CXCL12*
DDR1	*CCN2*	*RELB*	*CXCL14*
DDRGK1	*CCN3*	*REPS2*	*CXCL17*
DDX17	*CCN4*	*RFC2*	*CXCR5*
DDX18	*CCNB1*	*RFLNB*	*CXorf38*
DDX19A	*CCNC*	*RHBDL3*	*CXorf56*
DDX19B	*CCND1*	*RHEBL1*	*CXXC4*
DDX20	*CCND2*	*RHOH*	*CYB561*
DDX21	*CCNDBP1*	*RHPN2*	*CYB561D1*
DDX3Y	*CCNF*	*RIC1*	*CYB5D1*
DDX4	*CCNG1*	*RIMBP2*	*CYB5R1*
DDX42	*CCNG2*	*RIMS4*	*CYB5R4*
DDX43	*CCNH*	*RIOK2*	*CYBA*
DDX46	*CCNI*	*RLIM*	*CYBRD1*
DDX55	*CCNI2*	*RMND5A*	*CYCS*
DDX6	*CCNJ*	*RNASE7*	*CYHR1*

DECR1	*CCNL2*	*RNF121*	*CYP1B1*
DEDD	*CCNO*	*RNF144B*	*CYP20A1*
DEFB107A	*CCNT1*	*RNF149*	*CYP24A1*
DEFB107B	*CCNT2*	*RNF165*	*CYP27C1*
DEFB134	*CCNY*	*RNF168*	*CYP2A7*
DELE1	*CCPG1*	*RNF19B*	*CYP2B6*
DENND2A	*CCR1*	*RNF212*	*CYP2C8*
DENND3	*CCR10*	*RNF213*	*CYP2F1*
DENND5B	*CCR5*	*RNF24*	*CYP3A5*
DENND6B	*CCR6*	*RNF40*	*CYP4F11*
DENR	*CCR7*	*RNMT*	*CYP4F12*
DEPDC1	*CCR9*	*RNPEPL1*	*CYP4V2*
DEPTOR	*CCRL2*	*RO60*	*CYP4X1*
DERA	*CCSAP*	*ROPN1*	*CYP51A1*
DERL1	*CCSER1*	*RORB*	*CYP7A1*
DERL2	*CCSER2*	*RORC*	*CYP7B1*
DERL3	*CCT4*	*RPL10*	*CYSLTR2*
DFFA	*CCT6B*	*RPL10A*	*CYSRT1*
DGAT2L6	*CCZ1*	*RPL14*	*CYTH3*
DGCR2	*CCZ1B*	*RPL15*	*CYTH4*
DGCR6L	*CD109*	*RPL19*	*DAAM1*
DGCR8	*CD163L1*	*RPL21*	*DAAM2*
DGKB	*CD164*	*RPL26*	*DAB2IP*
DGKE	*CD180*	*RPL28*	*DACT1*
DGKG	*CD1B*	*RPL31*	*DAOA*
DGKH	*CD1C*	*RPL36A*	*DBN1*
DGKI	*CD1D*	*RPL39L*	*DBNL*
DHCR24	*CD200R1*	*RPL5*	*DBT*
DHDDS	*CD226*	*RPL7*	*DCAF12*
DHRS4	*CD244*	*RPL7A*	*DCAF17*
DHTKD1	*CD248*	*RPL7L1*	*DCAF5*
DHX15	*CD274*	*RPL9*	*DCAF8L1*
DHX35	*CD276*	*RPLP1*	*DCAKD*
DHX36	*CD28*	*RPS15*	*DCANP1*
DHX8	*CD2AP*	*RPS15A*	*DCBLD2*
DIAPH2	*CD2BP2*	*RPS20*	*DCDC1*
DICER1	*CD300E*	*RPS21*	*DCK*
DIDO1	*CD302*	*RPS27*	*DCLK1*
DIMT1	*CD36*	*RPS6KA2*	*DCLRE1A*
DIO2	*CD38*	*RPS8*	*DCLRE1C*
DIP2B	*CD3D*	*RPTOR*	*DCP2*
DIPK1A	*CD3EAP*	*RPUSD1*	*DCTN5*
DIPK2B	*CD3G*	*RRAGD*	*DCTN6*
DIRAS1	*CD44*	*RRM2*	*DCUN1D1*

DIRAS2	*CD46*	*RRN3*	*DCUN1D3*
DIRAS3	*CD47*	*RSL24D1*	*DCUN1D4*
DIS3	*CD53*	*RTL6*	*DCUN1D5*
DIS3L2	*CD55*	*RTL9*	*DDA1*
DISP2	*CD5L*	*RUNX1T1*	*DDAH1*
DKK2	*CD83*	*RUNX3*	*DDB2*
DLD	*CD84*	*RYR2*	*DDHD1*
DLEU7	*CD86*	*S100A10*	*DDHD2*
DLG2	*CD93*	*SAE1*	*DDI2*
DLG3	*CD96*	*SALL4*	*DDIT4*
DLGAP1	*CD99L2*	*SAMD14*	*DDR1*
DLGAP3	*CDADC1*	*SAP30BP*	*DDRGK1*
DLST	*CDC14A*	*SAP30L*	*DDX17*
DLX1	*CDC14B*	*SASH3*	*DDX18*
DLX3	*CDC23*	*SAT1*	*DDX19A*
DMBX1	*CDC25A*	*SATB1*	*DDX19B*
DMPK	*CDC25B*	*SCAMP2*	*DDX20*
DMRT2	*CDC27*	*SCD5*	*DDX21*
DMRT3	*CDC37*	*SCN2B*	*DDX3Y*
DMRTA1	*CDC37L1*	*SCNN1A*	*DDX42*
DMWD	*CDC42BPA*	*SCNN1G*	*DDX43*
DMXL1	*CDC42BPB*	*SCO1*	*DDX46*
DNAAF1	*CDC42EP3*	*SDHAF2*	*DDX55*
DNAAF2	*CDC42EP4*	*SEBOX*	*DDX6*
DNAH14	*CDC42SE2*	*SEMA4G*	*DECR1*
DNAJA2	*CDC5L*	*SEMA6A*	*DEFB107A*
DNAJB12	*CDC6*	*SEPSECS*	*DEFB107B*
DNAJB14	*CDC7*	*SEPTIN10*	*DEFB134*
DNAJB4	*CDC73*	*SERBP1*	*DELE1*
DNAJB6	*CDCA2*	*SERPINB5*	*DENND2A*
DNAJC10	*CDCA5*	*SERPINE1*	*DENND3*
DNAJC16	*CDCA7*	*SET*	*DENND5B*
DNAJC18	*CDCP1*	*SETX*	*DENND6B*
DNAJC19	*CDH1*	*SF3B3*	*DENR*
DNAJC22	*CDH10*	*SFT2D2*	*DEPDC1*
DNAJC24	*CDH11*	*SGCB*	*DEPTOR*
DNAJC30	*CDH12*	*SGCD*	*DERL1*
DNAJC5	*CDH13*	*SGPL1*	*DERL2*
DNAJC6	*CDH17*	*SGSM2*	*DERL3*
DNAL1	*CDH18*	*SGTB*	*DESI2*
DNM3	*CDH19*	*SH3BP2*	*DGAT2L6*
DNMT3A	*CDH2*	*SH3BP4*	*DGCR2*
DNMT3B	*CDH23*	*SH3D19*	*DGCR8*
DNTT	*CDH24*	*SH3RF2*	*DGKB*

DNTTIP2	*CDH26*	*SH3RF3*	*DGKE*
DOCK10	*CDH6*	*SHC1*	*DGKH*
DOCK5	*CDH7*	*SHH*	*DGKI*
DOCK7	*CDH8*	*SHLD1*	*DGLUCY*
DOK3	*CDHR1*	*SHOX*	*DHCR24*
DOK6	*CDK1*	*SIAE*	*DHDDS*
DONSON	*CDK12*	*SIK2*	*DHODH*
DOT1L	*CDK13*	*SIRT7*	*DHRS4*
DPEP2	*CDK14*	*SKI*	*DHTKD1*
DPF2	*CDK15*	*SKIDA1*	*DHX15*
DPH2	*CDK17*	*SLC12A2*	*DHX35*
DPH6	*CDK2*	*SLC14A1*	*DHX36*
DPM1	*CDK5*	*SLC17A4*	*DIAPH2*
DPP10	*CDK5R1*	*SLC17A8*	*DICER1*
DPP6	*CDK5R2*	*SLC18A1*	*DIDO1*
DPP8	*CDK5RAP2*	*SLC19A1*	*DIMT1*
DPPA3	*CDK6*	*SLC19A3*	*DIO2*
DPY19L1	*CDKL1*	*SLC24A4*	*DIP2B*
DPY19L3	*CDKL2*	*SLC25A38*	*DIPK1A*
DPY19L4	*CDKL3*	*SLC26A1*	*DIPK2B*
DPYSL2	*CDKN1A*	*SLC26A7*	*DIRAS1*
DPYSL3	*CDKN1B*	*SLC27A4*	*DIRAS2*
DPYSL4	*CDKN2AIP*	*SLC29A1*	*DIRAS3*
DPYSL5	*CDKN2AIPNL*	*SLC35F1*	*DIS3*
DR1	*CDKN2B*	*SLC35F2*	*DIS3L2*
DRAXIN	*CDKN3*	*SLC38A7*	*DISC1*
DRD1	*CDON*	*SLC39A1*	*DISP2*
DSCAM	*CDPF1*	*SLC39A14*	*DKK2*
DSCC1	*CDR1*	*SLC3A1*	*DLD*
DSG1	*CDS2*	*SLC43A3*	*DLEU7*
DSG2	*CDT1*	*SLC44A1*	*DLG2*
DSG3	*CDV3*	*SLC44A2*	*DLG3*
DST	*CDX2*	*SLC4A4*	*DLGAP1*
DSTN	*CDX4*	*SLC6A11*	*DLGAP3*
DTD2	*CDY1*	*SLC6A7*	*DLST*
DTNA	*CDY1B*	*SLC7A6OS*	*DLX1*
DTWD2	*CDYL*	*SLC8A2*	*DLX3*
DTX3	*CDYL2*	*SLC9A7*	*DMAC2*
DTX3L	*CEACAM1*	*SLCO3A1*	*DMBX1*
DUSP18	*CEACAM5*	*SLCO5A1*	*DMPK*
DUSP19	*CEACAM6*	*SLFN12L*	*DMRT2*
DUSP22	*CEACAM7*	*SLITRK3*	*DMRT3*
DUSP3	*CEACAM8*	*SLX4IP*	*DMRTA1*
DUSP6	*CEBPG*	*SMAD7*	*DNAAF1*

DVL2	*CEBPZOS*	*SMARCA5*	*DNAAF2*
DYNC1I1	*CELF1*	*SMARCD1*	*DNAH14*
DYNC1I2	*CELF2*	*SMC1A*	*DNAJA2*
DYNC1LI2	*CELF3*	*SMC4*	*DNAJB12*
DYNLL2	*CELF5*	*SMCHD1*	*DNAJB14*
DYRK1A	*CEMIP*	*SMG1*	*DNAJB2*
DYRK2	*CEMIP2*	*SMIM19*	*DNAJB4*
DYRK3	*CENPA*	*SMPD3*	*DNAJB6*
DZANK1	*CENPB*	*SMPDL3B*	*DNAJC10*
E2F2	*CENPC*	*SMURF2*	*DNAJC16*
E2F3	*CENPE*	*SNORC*	*DNAJC18*
E2F7	*CENPF*	*SNRNP27*	*DNAJC19*
EAF1	*CENPH*	*SNRPE*	*DNAJC22*
EBF3	*CENPI*	*SNX22*	*DNAJC24*
EBPL	*CENPL*	*SNX4*	*DNAJC27*
ECD	*CENPN*	*SNX8*	*DNAJC3*
ECPAS	*CENPO*	*SNX9*	*DNAJC30*
ECT2	*CENPP*	*SOBP*	*DNAJC5*
EDA	*CENPS-CORT*	*SOGA1*	*DNAJC6*
EDEM1	*CENPU*	*SORL1*	*DNAL1*
EDEM3	*CENPW*	*SOX1*	*DNASE1L1*
EDNRA	*CEP104*	*SP140L*	*DNM3*
EDNRB	*CEP120*	*SP6*	*DNMT3A*
EEF1AKMT2	*CEP126*	*SP7*	*DNMT3B*
EEF1AKMT4-ECE2	*CEP128*	*SP8*	*DNTT*
EEF1AKNMT	*CEP135*	*SPAG6*	*DNTTIP2*
EEF2	*CEP162*	*SPATA17*	*DOCK10*
EEFSEC	*CEP164*	*SPATA2L*	*DOCK7*
EFCAB1	*CEP170*	*SPECC1*	*DOK3*
EFCAB11	*CEP350*	*SPEN*	*DOK6*
EFCAB14	*CEP41*	*SPG7*	*DOK7*
EFCAB2	*CEP44*	*SPIB*	*DONSON*
EFHC2	*CEP55*	*SPOCK2*	*DOT1L*
EFNA1	*CEP57*	*SPOUT1*	*DPEP2*
EFNA2	*CEP57L1*	*SPRY3*	*DPF2*
EFNB2	*CEP63*	*SPSB1*	*DPH2*
EFR3A	*CEP68*	*SPTBN2*	*DPH3*
EFR3B	*CEP72*	*SRARP*	*DPH6*
EGF	*CEP76*	*SRCAP*	*DPM1*
EGFLAM	*CEP78*	*SRD5A1*	*DPP10*
EGFR	*CEP83*	*SRD5A3*	*DPP6*
EHD1	*CEP85L*	*SREK1IP1*	*DPP8*
EHD3	*CEP89*	*SRF*	*DPPA3*
EHD4	*CEP97*	*SRFBP1*	*DPY19L1*

EHF	*CEPT1*	*SRI*	*DPY19L3*
EHMT1	*CERK*	*SRMS*	*DPY19L4*
EID1	*CERKL*	*SRPX2*	*DPYD*
EIF1	*CERS2*	*SRSF3*	*DPYSL2*
EIF1AD	*CERS3*	*SSR3*	*DPYSL3*
EIF1AX	*CERS5*	*SSR4P1*	*DPYSL4*
EIF1B	*CERS6*	*SSTR1*	*DPYSL5*
EIF2AK1	*CETN3*	*SSX3*	*DR1*
EIF2AK2	*CFAP161*	*ST14*	*DRAM1*
EIF2AK3	*CFAP20*	*ST5*	*DRAXIN*
EIF2B2	*CFAP298*	*ST6GAL2*	*DRD1*
EIF2S1	*CFAP300*	*ST6GALNAC4*	*DSCAM*
EIF2S3	*CFAP43*	*STARD3NL*	*DSCC1*
EIF2S3B	*CFAP44*	*STAT5B*	*DSG2*
EIF3B	*CFAP45*	*STK17B*	*DSG3*
EIF3I	*CFAP47*	*STK24*	*DST*
EIF3J	*CFAP61*	*STK25*	*DSTN*
EIF3M	*CFAP65*	*STK35*	*DTD2*
EIF4E	*CFAP97*	*STMN3*	*DTL*
EIF4E2	*CFD*	*STMP1*	*DTNA*
EIF4E3	*CFH*	*STRADA*	*DTWD2*
EIF4EBP2	*CFHR1*	*STXBP6*	*DTX3*
EIF5	*CFHR3*	*SUGT1*	*DTX3L*
EIF5A2	*CFHR4*	*SULF1*	*DUSP1*
EIF5B	*CFL2*	*SULT4A1*	*DUSP18*
EIPR1	*CFLAR*	*SUPT16H*	*DUSP19*
ELAVL1	*CGGBP1*	*SUSD1*	*DUSP22*
ELAVL2	*CGN*	*SUV39H2*	*DUSP3*
ELAVL3	*CGNL1*	*SV2B*	*DUSP6*
ELAVL4	*CGRRF1*	*SV2C*	*DVL2*
ELF3	*CHAC2*	*SYMPK*	*DYNC1H1*
ELF4	*CHAF1B*	*SYNCRIP*	*DYNC1I1*
ELK1	*CHAMP1*	*SYNPO2*	*DYNC1I2*
ELK4	*CHCHD3*	*SYP*	*DYNC1LI2*
ELL2	*CHCHD4*	*SYT7*	*DYNLL2*
ELL3	*CHD1*	*SZRD1*	*DYRK1A*
ELMOD2	*CHD1L*	*TADA2B*	*DYRK2*
ELMOD3	*CHD2*	*TAOK1*	*DYRK3*
ELOB	*CHD5*	*TAPBP*	*DZANK1*
ELOC	*CHD6*	*TARDBP*	*E2F2*
ELOVL6	*CHD7*	*TASOR*	*E2F3*
ELP2	*CHD9*	*TBC1D16*	*E2F7*
ELP4	*CHDH*	*TBKBP1*	*EAF1*
EMB	*CHEK1*	*TBRG1*	*EBF3*

EMC2	*CHERP*	*TCAF2*	*EBNA1BP2*
EMC3	*CHIC1*	*TCEA3*	*EBPL*
EMC7	*CHIC2*	*TCF19*	*ECD*
EMC8	*CHKA*	*TCOF1*	*ECHDC3*
EML4	*CHL1*	*TCP1*	*ECPAS*
EML5	*CHM*	*TCTEX1D1*	*ECT2*
EML6	*CHML*	*TECPR2*	*EDA*
EMP1	*CHMP1B*	*TENM1*	*EDEM1*
EMX2	*CHMP2B*	*TENT4A*	*EDEM3*
EN1	*CHMP3*	*TENT5C*	*EDNRA*
ENAH	*CHMP5*	*TERF2IP*	*EDNRB*
ENC1	*CHN1*	*TEX264*	*EED*
ENDOD1	*CHN2*	*TFAM*	*EEF1A1*
ENGASE	*CHODL*	*TFCP2L1*	*EEF1AKMT2*
ENO1	*CHORDC1*	*TGFBR2*	*EEF1AKMT4-ECE2*
ENOSF1	*CHPT1*	*THAP2*	*EEF1AKNMT*
ENOX1	*CHRAC1*	*THAP6*	*EEFSEC*
ENPEP	*CHRDL1*	*THRA*	*EFCAB1*
ENPP5	*CHRM2*	*THRAP3*	*EFCAB11*
ENPP6	*CHRM3*	*THY1*	*EFCAB14*
ENSA	*CHRNA3*	*TIFA*	*EFCAB2*
ENTPD1	*CHRNA4*	*TIGD6*	*EFHC2*
ENTPD3	*CHRNA5*	*TIGIT*	*EFNA1*
ENTPD4	*CHRNA9*	*TIMM10B*	*EFNA2*
ENTPD7	*CHRNE*	*TIMM8A*	*EFNB2*
EOGT	*CHST10*	*TLCD5*	*EFR3A*
EPB41L1	*CHST11*	*TLE3*	*EFR3B*
EPB41L2	*CHST12*	*TLK2*	*EGF*
EPB41L4B	*CHST14*	*TM9SF3*	*EGFLAM*
EPB41L5	*CHST2*	*TMC2*	*EGFR*
EPC2	*CHST3*	*TMED4*	*EHD3*
EPHA4	*CHST6*	*TMED7*	*EHD4*
EPHA5	*CHST7*	*TMEM106B*	*EHF*
EPHA7	*CHST9*	*TMEM11*	*EHMT1*
EPHA8	*CHSY1*	*TMEM120B*	*EID1*
EPHX2	*CHSY3*	*TMEM134*	*EID2*
EPM2AIP1	*CHUK*	*TMEM135*	*EIF1*
EPN1	*CHURC1*	*TMEM161B*	*EIF1AD*
EPOR	*CHURC1-FNTB*	*TMEM184A*	*EIF1AX*
EPPIN	*CIAO1*	*TMEM185B*	*EIF1B*
EPS15	*CIAO2A*	*TMEM186*	*EIF2AK1*
EPSTI1	*CIDEB*	*TMEM200B*	*EIF2AK2*
ERAL1	*CILP*	*TMEM200C*	*EIF2AK3*
ERBB2	*CIPC*	*TMEM231*	*EIF2B2*

ERBB3	*CIRBP*	*TMEM236*	*EIF2S3*
ERBB4	*CISD1*	*TMEM255B*	*EIF2S3B*
ERC1	*CISD2*	*TMEM30A*	*EIF3A*
ERCC2	*CIT*	*TMEM87B*	*EIF3H*
ERCC4	*CITED2*	*TMPRSS13*	*EIF3J*
ERCC6	*CKAP2*	*TMPRSS9*	*EIF4E*
ERCC6L	*CKAP4*	*TNFAIP2*	*EIF4E3*
ERFE	*CKS1B*	*TNFAIP8*	*EIF4EBP2*
ERG	*CLASP2*	*TNFAIP8L1*	*EIF4G3*
ERI1	*CLCA4*	*TNFAIP8L3*	*EIF5*
ERICH3	*CLCC1*	*TNFRSF13C*	*EIF5A2*
ERLIN1	*CLCN3*	*TOGARAM2*	*EIF5B*
ERMAP	*CLCN5*	*TOM1L2*	*EIPR1*
ERMN	*CLCN6*	*TOMM40*	*ELAVL1*
ERMP1	*CLDN1*	*TOR1A*	*ELAVL2*
ERV3-1	*CLDN11*	*TOR2A*	*ELAVL3*
ERVMER34-1	*CLDN12*	*TP73*	*ELAVL4*
ESCO1	*CLDN16*	*TPO*	*ELF3*
ESCO2	*CLDN18*	*TPPP*	*ELF4*
ESF1	*CLDN19*	*TRAF1*	*ELK1*
ESR1	*CLDN22*	*TRAF3*	*ELK4*
ESRP1	*CLDN23*	*TRAK1*	*ELL2*
ESS2	*CLDN8*	*TRAM2*	*ELL3*
ESYT1	*CLEC12A*	*TRAPPC9*	*ELMOD2*
ESYT3	*CLEC12B*	*TRIB1*	*ELMOD3*
ETFBKMT	*CLEC16A*	*TRIB2*	*ELOC*
ETNK1	*CLEC18A*	*TRIM2*	*ELOVL6*
ETS1	*CLEC1A*	*TRIM38*	*ELP4*
ETV1	*CLEC1B*	*TRIM41*	*EMB*
ETV3	*CLEC2D*	*TRIM44*	*EMC2*
ETV5	*CLEC4C*	*TRIM54*	*EMC3*
EVI5	*CLEC4D*	*TRIM7*	*EMC4*
EVPLL	*CLEC4E*	*TRIM71*	*EMC8*
EVX1	*CLEC5A*	*TRMO*	*EML4*
EVX2	*CLEC7A*	*TRMT10C*	*EML5*
EXO5	*CLEC9A*	*TSC22D1*	*EML6*
EXOC2	*CLGN*	*TSHZ3*	*EMP1*
EXOC6B	*CLIC1*	*TSPAN11*	*EMX2*
EXOC8	*CLIC2*	*TSPAN14*	*EN1*
EXOSC1	*CLIC4*	*TSPAN17*	*ENAH*
EXOSC10	*CLIC5*	*TSPYL2*	*ENC1*
EXOSC2	*CLIC6*	*TSPYL4*	*ENDOD1*
EXOSC3	*CLINT1*	*TSPYL5*	*ENGASE*
EXOSC6	*CLIP3*	*TTC1*	*ENOSF1*

EXTL3	*CLIP4*	*TTC22*	*ENOX1*
EYA3	*CLK4*	*TTC30A*	*ENPEP*
EZH1	*CLLU1OS*	*TTC37*	*ENPP5*
EZR	*CLMN*	*TTC38*	*ENPP6*
F10	*CLMP*	*TTC39A*	*ENSA*
F7	*CLN8*	*TTC39B*	*ENTPD1*
FAAH	*CLNK*	*TTLL7*	*ENTPD3*
FAAP20	*CLNS1A*	*TTYH3*	*ENTPD4*
FABP2	*CLOCK*	*TUBGCP3*	*ENTPD5*
FADS1	*CLPB*	*TULP4*	*ENTPD6*
FAF1	*CLPTM1L*	*TUSC3*	*ENTPD7*
FAF2	*CLPX*	*TXLNA*	*ENY2*
FAHD1	*CLRN1*	*TYW5*	*EOGT*
FAIM2	*CLSPN*	*UBA2*	*EPB41L1*
FAM102A	*CLSTN2*	*UBB*	*EPB41L4B*
FAM102B	*CLSTN3*	*UBE2E3*	*EPB41L5*
FAM104B	*CLTC*	*UBE2I*	*EPC2*
FAM110B	*CLUAP1*	*UBE3B*	*EPHA4*
FAM114A1	*CLUL1*	*UBE4A*	*EPHA5*
FAM114A2	*CLVS2*	*UBN2*	*EPHA7*
FAM117B	*CLYBL*	*UBQLN1*	*EPHA8*
FAM118A	*CMBL*	*UBXN10*	*EPHX2*
FAM120A	*CMC1*	*UBXN2B*	*EPM2A*
FAM122A	*CMKLR1*	*UCKL1*	*EPM2AIP1*
FAM122C	*CMPK1*	*UGCG*	*EPN1*
FAM126B	*CMPK2*	*UGGT1*	*EPOR*
FAM131B	*CMSS1*	*UHRF1BP1*	*EPS15*
FAM135A	*CMTM4*	*UHRF1BP1L*	*EPSTI1*
FAM135B	*CMTM6*	*UMODL1*	*ERAL1*
FAM13A	*CMTR1*	*UNC45B*	*ERBB2*
FAM151B	*CMTR2*	*UNC50*	*ERBB3*
FAM155B	*CNBP*	*UPK3BL1*	*ERBB4*
FAM160B1	*CNDP2*	*UQCC2*	*ERC1*
FAM161A	*CNEP1R1*	*UQCR10*	*ERCC4*
FAM162A	*CNGB1*	*UQCRFS1*	*ERCC6*
FAM163A	*CNGB3*	*URB1-AS1*	*ERFE*
FAM167A	*CNIH4*	*URM1*	*ERG*
FAM168A	*CNKSR2*	*USP1*	*ERI1*
FAM168B	*CNKSR3*	*USP15*	*ERICH3*
FAM172A	*CNN1*	*USP3*	*ERMAP*
FAM181B	*CNNM1*	*USP35*	*ERMN*
FAM185A	*CNNM2*	*USP4*	*ERMP1*
FAM193A	*CNNM3*	*USP51*	*ERV3-1*
FAM199X	*CNNM4*	*UST*	*ERVMER34-1*

FAM204A	*CNOT1*	*UTP4*	*ESCO1*
FAM214A	*CNOT10*	*VAPA*	*ESCO2*
FAM216A	*CNOT2*	*VAPB*	*ESF1*
FAM217B	*CNOT4*	*VASN*	*ESR1*
FAM219A	*CNOT6*	*VAX1*	*ESRP1*
FAM219B	*CNOT6L*	*VCP*	*ESS2*
FAM222B	*CNOT7*	*VENTX*	*ESYT1*
FAM227A	*CNOT8*	*VHL*	*ESYT3*
FAM229B	*CNOT9*	*VLDLR*	*ETFBKMT*
FAM241A	*CNP*	*VNN1*	*ETNK1*
FAM3B	*CNPPD1*	*VPS45*	*ETS1*
FAM53B	*CNPY1*	*VSIG4*	*ETV1*
FAM53C	*CNR1*	*VSTM5*	*ETV3*
FAM71C	*CNR2*	*VWA1*	*ETV5*
FAM81A	*CNST*	*WARS*	*ETV6*
FAM83A	*CNTD1*	*WBP11*	*EVI5*
FAM83F	*CNTF*	*WDR20*	*EVL*
FAM83G	*CNTFR*	*WDR4*	*EVPLL*
FAM89A	*CNTLN*	*WDR55*	*EVX1*
FAM8A1	*CNTN1*	*WFIKKN2*	*EXO5*
FAM91A1	*CNTN2*	*WHAMM*	*EXOC2*
FAM98A	*CNTN3*	*WNT2B*	*EXOC6B*
FAN1	*CNTN5*	*WNT4*	*EXOC8*
FANCA	*CNTN6*	*WNT5B*	*EXOSC2*
FANCB	*CNTNAP3*	*WNT7B*	*EXOSC3*
FANCD2	*CNTNAP5*	*WSB1*	*EXOSC6*
FANCE	*COA1*	*WTIP*	*EXTL3*
FANCF	*COA4*	*WWC3*	*EYS*
FANCM	*COA5*	*WWP2*	*EZH1*
FAR2	*COA7*	*XIRP1*	*F13A1*
FARP1	*COBL*	*XKR4*	*F2R*
FASLG	*COBLL1*	*XKR7*	*F2RL2*
FASN	*COCH*	*XRCC5*	*F7*
FASTKD2	*COG1*	*XRCC6*	*FAAH*
FAT1	*COG5*	*XXYLT1*	*FAAP20*
FAT2	*COG6*	*YBX3*	*FABP2*
FAXDC2	*COIL*	*YIPF5*	*FADS1*
FBLN1	*COL11A1*	*YIPF6*	*FAF1*
FBLN7	*COL12A1*	*YPEL1*	*FAF2*
FBN1	*COL14A1*	*YTHDC1*	*FAHD1*
FBN2	*COL1A1*	*YWHAE*	*FAHD2A*
FBXL16	*COL1A2*	*ZADH2*	*FAIM2*
FBXL18	*COL20A1*	*ZBED1*	*FAM102A*
FBXL20	*COL21A1*	*ZBTB26*	*FAM104A*

FBXL3	*COL22A1*	*ZBTB4*	*FAM104B*
FBXL4	*COL23A1*	*ZBTB43*	*FAM110B*
FBXO21	*COL25A1*	*ZBTB7A*	*FAM114A2*
FBXO22	*COL26A1*	*ZBTB7B*	*FAM117B*
FBXO27	*COL28A1*	*ZBTB8A*	*FAM118A*
FBXO28	*COL3A1*	*ZC3H10*	*FAM120A*
FBXO3	*COL4A1*	*ZC3HAV1*	*FAM120AOS*
FBXO30	*COL4A3*	*ZCCHC12*	*FAM120C*
FBXO31	*COL4A4*	*ZCCHC4*	*FAM122A*
FBXO32	*COL5A2*	*ZDHHC18*	*FAM122C*
FBXO33	*COL5A3*	*ZFAND2A*	*FAM126B*
FBXO41	*COL6A3*	*ZFAT*	*FAM135A*
FBXO42	*COL6A5*	*ZFP37*	*FAM135B*
FBXO47	*COL7A1*	*ZFX*	*FAM13A*
FBXO8	*COL8A1*	*ZFYVE19*	*FAM151B*
FBXO9	*COL9A1*	*ZNF101*	*FAM155B*
FBXW2	*COL9A2*	*ZNF106*	*FAM160B1*
FBXW8	*COL9A3*	*ZNF124*	*FAM161A*
FCAR	*COLEC10*	*ZNF132*	*FAM162A*
FCF1	*COLEC11*	*ZNF146*	*FAM163A*
FCGR2A	*COMMD10*	*ZNF155*	*FAM163B*
FCGR2B	*COMMD2*	*ZNF169*	*FAM167A*
FCGR3A	*COMMD3-BMI1*	*ZNF19*	*FAM168A*
FCHO2	*COMMD8*	*ZNF197*	*FAM168B*
FCHSD1	*COPA*	*ZNF215*	*FAM172A*
FCN2	*COPS2*	*ZNF280D*	*FAM177A1*
FCSK	*COPS3*	*ZNF284*	*FAM181B*
FECH	*COPS4*	*ZNF317*	*FAM185A*
FEM1A	*COPS7B*	*ZNF346*	*FAM193A*
FEM1B	*COPS8*	*ZNF396*	*FAM199X*
FEM1C	*COQ10A*	*ZNF417*	*FAM204A*
FER	*COQ10B*	*ZNF430*	*FAM214A*
FERMT1	*COQ7*	*ZNF483*	*FAM216A*
FERMT3	*CORIN*	*ZNF496*	*FAM217B*
FEZ2	*CORO1C*	*ZNF510*	*FAM219A*
FFAR2	*CORO2A*	*ZNF511*	*FAM219B*
FFAR3	*CORT*	*ZNF548*	*FAM222B*
FGA	*COTL1*	*ZNF554*	*FAM227A*
FGD4	*COX10*	*ZNF576*	*FAM229B*
FGD6	*COX11*	*ZNF587B*	*FAM234B*
FGF10	*COX15*	*ZNF607*	*FAM241A*
FGF12	*COX16*	*ZNF609*	*FAM3B*
FGF14	*COX18*	*ZNF618*	*FAM53B*
FGF19	*COX19*	*ZNF652*	*FAM76A*

FGF2	*COX20*	*ZNF701*	*FAM81A*
FGF5	*COX6B1*	*ZNF704*	*FAM83A*
FGF9	*COX6C*	*ZNF738*	*FAM83F*
FGFR1OP	*COX7A2L*	*ZNF75A*	*FAM89A*
FGFR1OP2	*COX7C*	*ZNF778*	*FAM8A1*
FGR	*COX8C*	*ZNF784*	*FAM91A1*
FHL5	*CPA3*	*ZNF792*	*FAM98A*
FICD	*CPA4*	*ZNF793*	*FAN1*
FIGN	*CPD*	*ZNF831*	*FANCA*
FIGNL1	*CPE*	*ZNF845*	*FANCB*
FILIP1	*CPEB4*	*ZNF99*	*FANCD2*
FIZ1	*CPED1*	*ZNRF2*	*FANCF*
FKBP14	*CPLX1*	*ZYG11A*	*FANCM*
FKBP4	*CPLX2*	*ZZEF1*	*FAR2*
FKBP5	*CPLX3*		*FARP1*
FKBP7	*CPLX4*		*FARSA*
FKBP9	*CPM*		*FASLG*
FKTN	*CPN1*		*FASTKD2*
FLNA	*CPN2*		*FAT2*
FLRT2	*CPNE8*		*FAXC*
FLRT3	*CPPED1*		*FAXDC2*
FLT1	*CPQ*		*FBLN1*
FLYWCH2	*CPS1*		*FBLN7*
FMN1	*CPSF2*		*FBN1*
FMNL1	*CPSF3*		*FBN2*
FMR1	*CPSF6*		*FBN3*
FN1	*CPT1A*		*FBXL15*
FN3KRP	*CR1*		*FBXL16*
FNBP1	*CR2*		*FBXL18*
FNBP1L	*CRB1*		*FBXL20*
FNDC10	*CRBN*		*FBXL3*
FNDC3A	*CREB1*		*FBXL4*
FNDC3B	*CREB3*		*FBXO22*
FNDC9	*CREB3L2*		*FBXO27*
FNIP1	*CREB3L4*		*FBXO28*
FNIP2	*CREB5*		*FBXO3*
FNTA	*CREBL2*		*FBXO30*
FNTB	*CREBRF*		*FBXO31*
FOS	*CREG1*		*FBXO32*
FOSL2	*CREG2*		*FBXO33*
FOXA1	*CRELD1*		*FBXO41*
FOXF1	*CREM*		*FBXO42*
FOXJ1	*CRH*		*FBXO45*
FOXL1	*CRIM1*		*FBXO47*

FOXL2NB	*CRIP3*	*FBXO8*
FOXN1	*CRIPT*	*FBXO9*
FOXN2	*CRISP1*	*FBXW11*
FOXO1	*CRISP2*	*FBXW8*
FOXO3	*CRISP3*	*FCAR*
FOXO4	*CRISPLD2*	*FCF1*
FOXP1	*CRK*	*FCGR2A*
FOXP2	*CRKL*	*FCGR2B*
FOXRED1	*CRLF3*	*FCGR3A*
FPGS	*CRLS1*	*FCHO2*
FPGT	*CRMP1*	*FCHSD1*
FPR3	*CRNKL1*	*FCN2*
FRA10AC1	*CROT*	*FCRL3*
FRAT1	*CRP*	*FCSK*
FRAT2	*CRPPA*	*FECH*
FREM2	*CRTAM*	*FEM1B*
FRK	*CRTAP*	*FEM1C*
FRMD4B	*CRTC1*	*FER*
FRMD5	*CRTC3*	*FERMT1*
FRMD6	*CRX*	*FERMT3*
FRMPD4	*CRY1*	*FEZ1*
FRRS1	*CRYBG1*	*FEZ2*
FRRS1L	*CRYBG3*	*FFAR2*
FRS2	*CSAD*	*FFAR3*
FRYL	*CSDE1*	*FGA*
FSCB	*CSE1L*	*FGD4*
FSD2	*CSF1R*	*FGD6*
FSHB	*CSGALNACT1*	*FGF10*
FSTL4	*CSGALNACT2*	*FGF12*
FSTL5	*CSH2*	*FGF14*
FTO	*CSN1S1*	*FGF19*
FUBP1	*CSN2*	*FGF2*
FUCA2	*CSNK1A1*	*FGF5*
FUNDC2	*CSNK1A1L*	*FGF9*
FUS	*CSNK1G1*	*FGFR1OP*
FUT1	*CSNK1G3*	*FGFR1OP2*
FUT10	*CSNK2A1*	*FGL1*
FUT11	*CSPP1*	*FGL2*
FUT2	*CSRNP1*	*FGR*
FUT4	*CSRNP2*	*FHL5*
FUT8	*CSRNP3*	*FIBIN*
FUT9	*CST3*	*FICD*
FXN	*CSTB*	*FIGN*
FXR1	*CSTF1*	*FIGNL1*

FXYD5	*CSTF2*	*FILIP1*
FYTTD1	*CSTF2T*	*FIZ1*
FZD1	*CSTF3*	*FKBP14*
FZD3	*CT45A5*	*FKBP5*
FZD4	*CT83*	*FKBP7*
FZD5	*CTAGE1*	*FKBP9*
FZD8	*CTAGE8*	*FKTN*
FZR1	*CTAGE9*	*FLI1*
G2E3	*CTBP2*	*FLNA*
G3BP1	*CTBS*	*FLRT1*
G3BP2	*CTC1*	*FLRT2*
G6PC	*CTCF*	*FLRT3*
GAB1	*CTCFL*	*FLT1*
GAB2	*CTDP1*	*FLYWCH2*
GAB3	*CTDSPL*	*FMN1*
GABBR2	*CTDSPL2*	*FMNL1*
GABPA	*CTH*	*FMR1*
GABRA1	*CTHRC1*	*FN1*
GABRA3	*CTLA4*	*FN3KRP*
GABRB1	*CTNNA2*	*FNBP1*
GABRB3	*CTNNA3*	*FNBP1L*
GABRG1	*CTNNB1*	*FNBP4*
GABRG3	*CTNNBIP1*	*FNDC10*
GABRR1	*CTNNBL1*	*FNDC3A*
GABRR2	*CTNND2*	*FNDC3B*
GAD2	*CTNS*	*FNDC9*
GAGE1	*CTPS2*	*FNIP1*
GALC	*CTSB*	*FNIP2*
GALNS	*CTSC*	*FNTA*
GALNT10	*CTSH*	*FNTB*
GALNT15	*CTSL*	*FOS*
GALNT17	*CTSO*	*FOSL2*
GALNT18	*CTSS*	*FOXA1*
GALNT2	*CTSV*	*FOXA2*
GALNT3	*CTTN*	*FOXF1*
GALNT7	*CTTNBP2*	*FOXJ1*
GALR1	*CTTNBP2NL*	*FOXL1*
GANAB	*CTU1*	*FOXL2NB*
GAPVD1	*CTXN2*	*FOXN1*
GAR1	*CTXN3*	*FOXN2*
GAS1	*CUBN*	*FOXO1*
GAS2	*CUEDC2*	*FOXO3*
GAS2L3	*CUL1*	*FOXO4*
GAS5	*CUL3*	*FOXP1*

GAS7	*CUL5*	*FOXP2*
GAS8	*CUX1*	*FOXRED1*
GASK1B	*CWC22*	*FOXRED2*
GATA1	*CWF19L2*	*FPGT*
GATA4	*CX3CR1*	*FPR1*
GATA5	*CXADR*	*FPR3*
GATA6	*CXCL1*	*FRA10AC1*
GATAD1	*CXCL11*	*FRAT1*
GATAD2A	*CXCL12*	*FRAT2*
GATD3A	*CXCL13*	*FREM2*
GBA	*CXCL14*	*FRG2*
GBE1	*CXCL2*	*FRG2C*
GBGT1	*CXCL3*	*FRK*
GBP2	*CXCL5*	*FRMD3*
GBP4	*CXCL8*	*FRMD4B*
GBX2	*CXCL9*	*FRMD6*
GCC1	*CXCR2*	*FRMPD4*
GCG	*CXCR4*	*FRRS1*
GCLC	*CXorf21*	*FRRS1L*
GCLM	*CXorf38*	*FRS2*
GCN1	*CXorf56*	*FRYL*
GCNT1	*CXXC4*	*FSCB*
GCNT2	*CXXC5*	*FSCN1*
GCSAML	*CYB561A3*	*FSD2*
GDA	*CYB561D1*	*FSHB*
GDAP1	*CYB5D2*	*FSTL4*
GDAP2	*CYB5R2*	*FSTL5*
GDF9	*CYB5R4*	*FTO*
GDI1	*CYBB*	*FUBP1*
GDI2	*CYBRD1*	*FUCA2*
GDNF	*CYCS*	*FUNDC2*
GDPD1	*CYP11B1*	*FUS*
GDPGP1	*CYP19A1*	*FUT1*
GEM	*CYP1A1*	*FUT10*
GEMIN2	*CYP1A2*	*FUT11*
GEMIN6	*CYP20A1*	*FUT2*
GFAP	*CYP24A1*	*FUT4*
GFER	*CYP27B1*	*FUT8*
GFM1	*CYP27C1*	*FUT9*
GFOD1	*CYP2A7*	*FXN*
GFPT1	*CYP2C19*	*FXR1*
GFPT2	*CYP2C8*	*FXYD5*
GFRA1	*CYP39A1*	*FYTTD1*
GFRA2	*CYP3A5*	*FZD1*

GFRA4	*CYP3A7*	*FZD3*
GGA1	*CYP4V2*	*FZD4*
GGA2	*CYP4X1*	*FZD5*
GGCT	*CYP51A1*	*FZD8*
GGCX	*CYP7A1*	*FZR1*
GHITM	*CYP7B1*	*G2E3*
GHR	*CYP8B1*	*G3BP1*
GHRH	*CYSLTR1*	*G3BP2*
GID4	*CYSLTR2*	*G6PC*
GID8	*CYTIP*	*GAB1*
GIGYF1	*DAAM1*	*GAB2*
GIMAP4	*DACH2*	*GAB3*
GINM1	*DACT2*	*GABBR1*
GINS2	*DACT3*	*GABBR2*
GIPC2	*DAP*	*GABPA*
GIT1	*DAPP1*	*GABRA1*
GJA1	*DARS*	*GABRA3*
GJB2	*DAW1*	*GABRA4*
GJB6	*DAZ1*	*GABRB1*
GJC1	*DAZ3*	*GABRG1*
GJC3	*DAZ4*	*GABRG3*
GK	*DAZAP2*	*GABRR1*
GK5	*DAZL*	*GABRR2*
GLA	*DBF4*	*GAD2*
GLDN	*DBF4B*	*GAGE1*
GLG1	*DBH*	*GALC*
GLI2	*DBNL*	*GALNS*
GLI3	*DBR1*	*GALNT10*
GLIPR1L2	*DBT*	*GALNT15*
GLIS3	*DBX2*	*GALNT17*
GLMN	*DCAF10*	*GALNT18*
GLO1	*DCAF11*	*GALNT2*
GLOD4	*DCAF12L1*	*GALNT3*
GLP2R	*DCAF13*	*GALNT6*
GLRA2	*DCAF16*	*GALNT7*
GLRX2	*DCAF17*	*GALR1*
GLRX5	*DCAF4L1*	*GAPVD1*
GLS	*DCAF5*	*GAR1*
GLT8D2	*DCAF8L1*	*GAS1*
GLUD2	*DCAKD*	*GAS2*
GLUL	*DCBLD1*	*GAS8*
GLYAT	*DCBLD2*	*GASK1B*
GLYCTK	*DCC*	*GATA1*
GM2A	*DCDC1*	*GATA4*

GMDS	*DCHS2*	*GATA5*
GMEB1	*DCK*	*GATA6*
GMFB	*DCLK1*	*GATAD1*
GMNC	*DCLK2*	*GATAD2A*
GMNN	*DCLK3*	*GATAD2B*
GMPR	*DCN*	*GATC*
GNA13	*DCP2*	*GATD3A*
GNAI2	*DCPS*	*GBA*
GNAI3	*DCT*	*GBE1*
GNAL	*DCTN3*	*GBGT1*
GNAO1	*DCTN4*	*GBP1*
GNAQ	*DCTN5*	*GBP2*
GNAT1	*DCTN6*	*GBP4*
GNB1	*DCUN1D1*	*GBP6*
GNB3	*DCUN1D3*	*GBX2*
GNB4	*DCUN1D4*	*GCC1*
GNB5	*DCUN1D5*	*GCG*
GNG11	*DDB2*	*GCLC*
GNG12	*DDHD1*	*GCLM*
GNG2	*DDI1*	*GCN1*
GNG7	*DDI2*	*GCNT1*
GNL1	*DDIT4L*	*GCNT2*
GNL3L	*DDN*	*GCSAML*
GNPAT	*DDO*	*GDA*
GOLGA1	*DDR1*	*GDAP1*
GOLGA3	*DDR2*	*GDAP2*
GOLGA4	*DDX17*	*GDF9*
GOLGA7	*DDX18*	*GDI2*
GOLGA7B	*DDX19A*	*GDPD1*
GOLGA8H	*DDX19B*	*GDPGP1*
GOLGA8K	*DDX20*	*GEM*
GOLGA8M	*DDX21*	*GEMIN2*
GOLGA8O	*DDX3Y*	*GEMIN6*
GOLGA8R	*DDX4*	*GFAP*
GOLM1	*DDX46*	*GFER*
GOLPH3L	*DDX5*	*GFM1*
GOLT1B	*DDX50*	*GFOD1*
GON4L	*DDX55*	*GFPT1*
GON7	*DDX60L*	*GFPT2*
GOPC	*DECR1*	*GFRA1*
GORASP1	*DEDD2*	*GFRA2*
GOSR1	*DEF8*	*GFRA4*
GP5	*DEFB107A*	*GGA1*
GPAM	*DEFB107B*	*GGA2*

GPATCH2	*DEFB119*	*GGCT*
GPATCH2L	*DEFB125*	*GGCX*
GPATCH8	*DEGS1*	*GHITM*
GPBP1	*DELE1*	*GHR*
GPC6	*DENND1A*	*GHRH*
GPCPD1	*DENND1B*	*GHSR*
GPD1	*DENND2C*	*GID4*
GPD2	*DENND2D*	*GID8*
GPER1	*DENND4B*	*GIGYF1*
GPI	*DENND5B*	*GIMAP4*
GPKOW	*DENND6A*	*GINM1*
GPM6A	*DENR*	*GINS2*
GPM6B	*DEPDC1*	*GIPC2*
GPN2	*DEPDC1B*	*GIT1*
GPNMB	*DEPTOR*	*GJA1*
GPR107	*DERA*	*GJA3*
GPR12	*DERL1*	*GJB2*
GPR135	*DERL2*	*GJB4*
GPR137B	*DESI2*	*GJB6*
GPR143	*DET1*	*GJC1*
GPR15	*DEXI*	*GJC3*
GPR151	*DFFA*	*GK*
GPR160	*DFFB*	*GK5*
GPR17	*DGAT2*	*GLA*
GPR173	*DGCR2*	*GLDN*
GPR176	*DGKB*	*GLG1*
GPR180	*DGKE*	*GLI2*
GPR22	*DGKH*	*GLI3*
GPR25	*DGKI*	*GLIPR1*
GPR26	*DGKQ*	*GLIPR1L2*
GPR27	*DGKZ*	*GLIS3*
GPR34	*DGLUCY*	*GLMN*
GPR37	*DHCR24*	*GLOD4*
GPR39	*DHDDS*	*GLP2R*
GPR42	*DHFR*	*GLRA2*
GPR63	*DHFR2*	*GLRX2*
GPR68	*DHODH*	*GLRX5*
GPR85	*DHRS12*	*GLS*
GPRACR	*DHRS2*	*GLT8D2*
GPRC5A	*DHRSX*	*GLUD2*
GPRC5B	*DHTKD1*	*GLUL*
GPRIN2	*DHX15*	*GLYAT*
GPRIN3	*DHX29*	*GLYCTK*
GPSM2	*DHX30*	*GLYR1*

GPT2	*DHX33*	*GM2A*
GPX6	*DHX35*	*GMDS*
GRAMD4	*DHX36*	*GMEB1*
GRAP2	*DHX37*	*GMFB*
GRB10	*DHX40*	*GMNC*
GREB1	*DIABLO*	*GMNN*
GREM2	*DIAPH2*	*GMPR*
GRHL1	*DICER1*	*GMPS*
GRHPR	*DIDO1*	*GNA13*
GRIA2	*DIMT1*	*GNA14*
GRIA4	*DIO1*	*GNAI2*
GRID1	*DIO2*	*GNAI3*
GRIK3	*DIO3*	*GNAL*
GRIN2A	*DIP2B*	*GNAO1*
GRIN2B	*DIP2C*	*GNAQ*
GRIN3A	*DIPK2A*	*GNAS*
GRK3	*DIPK2B*	*GNAT1*
GRK5	*DIRAS2*	*GNB1*
GRM1	*DIRAS3*	*GNB1L*
GRM2	*DIS3*	*GNB3*
GRM4	*DIS3L2*	*GNB4*
GRPEL1	*DISC1*	*GNB5*
GRSF1	*DIXDC1*	*GNG11*
GSDMD	*DKC1*	*GNG12*
GSE1	*DKK1*	*GNG2*
GSK3B	*DKK2*	*GNG7*
GSPT1	*DKK3*	*GNGT2*
GSTK1	*DLC1*	*GNL1*
GSTM2	*DLD*	*GNL3L*
GSTO2	*DLEU7*	*GNPAT*
GTDC1	*DLG1*	*GNPNAT1*
GTF2A1	*DLG2*	*GOLGA1*
GTF2A2	*DLG3*	*GOLGA3*
GTF2B	*DLG5*	*GOLGA4*
GTF2E1	*DLGAP1*	*GOLGA7*
GTF2H2C	*DLGAP3*	*GOLGA7B*
GTF2H5	*DLK1*	*GOLGA8B*
GTF3C4	*DLL4*	*GOLGA8H*
GTF3C5	*DLX2*	*GOLGA8K*
GTF3C6	*DLX5*	*GOLGA8M*
GTPBP1	*DMAC1*	*GOLGA8O*
GTPBP10	*DMAC2L*	*GOLGA8R*
GTPBP8	*DMC1*	*GOLM1*
GUCY1A2	*DMD*	*GOLPH3L*

GUF1	*DMGDH*	*GON4L*
GVQW3	*DMKN*	*GON7*
GXYLT1	*DMP1*	*GOPC*
GXYLT2	*DMRT2*	*GORASP1*
GYPE	*DMRTA1*	*GOSR1*
GYS2	*DMRTB1*	*GPAM*
GZF1	*DMXL1*	*GPATCH2*
H1F0	*DMXL2*	*GPATCH2L*
H1FX	*DNAAF1*	*GPATCH4*
H2AFV	*DNAAF2*	*GPATCH8*
H3F3B	*DNAAF5*	*GPBP1*
HAAO	*DNAH12*	*GPC6*
HABP2	*DNAH14*	*GPCPD1*
HACD3	*DNAH3*	*GPD1*
HACD4	*DNAH5*	*GPD2*
HAP1	*DNAH8*	*GPER1*
HARS	*DNAJA2*	*GPI*
HAS2	*DNAJA3*	*GPKOW*
HAS3	*DNAJB12*	*GPM6A*
HAUS3	*DNAJB13*	*GPM6B*
HAUS8	*DNAJB14*	*GPNMB*
HAVCR2	*DNAJB4*	*GPR107*
HBEGF	*DNAJB5*	*GPR12*
HBS1L	*DNAJB6*	*GPR132*
HCAR1	*DNAJB7*	*GPR135*
HCFC1	*DNAJB9*	*GPR137B*
HCFC2	*DNAJC10*	*GPR143*
HCN1	*DNAJC12*	*GPR15*
HCN3	*DNAJC15*	*GPR151*
HCN4	*DNAJC16*	*GPR160*
HDAC11	*DNAJC18*	*GPR161*
HDAC4	*DNAJC19*	*GPR17*
HDAC6	*DNAJC21*	*GPR173*
HDAC8	*DNAJC22*	*GPR174*
HDAC9	*DNAJC24*	*GPR176*
HDGF	*DNAJC25*	*GPR180*
HDGFL1	*DNAJC25-GNG10*	*GPR22*
HDHD2	*DNAJC27*	*GPR25*
HDLBP	*DNAJC28*	*GPR26*
HDX	*DNAJC3*	*GPR27*
HEBP2	*DNAL1*	*GPR34*
HECTD2	*DNALI1*	*GPR37*
HECW1	*DNHD1*	*GPR37L1*
HEG1	*DNM1L*	*GPR42*

HELZ	*DNM3*	*GPR63*
HEMK1	*DNMT3A*	*GPR65*
HEPACAM	*DNMT3B*	*GPR68*
HERC2	*DNTTIP2*	*GPR78*
HERC4	*DOCK1*	*GPR85*
HES1	*DOCK10*	*GPRACR*
HES4	*DOCK3*	*GPRC5A*
HES6	*DOCK4*	*GPRC5B*
HES7	*DOCK5*	*GPRIN2*
HEXIM1	*DOCK7*	*GPRIN3*
HEYL	*DOCK8*	*GPSM2*
HHAT	*DOCK9*	*GPT2*
HHIP	*DOK3*	*GPX3*
HHIPL1	*DOK6*	*GPX6*
HIBCH	*DONSON*	*GRAMD1B*
HIF1A	*DOP1A*	*GRAMD4*
HIF1AN	*DOP1B*	*GRAP2*
HIGD1A	*DOT1L*	*GRAPL*
HIKESHI	*DPF2*	*GRB10*
HILPDA	*DPH3*	*GREB1*
HIP1	*DPH5*	*GREM2*
HIPK1	*DPH6*	*GRHL1*
HIPK2	*DPH7*	*GRHL3*
HIPK3	*DPM1*	*GRHPR*
HIST1H1C	*DPP10*	*GRIA2*
HIST1H2AG	*DPP4*	*GRIA4*
HIST1H2BD	*DPP6*	*GRID1*
HIST1H3H	*DPP8*	*GRID2*
HIST2H4B	*DPPA3*	*GRIK3*
HK2	*DPPA4*	*GRIN2A*
HLA-DPB1	*DPY19L1*	*GRIN2B*
HLTF	*DPY19L2*	*GRIN3A*
HMBOX1	*DPY19L3*	*GRK3*
HMG20A	*DPY19L4*	*GRK5*
HMGA1	*DPYD*	*GRM1*
HMGB2	*DPYSL2*	*GRM2*
HMGB3	*DPYSL5*	*GRM4*
HMGCLL1	*DR1*	*GRPEL1*
HMGCR	*DRAM1*	*GRSF1*
HMGN4	*DRAXIN*	*GSDMD*
HMGXB4	*DRC3*	*GSE1*
HNF1A	*DRD1*	*GSG1*
HNF4A	*DRG1*	*GSK3B*
HNF4G	*DRICH1*	*GSPT1*

HNMT	*DSC1*	*GSR*
HNRNPA0	*DSC2*	*GSTK1*
HNRNPA3	*DSC3*	*GSTM2*
HNRNPD	*DSCC1*	*GSTO2*
HNRNPH1	*DSE*	*GTDC1*
HNRNPH3	*DSEL*	*GTF2A1*
HNRNPR	*DSG2*	*GTF2A2*
HNRNPU	*DSG3*	*GTF2B*
HOGA1	*DSG4*	*GTF2E1*
HOMER1	*DSN1*	*GTF2F1*
HOMEZ	*DST*	*GTF2F2*
HOOK1	*DSTN*	*GTF2H2C*
HOOK3	*DTD2*	*GTF2H5*
HOXA13	*DTHD1*	*GTF3C4*
HOXA7	*DTL*	*GTF3C6*
HOXA9	*DTNA*	*GTPBP1*
HOXB13	*DTNBP1*	*GTPBP10*
HOXB5	*DTWD1*	*GTPBP8*
HOXB7	*DTWD2*	*GUCY1A2*
HOXB9	*DTX3L*	*GUF1*
HOXC10	*DTX4*	*GVQW3*
HOXC13	*DUOX1*	*GXYLT1*
HOXC5	*DUOXA1*	*GXYLT2*
HOXD1	*DUS1L*	*GYPE*
HOXD10	*DUS4L*	*GZF1*
HOXD12	*DUSP1*	*H1F0*
HOXD4	*DUSP12*	*H1FX*
HOXD8	*DUSP16*	*H2AFV*
HOXD9	*DUSP18*	*H2AFY*
HP1BP3	*DUSP19*	*H3F3B*
HPCAL4	*DUSP2*	*HAAO*
HPGD	*DUSP28*	*HABP2*
HPSE	*DUSP4*	*HACD2*
HPX	*DUSP5*	*HACD3*
HRH1	*DUSP6*	*HACD4*
HRK	*DUSP7*	*HAP1*
HRNR	*DUT*	*HAPLN1*
HS1BP3	*DUXA*	*HARS*
HS2ST1	*DVL3*	*HAS2*
HS3ST1	*DYDC1*	*HAS3*
HS3ST3B1	*DYNC1I1*	*HAUS3*
HS3ST5	*DYNC1I2*	*HAUS8*
HS6ST1	*DYNLL1*	*HAVCR1*
HS6ST2	*DYNLL2*	*HBEGF*

HS6ST3	*DYNLT1*	*HBS1L*
HSD17B12	*DYNLT3*	*HCAR1*
HSD17B13	*DYRK1A*	*HCFC2*
HSF5	*DYRK2*	*HCN1*
HSP90AA1	*DYRK3*	*HCN3*
HSPA13	*DYSF*	*HCN4*
HSPA14	*DZIP1L*	*HDAC11*
HSPB3	*DZIP3*	*HDAC4*
HSPBAP1	*E2F1*	*HDAC6*
HSPE1-MOB4	*E2F2*	*HDAC8*
HTR7	*E2F3*	*HDGF*
HTT	*E2F5*	*HDGFL1*
HUNK	*E2F7*	*HDHD2*
HUS1	*EAF2*	*HDLBP*
HYPK	*EBAG9*	*HDX*
IARS	*EBF1*	*HEATR1*
IBA57	*EBF2*	*HEBP2*
IBSP	*EBF3*	*HECTD1*
ICAM1	*EBF4*	*HECTD2*
ICE2	*EBNA1BP2*	*HECW1*
ICK	*EBPL*	*HECW2*
ICMT	*ECD*	*HEG1*
IER3IP1	*ECE1*	*HEMK1*
IFI44L	*ECHDC2*	*HEPACAM*
IFIT1	*ECHDC3*	*HERC2*
IFIT1B	*ECI2*	*HERC4*
IFIT2	*ECM2*	*HES4*
IFIT5	*ECPAS*	*HES6*
IFNAR2	*ECRG4*	*HES7*
IFNE	*ECT2*	*HEXIM1*
IFNGR1	*EDA*	*HEYL*
IFNLR1	*EDA2R*	*HGFAC*
IFRD1	*EDC3*	*HGH1*
IFT20	*EDEM1*	*HHAT*
IFT22	*EDEM3*	*HHIP*
IFT81	*EDF1*	*HHIPL1*
IGDCC3	*EDIL3*	*HIBCH*
IGDCC4	*EDN1*	*HIC2*
IGF1	*EDN3*	*HIF1A*
IGF2BP1	*EDNRA*	*HIF1AN*
IGF2BP2	*EDNRB*	*HIGD1A*
IGFBP4	*EDRF1*	*HIKESHI*
IGFBPL1	*EEA1*	*HINT3*
IGHMBP2	*EEF1AKMT2*	*HIP1*

IGLON5	*EEF1D*	*HIPK1*
IGSF11	*EEF1E1*	*HIPK2*
IGSF3	*EEF2K*	*HIPK3*
IKZF1	*EEF2KMT*	*HIST1H1C*
IKZF4	*EEPD1*	*HIST1H2AC*
IKZF5	*EFCAB1*	*HIST1H2AE*
IL12RB1	*EFCAB10*	*HIST1H2AG*
IL12RB2	*EFCAB11*	*HIST1H2BD*
IL13	*EFCAB12*	*HIST1H3D*
IL13RA1	*EFCAB14*	*HIST1H3H*
IL17D	*EFCAB2*	*HIST2H2AC*
IL17RB	*EFCAB7*	*HIST2H3D*
IL17REL	*EFEMP1*	*HIVEP1*
IL18BP	*EFHC1*	*HK2*
IL1RAP	*EFHC2*	*HLA-DOA*
IL1RAPL1	*EFNA2*	*HLA-DPB1*
IL1RL1	*EFNA4*	*HLTF*
IL2RG	*EFNA5*	*HLX*
IL36RN	*EFR3A*	*HMBOX1*
IL5RA	*EFR3B*	*HMG20A*
IL6R	*EGF*	*HMGB2*
ILDR2	*EGFR*	*HMGB3*
ILK	*EGLN1*	*HMGCLL1*
IMMP2L	*EGLN3*	*HMGCR*
IMPA2	*EGR1*	*HMGCS2*
IMPACT	*EGR2*	*HMGN4*
IMPAD1	*EGR3*	*HMGXB4*
IMPG2	*EHD1*	*HMOX1*
INA	*EHD3*	*HNF1A*
INAVA	*EHF*	*HNF4A*
INCENP	*EHHADH*	*HNF4G*
ING2	*EHMT1*	*HNMT*
ING3	*EI24*	*HNRNPA0*
INIP	*EID2*	*HNRNPA3*
INKA2	*EID2B*	*HNRNPD*
INMT	*EIF1AX*	*HNRNPH3*
INO80D	*EIF1AY*	*HNRNPR*
INPP4A	*EIF2A*	*HNRNPU*
INPP4B	*EIF2AK1*	*HNRNPUL2*
INPP5A	*EIF2AK2*	*HOGA1*
INSC	*EIF2B1*	*HOMER1*
INSIG1	*EIF2B2*	*HOMEZ*
INSIG2	*EIF2B3*	*HOOK1*
INSM1	*EIF2S1*	*HOOK3*

INSYN2B	*EIF2S3*	*HOXA10*
INTS2	*EIF2S3B*	*HOXA13*
INTU	*EIF3A*	*HOXA7*
IP6K1	*EIF3H*	*HOXA9*
IPMK	*EIF3J*	*HOXB13*
IPO11-LRRC70	*EIF3M*	*HOXB2*
IPO13	*EIF4A1*	*HOXB5*
IPO5	*EIF4A3*	*HOXB7*
IPO7	*EIF4E*	*HOXB9*
IPO9	*EIF4E2*	*HOXC10*
IPP	*EIF4E3*	*HOXC5*
IQCC	*EIF4EBP1*	*HOXC8*
IQCH	*EIF4EBP2*	*HOXD1*
IQCJ	*EIF5*	*HOXD10*
IQGAP1	*EIF5A2*	*HOXD12*
IQGAP2	*EIF5AL1*	*HOXD13*
IQSEC3	*EIF5B*	*HOXD4*
IRAK1	*ELAC1*	*HOXD8*
IRF2	*ELAC2*	*HP1BP3*
IRF2BP2	*ELAVL1*	*HPCAL4*
IRF4	*ELAVL4*	*HPGD*
IRF6	*ELF2*	*HPS5*
IRGQ	*ELF4*	*HPSE*
IRS1	*ELK1*	*HPSE2*
IRS4	*ELK3*	*HPX*
IRX5	*ELK4*	*HRH1*
ISCA1	*ELL*	*HRK*
ISG20L2	*ELL2*	*HRNR*
ISL1	*ELMO2*	*HS1BP3*
ISM2	*ELMOD1*	*HS2ST1*
IST1	*ELMOD2*	*HS3ST1*
ISY1	*ELOC*	*HS3ST3B1*
ISY1-RAB43	*ELOVL2*	*HS3ST5*
ITGA1	*ELOVL3*	*HS6ST1*
ITGA10	*ELOVL4*	*HS6ST2*
ITGA11	*ELOVL5*	*HS6ST3*
ITGA2	*ELOVL6*	*HSD17B12*
ITGA3	*ELOVL7*	*HSD17B13*
ITGA9	*ELP1*	*HSF5*
ITGB1BP1	*ELP2*	*HSP90AA1*
ITGB3	*ELP3*	*HSPA13*
ITGB3BP	*ELP4*	*HSPA14*
ITGB8	*ELP6*	*HSPA1B*
ITPA	*EMB*	*HSPB3*

ITPKB	*EMC1*	*HSPE1-MOB4*
ITPR1	*EMC2*	*HSPH1*
ITPR2	*EMC7*	*HTR5A*
ITPRIPL2	*EMCN*	*HTR5A-AS1*
ITSN1	*EML1*	*HTR6*
IWS1	*EML4*	*HTR7*
IYD	*EML5*	*HTT*
JADE2	*EML6*	*HUNK*
JAK3	*EMP1*	*HUS1*
JAKMIP2	*EMP2*	*IBA57*
JAKMIP3	*EN1*	*IBSP*
JAM2	*EN2*	*ICA1L*
JAM3	*ENAH*	*ICAM1*
JCAD	*ENAM*	*ICE2*
JHY	*ENC1*	*ICK*
JMY	*ENDOD1*	*ICMT*
JPH2	*ENDOV*	*IDE*
JPH3	*ENGASE*	*IDS*
JPT2	*ENKUR*	*IER3IP1*
JTB	*ENOPH1*	*IFI44L*
KANSL1	*ENOSF1*	*IFIT1*
KARS	*ENOX2*	*IFIT1B*
KAT14	*ENPEP*	*IFIT2*
KAT6B	*ENPP4*	*IFIT5*
KATNBL1	*ENPP5*	*IFNAR2*
KAZALD1	*ENPP6*	*IFNE*
KAZN	*ENSA*	*IFNGR1*
KBTBD11	*ENTHD1*	*IFNLR1*
KBTBD12	*ENTPD1*	*IFRD1*
KBTBD13	*ENTPD3*	*IFT20*
KBTBD3	*ENTPD4*	*IFT22*
KBTBD6	*ENTPD5*	*IFT74*
KBTBD7	*ENTPD7*	*IGDCC3*
KCMF1	*ENTR1*	*IGDCC4*
KCNA1	*ENY2*	*IGF1*
KCNA5	*EOGT*	*IGF2BP1*
KCNA6	*EOMES*	*IGF2BP2*
KCNA7	*EPAS1*	*IGFBP4*
KCNB1	*EPB41L1*	*IGFBP5*
KCNC1	*EPB41L2*	*IGFBPL1*
KCNC2	*EPB41L3*	*IGHMBP2*
KCND3	*EPB41L4B*	*IGLON5*
KCNE1	*EPB41L5*	*IGSF11*
KCNE3	*EPC2*	*IGSF3*

KCNF1	*EPGN*	*IKBIP*
KCNG3	*EPHA10*	*IKZF1*
KCNG4	*EPHA3*	*IKZF4*
KCNH1	*EPHA4*	*IKZF5*
KCNH8	*EPHA5*	*IL12RB1*
KCNJ1	*EPHA6*	*IL12RB2*
KCNJ11	*EPHA7*	*IL13*
KCNJ5	*EPHX2*	*IL13RA1*
KCNJ6	*EPM2A*	*IL17D*
KCNK1	*EPM2AIP1*	*IL17RA*
KCNK10	*EPN2*	*IL17RB*
KCNK12	*EPN3*	*IL17REL*
KCNK2	*EPPIN*	*IL18BP*
KCNK5	*EPSTI1*	*IL1RAP*
KCNMA1	*EPYC*	*IL1RAPL1*
KCNMB3	*ERAP1*	*IL1RL1*
KCNMB4	*ERAP2*	*IL27RA*
KCNN3	*ERBB3*	*IL2RG*
KCNQ3	*ERBB4*	*IL36G*
KCNS1	*ERBIN*	*IL36RN*
KCNS2	*ERC1*	*IL5RA*
KCNT2	*ERC2*	*IL6R*
KCNV2	*ERCC1*	*IL6ST*
KCTD10	*ERCC4*	*IL7R*
KCTD12	*ERCC6*	*ILDR2*
KCTD16	*ERCC6L*	*IMMP2L*
KCTD2	*ERCC6L2*	*IMPA2*
KCTD20	*EREG*	*IMPACT*
KCTD21	*ERFE*	*IMPAD1*
KCTD6	*ERG28*	*IMPDH1*
KCTD7	*ERGIC1*	*IMPG2*
KDELR1	*ERI1*	*INA*
KDELR2	*ERICH3*	*INAVA*
KDM2A	*ERICH4*	*INCENP*
KDM4E	*ERLEC1*	*INF2*
KDM5A	*ERLIN2*	*ING3*
KDM5B	*ERMAP*	*INIP*
KDM6A	*ERMN*	*INKA2*
KDM7A	*ERMP1*	*INMT*
KDR	*ERN1*	*INO80D*
KERA	*ERO1A*	*INPP4A*
KHDRBS2	*ERO1B*	*INPP4B*
KIAA0040	*ERP27*	*INPP5A*
KIAA0232	*ERP29*	*INSC*

KIAA0319	*ERP44*	*INSIG1*
KIAA0408	*ERVMER34-1*	*INSIG2*
KIAA0513	*ERVV-1*	*INSM1*
KIAA0586	*ERVW-1*	*INSM2*
KIAA0825	*ESCO2*	*INSR*
KIAA0895	*ESF1*	*INSYN2A*
KIAA1143	*ESM1*	*INSYN2B*
KIAA1147	*ESR1*	*INTS2*
KIAA1210	*ESRP1*	*INTS6*
KIAA1211	*ESRRG*	*INTU*
KIAA1324	*ESYT3*	*IP6K1*
KIAA1522	*ETFA*	*IPMK*
KIAA1549	*ETFBKMT*	*IPO11-LRRC70*
KIAA1549L	*ETFDH*	*IPO13*
KIAA1586	*ETNK1*	*IPO5*
KIAA1614	*ETNPPL*	*IPO7*
KIAA1755	*ETV1*	*IPO9*
KIAA1841	*ETV3*	*IPPK*
KIF13B	*ETV5*	*IQCC*
KIF14	*ETV6*	*IQCE*
KIF15	*ETV7*	*IQCH*
KIF16B	*EVA1A*	*IQCJ*
KIF18B	*EVI2B*	*IQGAP1*
KIF1B	*EVI5*	*IQGAP2*
KIF1BP	*EVX2*	*IQSEC3*
KIF1C	*EXD2*	*IRAK1*
KIF20A	*EXOC2*	*IRF1*
KIF21B	*EXOC3*	*IRF2*
KIF27	*EXOC3-AS1*	*IRF2BP2*
KIF3B	*EXOC3L4*	*IRF4*
KIF4A	*EXOC4*	*IRF6*
KIF5A	*EXOC6*	*IRGQ*
KIF5C	*EXOC6B*	*IRS1*
KIF6	*EXOSC1*	*IRS4*
KIN	*EXOSC3*	*IRX5*
KIR3DL1	*EXOSC6*	*ISCA1*
KIR3DL2	*EXPH5*	*ISCA2*
KIT	*EXT1*	*ISCU*
KITLG	*EXT2*	*ISG20*
KLC1	*EXTL2*	*ISG20L2*
KLF10	*EXTL3*	*ISL1*
KLF12	*EYA1*	*ISM2*
KLF13	*EYA3*	*IST1*
KLF3	*EYA4*	*ISY1*

KLF7	*EZH1*	*ISY1-RAB43*
KLF8	*F11*	*ITGA1*
KLF9	*F11R*	*ITGA10*
KLHDC10	*F13A1*	*ITGA11*
KLHDC3	*F13B*	*ITGA2*
KLHDC7A	*F2R*	*ITGA3*
KLHL1	*F2RL1*	*ITGA4*
KLHL11	*F2RL2*	*ITGA9*
KLHL14	*F3*	*ITGB3*
KLHL18	*F5*	*ITGB3BP*
KLHL2	*F7*	*ITGB8*
KLHL21	*F8*	*ITIH5*
KLHL23	*F8A2*	*ITPA*
KLHL24	*F9*	*ITPR1*
KLHL28	*FAAP20*	*ITPR2*
KLHL3	*FABP1*	*ITPRIPL2*
KLHL31	*FABP2*	*ITSN1*
KLHL36	*FABP4*	*IWS1*
KLHL38	*FABP5*	*IYD*
KLHL4	*FADS1*	*JAK3*
KLHL42	*FAF1*	*JAKMIP2*
KLHL5	*FAF2*	*JAKMIP3*
KLHL6	*FAH*	*JAM2*
KLHL7	*FAHD1*	*JAM3*
KLHL8	*FAIM2*	*JCAD*
KLK2	*FAM102A*	*JMJD4*
KLRB1	*FAM102B*	*JMY*
KLRC3	*FAM104A*	*JPH2*
KLRC4	*FAM104B*	*JPH3*
KMT2A	*FAM107A*	*KALRN*
KMT2C	*FAM107B*	*KANSL1*
KMT2D	*FAM110B*	*KANSL3*
KMT5B	*FAM110C*	*KARS*
KNSTRN	*FAM110D*	*KAT14*
KPNA1	*FAM111A*	*KAT6B*
KPNA2	*FAM111B*	*KATNBL1*
KPNA3	*FAM114A1*	*KAZALD1*
KPNA4	*FAM114A2*	*KAZN*
KPNA5	*FAM117B*	*KBTBD11*
KPNA6	*FAM118A*	*KBTBD12*
KPNB1	*FAM120A*	*KBTBD13*
KRIT1	*FAM120B*	*KBTBD2*
KRT222	*FAM120C*	*KBTBD6*
KRT23	*FAM122B*	*KBTBD7*

KRT31	*FAM122C*	*KCMF1*
KRT35	*FAM124A*	*KCNA1*
KRT77	*FAM124B*	*KCNA5*
KRT78	*FAM126A*	*KCNA6*
KRT81	*FAM126B*	*KCNA7*
KRT86	*FAM131B*	*KCNB1*
KRTAP4-4	*FAM133B*	*KCNC1*
KRTAP4-5	*FAM135A*	*KCND3*
KRTAP4-7	*FAM135B*	*KCNE1*
KRTAP4-9	*FAM13A*	*KCNE2*
KRTAP5-8	*FAM13B*	*KCNE3*
KRTAP8-1	*FAM149B1*	*KCNG3*
KRTCAP2	*FAM151B*	*KCNG4*
KSR1	*FAM153A*	*KCNH1*
KSR2	*FAM155A*	*KCNH8*
KYNU	*FAM155B*	*KCNIP1*
L2HGDH	*FAM160A2*	*KCNJ1*
L3MBTL1	*FAM160B1*	*KCNJ11*
LACC1	*FAM160B2*	*KCNJ15*
LAMA4	*FAM161A*	*KCNJ5*
LAMB3	*FAM162A*	*KCNJ6*
LAMP3	*FAM162B*	*KCNK1*
LAMTOR3	*FAM163A*	*KCNK10*
LANCL3	*FAM167A*	*KCNK12*
LARGE1	*FAM168A*	*KCNK2*
LARP1	*FAM168B*	*KCNK5*
LARP4	*FAM169A*	*KCNMA1*
LATS1	*FAM170B*	*KCNMB2*
LAYN	*FAM171B*	*KCNMB3*
LBH	*FAM172A*	*KCNMB4*
LBHD1	*FAM174B*	*KCNN3*
LBP	*FAM177A1*	*KCNQ3*
LBR	*FAM180A*	*KCNS1*
LCA5	*FAM184B*	*KCNS2*
LCK	*FAM186A*	*KCNT2*
LCLAT1	*FAM189B*	*KCNV2*
LCOR	*FAM199X*	*KCTD10*
LDB3	*FAM200A*	*KCTD12*
LDHA	*FAM204A*	*KCTD16*
LDHAL6B	*FAM20A*	*KCTD2*
LDHC	*FAM20B*	*KCTD20*
LDLR	*FAM210B*	*KCTD21*
LDLRAD2	*FAM216B*	*KCTD6*
LDLRAD4	*FAM217A*	*KCTD7*

LDLRAP1	*FAM217B*	*KDELR1*
LEP	*FAM221B*	*KDELR2*
LEPROT	*FAM227A*	*KDM2A*
LETM1	*FAM229B*	*KDM4E*
LETMD1	*FAM241A*	*KDM5A*
LFNG	*FAM3C*	*KDM5B*
LGALS3BP	*FAM47E*	*KDM6A*
LGALSL	*FAM49A*	*KDM7A*
LGI2	*FAM49B*	*KDR*
LGI3	*FAM53A*	*KERA*
LGMN	*FAM53C*	*KHDRBS2*
LHCGR	*FAM72A*	*KHSRP*
LHFPL2	*FAM72B*	*KIAA0040*
LHFPL4	*FAM72C*	*KIAA0087*
LHPP	*FAM72D*	*KIAA0232*
LHX4	*FAM76A*	*KIAA0319*
LHX8	*FAM76B*	*KIAA0408*
LHX9	*FAM81A*	*KIAA0513*
LIFR	*FAM83A*	*KIAA0586*
LILRB4	*FAM83B*	*KIAA0825*
LIMCH1	*FAM83E*	*KIAA0895*
LIMS1	*FAM83F*	*KIAA1143*
LIMS2	*FAM86B1*	*KIAA1147*
LIN28A	*FAM86C1*	*KIAA1210*
LIN54	*FAM89A*	*KIAA1211*
LINC00346	*FAM8A1*	*KIAA1324*
LINC01546	*FAM91A1*	*KIAA1324L*
LINC01559	*FAM92A*	*KIAA1522*
LINS1	*FAM98A*	*KIAA1549*
LIPC	*FAM98B*	*KIAA1549L*
LIPG	*FAM9A*	*KIAA1586*
LIPT2	*FAM9C*	*KIAA1755*
LITAF	*FAN1*	*KIAA1841*
LIX1	*FANCA*	*KIAA1958*
LLPH	*FANCB*	*KIF13B*
LMAN2L	*FANCC*	*KIF14*
LMBR1	*FANCG*	*KIF15*
LMBRD2	*FANCI*	*KIF16B*
LMO3	*FANCM*	*KIF18B*
LMO4	*FAR1*	*KIF1A*
LMOD1	*FAR2*	*KIF1B*
LMTK2	*FARP1*	*KIF1C*
LMX1A	*FARSB*	*KIF20A*
LMX1B	*FAS*	*KIF21A*

LNP1	*FASLG*	*KIF21B*
LNPEP	*FASTKD1*	*KIF24*
LNPK	*FASTKD2*	*KIF27*
LNX1	*FAT1*	*KIF3B*
LNX2	*FAT4*	*KIF5A*
LOC100129215	*FAXC*	*KIF5C*
LOC100996419	*FBH1*	*KIF6*
LOC105371730	*FBLIM1*	*KIFC2*
LOC107983990	*FBLN2*	*KIN*
LOC388242	*FBLN7*	*KIR3DL1*
LOC400464	*FBN1*	*KIR3DL2*
LOC401589	*FBN2*	*KIT*
LOC613038	*FBXL14*	*KLC1*
LOC644656	*FBXL18*	*KLF10*
LOC730098	*FBXL20*	*KLF11*
LOC730338	*FBXL22*	*KLF12*
LONP2	*FBXL3*	*KLF13*
LONRF2	*FBXL4*	*KLF3*
LONRF3	*FBXL5*	*KLF7*
LOXL3	*FBXL6*	*KLF8*
LPAR1	*FBXL7*	*KLF9*
LPAR3	*FBXO11*	*KLHDC10*
LPAR5	*FBXO16*	*KLHDC3*
LPCAT1	*FBXO17*	*KLHDC7A*
LPCAT2	*FBXO22*	*KLHDC8A*
LPGAT1	*FBXO28*	*KLHL1*
LPP	*FBXO3*	*KLHL11*
LRATD1	*FBXO30*	*KLHL14*
LRATD2	*FBXO31*	*KLHL18*
LRCH2	*FBXO32*	*KLHL2*
LRFN2	*FBXO33*	*KLHL21*
LRIF1	*FBXO34*	*KLHL23*
LRIG3	*FBXO40*	*KLHL24*
LRP10	*FBXO42*	*KLHL28*
LRP6	*FBXO43*	*KLHL3*
LRRC14	*FBXO44*	*KLHL30*
LRRC14B	*FBXO45*	*KLHL31*
LRRC15	*FBXO47*	*KLHL36*
LRRC19	*FBXO48*	*KLHL38*
LRRC2	*FBXO5*	*KLHL4*
LRRC20	*FBXO9*	*KLHL5*
LRRC28	*FBXW11*	*KLHL6*
LRRC3	*FBXW2*	*KLHL7*
LRRC34	*FBXW7*	*KLHL8*

LRRC58	*FBXW8*	*KLK2*
LRRC7	*FCAR*	*KLK3*
LRRC75A	*FCER1A*	*KLRB1*
LRRC8C	*FCF1*	*KLRC3*
LRRCC1	*FCGR1A*	*KLRC4*
LRRN1	*FCGR1B*	*KLRG2*
LRRTM2	*FCGR2B*	*KMT2A*
LRSAM1	*FCGR3A*	*KMT2C*
LSAMP	*FCGR3B*	*KMT5B*
LSG1	*FCHO2*	*KNSTRN*
LSM10	*FCN2*	*KPNA1*
LSM14A	*FCRL1*	*KPNA2*
LSM6	*FCRL3*	*KPNA3*
LSM8	*FCRL4*	*KPNA4*
LTBP2	*FCRL5*	*KPNA5*
LTF	*FCRL6*	*KPNA6*
LTO1	*FCRLB*	*KREMEN1*
LUC7L	*FDX1*	*KRIT1*
LUC7L2	*FDXACB1*	*KRT222*
LUC7L3	*FECH*	*KRT23*
LUZP2	*FEM1A*	*KRT31*
LY6E	*FEM1B*	*KRT35*
LY75	*FEM1C*	*KRT77*
LY9	*FER*	*KRT78*
LYN	*FERMT1*	*KRT81*
LYPD6	*FERMT2*	*KRT86*
LYPLA1	*FERMT3*	*KRTAP4-4*
LYRM2	*FEZ1*	*KRTAP4-5*
LYRM4	*FGA*	*KRTAP5-8*
LYRM7	*FGB*	*KRTAP8-1*
LYRM9	*FGD1*	*KRTCAP2*
LYSMD1	*FGD2*	*KSR1*
LYSMD2	*FGD4*	*KSR2*
LZTFL1	*FGD6*	*KYNU*
LZTS1	*FGF1*	*L1TD1*
M1AP	*FGF10*	*L2HGDH*
M6PR	*FGF12*	*L3MBTL1*
MAB21L1	*FGF13*	*LACC1*
MACF1	*FGF14*	*LAD1*
MAD2L1	*FGF16*	*LAIR1*
MAGEB10	*FGF18*	*LAMA4*
MAGEL2	*FGF19*	*LAMB3*
MAGI1	*FGF2*	*LAMC1*
MAGI2	*FGF5*	*LAMP3*

MAGOHB	*FGF9*	*LAMTOR3*
MAK16	*FGFBP1*	*LAMTOR5*
MALSU1	*FGFBP2*	*LANCL3*
MAML1	*FGFBP3*	*LARGE1*
MAML3	*FGFR1OP*	*LARP1*
MAMLD1	*FGFR1OP2*	*LARP4*
MAN1A1	*FGG*	*LARS2*
MAN1C1	*FGL2*	*LATS1*
MAN2A1	*FHDC1*	*LATS2*
MAN2B2	*FHL1*	*LAYN*
MANEAL	*FHL2*	*LBH*
MANSC1	*FHL3*	*LBHD1*
MAP1B	*FHL5*	*LBP*
MAP1LC3B	*FIBIN*	*LBR*
MAP2K4	*FIG4*	*LCA5*
MAP2K6	*FIGN*	*LCK*
MAP3K10	*FIGNL1*	*LCLAT1*
MAP3K13	*FILIP1*	*LCOR*
MAP3K20	*FILIP1L*	*LDB3*
MAP3K3	*FIP1L1*	*LDHA*
MAP3K4	*FITM2*	*LDHAL6B*
MAP3K7	*FJX1*	*LDHB*
MAP3K8	*FKBP14*	*LDLR*
MAP3K9	*FKBP1B*	*LDLRAD1*
MAP4K2	*FKBP4*	*LDLRAD2*
MAP4K4	*FKBP5*	*LDLRAD4*
MAP4K5	*FKBP7*	*LDLRAP1*
MAP6	*FKBP9*	*LEPROT*
MAP7	*FKRP*	*LETM1*
MAP7D2	*FKTN*	*LETMD1*
MAP9	*FLCN*	*LFNG*
MAPK1	*FLG*	*LGALSL*
MAPK10	*FLG2*	*LGI2*
MAPK13	*FLJ22447*	*LGI3*
MAPK14	*FLRT1*	*LGMN*
MAPK1IP1L	*FLRT2*	*LGR4*
MAPK8	*FLRT3*	*LGSN*
MAPK9	*FLT1*	*LGTN*
MAPKAP1	*FLT3*	*LHCGR*
MAPKAPK5	*FLVCR1*	*LHFPL2*
MAPRE1	*FLYWCH2*	*LHPP*
MAPRE2	*FMC1*	*LHX4*
MAPT	*FMN1*	*LHX8*
MARCH2	*FMN2*	*LHX9*

MARCH3	*FMNL2*	*LIF*
MARCH5	*FMNL3*	*LIFR*
MARCH6	*FMO3*	*LILRB4*
MARCH7	*FMOD*	*LIMCH1*
MARCH8	*FMR1*	*LIMS1*
MARF1	*FN1*	*LIMS2*
MARK1	*FN3KRP*	*LIN28A*
MARK4	*FNBP1*	*LIN54*
MARS	*FNBP1L*	*LIN7A*
MARS2	*FNDC3A*	*LINC00346*
MARVELD1	*FNDC3B*	*LINC00908*
MASTL	*FNDC4*	*LINC01460*
MAT1A	*FNIP1*	*LINC01546*
MAT2A	*FNIP2*	*LINC01559*
MAU2	*FNTB*	*LINC01664*
MAVS	*FOPNL*	*LINC01973*
MAZ	*FOSL2*	*LINC02312*
MBD2	*FOXA1*	*LINS1*
MBD5	*FOXA2*	*LIPC*
MBD6	*FOXA3*	*LIPG*
MBLAC2	*FOXB1*	*LIPT2*
MBNL1	*FOXC1*	*LITAF*
MBNL3	*FOXD3*	*LIX1*
MBP	*FOXD4*	*LLPH*
MCC	*FOXD4L1*	*LMAN2L*
MCCC2	*FOXD4L3*	*LMBR1*
MCF2L	*FOXE1*	*LMBRD2*
MCF2L2	*FOXE3*	*LMO3*
MCFD2	*FOXF2*	*LMO4*
MCHR2	*FOXJ2*	*LMO7*
MCL1	*FOXK1*	*LMOD1*
MCM10	*FOXK2*	*LMTK2*
MCM3	*FOXL1*	*LMX1A*
MCM3AP	*FOXN2*	*LMX1B*
MCM7	*FOXN3*	*LNP1*
MCM8	*FOXO1*	*LNPEP*
MCMBP	*FOXO3*	*LNPK*
MCMDC2	*FOXP1*	*LNX1*
MCOLN2	*FOXP2*	*LNX2*
MCOLN3	*FOXR2*	*LOC100129215*
MCTP1	*FPGT*	*LOC100996419*
MCTP2	*FPGT-TNNI3K*	*LOC105371730*
MCTS1	*FPR1*	*LOC107983990*
MCU	*FPR2*	*LOC400464*

MCUR1	*FRA10AC1*	*LOC401589*
MDH2	*FRAS1*	*LOC644656*
MDM1	*FRAT1*	*LOC730098*
MDM2	*FRAT2*	*LOC730338*
MDM4	*FREM2*	*LONP2*
MDN1	*FRG2*	*LONRF2*
MEA1	*FRG2C*	*LONRF3*
MECP2	*FRK*	*LOXL3*
MED1	*FRMD3*	*LPAR1*
MED12L	*FRMD4B*	*LPAR3*
MED13L	*FRMD5*	*LPAR5*
MED17	*FRMD6*	*LPCAT1*
MED21	*FRMD7*	*LPCAT2*
MED28	*FRMPD4*	*LPGAT1*
MED29	*FRRS1*	*LPP*
MED7	*FRRS1L*	*LRAT*
MED8	*FRS2*	*LRATD1*
MED9	*FRYL*	*LRATD2*
MEF2C	*FRZB*	*LRCH1*
MEF2D	*FSBP*	*LRCH2*
MEGF9	*FSCB*	*LRFN2*
MEI4	*FSCN1*	*LRIF1*
MEIS1	*FSD1L*	*LRIG3*
MELK	*FSD2*	*LRP10*
MELTF	*FSHB*	*LRP12*
MEMO1	*FSHR*	*LRP5L*
MEOX1	*FST*	*LRP6*
MESD	*FSTL1*	*LRRC14*
MESP1	*FSTL4*	*LRRC14B*
MEST	*FSTL5*	*LRRC15*
METAP1	*FTCDNL1*	*LRRC19*
METTL14	*FTO*	*LRRC2*
METTL15	*FTSJ3*	*LRRC20*
METTL16	*FUBP1*	*LRRC28*
METTL21A	*FUBP3*	*LRRC3*
METTL22	*FUCA1*	*LRRC34*
METTL24	*FUNDC2*	*LRRC40*
METTL2A	*FURIN*	*LRRC57*
METTL8	*FUS*	*LRRC58*
MEX3A	*FUT11*	*LRRC7*
MEX3B	*FUT4*	*LRRC75A*
MFAP3L	*FUT5*	*LRRC8B*
MFN1	*FUT6*	*LRRC8C*
MFN2	*FUT8*	*LRRC8D*

MFSD13A	*FUT9*	*LRRCC1*
MFSD14C	*FXN*	*LRRN1*
MFSD4A	*FXR1*	*LRRTM2*
MFSD6	*FXYD5*	*LRSAM1*
MFSD9	*FYB1*	*LSAMP*
MGA	*FYB2*	*LSG1*
MGAT2	*FYCO1*	*LSM10*
MGAT4B	*FYN*	*LSM14A*
MGAT5	*FYTTD1*	*LSM6*
MGMT	*FZD1*	*LSM8*
MGP	*FZD2*	*LSMEM1*
MGST3	*FZD3*	*LTBP2*
MIA3	*FZD4*	*LTF*
MIB1	*FZD5*	*LTO1*
MICA	*FZD6*	*LUC7L*
MICAL3	*FZD7*	*LUC7L3*
MICOS10	*FZD8*	*LUZP2*
MIDN	*FZR1*	*LY6D*
MIEF1	*G2E3*	*LY6E*
MIEF2	*G3BP1*	*LY6K*
MIER1	*G3BP2*	*LY75*
MIER3	*G6PC*	*LY9*
MIGA1	*G6PC2*	*LYN*
MIGA2	*GAB1*	*LYPD6*
MINAR1	*GAB2*	*LYPLA1*
MINDY2	*GAB3*	*LYPLA2*
MINK1	*GABARAPL1*	*LYRM2*
MIP	*GABBR2*	*LYRM4*
MIPOL1	*GABPA*	*LYRM7*
MIR2052HG	*GABPB1*	*LYRM9*
MIS12	*GABPB2*	*LYSMD1*
MITF	*GABRA1*	*LYSMD2*
MKI67	*GABRA3*	*LYVE1*
MKKS	*GABRA4*	*LZTFL1*
MKLN1	*GABRA5*	*LZTS1*
MKNK1	*GABRB1*	*M1AP*
MLEC	*GABRB3*	*M6PR*
MLIP	*GABRE*	*MAB21L1*
MLLT10	*GABRG1*	*MACROD2*
MLX	*GABRP*	*MAD2L1*
MLXIP	*GABRR1*	*MAGEB10*
MMD2	*GABRR2*	*MAGEB2*
MME	*GAD1*	*MAGEL2*
MMP1	*GAD2*	*MAGI1*

MMP13	*GADL1*	*MAGI2*
MMP14	*GAGE1*	*MAGI3*
MMP16	*GALK2*	*MAGOHB*
MMP19	*GALNS*	*MAK16*
MMP24	*GALNT1*	*MALSU1*
MNT	*GALNT10*	*MAML1*
MOAP1	*GALNT13*	*MAML3*
MOB1A	*GALNT15*	*MAMLD1*
MOB1B	*GALNT17*	*MAN1C1*
MOB3B	*GALNT3*	*MAN2A1*
MOB4	*GALNT4*	*MAN2B2*
MOBP	*GALNT5*	*MANEAL*
MOCS2	*GALNT6*	*MAP1B*
MOCS3	*GALNT7*	*MAP1LC3B*
MON1B	*GALNT8*	*MAP2K4*
MON2	*GALNTL6*	*MAP2K6*
MORC2	*GALR1*	*MAP3K10*
MORC3	*GALR2*	*MAP3K13*
MORF4L1	*GANC*	*MAP3K2*
MORF4L2	*GAP43*	*MAP3K20*
MORN4	*GAPT*	*MAP3K3*
MOSMO	*GAPVD1*	*MAP3K7*
MOXD1	*GAR1*	*MAP3K8*
MPC2	*GAREM1*	*MAP3K9*
MPHOSPH8	*GARNL3*	*MAP4K2*
MPI	*GART*	*MAP4K4*
MPP5	*GAS1*	*MAP4K5*
MPPE1	*GAS2*	*MAP6*
MPPED1	*GAS2L3*	*MAP7*
MPRIP	*GAS7*	*MAP9*
MPV17L	*GASK1A*	*MAPK1*
MPZL1	*GASK1B*	*MAPK10*
MPZL2	*GATA3*	*MAPK13*
MPZL3	*GATA6*	*MAPK14*
MRAP	*GATAD1*	*MAPK1IP1L*
MRAP2	*GATAD2B*	*MAPK8*
MRAS	*GATC*	*MAPK8IP1*
MRE11	*GATD3A*	*MAPK9*
MRGBP	*GATM*	*MAPKAP1*
MRGPRF	*GBE1*	*MAPRE1*
MRGPRX2	*GBP1*	*MAPRE2*
MRI1	*GBP2*	*MAPT*
MRM3	*GBP3*	*MARCH3*
MRO	*GBP4*	*MARCH5*

MROH1	*GBP6*	*MARCH6*
MROH6	*GBP7*	*MARCH7*
MROH8	*GBX2*	*MARCH8*
MRPL15	*GCA*	*MARF1*
MRPL17	*GCC1*	*MARK1*
MRPL19	*GCC2*	*MARK4*
MRPL22	*GCFC2*	*MARS2*
MRPL3	*GCH1*	*MARVELD1*
MRPL30	*GCLM*	*MASTL*
MRPL33	*GCM2*	*MAT1A*
MRPL50	*GCNT1*	*MAT2A*
MRPL57	*GCNT2*	*MAU2*
MRPS14	*GCNT3*	*MAVS*
MRPS24	*GCOM1*	*MBD1*
MRPS25	*GCSAML*	*MBD2*
MRPS30	*GCSH*	*MBD5*
MRPS35	*GDA*	*MBD6*
MRPS7	*GDAP1*	*MBLAC2*
MRRF	*GDAP2*	*MBNL1*
MRS2	*GDE1*	*MBNL2*
MRTFA	*GDF11*	*MBNL3*
MRTO4	*GDF6*	*MBOAT7*
MS4A15	*GDF7*	*MBP*
MSANTD2	*GDI1*	*MC2R*
MSANTD3	*GDI2*	*MCC*
MSANTD4	*GDNF*	*MCCC2*
MSH3	*GDPD1*	*MCEMP1*
MSH6	*GDPD4*	*MCF2L*
MSI2	*GDPGP1*	*MCF2L2*
MSL1	*GEMIN2*	*MCFD2*
MSL2	*GEMIN4*	*MCHR2*
MSL3	*GEMIN7*	*MCM10*
MSN	*GEMIN8*	*MCM3AP*
MSRB3	*GEN1*	*MCM8*
MSTN	*GFI1*	*MCMBP*
MT1A	*GFM1*	*MCMDC2*
MT1F	*GFM2*	*MCOLN2*
MTA2	*GFOD1*	*MCOLN3*
MTDH	*GFPT1*	*MCPH1*
MTERF1	*GFRA1*	*MCTP1*
MTERF2	*GFRA2*	*MCTP2*
MTERF4	*GGA2*	*MCTS1*
MTF1	*GGCX*	*MCU*
MTFR1	*GHITM*	*MCUR1*

MTFR2	*GHR*	*MDFIC*
MTHFD2	*GHRL*	*MDM1*
MTHFS	*GHSR*	*MDM2*
MTMR1	*GID8*	*MDM4*
MTMR10	*GIMAP1*	*MDN1*
MTMR2	*GIMAP4*	*MECP2*
MTMR3	*GIMAP5*	*MED1*
MTMR6	*GIMAP8*	*MED11*
MTMR8	*GIN1*	*MED12L*
MTMR9	*GINM1*	*MED13L*
MTR	*GINS1*	*MED17*
MTRNR2L3	*GINS2*	*MED20*
MTRNR2L4	*GINS3*	*MED21*
MTRNR2L5	*GINS4*	*MED23*
MTRNR2L6	*GIPC2*	*MED28*
MTURN	*GIPC3*	*MED29*
MTX3	*GIPR*	*MED7*
MUC15	*GIT2*	*MED8*
MUC21	*GJA3*	*MED9*
MUCL3	*GJB2*	*MEDAG*
MUSK	*GJB4*	*MEF2A*
MXD1	*GJB6*	*MEF2C*
MXD3	*GJB7*	*MEF2D*
MXD4	*GJC1*	*MEGF8*
MXI1	*GJE1*	*MEGF9*
MYB	*GK*	*MEI4*
MYBL1	*GK2*	*MEIS1*
MYC	*GK5*	*MELK*
MYCL	*GKN1*	*MELTF*
MYCT1	*GLA*	*MEMO1*
MYEF2	*GLB1*	*MEOX2*
MYH9	*GLCCI1*	*MEPE*
MYL2	*GLDC*	*MESD*
MYLIP	*GLDN*	*MESP1*
MYLK3	*GLG1*	*MEST*
MYNN	*GLI3*	*METAP1*
MYO10	*GLIPR1*	*METAP2*
MYO16	*GLIPR1L2*	*METTL14*
MYO1F	*GLIPR2*	*METTL15*
MYO5A	*GLIS3*	*METTL16*
MYO6	*GLMN*	*METTL21A*
MYOD1	*GLO1*	*METTL22*
MYOG	*GLOD4*	*METTL24*
MYOM2	*GLP2R*	*METTL2A*

MYORG	*GLRA2*	*METTL8*
MYOZ2	*GLRA3*	*MEX3A*
MYT1	*GLRB*	*MEX3B*
MYT1L	*GLRX*	*MFAP3L*
N4BP1	*GLRX2*	*MFN1*
N4BP3	*GLS*	*MFN2*
N6AMT1	*GLTP*	*MFSD14C*
NAA15	*GLUD1*	*MFSD2A*
NAA25	*GLUD2*	*MFSD4A*
NAA30	*GLUL*	*MFSD6*
NAA35	*GLYAT*	*MFSD9*
NAA38	*GLYATL2*	*MGA*
NAA50	*GLYATL3*	*MGAT5*
NAA60	*GLYR1*	*MGME1*
NAAA	*GM2A*	*MGMT*
NAALADL2	*GMCL1*	*MGP*
NABP1	*GMEB1*	*MGST3*
NABP2	*GMEB2*	*MIA3*
NADK	*GMFB*	*MICA*
NAF1	*GMNC*	*MICAL3*
NAGS	*GMPR*	*MICOS10*
NANOS1	*GMPS*	*MIDN*
NANOS2	*GNA11*	*MIEF1*
NANP	*GNA12*	*MIEN1*
NAP1L1	*GNA13*	*MIER1*
NAP1L2	*GNAI3*	*MIER3*
NAP1L3	*GNAL*	*MIGA2*
NAP1L4	*GNAO1*	*MINAR1*
NAP1L5	*GNAQ*	*MINDY2*
NAPA	*GNAS*	*MIPOL1*
NAPEPLD	*GNAT1*	*MIR2052HG*
NAPG	*GNB1*	*MIS12*
NARS	*GNB4*	*MITF*
NAT1	*GNE*	*MKI67*
NAT8	*GNG10*	*MKKS*
NBEA	*GNG12*	*MKLN1*
NBEAL1	*GNG2*	*MKNK1*
NBEAL2	*GNG4*	*MKRN3*
NBN	*GNG5*	*MLEC*
NBPF3	*GNG7*	*MLH3*
NCALD	*GNGT2*	*MLIP*
NCAPD2	*GNL1*	*MLLT10*
NCAPD3	*GNL3*	*MLLT3*
NCAPG	*GNL3L*	*MLX*

NCAPH	*GNPAT*	*MLXIP*
NCAPH2	*GNPDA1*	*MMD2*
NCBP1	*GNPDA2*	*MME*
NCF1	*GNPTAB*	*MMP1*
NCK1	*GNPTG*	*MMP14*
NCK2	*GNRH1*	*MMP16*
NCKAP1	*GNRHR*	*MMP19*
NCKAP5	*GOLGA1*	*MMP2*
NCKAP5L	*GOLGA3*	*MMP24*
NCKIPSD	*GOLGA6A*	*MNT*
NCOA7	*GOLGA6B*	*MOAP1*
NCOR1	*GOLGA6C*	*MOB1A*
NCR3LG1	*GOLGA6D*	*MOB1B*
NCS1	*GOLGA6L4*	*MOB3B*
NDC1	*GOLGA7*	*MOB3C*
NDE1	*GOLGA7B*	*MOB4*
NDEL1	*GOLGA8B*	*MOBP*
NDFIP1	*GOLGA8F*	*MOCS2*
NDNF	*GOLGA8H*	*MOCS3*
NDOR1	*GOLGA8K*	*MON1B*
NDST3	*GOLGA8M*	*MON2*
NDUFA1	*GOLGA8N*	*MORC2*
NDUFA5	*GOLGA8O*	*MORC3*
NDUFA7	*GOLGA8R*	*MORF4L1*
NDUFAF6	*GOLGB1*	*MORF4L2*
NDUFB3	*GOLIM4*	*MORN4*
NDUFB5	*GOLM1*	*MOSMO*
NDUFC1	*GOLPH3L*	*MOXD1*
NDUFC2	*GOLT1B*	*MPC2*
NDUFV3	*GON7*	*MPI*
NEBL	*GOPC*	*MPP5*
NECAB1	*GORAB*	*MPPED1*
NECAB3	*GORASP1*	*MPPED2*
NECAP2	*GORASP2*	*MPRIP*
NECTIN1	*GOSR1*	*MPV17L*
NECTIN3	*GOT2*	*MPZL1*
NEFH	*GP1BA*	*MPZL2*
NEGR1	*GP5*	*MPZL3*
NEK2	*GPAM*	*MRAP*
NEK4	*GPANK1*	*MRAP2*
NEK7	*GPAT4*	*MRAS*
NEK8	*GPATCH1*	*MRE11*
NEK9	*GPATCH11*	*MRGBP*
NENF	*GPATCH2*	*MRGPRF*

NEPRO	*GPATCH2L*	*MRGPRX2*
NET1	*GPBP1*	*MRI1*
NETO1	*GPBP1L1*	*MRM3*
NETO2	*GPC1*	*MRO*
NEU3	*GPC4*	*MRPL15*
NEURL1	*GPC5*	*MRPL17*
NEURL1B	*GPC6*	*MRPL19*
NEUROD2	*GPCPD1*	*MRPL22*
NEUROG2	*GPD1L*	*MRPL3*
NEXMIF	*GPD2*	*MRPL30*
NFAT5	*GPHN*	*MRPL33*
NFATC2	*GPKOW*	*MRPL43*
NFATC3	*GPLD1*	*MRPL44*
NFATC4	*GPM6A*	*MRPL50*
NFIB	*GPM6B*	*MRPL57*
NFIC	*GPNMB*	*MRPS14*
NFKBID	*GPR1*	*MRPS18B*
NFS1	*GPR107*	*MRPS18C*
NFYB	*GPR12*	*MRPS23*
NFYC	*GPR135*	*MRPS24*
NGB	*GPR137B*	*MRPS25*
NGRN	*GPR137C*	*MRPS27*
NHLRC2	*GPR141*	*MRPS30*
NHLRC3	*GPR15*	*MRPS35*
NHS	*GPR150*	*MRPS7*
NHSL1	*GPR151*	*MRRF*
NIBAN1	*GPR155*	*MRS2*
NIFK	*GPR156*	*MRTFA*
NINJ1	*GPR157*	*MRTO4*
NIPA1	*GPR158*	*MSANTD2*
NIPA2	*GPR160*	*MSANTD3*
NIPAL2	*GPR161*	*MSANTD4*
NIPAL4	*GPR17*	*MSH2*
NIPBL	*GPR173*	*MSH3*
NISCH	*GPR174*	*MSH6*
NIT1	*GPR176*	*MSI2*
NIT2	*GPR18*	*MSL1*
NKAIN1	*GPR180*	*MSL2*
NKAIN3	*GPR183*	*MSL3*
NKAP	*GPR22*	*MSN*
NKAPD1	*GPR26*	*MSRB3*
NKAPL	*GPR34*	*MSTN*
NKD1	*GPR37*	*MT1A*
NKIRAS2	*GPR39*	*MT1F*

NKRF	*GPR45*	*MTDH*
NKTR	*GPR50*	*MTERF1*
NKX2-1	*GPR62*	*MTERF2*
NKX2-4	*GPR63*	*MTERF4*
NKX3-2	*GPR65*	*MTF1*
NLE1	*GPR75*	*MTF2*
NLGN4X	*GPR78*	*MTFR1*
NLK	*GPR82*	*MTFR2*
NLN	*GPR85*	*MTHFD2*
NLRP1	*GPR88*	*MTHFS*
NLRP3	*GPR89A*	*MTM1*
NLRP9	*GPR89B*	*MTMR1*
NME6	*GPRASP1*	*MTMR10*
NMNAT1	*GPRASP2*	*MTMR2*
NMNAT2	*GPRC5A*	*MTMR3*
NMT2	*GPRC5B*	*MTMR6*
NNT	*GPRIN2*	*MTMR7*
NOA1	*GPRIN3*	*MTMR8*
NOCT	*GPSM2*	*MTMR9*
NOL12	*GPT2*	*MTO1*
NOL4	*GPX8*	*MTR*
NOL9	*GRAMD1A*	*MTRNR2L3*
NOLC1	*GRAMD2A*	*MTRNR2L4*
NOM1	*GRAMD2B*	*MTRNR2L5*
NOMO1	*GRAP2*	*MTRNR2L6*
NOMO3	*GRB14*	*MTSS2*
NOP9	*GREB1*	*MTTP*
NOS1	*GREM1*	*MTURN*
NOS1AP	*GREM2*	*MTUS1*
NOTCH2	*GRHL3*	*MTX3*
NOTCH3	*GRHPR*	*MUC15*
NOVA1	*GRIA1*	*MUC21*
NOVA2	*GRIA2*	*MUCL3*
NOX1	*GRIA4*	*MUSK*
NOX4	*GRID2*	*MVK*
NPAP1	*GRIK2*	*MXD1*
NPAS4	*GRIK3*	*MXD3*
NPBWR1	*GRIK4*	*MXD4*
NPC1	*GRIN2A*	*MXI1*
NPC2	*GRIN2B*	*MYB*
NPFFR1	*GRIN3A*	*MYBL1*
NPHP3	*GRIP1*	*MYBPC1*
NPL	*GRK1*	*MYCBP*
NPLOC4	*GRK3*	*MYCL*

NPNT	*GRK5*	*MYCT1*
NPR2	*GRK7*	*MYEF2*
NPR3	*GRM1*	*MYH9*
NPTX1	*GRM4*	*MYLIP*
NPTXR	*GRM6*	*MYLK3*
NR0B1	*GRPEL1*	*MYNN*
NR1D2	*GRPEL2*	*MYO10*
NR1I2	*GRPR*	*MYO16*
NR2C1	*GRSF1*	*MYO1A*
NR2C2	*GRTP1*	*MYO1F*
NR2C2AP	*GRWD1*	*MYO5A*
NR2E1	*GSC2*	*MYO6*
NR2F6	*GSDMA*	*MYO7A*
NR3C1	*GSDMC*	*MYO9B*
NR5A2	*GSE1*	*MYOCD*
NR6A1	*GSG1*	*MYOD1*
NRARP	*GSK3B*	*MYOG*
NRF1	*GSKIP*	*MYOM2*
NRG1	*GSPT1*	*MYORG*
NRG3	*GSPT2*	*MYOZ2*
NRG4	*GSR*	*MYT1*
NRK	*GSTK1*	*MYT1L*
NRP1	*GSTO1*	*N4BP1*
NRP2	*GSTO2*	*N4BP2L2*
NRXN1	*GTDC1*	*N4BP3*
NRXN3	*GTF2A1*	*N6AMT1*
NSD2	*GTF2A2*	*NAA15*
NSL1	*GTF2B*	*NAA25*
NSMCE3	*GTF2E1*	*NAA30*
NSMCE4A	*GTF2E2*	*NAA35*
NSMF	*GTF2F1*	*NAA38*
NSUN4	*GTF2H2C*	*NAA50*
NSUN5	*GTF2H3*	*NAA60*
NSUN7	*GTF2H5*	*NAAA*
NT5C1A	*GTF3C3*	*NABP1*
NT5C2	*GTF3C4*	*NABP2*
NT5DC1	*GTF3C6*	*NADK*
NT5M	*GTPBP1*	*NAF1*
NTF3	*GTPBP10*	*NAGS*
NTM	*GTPBP4*	*NAIP*
NTPCR	*GTPBP8*	*NAMPT*
NTRK2	*GUCA1A*	*NANOS1*
NTSR1	*GUCA1C*	*NANOS2*
NUAK1	*GUCY1A1*	*NANP*

NUDCD2	*GUCY1A2*	*NAP1L1*
NUDCD3	*GUCY1B1*	*NAP1L2*
NUDT13	*GUCY2C*	*NAP1L3*
NUDT16	*GUF1*	*NAP1L4*
NUDT21	*GULP1*	*NAP1L5*
NUDT3	*GVQW3*	*NAPA*
NUF2	*GXYLT1*	*NAPEPLD*
NUFIP1	*GXYLT2*	*NAPG*
NUFIP2	*GYG1*	*NARS*
NUMBL	*GYG2*	*NAT1*
NUP155	*GYPA*	*NAT10*
NUP160	*GYPE*	*NAT8*
NUP205	*GYS1*	*NBEA*
NUP35	*GYS2*	*NBEAL1*
NUP43	*GZF1*	*NBEAL2*
NUP58	*H1FOO*	*NBN*
NUP88	*H2AFJ*	*NBPF3*
NUP93	*H2AFV*	*NCALD*
NUS1	*H2BFM*	*NCAPD3*
NUTF2	*H3F3B*	*NCAPG*
NUTM2D	*H6PD*	*NCAPH*
NUTM2E	*HAAO*	*NCAPH2*
NUTM2G	*HACD2*	*NCF1*
NWD1	*HACD3*	*NCK1*
NWD2	*HACD4*	*NCK2*
NXN	*HACE1*	*NCKAP1*
NXPH1	*HADHB*	*NCKAP5*
NXT2	*HAL*	*NCKIPSD*
NYAP2	*HAND2*	*NCL*
OAS2	*HAO1*	*NCOA7*
OAS3	*HAPLN1*	*NCOR1*
OAZ2	*HARBI1*	*NCR3LG1*
OCIAD2	*HAS2*	*NCS1*
OCRL	*HAUS2*	*NDE1*
ODAPH	*HAUS3*	*NDEL1*
ODC1	*HAUS5*	*NDFIP1*
ODF2L	*HAUS6*	*NDNF*
OGDH	*HAUS8*	*NDOR1*
OGFOD1	*HAVCR1*	*NDST3*
OGFOD3	*HAVCR2*	*NDUFA1*
OGFRL1	*HBEGF*	*NDUFA5*
OIP5	*HBP1*	*NDUFA7*
OLA1	*HBS1L*	*NDUFAF6*
OLFM1	*HCAR1*	*NDUFB3*

OLFM4	*HCAR2*	*NDUFB5*
OLIG2	*HCAR3*	*NDUFC1*
ONECUT1	*HCCS*	*NDUFC2*
ONECUT2	*HCFC2*	*NDUFS1*
OPA3	*HCLS1*	*NDUFV3*
OPALIN	*HCN1*	*NEBL*
OPCML	*HDAC11*	*NECAB1*
OPHN1	*HDAC2*	*NECAB3*
OPN1MW2	*HDAC4*	*NECAP2*
OPRK1	*HDAC5*	*NECTIN1*
OPRM1	*HDAC8*	*NECTIN3*
OR10H5	*HDAC9*	*NEFH*
OR1L8	*HDGF*	*NEGR1*
OR2A4	*HDGFL3*	*NEIL2*
OR2B11	*HDHD2*	*NEK10*
OR51E1	*HDHD5*	*NEK2*
ORAI2	*HDX*	*NEK7*
ORAI3	*HEATR1*	*NEK8*
ORM1	*HEATR3*	*NEK9*
ORMDL2	*HEATR5B*	*NEMP1*
ORMDL3	*HEATR6*	*NENF*
OS9	*HEBP2*	*NEPRO*
OSBP2	*HECA*	*NET1*
OSBPL10	*HECTD1*	*NETO1*
OSBPL2	*HECTD2*	*NETO2*
OSBPL3	*HECW1*	*NEU3*
OSBPL6	*HECW2*	*NEURL1*
OSBPL7	*HEG1*	*NEURL1B*
OSBPL8	*HELB*	*NEURL4*
OSGIN2	*HELLS*	*NEUROD2*
OSMR	*HELQ*	*NEUROD4*
OSTM1	*HELZ*	*NEUROG2*
OTUB2	*HEMK1*	*NEXMIF*
OTUD4	*HENMT1*	*NFAT5*
OTULINL	*HEPACAM*	*NFATC2*
OTX2	*HEPH*	*NFATC3*
OXA1L	*HEPHL1*	*NFATC4*
OXCT1	*HERC2*	*NFIB*
OXNAD1	*HERC3*	*NFIC*
OXR1	*HERC4*	*NFKBID*
OXSR1	*HERPUD2*	*NFS1*
OXTR	*HES2*	*NFX1*
P2RX4	*HEXIM1*	*NFYA*
P2RY1	*HEXIM2*	*NFYB*

P2RY12	*HEY2*	*NFYC*
P2RY2	*HEYL*	*NGB*
P4HA2	*HFE*	*NGRN*
PABPC3	*HFM1*	*NHLRC2*
PABPN1	*HGF*	*NHLRC3*
PACRGL	*HGFAC*	*NHLRC4*
PACS1	*HGS*	*NHS*
PACS2	*HGSNAT*	*NHSL1*
PADI1	*HHEX*	*NIBAN1*
PADI2	*HHIP*	*NIBAN3*
PAF1	*HHIPL1*	*NIFK*
PAFAH1B2	*HIBADH*	*NIN*
PAG1	*HIF1A*	*NINJ1*
PAGR1	*HIF3A*	*NIPA1*
PAH	*HIGD1A*	*NIPA2*
PAICS	*HIKESHI*	*NIPAL2*
PAIP1	*HINT1*	*NIPAL4*
PAIP2	*HINT3*	*NIPBL*
PAIP2B	*HIP1*	*NISCH*
PAK1	*HIPK1*	*NIT1*
PAK1IP1	*HIPK2*	*NIT2*
PAK2	*HIPK3*	*NKAIN1*
PAK3	*HIPK4*	*NKAIN3*
PALLD	*HIST1H2AE*	*NKAP*
PAN2	*HIST1H2AG*	*NKAPL*
PANK1	*HIST1H2AH*	*NKD1*
PANK3	*HIST1H2BD*	*NKIRAS1*
PANK4	*HIST1H2BG*	*NKRF*
PAOX	*HIST1H4C*	*NKTR*
PAPLN	*HIST2H2BE*	*NKX2-1*
PAPOLA	*HIVEP3*	*NKX2-4*
PAPOLG	*HJURP*	*NKX3-2*
PAPSS1	*HJV*	*NLE1*
PAQR3	*HK2*	*NLGN4X*
PARD3B	*HLA-DOA*	*NLK*
PARD6G-AS1	*HLA-DOB*	*NLN*
PARK7	*HLA-DPA1*	*NLRP1*
PARL	*HLA-DPB1*	*NLRP3*
PARP14	*HLA-DQA1*	*NLRP9*
PARP15	*HLA-DQA2*	*NME2*
PARP4	*HLA-DRB1*	*NME6*
PARP9	*HLA-DRB5*	*NMNAT1*
PARVA	*HLA-E*	*NMNAT2*
PARVB	*HLF*	*NMT2*

PARVG	*HLTF*	*NNT*
PATL1	*HM13*	*NOCT*
PATZ1	*HMBOX1*	*NOD1*
PAWR	*HMG20A*	*NOL11*
PAX1	*HMGA2*	*NOL12*
PAX3	*HMGB1*	*NOL3*
PAX5	*HMGB3*	*NOL4L*
PAX7	*HMGCLL1*	*NOL9*
PAX8	*HMGCR*	*NOM1*
PAXBP1	*HMGCS1*	*NOMO1*
PBRM1	*HMGN1*	*NOMO3*
PBX1	*HMGN2*	*NOP58*
PBX2	*HMGN4*	*NOP9*
PBX3	*HMGN5*	*NOS1*
PBXIP1	*HMGXB4*	*NOS1AP*
PCARE	*HMMR*	*NOTCH2*
PCBP2	*HMSD*	*NOTCH3*
PCDH10	*HMX3*	*NOVA1*
PCDH11X	*HNF4A*	*NOX1*
PCDH15	*HNMT*	*NOX4*
PCDH19	*HNRNPA0*	*NPAP1*
PCDH20	*HNRNPA1*	*NPAS4*
PCDH7	*HNRNPA2B1*	*NPBWR1*
PCDH9	*HNRNPA3*	*NPC2*
PCDHA1	*HNRNPC*	*NPFFR1*
PCDHA10	*HNRNPD*	*NPL*
PCDHA11	*HNRNPDL*	*NPLOC4*
PCDHA12	*HNRNPF*	*NPM1*
PCDHA13	*HNRNPK*	*NPNT*
PCDHA2	*HNRNPL*	*NPPC*
PCDHA3	*HNRNPLL*	*NPR2*
PCDHA4	*HNRNPR*	*NPR3*
PCDHA5	*HNRNPU*	*NPTX1*
PCDHA6	*HNRNPUL1*	*NPTXR*
PCDHA7	*HNRNPUL2*	*NQO1*
PCDHA8	*HOMER1*	*NR0B1*
PCDHA9	*HOMEZ*	*NR1D2*
PCDHAC1	*HOOK1*	*NR1I2*
PCDHAC2	*HOOK3*	*NR2C1*
PCDHGA1	*HOPX*	*NR2C2*
PCDHGA10	*HOXA1*	*NR2C2AP*
PCDHGA11	*HOXA13*	*NR2E1*
PCDHGA12	*HOXA2*	*NR2F6*
PCDHGA2	*HOXB2*	*NR3C1*

PCDHGA3	*HOXB3*	*NR4A2*
PCDHGA4	*HOXC10*	*NR5A2*
PCDHGA5	*HOXC11*	*NR6A1*
PCDHGA6	*HOXC4*	*NRARP*
PCDHGA7	*HOXC6*	*NRF1*
PCDHGA8	*HOXC8*	*NRG1*
PCDHGA9	*HOXD1*	*NRG2*
PCDHGB1	*HOXD11*	*NRG3*
PCDHGB2	*HOXD12*	*NRG4*
PCDHGB3	*HOXD3*	*NRK*
PCDHGB4	*HOXD4*	*NRP1*
PCDHGB6	*HOXD8*	*NRXN1*
PCDHGB7	*HOXD9*	*NRXN3*
PCDHGC3	*HP1BP3*	*NSD2*
PCDHGC4	*HPCA*	*NSL1*
PCDHGC5	*HPCAL4*	*NSMCE3*
PCGF3	*HPDL*	*NSMCE4A*
PCLAF	*HPF1*	*NSUN4*
PCNT	*HPGD*	*NSUN5*
PCNX1	*HPR*	*NSUN7*
PCSK1	*HPRT1*	*NT5C1A*
PCSK9	*HPS3*	*NT5DC1*
PCYOX1	*HPS4*	*NT5DC2*
PCYT1A	*HPS5*	*NT5M*
PCYT1B	*HPSE*	*NTF3*
PCYT2	*HPX*	*NTM*
PDAP1	*HRH4*	*NTPCR*
PDCD10	*HRK*	*NTRK2*
PDCD2	*HRNR*	*NTSR1*
PDCD6IP	*HS2ST1*	*NUAK1*
PDCD7	*HS3ST1*	*NUBPL*
PDCL	*HS3ST3A1*	*NUCKS1*
PDE10A	*HS3ST3B1*	*NUDCD2*
PDE11A	*HS6ST2*	*NUDCD3*
PDE1C	*HS6ST3*	*NUDT16*
PDE3A	*HSBP1*	*NUDT21*
PDE4A	*HSD11B1*	*NUDT3*
PDE4B	*HSD17B11*	*NUF2*
PDE4D	*HSD17B12*	*NUFIP1*
PDE4DIP	*HSD17B13*	*NUFIP2*
PDE5A	*HSD17B6*	*NUP155*
PDE6B	*HSDL1*	*NUP160*
PDE7A	*HSDL2*	*NUP205*
PDE7B	*HSF5*	*NUP210*

PDGFA	*HSFY2*	*NUP35*
PDGFD	*HSP90B1*	*NUP43*
PDGFRB	*HSPA12A*	*NUP58*
PDHA1	*HSPA13*	*NUP62CL*
PDIA6	*HSPA14*	*NUP88*
PDIK1L	*HSPA1B*	*NUP93*
PDK1	*HSPA1L*	*NUS1*
PDLIM2	*HSPA4*	*NUTF2*
PDLIM5	*HSPA4L*	*NUTM2A*
PDP1	*HSPA5*	*NUTM2D*
PDP2	*HSPA6*	*NUTM2E*
PDPK1	*HSPB3*	*NUTM2G*
PDS5A	*HSPB7*	*NWD1*
PDS5B	*HSPB8*	*NWD2*
PDSS2	*HSPBAP1*	*NXF1*
PDXDC1	*HSPD1*	*NXN*
PEBP1	*HSPE1-MOB4*	*NXPH1*
PEG10	*HTATIP2*	*NXT2*
PELI1	*HTR1D*	*NYAP1*
PELI2	*HTR1E*	*NYAP2*
PER1	*HTR1F*	*OAS2*
PER2	*HTR2A*	*OAS3*
PERP	*HTR2B*	*OCA2*
PES1	*HTR2C*	*OCIAD2*
PEX1	*HTR3B*	*OCRL*
PEX11A	*HTR3E*	*ODAPH*
PEX11B	*HTR5A*	*ODC1*
PEX13	*HTR5A-AS1*	*ODF2L*
PEX2	*HTRA1*	*OGDH*
PEX26	*HTT*	*OGFOD1*
PEX3	*HUNK*	*OGFOD3*
PEX5L	*HUS1*	*OGFRL1*
PFDN2	*HUWE1*	*OIP5*
PFDN6	*HVCN1*	*OIT3*
PFKFB2	*HYAL4*	*OLA1*
PFKFB4	*HYKK*	*OLFM1*
PFN1	*HYLS1*	*OLFM4*
PFN2	*IAH1*	*OLIG2*
PGAM1	*IAPP*	*ONECUT1*
PGAM4	*IARS2*	*ONECUT2*
PGAP1	*IBSP*	*OPA3*
PGBD1	*IBTK*	*OPALIN*
PGBD3	*ICA1L*	*OPCML*
PGBD4	*ICAM1*	*OPHN1*

PGGHG	*ICE1*	*OPN1MW2*
PGGT1B	*ICE2*	*OPRK1*
PGLYRP1	*ICK*	*OPRM1*
PGLYRP4	*ICMT*	*OR10H5*
PGM2	*ICOS*	*OR13C3*
PGM2L1	*ICOSLG*	*OR1L8*
PGPEP1	*ID3*	*OR2A4*
PGPEP1L	*ID4*	*OR2B11*
PGR	*IDE*	*OR51E1*
PHACTR2	*IDH1*	*OR7A5*
PHACTR4	*IDH3A*	*OR8J1*
PHAX	*IDI1*	*ORAI2*
PHC1	*IDI2*	*ORAI3*
PHC2	*IDS*	*ORM1*
PHC3	*IER3IP1*	*ORMDL2*
PHETA2	*IER5*	*ORMDL3*
PHEX	*IER5L*	*OSBP2*
PHF12	*IFI44L*	*OSBPL10*
PHF19	*IFIT1*	*OSBPL2*
PHF20	*IFIT3*	*OSBPL3*
PHF8	*IFIT5*	*OSBPL6*
PHIP	*IFNA1*	*OSBPL7*
PHKA2	*IFNA10*	*OSBPL8*
PHKG2	*IFNA14*	*OSGIN2*
PHLDB2	*IFNA16*	*OSMR*
PHRF1	*IFNA17*	*OSTM1*
PHYHIP	*IFNA2*	*OTOGL*
PI15	*IFNA21*	*OTUB2*
PI16	*IFNA4*	*OTUD3*
PI4K2A	*IFNA6*	*OTUD4*
PID1	*IFNA7*	*OTUD6B*
PIGF	*IFNAR1*	*OTULINL*
PIGH	*IFNG*	*OXA1L*
PIGK	*IFNGR1*	*OXCT1*
PIGL	*IFNK*	*OXNAD1*
PIGO	*IFRD1*	*OXR1*
PIGP	*IFT122*	*OXSR1*
PIGQ	*IFT22*	*OXTR*
PIGT	*IFT57*	*P2RX4*
PIH1D3	*IFT74*	*P2RY1*
PIK3AP1	*IFT80*	*P2RY12*
PIK3C2A	*IFT81*	*P2RY14*
PIK3C2B	*IFT88*	*P2RY2*
PIK3CA	*IGDCC3*	*P4HA2*

PIK3CG	*IGDCC4*	*PABPC1*
PIK3R1	*IGF1*	*PABPC3*
PIN4	*IGF1R*	*PABPN1*
PIP4P1	*IGF2BP1*	*PACRGL*
PIP4P2	*IGF2BP3*	*PACS1*
PIP5K1A	*IGF2R*	*PACS2*
PIRT	*IGFBP4*	*PADI1*
PITPNA	*IGFBP5*	*PADI2*
PITPNB	*IGSF10*	*PAF1*
PITPNC1	*IGSF11*	*PAFAH1B2*
PITPNM2	*IGSF3*	*PAG1*
PIWIL1	*IGSF6*	*PAGR1*
PIWIL2	*IKBIP*	*PAH*
PKDCC	*IKZF1*	*PAICS*
PKDREJ	*IKZF3*	*PAIP1*
PKHD1	*IKZF5*	*PAIP2*
PKHD1L1	*IL10RA*	*PAIP2B*
PKIA	*IL11*	*PAK1*
PKLR	*IL12RB2*	*PAK1IP1*
PKM	*IL15RA*	*PAK2*
PKNOX2	*IL16*	*PAK3*
PKP1	*IL17A*	*PALB2*
PKP2	*IL17RA*	*PALLD*
PKP4	*IL17RB*	*PALM2-AKAP2*
PLA2G12A	*IL17RD*	*PANK1*
PLA2G2C	*IL17REL*	*PANK3*
PLA2G4D	*IL18R1*	*PANK4*
PLA2G7	*IL1A*	*PAOX*
PLAAT3	*IL1F10*	*PAPLN*
PLAAT5	*IL1R1*	*PAPOLA*
PLAC8	*IL1RAP*	*PAPOLG*
PLAG1	*IL1RAPL1*	*PAPPA2*
PLAGL1	*IL1RL1*	*PAPSS1*
PLAUR	*IL1RN*	*PAPSS2*
PLCB1	*IL22RA1*	*PAQR3*
PLCB2	*IL22RA2*	*PARD3*
PLCE1	*IL24*	*PARD3B*
PLCG1	*IL2RB*	*PARD6G-AS1*
PLCH2	*IL3*	*PARL*
PLCL1	*IL31RA*	*PARP14*
PLCXD1	*IL33*	*PARP15*
PLCXD3	*IL3RA*	*PARP4*
PLD5	*IL5*	*PARP9*
PLEKHA3	*IL5RA*	*PARVA*

PLEKHA5	*IL6*	*PARVB*
PLEKHA6	*IL6ST*	*PARVG*
PLEKHA8	*IL7*	*PATE2*
PLEKHB1	*ILDR2*	*PATL1*
PLEKHB2	*ILF2*	*PATZ1*
PLEKHF1	*ILF3*	*PAWR*
PLEKHG4B	*ILVBL*	*PAX1*
PLEKHH2	*IMMP2L*	*PAX3*
PLEKHM1	*IMP4*	*PAX5*
PLEKHM2	*IMPA1*	*PAX6*
PLEKHM3	*IMPA2*	*PAX7*
PLEKHO1	*IMPACT*	*PAX8*
PLIN3	*IMPAD1*	*PAXBP1*
PLPP3	*IMPG1*	*PBRM1*
PLPP4	*IMPG2*	*PBX1*
PLPPR1	*INA*	*PBX3*
PLRG1	*INCENP*	*PBXIP1*
PLS1	*INF2*	*PCARE*
PLSCR1	*ING1*	*PCDH10*
PLSCR4	*ING2*	*PCDH11X*
PLXDC1	*ING3*	*PCDH15*
PLXDC2	*ING4*	*PCDH19*
PLXNA2	*INHBA*	*PCDH20*
PLXNA3	*INHBB*	*PCDH7*
PLXNB2	*INIP*	*PCDH9*
PLXNC1	*INKA2*	*PCDHA1*
PLXND1	*INMT*	*PCDHA10*
PM20D2	*INO80D*	*PCDHA11*
PMEPA1	*INPP1*	*PCDHA12*
PML	*INPP4A*	*PCDHA13*
PNKD	*INPP4B*	*PCDHA2*
PNMA8B	*INSC*	*PCDHA3*
PNN	*INSIG1*	*PCDHA4*
PNO1	*INSIG2*	*PCDHA5*
PNP	*INSL5*	*PCDHA6*
PNPLA3	*INSM1*	*PCDHA7*
PNPLA4	*INSM2*	*PCDHA8*
PNPLA8	*INSR*	*PCDHA9*
PNRC2	*INSYN2A*	*PCDHAC1*
POC1A	*INSYN2B*	*PCDHAC2*
POC1B	*INTS12*	*PCDHB5*
POC5	*INTS13*	*PCDHGA1*
POF1B	*INTS14*	*PCDHGA10*
POFUT1	*INTS2*	*PCDHGA11*

POGZ	*INTS3*	*PCDHGA12*
POLA2	*INTS6*	*PCDHGA2*
POLD2	*INTS7*	*PCDHGA3*
POLD4	*INTS8*	*PCDHGA4*
POLDIP3	*INTU*	*PCDHGA5*
POLE	*INVS*	*PCDHGA6*
POLH	*IP6K1*	*PCDHGA7*
POLR1A	*IPCEF1*	*PCDHGA8*
POLR1D	*IPMK*	*PCDHGA9*
POLR1E	*IPO11-LRRC70*	*PCDHGB1*
POLR2G	*IPO5*	*PCDHGB2*
POLR2H	*IPO7*	*PCDHGB3*
POLR3E	*IPO8*	*PCDHGB4*
POLR3F	*IPO9*	*PCDHGB6*
POLR3H	*IPP*	*PCDHGB7*
POM121	*IPPK*	*PCDHGC3*
POMGNT1	*IQCB1*	*PCDHGC4*
POMGNT2	*IQCE*	*PCDHGC5*
POMT2	*IQCG*	*PCF11*
POP1	*IQCH*	*PCGF3*
POP4	*IQCJ*	*PCLAF*
POPDC2	*IQGAP1*	*PCNX1*
POT1	*IQGAP3*	*PCSK1*
POU2F1	*IQSEC2*	*PCSK7*
POU3F2	*IRAK1BP1*	*PCSK9*
POU3F3	*IRAK2*	*PCYOX1*
POU4F2	*IRAK3*	*PCYT1B*
PPAN-P2RY11	*IRAK4*	*PCYT2*
PPARGC1A	*IREB2*	*PDAP1*
PPARGC1B	*IRF1*	*PDCD10*
PPCDC	*IRF2BP2*	*PDCD2*
PPDPF	*IRF2BPL*	*PDCD6IP*
PPDPFL	*IRF4*	*PDCD7*
PPFIA1	*IRF8*	*PDCL*
PPFIBP1	*IRGQ*	*PDE10A*
PPFIBP2	*IRS1*	*PDE11A*
PPIA	*IRS2*	*PDE1C*
PPIAL4D	*IRS4*	*PDE3A*
PPIAL4G	*IRX1*	*PDE4A*
PPIE	*IRX4*	*PDE4B*
PPIG	*ISCA1*	*PDE4D*
PPIL1	*ISCA2*	*PDE4DIP*
PPIL2	*ISG20L2*	*PDE5A*
PPIL6	*ISL1*	*PDE6B*

PPIP5K2	*ISL2*	*PDE7A*
PPM1B	*ISLR2*	*PDE7B*
PPM1E	*ISM1*	*PDF*
PPM1F	*IST1*	*PDGFA*
PPM1H	*ISY1-RAB43*	*PDGFD*
PPM1L	*ITCH*	*PDGFRB*
PPME1	*ITGA1*	*PDHA1*
PPP1R12A	*ITGA11*	*PDIA6*
PPP1R12B	*ITGA2*	*PDIK1L*
PPP1R13B	*ITGA4*	*PDK1*
PPP1R14C	*ITGA8*	*PDLIM2*
PPP1R15A	*ITGA9*	*PDLIM5*
PPP1R15B	*ITGAL*	*PDP1*
PPP1R1A	*ITGAV*	*PDP2*
PPP1R26	*ITGB1BP1*	*PDPK1*
PPP1R3D	*ITGB3BP*	*PDPR*
PPP1R3G	*ITGB6*	*PDS5A*
PPP1R7	*ITGB8*	*PDS5B*
PPP1R8	*ITGBL1*	*PDXDC1*
PPP1R9A	*ITIH2*	*PDZD2*
PPP2R1A	*ITK*	*PEG10*
PPP2R1B	*ITM2B*	*PELI1*
PPP2R2A	*ITPA*	*PELI2*
PPP2R5E	*ITPK1*	*PELO*
PPP3CB	*ITPKB*	*PER1*
PPP3CC	*ITPR1*	*PER2*
PPP3R1	*ITPR2*	*PERP*
PPP3R2	*ITPRID1*	*PES1*
PPP4R2	*ITPRIP*	*PEX1*
PPP5C	*ITPRIPL2*	*PEX11A*
PPP5D1	*ITSN1*	*PEX11B*
PPP6C	*IVNS1ABP*	*PEX13*
PPRC1	*IWS1*	*PEX19*
PPTC7	*IYD*	*PEX2*
PQLC1	*JADE1*	*PEX26*
PQLC2L	*JADE2*	*PEX3*
PQLC3	*JAGN1*	*PEX5L*
PRAC1	*JAK1*	*PFAS*
PRC1	*JAK2*	*PFDN2*
PRDM1	*JAKMIP1*	*PFDN6*
PRDM10	*JAKMIP2*	*PFKFB2*
PRDM16	*JAKMIP3*	*PFKFB3*
PRDM2	*JAM2*	*PFKFB4*
PRDM5	*JAM3*	*PFN2*

PRDM6	*JAML*	*PGAM1*
PRDM7	*JARID2*	*PGAM4*
PRDM9	*JAZF1*	*PGAP1*
PRDX4	*JCAD*	*PGAP3*
PRDX6	*JDP2*	*PGBD1*
PRELID2	*JHY*	*PGBD3*
PRELID3B	*JMJD6*	*PGBD4*
PRELP	*JMY*	*PGGHG*
PREX2	*JOSD1*	*PGGT1B*
PRICKLE2	*JPH1*	*PGK1*
PRICKLE4	*JPH4*	*PGLYRP1*
PRKAA2	*JTB*	*PGLYRP4*
PRKAB2	*JUN*	*PGM2*
PRKACB	*KALRN*	*PGM2L1*
PRKAR1A	*KANK2*	*PGM3*
PRKAR2A	*KANK3*	*PGPEP1*
PRKCB	*KANSL3*	*PGPEP1L*
PRKCH	*KARS*	*PGR*
PRKCQ	*KAT14*	*PHACTR2*
PRKD1	*KAT2B*	*PHACTR4*
PRKG1	*KAT6A*	*PHAX*
PRKN	*KAT6B*	*PHC1*
PRKRA	*KAT7*	*PHC2*
PRKX	*KAT8*	*PHC3*
PRMT2	*KATNAL1*	*PHETA2*
PRMT3	*KATNAL2*	*PHEX*
PRMT6	*KATNBL1*	*PHF12*
PRMT9	*KAZALD1*	*PHF13*
PROB1	*KBTBD11*	*PHF19*
PROM1	*KBTBD12*	*PHF20*
PROM2	*KBTBD3*	*PHF21A*
PROSER3	*KBTBD6*	*PHF7*
PROX1	*KBTBD7*	*PHF8*
PRPF38A	*KCMF1*	*PHIP*
PRPF39	*KCNA1*	*PHKA2*
PRPF4B	*KCNA2*	*PHKG2*
PRPS1	*KCNA3*	*PHLDB2*
PRPS1L1	*KCNAB1*	*PHTF2*
PRR14L	*KCNB1*	*PHYHIP*
PRR15	*KCNC1*	*PI15*
PRR29	*KCNC2*	*PI16*
PRR3	*KCND2*	*PI4K2A*
PRR5L	*KCNE1*	*PIGF*
PRRC1	*KCNE2*	*PIGK*

PRRC2A	*KCNE4*	*PIGL*
PRRG1	*KCNG3*	*PIGO*
PRRG4	*KCNG4*	*PIGP*
PRRT1	*KCNH1*	*PIGT*
PRRX1	*KCNH5*	*PIK3C2B*
PRSS12	*KCNH6*	*PIK3CA*
PRSS23	*KCNH8*	*PIK3CG*
PRSS36	*KCNIP2*	*PIK3R1*
PRSS37	*KCNIP3*	*PIK3R3*
PRSS8	*KCNIP4*	*PIMREG*
PRTG	*KCNJ1*	*PIN1*
PRUNE1	*KCNJ13*	*PIN4*
PRXL2A	*KCNJ15*	*PIP4P1*
PSAP	*KCNJ2*	*PIP4P2*
PSD2	*KCNJ3*	*PIRT*
PSD3	*KCNJ5*	*PITHD1*
PSD4	*KCNJ6*	*PITPNA*
PSEN1	*KCNJ8*	*PITPNB*
PSIP1	*KCNJ9*	*PITPNC1*
PSMA4	*KCNK1*	*PITPNM1*
PSMB1	*KCNK10*	*PITPNM2*
PSMB11	*KCNK12*	*PIWIL1*
PSMB5	*KCNK13*	*PKD2*
PSMB7	*KCNK15*	*PKDCC*
PSMC4	*KCNK5*	*PKDREJ*
PSMD11	*KCNK6*	*PKHD1*
PSME3	*KCNK9*	*PKHD1L1*
PSMF1	*KCNMA1*	*PKIA*
PSPH	*KCNMB1*	*PKLR*
PTAFR	*KCNMB2*	*PKM*
PTAR1	*KCNMB3*	*PKNOX1*
PTBP2	*KCNMB4*	*PKNOX2*
PTBP3	*KCNN2*	*PKP1*
PTCD2	*KCNN3*	*PKP4*
PTCD3	*KCNQ3*	*PLA2G12A*
PTCHD1	*KCNQ5*	*PLA2G2C*
PTCHD4	*KCNS2*	*PLA2G4A*
PTDSS1	*KCNT2*	*PLA2G4D*
PTDSS2	*KCNV1*	*PLA2G7*
PTEN	*KCTD10*	*PLA2R1*
PTGER2	*KCTD12*	*PLAAT3*
PTGFR	*KCTD14*	*PLAAT5*
PTGIS	*KCTD15*	*PLAC8*
PTGS1	*KCTD16*	*PLAG1*

PTH2R	*KCTD3*	*PLAUR*
PTHLH	*KCTD7*	*PLCB1*
PTK2	*KCTD8*	*PLCE1*
PTPA	*KCTD9*	*PLCG1*
PTPMT1	*KDELR1*	*PLCH2*
PTPN1	*KDELR3*	*PLCL1*
PTPN11	*KDF1*	*PLCXD1*
PTPN14	*KDM2A*	*PLCXD3*
PTPN18	*KDM2B*	*PLD5*
PTPN3	*KDM3B*	*PLEKHA3*
PTPN4	*KDM4C*	*PLEKHA5*
PTPRJ	*KDM5A*	*PLEKHA6*
PTPRK	*KDM5B*	*PLEKHA8*
PTPRM	*KDM5C*	*PLEKHB1*
PTPRN	*KDM5D*	*PLEKHB2*
PTPRO	*KDM6A*	*PLEKHF1*
PTPRR	*KDM7A*	*PLEKHG4B*
PTPRT	*KERA*	*PLEKHH2*
PTPRZ1	*KHDC4*	*PLEKHM2*
PTRH2	*KHDRBS1*	*PLEKHM3*
PUM3	*KIAA0040*	*PLEKHO1*
PURA	*KIAA0087*	*PLEKHS1*
PURB	*KIAA0232*	*PLIN3*
PURG	*KIAA0319*	*PLOD2*
PUS7	*KIAA0355*	*PLP2*
PVR	*KIAA0408*	*PLPP3*
PWP1	*KIAA0513*	*PLPP4*
PWP2	*KIAA0586*	*PLPPR1*
PWWP2A	*KIAA0753*	*PLRG1*
PXDN	*KIAA0825*	*PLS1*
PXK	*KIAA0895*	*PLSCR1*
PXMP4	*KIAA0930*	*PLSCR4*
PXN	*KIAA1143*	*PLXDC1*
PXYLP1	*KIAA1147*	*PLXDC2*
PYCR1	*KIAA1211*	*PLXNA2*
PYGB	*KIAA1217*	*PLXNA3*
PYGO1	*KIAA1257*	*PLXNB2*
PYROXD1	*KIAA1324*	*PLXNC1*
QKI	*KIAA1324L*	*PLXND1*
QPRT	*KIAA1328*	*PM20D2*
QSER1	*KIAA1549*	*PMEPA1*
QSOX1	*KIAA1549L*	*PML*
QSOX2	*KIAA1586*	*PMPCA*
R3HCC1	*KIAA1614*	*PNISR*

RAB11B	*KIAA1841*	*PNKD*
RAB11FIP1	*KIAA1958*	*PNN*
RAB11FIP2	*KIAA2026*	*PNO1*
RAB11FIP5	*KIDINS220*	*PNP*
RAB14	*KIF11*	*PNPLA1*
RAB15	*KIF13A*	*PNPLA3*
RAB18	*KIF14*	*PNPLA4*
RAB1A	*KIF16B*	*PNPLA8*
RAB1B	*KIF18A*	*PNRC2*
RAB22A	*KIF1A*	*POC1A*
RAB23	*KIF1B*	*POF1B*
RAB27A	*KIF1BP*	*POFUT1*
RAB27B	*KIF1C*	*POFUT2*
RAB30	*KIF21A*	*POGLUT3*
RAB31	*KIF23*	*POGZ*
RAB32	*KIF27*	*POLA2*
RAB33B	*KIF2A*	*POLD3*
RAB36	*KIF3A*	*POLDIP3*
RAB38	*KIF3B*	*POLG*
RAB39B	*KIF3C*	*POLH*
RAB3A	*KIF4A*	*POLQ*
RAB3B	*KIF4B*	*POLR1A*
RAB3C	*KIF5A*	*POLR1D*
RAB3D	*KIF5B*	*POLR1E*
RAB3GAP1	*KIF5C*	*POLR2G*
RAB3GAP2	*KIFAP3*	*POLR2H*
RAB40B	*KIN*	*POLR3E*
RAB42	*KIR2DL1*	*POLR3F*
RAB43	*KIR2DL3*	*POLR3H*
RAB4A	*KIR2DL4*	*POM121*
RAB5B	*KIR3DL1*	*POMGNT2*
RAB7A	*KIR3DL2*	*POMP*
RAB9B	*KIR3DL3*	*POMT2*
RABGAP1	*KIRREL2*	*POP1*
RABIF	*KIT*	*POP4*
RABL2B	*KL*	*POPDC2*
RABL6	*KLC1*	*POT1*
RAD1	*KLC4*	*POU2F1*
RAD23B	*KLF10*	*POU3F2*
RAD50	*KLF12*	*POU3F3*
RAD51	*KLF13*	*POU4F2*
RAD51C	*KLF15*	*PP7080*
RAD52	*KLF17*	*PPAN-P2RY11*
RAD54L	*KLF2*	*PPARGC1A*

RAD9B	*KLF3*	*PPARGC1B*
RADX	*KLF5*	*PPDPFL*
RAG1	*KLF6*	*PPFIBP1*
RAI14	*KLF7*	*PPFIBP2*
RALA	*KLF8*	*PPIA*
RALGAPA1	*KLF9*	*PPIAL4D*
RALGPS1	*KLHDC1*	*PPIAL4G*
RALY	*KLHDC10*	*PPIE*
RAN	*KLHDC8A*	*PPIG*
RANBP10	*KLHDC9*	*PPIL1*
RANBP17	*KLHL1*	*PPIL6*
RANBP2	*KLHL11*	*PPIP5K2*
RANBP3L	*KLHL14*	*PPM1A*
RANBP6	*KLHL15*	*PPM1B*
RANGAP1	*KLHL18*	*PPM1E*
RAP1A	*KLHL20*	*PPM1F*
RAP1B	*KLHL21*	*PPM1H*
RAP1GAP2	*KLHL23*	*PPM1L*
RAP2A	*KLHL24*	*PPME1*
RAP2B	*KLHL28*	*PPP1R12A*
RAPGEF1	*KLHL3*	*PPP1R12B*
RAPGEF2	*KLHL30*	*PPP1R13B*
RAPGEF3	*KLHL31*	*PPP1R14C*
RAPGEF5	*KLHL36*	*PPP1R15A*
RAPGEF6	*KLHL38*	*PPP1R15B*
RAPH1	*KLHL4*	*PPP1R1A*
RASA2	*KLHL41*	*PPP1R26*
RASA4	*KLHL42*	*PPP1R3D*
RASA4B	*KLHL5*	*PPP1R3G*
RASEF	*KLHL6*	*PPP1R7*
RASGRF2	*KLHL7*	*PPP1R8*
RASGRP1	*KLHL8*	*PPP1R9A*
RASSF1	*KLHL9*	*PPP2R1A*
RASSF2	*KLK13*	*PPP2R1B*
RASSF3	*KLK3*	*PPP2R2A*
RASSF6	*KLLN*	*PPP2R2B*
RASSF8	*KLRB1*	*PPP2R5C*
RASSF9	*KLRC1*	*PPP2R5D*
RB1	*KLRC2*	*PPP2R5E*
RBAK	*KLRC3*	*PPP3CB*
RBBP7	*KLRC4*	*PPP3CC*
RBCK1	*KLRD1*	*PPP3R1*
RBFOX1	*KLRG1*	*PPP3R2*
RBFOX2	*KLRG2*	*PPP4R2*

RBM10	*KLRK1*	*PPP5C*
RBM14	*KMO*	*PPP5D1*
RBM14-RBM4	*KMT2A*	*PPP6C*
RBM15B	*KMT2C*	*PPTC7*
RBM18	*KMT5B*	*PPWD1*
RBM24	*KNG1*	*PQLC1*
RBM27	*KNL1*	*PQLC2L*
RBM28	*KNSTRN*	*PQLC3*
RBM4	*KNTC1*	*PRAC1*
RBM45	*KPNA1*	*PRC1*
RBM48	*KPNA3*	*PRDM1*
RBM4B	*KPNA4*	*PRDM10*
RBM5	*KPNA5*	*PRDM16*
RBM7	*KPNA6*	*PRDM2*
RBMS1	*KPNB1*	*PRDM5*
RBMS2	*KRAS*	*PRDM6*
RBMX2	*KRBOX4*	*PRDM7*
RBMXL1	*KREMEN1*	*PRDM9*
RBP2	*KRIT1*	*PRDX2*
RBPMS	*KRR1*	*PRDX6*
RBPMS2	*KRT1*	*PRELID2*
RBSN	*KRT10*	*PRELID3B*
RBX1	*KRT15*	*PREX2*
RCAN3	*KRT19*	*PRICKLE2*
RCC2	*KRT2*	*PRKAA2*
RCCD1	*KRT20*	*PRKAB2*
RCL1	*KRT222*	*PRKACB*
RCN2	*KRT24*	*PRKAG2*
RCOR1	*KRT26*	*PRKAR1A*
RCSD1	*KRT3*	*PRKAR2A*
RCVRN	*KRT35*	*PRKAR2B*
RD3	*KRT6A*	*PRKCA*
RD3L	*KRT6C*	*PRKCB*
RDH13	*KRT77*	*PRKCH*
RDX	*KRT82*	*PRKCQ*
RECK	*KRT85*	*PRKD1*
RECQL5	*KRTAP1-1*	*PRKG1*
REL	*KRTAP13-2*	*PRKN*
RELN	*KRTAP1-5*	*PRKRA*
RELT	*KRTAP17-1*	*PRKX*
RER1	*KRTAP19-3*	*PRMT2*
RERG	*KRTAP24-1*	*PRMT3*
RESF1	*KRTAP3-1*	*PRMT6*
RETREG1	*KRTAP4-11*	*PRMT9*

RETREG2	*KRTAP4-7*	*PROB1*
RETREG3	*KRTAP4-9*	*PROM2*
REV1	*KRTAP5-6*	*PROSER3*
REXO5	*KSR1*	*PROX1*
RFC1	*KSR2*	*PRPF38A*
RFC2	*KXD1*	*PRPF38B*
RFESD	*KYAT1*	*PRPF39*
RFK	*KYAT3*	*PRPF4B*
RFLNB	*KYNU*	*PRPS1*
RFPL4B	*L1TD1*	*PRPS1L1*
RFX2	*L2HGDH*	*PRR14L*
RFX3	*L3MBTL4*	*PRR15*
RFX4	*LACC1*	*PRR15L*
RFX5	*LACTB2-AS1*	*PRR29*
RGL1	*LALBA*	*PRR3*
RGL3	*LAMA1*	*PRR5L*
RGMB	*LAMA2*	*PRRC1*
RGP1	*LAMA4*	*PRRG1*
RGS10	*LAMB4*	*PRRG4*
RGS12	*LAMC1*	*PRRT1*
RGS18	*LAMC2*	*PRRX1*
RGS20	*LAMC3*	*PRSS12*
RGS4	*LAMP2*	*PRSS23*
RGS5	*LAMP3*	*PRSS36*
RGS6	*LAMTOR3*	*PRSS37*
RGS7	*LANCL2*	*PRSS8*
RGS9BP	*LANCL3*	*PRTG*
RHBDD1	*LAPTM4A*	*PRUNE1*
RHBDL3	*LAPTM5*	*PRUNE2*
RHEB	*LARP1B*	*PRXL2A*
RHOA	*LARP4*	*PSAP*
RHOBTB3	*LARP6*	*PSD3*
RHOC	*LARP7*	*PSD4*
RHOT1	*LARS*	*PSEN1*
RHOT2	*LARS2*	*PSG1*
RHOU	*LASP1*	*PSG4*
RIBC1	*LAT2*	*PSG8*
RIC1	*LATS1*	*PSIP1*
RIC8B	*LATS2*	*PSMA4*
RICTOR	*LAX1*	*PSMB1*
RILPL1	*LAYN*	*PSMB11*
RIMBP2	*LBH*	*PSMB5*
RIMKLA	*LBHD1*	*PSMB7*
RIMS2	*LBR*	*PSMC4*

RIMS3	*LBX1*	*PSMD11*
RIMS4	*LCA5*	*PSMD4*
RIPOR3	*LCE1A*	*PSME3*
RIPPLY1	*LCE1F*	*PSMF1*
RIPPLY3	*LCLAT1*	*PSPH*
RIT1	*LCOR*	*PTAFR*
RIT2	*LCORL*	*PTAR1*
RITA1	*LCP2*	*PTBP2*
RLIM	*LDAH*	*PTBP3*
RLN3	*LDB1*	*PTCD2*
RMI2	*LDB3*	*PTCD3*
RMND5A	*LDHA*	*PTCH1*
RNASE4	*LDLR*	*PTCHD1*
RNASEH2B	*LDLRAD1*	*PTCHD4*
RNF10	*LDLRAD2*	*PTDSS1*
RNF103-CHMP3	*LDLRAD3*	*PTEN*
RNF11	*LDLRAD4*	*PTGER2*
RNF130	*LDLRAP1*	*PTGFR*
RNF135	*LEF1*	*PTGS1*
RNF141	*LEKR1*	*PTH2R*
RNF144A	*LEMD3*	*PTHLH*
RNF144B	*LEP*	*PTK2*
RNF149	*LEPR*	*PTPA*
RNF150	*LEPROT*	*PTPMT1*
RNF152	*LEPROTL1*	*PTPN1*
RNF165	*LETM1*	*PTPN11*
RNF168	*LETM2*	*PTPN18*
RNF169	*LETMD1*	*PTPN3*
RNF180	*LGALSL*	*PTPN4*
RNF187	*LGI2*	*PTPRF*
RNF19B	*LGR4*	*PTPRJ*
RNF2	*LGR5*	*PTPRK*
RNF20	*LGSN*	*PTPRM*
RNF212B	*LHCGR*	*PTPRN*
RNF213	*LHPP*	*PTPRO*
RNF215	*LHX4*	*PTPRR*
RNF217	*LHX6*	*PTPRT*
RNF24	*LHX8*	*PTPRZ1*
RNF32	*LHX9*	*PTRH2*
RNF4	*LIFR*	*PUM3*
RNF41	*LIG3*	*PURA*
RNF43	*LILRA1*	*PURB*
RNF44	*LILRA6*	*PURG*
RNF7	*LILRB4*	*PUS7*

RNFT2	*LIMCH1*	*PVR*
RNGTT	*LIMD1*	*PWP1*
RNLS	*LIN28A*	*PWP2*
RNMT	*LIN28B*	*PWWP2A*
RNPEPL1	*LIN52*	*PXDN*
RNPS1	*LIN54*	*PXK*
RO60	*LIN7A*	*PXMP4*
ROBO2	*LIN7C*	*PXN*
ROMO1	*LIN9*	*PXYLP1*
ROPN1	*LINC00239*	*PYCR1*
RORA	*LINC00346*	*PYGB*
RORB	*LINC00598*	*PYGO1*
ROS1	*LINC01124*	*PYROXD1*
RPA3	*LINC01148*	*QKI*
RPAP2	*LINC01164*	*QPRT*
RPAP3	*LINC01465*	*QSER1*
RPE	*LINC01554*	*QSOX1*
RPH3A	*LINC01555*	*QSOX2*
RPL10	*LINC01559*	*R3HCC1*
RPL10A	*LINC01590*	*RAB11A*
RPL12	*LINC01619*	*RAB11B*
RPL13	*LINC01664*	*RAB11FIP2*
RPL15	*LINC01909*	*RAB11FIP3*
RPL17-C18orf32	*LINC02089*	*RAB11FIP5*
RPL24	*LINC02166*	*RAB14*
RPL27A	*LINC02312*	*RAB15*
RPL28	*LINC02453*	*RAB17*
RPL31	*LINGO2*	*RAB18*
RPL37	*LINGO4*	*RAB1A*
RPL37A	*LIPA*	*RAB1B*
RPL3L	*LIPC*	*RAB22A*
RPL7L1	*LIPG*	*RAB23*
RPL8	*LIPH*	*RAB27A*
RPP14	*LIPN*	*RAB27B*
RPP40	*LIPT2*	*RAB30*
RPRD1B	*LITAF*	*RAB31*
RPRD2	*LIX1*	*RAB32*
RPS14	*LL22NC03-63E9.3*	*RAB33B*
RPS15A	*LLPH*	*RAB36*
RPS17	*LMAN1*	*RAB37*
RPS2	*LMAN2L*	*RAB38*
RPS20	*LMBR1*	*RAB39B*
RPS23	*LMBRD2*	*RAB3A*
RPS3	*LMCD1*	*RAB3B*

RPS6KA1	*LMLN*	*RAB3C*
RPS6KA6	*LMNB2*	*RAB3D*
RPS6KB1	*LMNTD1*	*RAB3GAP1*
RPTOR	*LMO2*	*RAB3GAP2*
RPUSD2	*LMO3*	*RAB40B*
RRAGC	*LMO4*	*RAB42*
RREB1	*LMO7*	*RAB43*
RRM2B	*LMOD1*	*RAB4A*
RRP12	*LMOD2*	*RAB5A*
RRP36	*LMTK2*	*RAB5B*
RRP7A	*LMX1A*	*RAB5C*
RRP8	*LNP1*	*RAB6B*
RS1	*LNPEP*	*RAB7A*
RSBN1	*LNPK*	*RAB9B*
RSBN1L	*LNX1*	*RABGAP1*
RSC1A1	*LNX2*	*RABIF*
RSF1	*LOC100131496*	*RABL2B*
RSKR	*LOC100288208*	*RACGAP1*
RSL1D1	*LOC100996385*	*RAD1*
RSPH3	*LOC100996419*	*RAD18*
RSPO2	*LOC105375614*	*RAD23B*
RSRC2	*LOC107983990*	*RAD50*
RTCB	*LOC107986898*	*RAD51*
RTF1	*LOC400464*	*RAD51AP1*
RTKN2	*LOC401589*	*RAD51B*
RTL4	*LOC644656*	*RAD51C*
RTL6	*LOC730098*	*RAD52*
RTL8B	*LOC730338*	*RAD54L*
RTN1	*LOH12CR2*	*RAD9B*
RTN3	*LONP2*	*RADX*
RUBCN	*LONRF1*	*RAG1*
RUBCNL	*LONRF2*	*RAI14*
RUFY2	*LONRF3*	*RALA*
RUNDC1	*LOX*	*RALGAPA1*
RUNDC3A	*LPAR3*	*RALGPS1*
RUNX1T1	*LPAR4*	*RALY*
RUNX2	*LPCAT1*	*RAN*
RWDD1	*LPCAT2*	*RANBP10*
RWDD2A	*LPCAT3*	*RANBP17*
RWDD2B	*LPGAT1*	*RANBP2*
RXRB	*LPIN1*	*RANBP3L*
RYR2	*LPIN2*	*RANBP6*
S100A16	*LPO*	*RANGAP1*
S100B	*LPP*	*RAP1A*

S100PBP	*LPXN*	*RAP1B*
S1PR1	*LRAT*	*RAP2A*
S1PR3	*LRATD1*	*RAP2B*
SACM1L	*LRATD2*	*RAPGEF1*
SAFB	*LRCH1*	*RAPGEF2*
SAFB2	*LRCH2*	*RAPGEF3*
SALL4	*LRCH3*	*RAPGEF5*
SAMD1	*LRGUK*	*RAPGEF6*
SAMD10	*LRIF1*	*RAPH1*
SAMD12	*LRIG1*	*RASA2*
SAMD4A	*LRIG2*	*RASA4*
SAMD5	*LRIG3*	*RASA4B*
SAMD8	*LRP10*	*RASEF*
SAMHD1	*LRP11*	*RASGRF1*
SAP130	*LRP12*	*RASGRF2*
SAP30	*LRP1B*	*RASGRP1*
SAP30L	*LRP5L*	*RASSF1*
SAPCD2	*LRP6*	*RASSF2*
SAR1A	*LRP8*	*RASSF3*
SAR1B	*LRPAP1*	*RASSF6*
SARAF	*LRPPRC*	*RASSF8*
SARDH	*LRR1*	*RASSF9*
SARS	*LRRC1*	*RB1*
SART1	*LRRC14B*	*RBAK*
SATB1	*LRRC15*	*RBBP7*
SAXO2	*LRRC18*	*RBCK1*
SBF1	*LRRC19*	*RBFA*
SBK1	*LRRC2*	*RBFOX1*
SC5D	*LRRC28*	*RBFOX2*
SCAF8	*LRRC3*	*RBM14-RBM4*
SCAI	*LRRC31*	*RBM18*
SCARB2	*LRRC32*	*RBM24*
SCD	*LRRC34*	*RBM27*
SCD5	*LRRC37A3*	*RBM28*
SCG3	*LRRC3B*	*RBM33*
SCG5	*LRRC3C*	*RBM39*
SCHIP1	*LRRC40*	*RBM4*
SCLT1	*LRRC49*	*RBM45*
SCN11A	*LRRC4C*	*RBM48*
SCN1A	*LRRC57*	*RBM4B*
SCN8A	*LRRC58*	*RBM5*
SCNN1A	*LRRC59*	*RBM6*
SCO1	*LRRC7*	*RBM7*
SCRN1	*LRRC73*	*RBMS1*

SCRN3	*LRRC75A*	*RBMS2*
SCUBE3	*LRRC8B*	*RBMX2*
SDAD1	*LRRC8C*	*RBMXL1*
SDC1	*LRRCC1*	*RBP2*
SDC2	*LRRFIP1*	*RBPMS*
SDCBP	*LRRK2*	*RBPMS2*
SDHA	*LRRN1*	*RBSN*
SDHAF1	*LRRN3*	*RBX1*
SDHAF2	*LRRTM1*	*RCAN3*
SDK1	*LRRTM2*	*RCBTB2*
SEC16B	*LRRTM3*	*RCC2*
SEC22C	*LRRTM4*	*RCCD1*
SEC23IP	*LRTOMT*	*RCL1*
SEC24A	*LSAMP*	*RCN2*
SEC24B	*LSG1*	*RCOR1*
SEC24C	*LSM10*	*RCSD1*
SEC31B	*LSM11*	*RCVRN*
SEC62	*LSM14A*	*RD3*
SEC63	*LSM2*	*RD3L*
SECISBP2	*LSM3*	*RDH13*
SECISBP2L	*LSM5*	*RDX*
SEH1L	*LSM6*	*RECK*
SELENOF	*LSM8*	*RECQL5*
SELENOI	*LTA*	*REEP3*
SELENOS	*LTB4R*	*REL*
SELENOT	*LTBP2*	*RELN*
SELENOV	*LTBR*	*RELT*
SELP	*LTV1*	*RER1*
SEM1	*LUC7L3*	*RERG*
SEMA3A	*LUM*	*RESF1*
SEMA3C	*LURAP1*	*RETREG1*
SEMA4B	*LURAP1L*	*RETREG2*
SEMA4C	*LUZP1*	*RETREG3*
SEMA4F	*LUZP2*	*REV1*
SEMA4G	*LUZP6*	*REXO5*
SEMA5A	*LVRN*	*RFC2*
SEMA6A	*LXN*	*RFESD*
SEMA6D	*LY6D*	*RFK*
SENP2	*LY6G5B*	*RFLNB*
SENP7	*LY6K*	*RFPL4B*
SEPHS1	*LY75*	*RFX2*
SEPTIN11	*LY75-CD302*	*RFX3*
SEPTIN14	*LYN*	*RFX4*
SEPTIN2	*LYPD6*	*RFX5*

SEPTIN7	*LYPLA1*	*RGL1*
SEPTIN8	*LYPLAL1*	*RGL3*
SERAC1	*LYRM2*	*RGMB*
SERBP1	*LYRM4*	*RGP1*
SERHL2	*LYRM7*	*RGS10*
SERINC1	*LYSMD3*	*RGS12*
SERINC3	*LYVE1*	*RGS13*
SERPINB2	*LZIC*	*RGS18*
SERPINB5	*LZTFL1*	*RGS20*
SERPINB9	*LZTS1*	*RGS4*
SERPIND1	*M1AP*	*RGS5*
SERPINH1	*M6PR*	*RGS6*
SERTAD2	*MAATS1*	*RGS7*
SERTAD4	*MAB21L3*	*RGS9BP*
SERTM1	*MACC1*	*RHBDD1*
SESN2	*MACF1*	*RHBDL3*
SESN3	*MACO1*	*RHEB*
SESTD1	*MACROD2*	*RHOA*
SETD1A	*MAD2L1*	*RHOBTB3*
SETD1B	*MADD*	*RHOC*
SETD5	*MAF*	*RHOH*
SETD7	*MAFB*	*RHOT1*
SETD9	*MAFF*	*RHOT2*
SF3A1	*MAFG*	*RHOU*
SF3A3	*MAFK*	*RIBC1*
SF3B3	*MAG*	*RIC1*
SF3B4	*MAGEA11*	*RIC8B*
SF3B6	*MAGEB10*	*RICTOR*
SFPQ	*MAGEB18*	*RILPL1*
SFRP1	*MAGEC2*	*RIMBP2*
SFRP2	*MAGEL2*	*RIMKLA*
SFT2D2	*MAGI1*	*RIMKLB*
SFT2D3	*MAGI2*	*RIMS2*
SFXN1	*MAGI3*	*RIMS3*
SFXN2	*MAGOHB*	*RIMS4*
SFXN5	*MAK*	*RIN2*
SGCD	*MAK16*	*RIPOR3*
SGCG	*MALSU1*	*RIPPLY1*
SGO1	*MALT1*	*RIPPLY3*
SGPL1	*MAML1*	*RIT1*
SGTB	*MAML2*	*RIT2*
SH2B3	*MAML3*	*RLIM*
SH2D1B	*MAMLD1*	*RLN3*
SH2D4A	*MAN1A1*	*RMI2*

SH3BGRL	*MAN1A2*	*RMND5A*
SH3BGRL2	*MAN2A1*	*RNASE4*
SH3BP2	*MANBA*	*RNASEH2B*
SH3BP4	*MANBAL*	*RNF10*
SH3D19	*MANEA*	*RNF103-CHMP3*
SH3GLB1	*MANSC1*	*RNF11*
SH3KBP1	*MAOA*	*RNF130*
SH3PXD2B	*MAP10*	*RNF135*
SH3RF2	*MAP1B*	*RNF141*
SH3RF3	*MAP2*	*RNF144A*
SHB	*MAP2K1*	*RNF144B*
SHC3	*MAP2K4*	*RNF149*
SHCBP1	*MAP2K6*	*RNF150*
SHE	*MAP3K1*	*RNF152*
SHISA2	*MAP3K13*	*RNF157*
SHISA6	*MAP3K19*	*RNF165*
SHISA7	*MAP3K20*	*RNF168*
SHISA9	*MAP3K21*	*RNF169*
SHISAL1	*MAP3K5*	*RNF180*
SHLD2	*MAP3K7*	*RNF182*
SHMT1	*MAP3K7CL*	*RNF185*
SHOC2	*MAP3K8*	*RNF187*
SHPRH	*MAP3K9*	*RNF19B*
SHQ1	*MAP4K3*	*RNF2*
SHROOM1	*MAP6*	*RNF20*
SHROOM2	*MAP6D1*	*RNF207*
SHROOM3	*MAP7*	*RNF212*
SHROOM4	*MAP9*	*RNF212B*
SHTN1	*MAPK1*	*RNF213*
SI	*MAPK10*	*RNF215*
SIAE	*MAPK13*	*RNF217*
SIAH3	*MAPK14*	*RNF24*
SIGIRR	*MAPK1IP1L*	*RNF32*
SIGLEC1	*MAPK6*	*RNF4*
SIGLEC11	*MAPK8*	*RNF41*
SIGLEC14	*MAPK9*	*RNF43*
SIGLEC6	*MAPKAP1*	*RNF44*
SIGLEC9	*MAPKAPK3*	*RNF6*
SIKE1	*MAPKAPK5*	*RNF7*
SIM1	*MAPRE1*	*RNFT2*
SIM2	*MAPRE2*	*RNGTT*
SIN3B	*MAPT*	*RNLS*
SIPA1	*MARC1*	*RNMT*
SIRPA	*MARCH1*	*RNPEPL1*

SIRPB2	*MARCH3*	*RNPS1*
SIRT1	*MARCH4*	*RO60*
SIX1	*MARCH6*	*ROBO2*
SIX6	*MARCH7*	*ROCK1*
SKA2	*MARCH8*	*ROMO1*
SKA3	*MARCH9*	*ROPN1*
SKAP2	*MARF1*	*RORA*
SKIL	*MARK4*	*RORB*
SKIV2L	*MARS2*	*ROS1*
SKP1	*MASP1*	*RPA4*
SLAIN2	*MASP2*	*RPAP2*
SLAMF1	*MAST3*	*RPAP3*
SLAMF6	*MASTL*	*RPE*
SLAMF7	*MAT2B*	*RPH3A*
SLAMF8	*MATN1*	*RPL10*
SLBP	*MATN2*	*RPL10A*
SLC10A2	*MAVS*	*RPL12*
SLC10A7	*MB21D2*	*RPL13*
SLC13A1	*MBD1*	*RPL13A*
SLC14A1	*MBD2*	*RPL14*
SLC15A2	*MBD5*	*RPL15*
SLC16A6	*MBD6*	*RPL17-C18orf32*
SLC16A7	*MBL2*	*RPL21*
SLC17A2	*MBLAC2*	*RPL24*
SLC17A4	*MBNL1*	*RPL27A*
SLC17A6	*MBNL2*	*RPL28*
SLC17A8	*MBNL3*	*RPL31*
SLC18A1	*MBOAT2*	*RPL37*
SLC19A1	*MBOAT7*	*RPL37A*
SLC19A3	*MBP*	*RPL3L*
SLC1A1	*MBTD1*	*RPL7*
SLC1A2	*MBTPS1*	*RPL8*
SLC22A10	*MBTPS2*	*RPLP0*
SLC22A11	*MC2R*	*RPP14*
SLC22A15	*MCAT*	*RPP40*
SLC22A9	*MCC*	*RPRD1B*
SLC24A1	*MCCC1*	*RPRD2*
SLC24A2	*MCCC2*	*RPS14*
SLC24A3	*MCEMP1*	*RPS15A*
SLC24A4	*MCF2*	*RPS20*
SLC25A1	*MCF2L2*	*RPS23*
SLC25A10	*MCFD2*	*RPS24*
SLC25A11	*MCHR2*	*RPS29*
SLC25A12	*MCL1*	*RPS3*

SLC25A13	*MCM10*	*RPS6KA1*
SLC25A15	*MCM3AP*	*RPS6KA6*
SLC25A16	*MCM5*	*RPS6KB1*
SLC25A21	*MCM8*	*RPS7*
SLC25A21-AS1	*MCM9*	*RPTOR*
SLC25A24	*MCMBP*	*RPUSD2*
SLC25A26	*MCMDC2*	*RRAGC*
SLC25A34	*MCOLN2*	*RREB1*
SLC25A36	*MCPH1*	*RRM2B*
SLC25A37	*MCTP1*	*RRN3*
SLC25A39	*MCTP2*	*RRP12*
SLC25A43	*MCTS1*	*RRP15*
SLC25A44	*MCU*	*RRP36*
SLC25A45	*MCUB*	*RRP8*
SLC25A46	*MCUR1*	*RS1*
SLC25A51	*MDFIC*	*RSBN1*
SLC26A2	*MDH2*	*RSBN1L*
SLC27A4	*MDM1*	*RSC1A1*
SLC28A1	*MDM4*	*RSF1*
SLC29A1	*MDN1*	*RSKR*
SLC2A10	*ME1*	*RSL1D1*
SLC2A13	*ME2*	*RSPH3*
SLC2A3	*MEAF6*	*RSPH9*
SLC2A4	*MECOM*	*RSPO2*
SLC2A5	*MECP2*	*RSPO3*
SLC30A1	*MED1*	*RSRC2*
SLC30A4	*MED10*	*RTCA*
SLC30A5	*MED12L*	*RTF1*
SLC30A6	*MED13*	*RTKN2*
SLC30A7	*MED13L*	*RTL4*
SLC30A8	*MED14*	*RTL6*
SLC31A1	*MED17*	*RTL8B*
SLC34A1	*MED18*	*RTN1*
SLC34A2	*MED21*	*RTN3*
SLC35A2	*MED28*	*RUBCN*
SLC35A4	*MED4*	*RUBCNL*
SLC35B4	*MED6*	*RUFY2*
SLC35D2	*MED7*	*RUNDC1*
SLC35E1	*MED9*	*RUNDC3A*
SLC35E4	*MEF2A*	*RUNDC3B*
SLC35F1	*MEF2C*	*RUNX1*
SLC35F5	*MEFV*	*RUNX1T1*
SLC35F6	*MEGF10*	*RUNX2*
SLC36A1	*MEGF6*	*RWDD1*

SLC36A3	*MEGF8*	*RWDD2A*
SLC36A4	*MEGF9*	*RWDD2B*
SLC37A4	*MEI4*	*RXFP3*
SLC38A1	*MEIOC*	*RYR2*
SLC38A7	*MEIS1*	*S100A16*
SLC38A9	*MEIS2*	*S100B*
SLC39A10	*MELK*	*S100PBP*
SLC39A13	*MEMO1*	*S1PR1*
SLC39A14	*MEP1A*	*S1PR3*
SLC39A2	*MEPCE*	*SACM1L*
SLC39A6	*MERTK*	*SAFB*
SLC39A7	*MESD*	*SAFB2*
SLC39A8	*MESP1*	*SALL1*
SLC39A9	*MEST*	*SALL4*
SLC3A1	*MET*	*SAMD1*
SLC40A1	*METAP1*	*SAMD10*
SLC41A1	*METAP2*	*SAMD12*
SLC44A1	*METTL14*	*SAMD4A*
SLC44A5	*METTL15*	*SAMD5*
SLC45A3	*METTL16*	*SAMD8*
SLC47A1	*METTL21A*	*SAMHD1*
SLC4A4	*METTL22*	*SAP130*
SLC4A7	*METTL24*	*SAP30*
SLC4A8	*METTL2A*	*SAP30L*
SLC5A12	*METTL4*	*SAPCD2*
SLC5A3	*METTL6*	*SAR1A*
SLC5A7	*METTL8*	*SAR1B*
SLC6A1	*MEX3A*	*SARAF*
SLC6A11	*MEX3B*	*SARDH*
SLC6A15	*MFAP3L*	*SARS*
SLC6A17	*MFAP5*	*SATB1*
SLC6A19	*MFF*	*SAXO2*
SLC6A2	*MFHAS1*	*SBF1*
SLC6A3	*MFN1*	*SBK1*
SLC6A8	*MFSD11*	*SC5D*
SLC6A9	*MFSD13A*	*SCAF8*
SLC7A11	*MFSD14B*	*SCAI*
SLC7A14	*MFSD14C*	*SCARB2*
SLC7A2	*MFSD2A*	*SCD*
SLC7A6	*MFSD2B*	*SCD5*
SLC7A6OS	*MFSD4A*	*SCG3*
SLC7A9	*MFSD4B*	*SCHIP1*
SLC8A1	*MFSD6*	*SCIN*
SLC9A3R2	*MFSD9*	*SCLT1*

SLC9B2	*MGA*	*SCN11A*
SLCO1A2	*MGAM*	*SCN1A*
SLCO1C1	*MGARP*	*SCN8A*
SLCO3A1	*MGAT1*	*SCN9A*
SLCO5A1	*MGAT2*	*SCO1*
SLF1	*MGAT3*	*SCRN1*
SLF2	*MGAT4A*	*SCRN3*
SLFN11	*MGC15885*	*SCUBE3*
SLFN12L	*MGME1*	*SDAD1*
SLFN5	*MGP*	*SDC1*
SLFNL1	*MGRN1*	*SDC2*
SLIT1	*MGST1*	*SDCBP*
SLIT2	*MGST3*	*SDE2*
SLITRK5	*MIA2*	*SDHAF1*
SLK	*MIA3*	*SDHAF2*
SLTM	*MIB1*	*SDK1*
SLX4IP	*MICA*	*SDR16C5*
SMAD2	*MICALCL*	*SEC16B*
SMAD5	*MICALL1*	*SEC22C*
SMAD7	*MICOS10*	*SEC23IP*
SMAP1	*MICU2*	*SEC24A*
SMARCA1	*MICU3*	*SEC24B*
SMARCA2	*MID1*	*SEC24C*
SMARCA4	*MID2*	*SEC31B*
SMARCA5	*MIDN*	*SEC62*
SMARCD1	*MIEF1*	*SEC63*
SMARCE1	*MIEF2*	*SECISBP2*
SMC1A	*MIER1*	*SECISBP2L*
SMC2	*MIER3*	*SEH1L*
SMC3	*MIGA1*	*SELENOF*
SMC6	*MINAR1*	*SELENOI*
SMG7	*MINAR2*	*SELENOS*
SMG9	*MINDY2*	*SELENOT*
SMIM10	*MINK1*	*SELENOV*
SMIM11A	*MINPP1*	*SELP*
SMIM12	*MIOS*	*SEM1*
SMIM13	*MIPEP*	*SEMA3A*
SMIM15	*MIPOL1*	*SEMA3C*
SMIM17	*MIR2052HG*	*SEMA3D*
SMIM21	*MIR6501*	*SEMA4B*
SMIM30	*MIS12*	*SEMA4C*
SMLR1	*MIS18BP1*	*SEMA4F*
SMNDC1	*MITF*	*SEMA4G*
SMOC1	*MKI67*	*SEMA5A*

SMOX	*MKKS*	*SEMA6A*
SMPD3	*MKLN1*	*SEMA6D*
SMTNL2	*MKRN1*	*SENP2*
SMUG1	*MKRN2*	*SENP7*
SMURF1	*MKX*	*SEPHS1*
SMYD2	*MLANA*	*SEPTIN14*
SNAP23	*MLEC*	*SEPTIN2*
SNAP25	*MLH1*	*SEPTIN6*
SNAP29	*MLH3*	*SEPTIN7*
SNAP47	*MLIP*	*SEPTIN8*
SNAPC1	*MLLT10*	*SERAC1*
SNCB	*MLLT3*	*SERBP1*
SND1	*MLXIP*	*SERHL2*
SNF8	*MMAB*	*SERINC1*
SNN	*MMADHC*	*SERINC3*
SNPH	*MMD*	*SERINC4*
SNRNP200	*MMGT1*	*SERPINB2*
SNRNP40	*MMP1*	*SERPINB5*
SNRNP48	*MMP10*	*SERPINB9*
SNRPD1	*MMP13*	*SERPIND1*
SNRPD3	*MMP16*	*SERPING1*
SNTB2	*MMP17*	*SERPINH1*
SNX1	*MMP19*	*SERTAD2*
SNX11	*MMP20*	*SERTAD4*
SNX13	*MMP24*	*SERTM1*
SNX19	*MMP27*	*SESN2*
SNX2	*MMP8*	*SESN3*
SNX21	*MMRN2*	*SESTD1*
SNX24	*MMS22L*	*SETD1A*
SNX27	*MMUT*	*SETD4*
SNX29	*MN1*	*SETD5*
SNX30	*MNAT1*	*SETD7*
SNX32	*MNDA*	*SETD9*
SNX33	*MNS1*	*SETMAR*
SNX4	*MOB1A*	*SEZ6L*
SOBP	*MOB1B*	*SF1*
SOCS1	*MOB3A*	*SF3A1*
SOCS3	*MOB3B*	*SF3A3*
SOCS4	*MOB4*	*SF3B1*
SOCS5	*MOCOS*	*SF3B3*
SOD2	*MOCS2*	*SF3B4*
SON	*MOCS3*	*SF3B6*
SORCS3	*MOGS*	*SFPQ*
SORD	*MON1B*	*SFRP1*

SORL1	*MON2*	*SFRP2*
SORT1	*MORC1*	*SFT2D2*
SOS2	*MORC2*	*SFT2D3*
SOSTDC1	*MORC4*	*SFXN1*
SOWAHC	*MORF4L1*	*SFXN2*
SOX1	*MORF4L2*	*SFXN5*
SOX10	*MOSMO*	*SGCD*
SOX11	*MOSPD1*	*SGCE*
SOX3	*MOSPD2*	*SGO1*
SOX4	*MOXD1*	*SGPL1*
SOX5	*MPC1*	*SGTA*
SP1	*MPC2*	*SGTB*
SP100	*MPDZ*	*SH2D1B*
SP3	*MPEG1*	*SH2D4A*
SP4	*MPHOSPH8*	*SH3BGRL*
SP8	*MPI*	*SH3BGRL2*
SPA17	*MPIG6B*	*SH3BP2*
SPAG6	*MPL*	*SH3BP4*
SPAG9	*MPLKIP*	*SH3D19*
SPARC	*MPND*	*SH3D21*
SPART	*MPP1*	*SH3GLB1*
SPATA17	*MPP3*	*SH3KBP1*
SPATA2	*MPP4*	*SH3PXD2B*
SPATA5	*MPP5*	*SH3RF2*
SPATS2	*MPP6*	*SH3RF3*
SPATS2L	*MPP7*	*SHB*
SPCS1	*MPPED1*	*SHC3*
SPCS2	*MPPED2*	*SHCBP1*
SPECC1	*MPRIP*	*SHE*
SPEF2	*MPV17L*	*SHH*
SPIB	*MPZL1*	*SHISA6*
SPIC	*MPZL2*	*SHISA7*
SPICE1	*MPZL3*	*SHISA9*
SPIDR	*MRAP2*	*SHISAL1*
SPIN3	*MRAS*	*SHLD2*
SPIN4	*MRE11*	*SHMT1*
SPINT1	*MREG*	*SHOC2*
SPNS1	*MRGBP*	*SHPRH*
SPOCK1	*MRGPRF*	*SHQ1*
SPOCK2	*MRI1*	*SHROOM1*
SPOCK3	*MRM3*	*SHROOM2*
SPOUT1	*MRO*	*SHROOM3*
SPPL2A	*MROH1*	*SHROOM4*
SPPL3	*MROH9*	*SHTN1*

SPRED1	*MRPL1*	*SI*
SPRED2	*MRPL10*	*SIAE*
SPRY1	*MRPL11*	*SIAH3*
SPRY4	*MRPL12*	*SIGIRR*
SPRYD4	*MRPL13*	*SIGLEC1*
SPTBN1	*MRPL17*	*SIGLEC11*
SPTBN2	*MRPL18*	*SIGLEC14*
SPTSSA	*MRPL19*	*SIGLEC6*
SQSTM1	*MRPL22*	*SIGLEC9*
SRD5A1	*MRPL3*	*SIKE1*
SRD5A3	*MRPL30*	*SIM1*
SREBF2	*MRPL34*	*SIM2*
SREK1	*MRPL35*	*SIN3A*
SREK1IP1	*MRPL36*	*SIN3B*
SRFBP1	*MRPL38*	*SIPA1*
SRGAP1	*MRPL42*	*SIRPA*
SRI	*MRPL43*	*SIRT1*
SRPK2	*MRPL44*	*SIX1*
SRPRA	*MRPL46*	*SIX4*
SRPRB	*MRPL50*	*SIX6*
SRPX2	*MRPL51*	*SKA3*
SRR	*MRPS10*	*SKAP2*
SRRM2	*MRPS14*	*SKI*
SRRM4	*MRPS16*	*SKP1*
SRRT	*MRPS17*	*SLAIN2*
SRSF1	*MRPS18C*	*SLAMF1*
SRSF10	*MRPS23*	*SLAMF6*
SRSF11	*MRPS25*	*SLAMF7*
SRSF2	*MRPS30*	*SLAMF8*
SRSF3	*MRPS31*	*SLBP*
SRSF7	*MRPS35*	*SLC10A2*
SS18	*MRPS36*	*SLC10A7*
SSBP2	*MRPS5*	*SLC13A1*
SSBP3	*MRRF*	*SLC14A1*
SSC4D	*MRS2*	*SLC15A2*
SSH2	*MRTFA*	*SLC16A6*
SSPN	*MRTO4*	*SLC16A7*
SSR1	*MS4A15*	*SLC17A2*
SSR3	*MS4A2*	*SLC17A4*
SSR4P1	*MS4A3*	*SLC17A6*
SSRP1	*MS4A4A*	*SLC17A8*
SSX2	*MS4A6A*	*SLC18A1*
SSX2IP	*MS4A7*	*SLC19A1*
SSX3	*MSANTD2*	*SLC19A3*

ST13	*MSANTD3*	*SLC1A1*
ST3GAL6	*MSANTD4*	*SLC1A2*
ST5	*MSC*	*SLC1A5*
ST6GAL1	*MSH2*	*SLC22A11*
ST6GAL2	*MSH3*	*SLC22A15*
ST6GALNAC3	*MSH4*	*SLC22A9*
ST6GALNAC5	*MSH6*	*SLC23A2*
ST7L	*MSI2*	*SLC24A1*
ST8SIA1	*MSL1*	*SLC24A2*
ST8SIA3	*MSL2*	*SLC24A4*
ST8SIA4	*MSL3*	*SLC24A5*
STAC	*MSMO1*	*SLC25A1*
STAM	*MSN*	*SLC25A10*
STAMBP	*MSR1*	*SLC25A11*
STAMBPL1	*MSRB2*	*SLC25A12*
STARD4	*MSRB3*	*SLC25A13*
STARD5	*MSTN*	*SLC25A16*
STARD8	*MSX2*	*SLC25A21*
STAT2	*MT1A*	*SLC25A21-AS1*
STAT3	*MT1F*	*SLC25A24*
STAT5A	*MTA3*	*SLC25A26*
STAT5B	*MTBP*	*SLC25A30*
STAU2	*MTCH2*	*SLC25A34*
STC1	*MTDH*	*SLC25A36*
STIL	*MTERF1*	*SLC25A37*
STIM1	*MTERF2*	*SLC25A39*
STIM2	*MTF2*	*SLC25A4*
STK10	*MTFMT*	*SLC25A43*
STK17B	*MTFR1*	*SLC25A44*
STK24	*MTFR1L*	*SLC25A45*
STK25	*MTG1*	*SLC25A46*
STK32A	*MTHFD1L*	*SLC26A2*
STK33	*MTHFD2*	*SLC26A7*
STK35	*MTHFD2L*	*SLC27A4*
STK38L	*MTHFS*	*SLC28A1*
STK4	*MTM1*	*SLC29A1*
STMN1	*MTMR1*	*SLC2A10*
STMP1	*MTMR10*	*SLC2A13*
STN1	*MTMR14*	*SLC2A3*
STOML1	*MTMR2*	*SLC2A4*
STOX2	*MTMR3*	*SLC2A5*
STPG1	*MTMR6*	*SLC30A1*
STPG4	*MTMR7*	*SLC30A4*
STRAP	*MTMR8*	*SLC30A6*

STRN	*MTMR9*	*SLC30A7*
STS	*MTO1*	*SLC30A8*
STUM	*MTPAP*	*SLC31A1*
STX12	*MTPN*	*SLC34A1*
STX17	*MTR*	*SLC34A2*
STX1B	*MTREX*	*SLC35A4*
STX4	*MTRF1L*	*SLC35A5*
STX6	*MTRNR2L1*	*SLC35B4*
STX7	*MTRNR2L4*	*SLC35D1*
STXBP4	*MTRR*	*SLC35D2*
STYX	*MTSS1*	*SLC35E1*
STYXL1	*MTTP*	*SLC35E4*
SUB1	*MTURN*	*SLC35F1*
SUCNR1	*MTUS1*	*SLC35F5*
SUGT1	*MTX3*	*SLC35F6*
SULT1B1	*MUC15*	*SLC36A1*
SULT1C2	*MUC7*	*SLC36A3*
SULT4A1	*MUCL1*	*SLC37A4*
SUMO2	*MUCL3*	*SLC38A1*
SUN2	*MUSK*	*SLC38A7*
SUPT7L	*MVP*	*SLC38A9*
SURF6	*MXD1*	*SLC39A1*
SUSD1	*MXD3*	*SLC39A10*
SUSD4	*MXD4*	*SLC39A13*
SUSD5	*MXI1*	*SLC39A14*
SUV39H2	*MXRA7*	*SLC39A2*
SV2A	*MYADM*	*SLC39A3*
SV2B	*MYB*	*SLC39A6*
SV2C	*MYBL1*	*SLC39A7*
SVIP	*MYBPC1*	*SLC39A8*
SWAP70	*MYCBP*	*SLC39A9*
SWT1	*MYCBP2*	*SLC3A1*
SYAP1	*MYCT1*	*SLC40A1*
SYBU	*MYD88*	*SLC41A1*
SYCE2	*MYEF2*	*SLC44A1*
SYDE2	*MYF5*	*SLC44A5*
SYK	*MYH10*	*SLC45A3*
SYNC	*MYH9*	*SLC45A4*
SYNCRIP	*MYL12A*	*SLC47A1*
SYNDIG1L	*MYLIP*	*SLC4A4*
SYNE4	*MYLK3*	*SLC4A7*
SYNGR1	*MYNN*	*SLC4A8*
SYNGR3	*MYO16*	*SLC5A12*
SYNJ2	*MYO1A*	*SLC5A3*

SYNJ2BP	*MYO1B*	*SLC5A7*
SYNPO	*MYO3A*	*SLC6A1*
SYNPO2	*MYO5A*	*SLC6A11*
SYNPO2L	*MYO5B*	*SLC6A15*
SYNPR	*MYO5C*	*SLC6A17*
SYS1	*MYO6*	*SLC6A19*
SYT1	*MYO9A*	*SLC6A3*
SYT10	*MYOC*	*SLC6A8*
SYT11	*MYOCD*	*SLC6A9*
SYT7	*MYOF*	*SLC7A11*
SZRD1	*MYOM2*	*SLC7A14*
SZT2	*MYORG*	*SLC7A2*
TAB1	*MYOZ2*	*SLC7A6*
TAB3	*MYOZ3*	*SLC7A6OS*
TACR1	*MYPN*	*SLC7A9*
TADA2B	*MYPOP*	*SLC8A1*
TAF11	*MYRIP*	*SLC9A2*
TAF12	*MYSM1*	*SLC9A3R2*
TAF13	*MYT1*	*SLC9A7*
TAF1A	*MYT1L*	*SLC9B2*
TAF1D	*MYZAP*	*SLCO1A2*
TAF3	*MZT1*	*SLCO1C1*
TAF4B	*N4BP2*	*SLCO2B1*
TAF5L	*N4BP2L1*	*SLCO3A1*
TAF7L	*N4BP2L2*	*SLCO5A1*
TAF9B	*N4BP3*	*SLF1*
TAFA1	*N6AMT1*	*SLF2*
TAFA2	*NAA11*	*SLFN12L*
TAL1	*NAA15*	*SLFN5*
TANC2	*NAA16*	*SLFNL1*
TANGO6	*NAA25*	*SLIT1*
TAOK1	*NAA30*	*SLIT2*
TAPBP	*NAA35*	*SLITRK5*
TARDBP	*NAA40*	*SLK*
TARS2	*NAA50*	*SLMAP*
TARSL2	*NAAA*	*SLTM*
TASOR	*NAALAD2*	*SLX4IP*
TATDN2	*NAALADL2*	*SMAD2*
TBC1D12	*NAB1*	*SMAD5*
TBC1D19	*NABP1*	*SMAD7*
TBC1D20	*NACC1*	*SMAP1*
TBC1D22B	*NADK*	*SMARCA1*
TBC1D24	*NADK2*	*SMARCA2*
TBC1D26	*NAF1*	*SMARCA5*

TBC1D30	*NAGA*	*SMARCAD1*
TBC1D4	*NAGPA*	*SMARCD1*
TBC1D8B	*NAIP*	*SMC1A*
TBCEL	*NALCN*	*SMC2*
TBL1X	*NAMPT*	*SMC3*
TBL1XR1	*NANOG*	*SMCHD1*
TBPL2	*NANOGNB*	*SMCR8*
TBR1	*NANOS1*	*SMG1*
TBX1	*NAP1L1*	*SMG7*
TBX15	*NAP1L2*	*SMG9*
TBX18	*NAP1L4*	*SMIM12*
TBX20	*NAP1L5*	*SMIM13*
TBX3	*NAP1L6*	*SMIM15*
TBXT	*NAPB*	*SMIM17*
TCEA1	*NAPEPLD*	*SMIM19*
TCEAL1	*NAPG*	*SMIM21*
TCERG1	*NARS*	*SMIM30*
TCF12	*NARS2*	*SMLR1*
TCF23	*NAT1*	*SMNDC1*
TCF3	*NAT10*	*SMOC1*
TCF4	*NBEA*	*SMOX*
TCF7L2	*NBEAL1*	*SMPD3*
TCFL5	*NBN*	*SMTNL2*
TCHP	*NBPF1*	*SMUG1*
TCIRG1	*NBPF3*	*SMURF1*
TCL1A	*NBPF4*	*SMYD2*
TCOF1	*NBR1*	*SNAP23*
TCP10	*NCALD*	*SNAP25*
TCTA	*NCAM1*	*SNAP29*
TCTE1	*NCAM2*	*SNAP47*
TCTN2	*NCAN*	*SNAPC1*
TDG	*NCAPD3*	*SNCB*
TDRD1	*NCAPG*	*SND1*
TDRD7	*NCAPG2*	*SNF8*
TDRKH	*NCAPH*	*SNN*
TDRP	*NCBP1*	*SNPH*
TEC	*NCBP2*	*SNRNP200*
TECPR2	*NCEH1*	*SNRNP48*
TECRL	*NCK1*	*SNRPD1*
TEDC1	*NCKAP1*	*SNRPD3*
TEK	*NCKAP1L*	*SNTB2*
TELO2	*NCKAP5*	*SNX1*
TENM2	*NCKIPSD*	*SNX11*
TENM3	*NCL*	*SNX13*

TENM4	*NCMAP*	*SNX19*
TENT4A	*NCOA1*	*SNX2*
TENT5C	*NCOA2*	*SNX24*
TEP1	*NCOA4*	*SNX27*
TERF2	*NCOA5*	*SNX29*
TERF2IP	*NCOA7*	*SNX30*
TESMIN	*NCOR1*	*SNX32*
TET2	*NCR3LG1*	*SNX33*
TET3	*NCS1*	*SNX4*
TEX10	*NDC1*	*SOAT1*
TEX13B	*NDEL1*	*SOBP*
TEX19	*NDFIP1*	*SOCS1*
TEX2	*NDN*	*SOCS3*
TEX22	*NDNF*	*SOCS4*
TFAP2A	*NDP*	*SOCS5*
TFAP2B	*NDRG1*	*SOCS6*
TFB1M	*NDRG3*	*SOD2*
TFCP2	*NDST3*	*SOGA1*
TFCP2L1	*NDST4*	*SORCS3*
TFEC	*NDUFA10*	*SORD*
TFG	*NDUFA4L2*	*SORL1*
TFR2	*NDUFA5*	*SORT1*
TFRC	*NDUFA8*	*SOS2*
TGFA	*NDUFA9*	*SOSTDC1*
TGFB2	*NDUFAF4*	*SOWAHC*
TGFBR1	*NDUFAF5*	*SOX1*
TGFBR2	*NDUFAF7*	*SOX10*
TGFBR3	*NDUFB4*	*SOX11*
TGFBRAP1	*NDUFB5*	*SOX3*
TGIF1	*NDUFB9*	*SOX4*
TGIF2LX	*NDUFC1*	*SP1*
TGIF2LY	*NDUFC2*	*SP100*
TGOLN2	*NDUFS1*	*SP140L*
TGS1	*NDUFV3*	*SP3*
THAP1	*NEB*	*SP4*
THAP2	*NEBL*	*SP6*
THAP5	*NECAB1*	*SPA17*
THAP6	*NECAB3*	*SPAG5*
THAP8	*NECAP1*	*SPAG6*
THBD	*NECTIN3*	*SPAG9*
THBS1	*NEDD1*	*SPARC*
THBS2	*NEDD4*	*SPART*
THBS4	*NEDD9*	*SPATA17*
THEM4	*NEFM*	*SPATA2*

THEMIS2	*NEGR1*	*SPATA33*
THG1L	*NEIL3*	*SPATA5*
THOP1	*NEK10*	*SPATS2*
THRAP3	*NEK11*	*SPATS2L*
THRB	*NEK2*	*SPCS1*
THSD7A	*NEK3*	*SPCS2*
THSD7B	*NEK4*	*SPCS3*
THUMPD1	*NEK5*	*SPDL1*
TIAM1	*NEK7*	*SPECC1*
TIFA	*NEK9*	*SPEF2*
TIFAB	*NELL1*	*SPEN*
TIGAR	*NEMF*	*SPHAR*
TIGD2	*NEMP1*	*SPIB*
TIGD5	*NEMP2*	*SPIC*
TIGIT	*NENF*	*SPICE1*
TIMD4	*NEO1*	*SPIDR*
TIMELESS	*NEPRO*	*SPIN3*
TIMM22	*NET1*	*SPIN4*
TIMM50	*NETO1*	*SPINT1*
TIMP3	*NETO2*	*SPNS1*
TIMP4	*NEU3*	*SPOCK1*
TIPARP	*NEUROD1*	*SPOCK2*
TIPRL	*NEUROD4*	*SPOCK3*
TIRAP	*NEUROD6*	*SPOUT1*
TK1	*NEUROG2*	*SPPL2A*
TK2	*NEUROG3*	*SPPL3*
TLCD2	*NEXMIF*	*SPRED1*
TLCD4	*NF1*	*SPRED2*
TLE3	*NFAM1*	*SPRY1*
TLK2	*NFASC*	*SPRY3*
TLL1	*NFAT5*	*SPRY4*
TLL2	*NFATC1*	*SPRYD3*
TLN1	*NFATC2*	*SPRYD4*
TLN2	*NFATC4*	*SPTB*
TLNRD1	*NFE2L3*	*SPTBN1*
TLR10	*NFIA*	*SPTBN2*
TLR2	*NFIB*	*SPTBN5*
TLR8	*NFIL3*	*SPTLC3*
TM2D2	*NFKB2*	*SPTSSA*
TM2D3	*NFKBIA*	*SQSTM1*
TMBIM1	*NFKBIZ*	*SRC*
TMBIM6	*NFRKB*	*SRD5A3*
TMC6	*NFS1*	*SREBF2*
TMC7	*NFXL1*	*SREK1*

TMCC1	*NFYA*	*SREK1IP1*
TMCC3	*NFYC*	*SRFBP1*
TMCO1	*NGDN*	*SRGAP1*
TMCO4	*NGRN*	*SRGAP3*
TMED3	*NHLH1*	*SRGN*
TMED5	*NHLRC2*	*SRI*
TMED7	*NHLRC3*	*SRP68*
TMEM106B	*NHS*	*SRPK2*
TMEM11	*NIBAN1*	*SRPRA*
TMEM120B	*NID1*	*SRPX2*
TMEM127	*NIF3L1*	*SRR*
TMEM129	*NIFK*	*SRRD*
TMEM132B	*NIN*	*SRSF1*
TMEM132C	*NINL*	*SRSF10*
TMEM139	*NIPA1*	*SRSF11*
TMEM140	*NIPA2*	*SRSF2*
TMEM141	*NIPAL1*	*SRSF3*
TMEM151A	*NIPAL2*	*SRSF7*
TMEM151B	*NIPAL3*	*SRXN1*
TMEM154	*NIPAL4*	*SSBP2*
TMEM158	*NIPBL*	*SSBP3*
TMEM164	*NIPSNAP2*	*SSC4D*
TMEM165	*NIPSNAP3A*	*SSH2*
TMEM167A	*NIPSNAP3B*	*SSH3*
TMEM167B	*NIT1*	*SSPN*
TMEM168	*NKAIN1*	*SSR1*
TMEM169	*NKAIN2*	*SSR3*
TMEM170A	*NKAP*	*SSR4P1*
TMEM170B	*NKD1*	*SSRP1*
TMEM178B	*NKIRAS1*	*SSX2*
TMEM18	*NKIRAS2*	*SSX2IP*
TMEM182	*NKRF*	*SSX3*
TMEM185B	*NKTR*	*ST13*
TMEM186	*NKX2-1*	*ST3GAL6*
TMEM19	*NKX2-2*	*ST5*
TMEM198	*NKX2-4*	*ST6GAL1*
TMEM200B	*NKX3-1*	*ST6GAL2*
TMEM201	*NKX3-2*	*ST6GALNAC3*
TMEM207	*NKX6-2*	*ST6GALNAC5*
TMEM209	*NLE1*	*ST7L*
TMEM211	*NLGN1*	*ST8SIA1*
TMEM212	*NLGN4X*	*ST8SIA3*
TMEM214	*NLK*	*ST8SIA4*
TMEM220	*NLN*	*STAC*

TMEM230	*NLRP6*	*STAG2*
TMEM231	*NLRP9*	*STAM*
TMEM233	*NMD3*	*STAM2*
TMEM235	*NME6*	*STAMBP*
TMEM236	*NMNAT1*	*STAMBPL1*
TMEM237	*NMNAT2*	*STARD3NL*
TMEM241	*NMT2*	*STARD4*
TMEM245	*NNT*	*STARD5*
TMEM246	*NOA1*	*STARD8*
TMEM248	*NOC3L*	*STAT1*
TMEM25	*NOCT*	*STAT2*
TMEM251	*NOD2*	*STAT3*
TMEM253	*NOL10*	*STAT5A*
TMEM258	*NOL11*	*STAT5B*
TMEM26	*NOL12*	*STAT6*
TMEM263	*NOL4*	*STAU2*
TMEM268	*NOL4L*	*STC1*
TMEM33	*NOL7*	*STIL*
TMEM39A	*NOL8*	*STIM1*
TMEM40	*NOLC1*	*STIM2*
TMEM41A	*NOM1*	*STK10*
TMEM43	*NOP53*	*STK17B*
TMEM52B	*NOP9*	*STK24*
TMEM53	*NOS1*	*STK25*
TMEM62	*NOS3*	*STK32A*
TMEM63C	*NOTCH2*	*STK33*
TMEM64	*NOTCH3*	*STK35*
TMEM65	*NOTO*	*STK38L*
TMEM74B	*NOVA1*	*STK4*
TMEM86A	*NOVA2*	*STMN1*
TMEM87A	*NOX4*	*STMP1*
TMEM87B	*NPAP1*	*STN1*
TMEM92	*NPAS3*	*STOML1*
TMEM9B	*NPAS4*	*STOX2*
TMLHE	*NPAT*	*STPG1*
TMOD2	*NPC1*	*STPG4*
TMOD3	*NPEPPS*	*STRAP*
TMPO	*NPFFR1*	*STRN*
TMPPE	*NPHP1*	*STS*
TMPRSS11B	*NPHP3*	*STUM*
TMPRSS4	*NPL*	*STX12*
TMSB15A	*NPLOC4*	*STX17*
TMSB15B	*NPM1*	*STX1B*
TMTC1	*NPPC*	*STX3*

TMTC2	*NPR3*	*STX6*
TMUB1	*NPRL2*	*STX7*
TNF	*NPTXR*	*STXBP4*
TNFAIP2	*NPY*	*STXBP5L*
TNFAIP3	*NPY1R*	*STYX*
TNFAIP8	*NPY2R*	*STYXL1*
TNFAIP8L1	*NPY4R*	*SUB1*
TNFRSF10A	*NPY5R*	*SUCLG2*
TNFRSF10C	*NQO2*	*SUCNR1*
TNFRSF11B	*NR0B1*	*SUGT1*
TNFRSF13C	*NR1D2*	*SULT1B1*
TNFRSF14	*NR1H4*	*SULT1C2*
TNFRSF19	*NR2C1*	*SULT1E1*
TNFRSF1B	*NR2C2*	*SULT4A1*
TNFRSF8	*NR2F2*	*SUN2*
TNFSF10	*NR4A2*	*SUPT20H*
TNFSF12-TNFSF13	*NR5A2*	*SUPT3H*
TNFSF13	*NR6A1*	*SUPT7L*
TNFSF13B	*NRAS*	*SURF6*
TNFSF15	*NRBP2*	*SUSD1*
TNFSF4	*NRCAM*	*SUSD5*
TNIK	*NREP*	*SUV39H2*
TNIP1	*NRF1*	*SV2A*
TNKS	*NRG1*	*SV2B*
TNKS2	*NRG2*	*SV2C*
TNN	*NRG3*	*SVIP*
TNNI1	*NRG4*	*SWAP70*
TNPO1	*NRK*	*SWT1*
TNR	*NRL*	*SYAP1*
TNRC6A	*NRN1*	*SYBU*
TNRC6B	*NRP1*	*SYCE2*
TNRC6C	*NRP2*	*SYDE2*
TNS3	*NRXN1*	*SYK*
TNS4	*NRXN3*	*SYNC*
TOGARAM2	*NSD1*	*SYNCRIP*
TOLLIP	*NSD2*	*SYNGR1*
TOM1	*NSD3*	*SYNGR3*
TOM1L1	*NSDHL*	*SYNJ2*
TOMM20	*NSF*	*SYNJ2BP*
TOMM34	*NSL1*	*SYNPO*
TOMM7	*NSMAF*	*SYNPO2*
TOP3A	*NSMCE2*	*SYNPO2L*
TOPBP1	*NSRP1*	*SYNPR*
TOR1AIP1	*NSUN2*	*SYS1*

TOR1AIP2	*NSUN3*	*SYT1*
TOX	*NSUN4*	*SYT10*
TOX3	*NSUN5*	*SYT11*
TP53	*NSUN7*	*SYT13*
TP53BP2	*NT5C1A*	*SYT7*
TP53INP1	*NT5C1B*	*SYTL4*
TP53INP2	*NT5C1B-RDH14*	*SZRD1*
TP63	*NT5C2*	*SZT2*
TPD52	*NT5DC1*	*TAB3*
TPD52L1	*NT5DC3*	*TACO1*
TPGS2	*NT5DC4*	*TACR1*
TPH1	*NTM*	*TADA2B*
TPK1	*NTN4*	*TAF10*
TPM3	*NTN5*	*TAF11*
TPPP3	*NTNG1*	*TAF12*
TPRA1	*NTNG2*	*TAF13*
TPRG1	*NTPCR*	*TAF1A*
TPRG1L	*NTRK2*	*TAF1D*
TPT1	*NTRK3*	*TAF3*
TRA2B	*NUAK1*	*TAF4*
TRABD2A	*NUBPL*	*TAF4B*
TRABD2B	*NUCB1*	*TAF5L*
TRAF2	*NUCB2*	*TAF7L*
TRAF3IP1	*NUCKS1*	*TAF8*
TRAF3IP2	*NUDCD1*	*TAF9B*
TRAF4	*NUDCD2*	*TAFA1*
TRAF5	*NUDCD3*	*TAFA2*
TRAF6	*NUDT11*	*TAGAP*
TRAK1	*NUDT12*	*TAL1*
TRAM1	*NUDT15*	*TAL2*
TRAM2	*NUDT16*	*TANC2*
TRAP1	*NUDT19*	*TANGO6*
TRAPPC10	*NUDT21*	*TAOK1*
TRAPPC6B	*NUDT3*	*TAPBP*
TRAPPC9	*NUDT4*	*TARDBP*
TRARG1	*NUDT5*	*TARS2*
TRAT1	*NUDT9*	*TARSL2*
TRDMT1	*NUF2*	*TAS2R5*
TREML2	*NUFIP1*	*TASOR*
TRIM14	*NUFIP2*	*TBC1D12*
TRIM2	*NUGGC*	*TBC1D15*
TRIM24	*NUMBL*	*TBC1D19*
TRIM28	*NUP133*	*TBC1D20*
TRIM35	*NUP160*	*TBC1D22B*

TRIM36	*NUP205*	*TBC1D24*
TRIM4	*NUP214*	*TBC1D26*
TRIM44	*NUP35*	*TBC1D30*
TRIM47	*NUP37*	*TBC1D4*
TRIM5	*NUP42*	*TBC1D8B*
TRIM58	*NUP43*	*TBC1D9*
TRIM62	*NUP50*	*TBCA*
TRIM64B	*NUP58*	*TBCEL*
TRIM71	*NUP62*	*TBK1*
TRIM72	*NUP62CL*	*TBL1X*
TRIM74	*NUP88*	*TBL1XR1*
TRIM9	*NUP93*	*TBPL2*
TRIP13	*NUP98*	*TBR1*
TRIP4	*NUS1*	*TBX15*
TRIQK	*NUTF2*	*TBX18*
TRMO	*NUTM2D*	*TBX20*
TRMT10B	*NVL*	*TBX3*
TRMT2A	*NWD1*	*TBXT*
TRMT5	*NWD2*	*TCEA1*
TRMT6	*NXF1*	*TCEAL1*
TRMT9B	*NXNL2*	*TCEAL8*
TRNP1	*NXPH1*	*TCF12*
TROAP	*NXPH2*	*TCF21*
TRPA1	*NXPH3*	*TCF23*
TRPC1	*NXT2*	*TCF24*
TRPC3	*NYAP1*	*TCF3*
TRPC4	*NYNRIN*	*TCF4*
TRPC4AP	*OARD1*	*TCF7L2*
TRPC5	*OAS1*	*TCFL5*
TRPM3	*OAS2*	*TCHP*
TRPS1	*OAS3*	*TCL1A*
TRPV2	*OASL*	*TCP10*
TRUB1	*OAT*	*TCTA*
TSC1	*OCIAD1*	*TCTE1*
TSC22D2	*OCIAD2*	*TCTN2*
TSC22D3	*OCLN*	*TDG*
TSEN2	*OCRL*	*TDRD1*
TSEN54	*ODAPH*	*TDRD10*
TSFM	*ODF2*	*TDRD9*
TSHZ2	*ODF2L*	*TDRKH*
TSKU	*ODF4*	*TDRP*
TSNARE1	*OGA*	*TEC*
TSPAN12	*OGFOD1*	*TECPR1*
TSPAN13	*OGFOD3*	*TECPR2*

TSPAN14	*OGFRL1*	*TECRL*
TSPAN15	*OIP5*	*TECTB*
TSPAN17	*OIT3*	*TEDC1*
TSPAN2	*OLA1*	*TEK*
TSPAN3	*OLFM1*	*TEKT1*
TSPAN5	*OLFM2*	*TELO2*
TSPAN7	*OLFM3*	*TENM1*
TSPYL1	*OLFM4*	*TENM2*
TSPYL5	*OLFML2A*	*TENM4*
TSSK1B	*OLFML3*	*TENT4A*
TSTD2	*OLIG2*	*TEPP*
TTBK2	*OLIG3*	*TERF2*
TTC13	*OLR1*	*TERF2IP*
TTC14	*OMA1*	*TESMIN*
TTC28	*OMD*	*TET2*
TTC30B	*OMG*	*TET3*
TTC31	*OMP*	*TEX19*
TTC37	*ONECUT1*	*TEX2*
TTC39A	*ONECUT2*	*TEX22*
TTC39B	*ONECUT3*	*TEX261*
TTC39C	*OPA3*	*TFAP2A*
TTC4	*OPALIN*	*TFB1M*
TTC7A	*OPCML*	*TFCP2*
TTC8	*OPHN1*	*TFCP2L1*
TTC9	*OPN1MW2*	*TFEC*
TTF2	*OPN1SW*	*TFG*
TTI2	*OPN3*	*TFR2*
TTL	*OPN4*	*TFRC*
TTLL11	*OPRK1*	*TGFA*
TTLL12	*OPRM1*	*TGFB2*
TTLL4	*OR1L8*	*TGFBR1*
TTLL7	*OR2A4*	*TGFBR2*
TTPAL	*OR2H1*	*TGFBR3*
TTR	*OR2L13*	*TGFBRAP1*
TUB	*OR52I2*	*TGIF1*
TUBA1A	*OR6C4*	*TGIF2LX*
TUBB6	*OR7A17*	*TGIF2LY*
TUBGCP3	*OR7A5*	*TGOLN2*
TULP4	*OR7D2*	*TGS1*
TVP23A	*OR9Q1*	*THAP1*
TWF1	*ORC2*	*THAP2*
TWISTNB	*ORC4*	*THAP6*
TWNK	*ORC5*	*THAP8*
TXK	*ORMDL1*	*THBD*

TXLNA	*OSBP*	*THBS1*
TXLNB	*OSBP2*	*THBS2*
TXLNG	*OSBPL10*	*THEM4*
TXN	*OSBPL11*	*THEMIS2*
TXNDC12	*OSBPL1A*	*THG1L*
TXNDC16	*OSBPL2*	*THOC5*
TXNRD2	*OSBPL3*	*THRB*
TYR	*OSBPL6*	*THSD7A*
TYRP1	*OSBPL7*	*THSD7B*
TYW3	*OSBPL8*	*THUMPD1*
UBA1	*OSCP1*	*TIAM1*
UBA2	*OSER1*	*TICAM1*
UBA6	*OSGIN2*	*TIFA*
UBAC1	*OSMR*	*TIFAB*
UBASH3B	*OSTC*	*TIGAR*
UBD	*OSTF1*	*TIGD2*
UBE2D1	*OSTM1*	*TIGD5*
UBE2D3	*OTOGL*	*TIGD6*
UBE2E1	*OTOR*	*TIGD7*
UBE2E2	*OTUB1*	*TIGIT*
UBE2E3	*OTUD1*	*TIMD4*
UBE2G2	*OTUD3*	*TIMELESS*
UBE2I	*OTUD4*	*TIMM22*
UBE2J1	*OTUD6B*	*TIMMDC1*
UBE2J2	*OTUD7A*	*TIMP3*
UBE2K	*OTULIN*	*TIMP4*
UBE2N	*OTULINL*	*TIPARP*
UBE2O	*OVCH1-AS1*	*TIPRL*
UBE2QL1	*OVOL1*	*TIRAP*
UBE2T	*OXA1L*	*TK1*
UBE2V2	*OXGR1*	*TK2*
UBE2W	*OXNAD1*	*TKFC*
UBE3B	*OXR1*	*TLCD2*
UBE4A	*OXSR1*	*TLCD3A*
UBE4B	*OXTR*	*TLCD4*
UBIAD1	*P2RX1*	*TLE3*
UBN2	*P2RX5*	*TLE4*
UBOX5	*P2RX7*	*TLK2*
UBP1	*P2RY1*	*TLL1*
UBR5	*P2RY10*	*TLL2*
UBR7	*P2RY12*	*TLN1*
UBTD2	*P2RY13*	*TLN2*
UBXN10	*P2RY14*	*TLNRD1*
UBXN2A	*P2RY2*	*TLR2*

UBXN2B	*P2RY8*	*TLR6*
UBXN7	*P3H2*	*TLR8*
UCHL5	*P4HA2*	*TM2D2*
UCMA	*P4HB*	*TM2D3*
UCP2	*PA2G4*	*TM4SF18*
UCP3	*PABPC1*	*TM9SF2*
UEVLD	*PABPC1L2A*	*TM9SF3*
UFL1	*PABPC1L2B*	*TMA16*
UGCG	*PABPC3*	*TMBIM1*
UGT2B15	*PABPC4L*	*TMBIM6*
UGT3A1	*PABPC5*	*TMC2*
UGT8	*PACC1*	*TMC5*
UHMK1	*PACRGL*	*TMC6*
UHRF1BP1L	*PACS1*	*TMC7*
UHRF2	*PACS2*	*TMCC1*
ULK2	*PACSIN2*	*TMCC3*
ULK3	*PADI2*	*TMCO1*
UMPS	*PAFAH1B1*	*TMCO4*
UNC119B	*PAFAH1B2*	*TMCO5A*
UNC13A	*PAG1*	*TMED3*
UNC13B	*PAH*	*TMED5*
UNC5B	*PAICS*	*TMED7*
UNC5C	*PAIP1*	*TMED9*
UNC5D	*PAIP2B*	*TMEM104*
UNC79	*PAK1*	*TMEM106B*
UNKL	*PAK1IP1*	*TMEM120B*
UPF1	*PAK2*	*TMEM121B*
UPF3B	*PAK3*	*TMEM127*
UPP2	*PALLD*	*TMEM129*
UQCC3	*PALM2-AKAP2*	*TMEM132B*
UQCR10	*PALMD*	*TMEM132C*
UQCR11	*PAM*	*TMEM135*
UQCRB	*PANK1*	*TMEM139*
UQCRFS1	*PANK2*	*TMEM140*
URB1	*PANK3*	*TMEM141*
URGCP-MRPS24	*PANK4*	*TMEM151A*
URI1	*PANX1*	*TMEM151B*
UROD	*PAOX*	*TMEM154*
UROS	*PAPLN*	*TMEM158*
USB1	*PAPOLA*	*TMEM164*
USF2	*PAPOLB*	*TMEM165*
USF3	*PAPOLG*	*TMEM167A*
USP12	*PAPPA*	*TMEM167B*
USP13	*PAPPA2*	*TMEM168*

USP14	*PAPSS2*	*TMEM169*
USP15	*PAQR3*	*TMEM170A*
USP18	*PAQR5*	*TMEM170B*
USP21	*PAQR7*	*TMEM178B*
USP22	*PAQR8*	*TMEM18*
USP3	*PAQR9*	*TMEM182*
USP30	*PARD3B*	*TMEM183A*
USP31	*PARD6B*	*TMEM184C*
USP32	*PARD6G*	*TMEM185B*
USP35	*PARD6G-AS1*	*TMEM186*
USP36	*PARK7*	*TMEM19*
USP39	*PARL*	*TMEM196*
USP4	*PARN*	*TMEM198*
USP40	*PARP1*	*TMEM199*
USP46	*PARP11*	*TMEM200C*
USP47	*PARP15*	*TMEM201*
USP48	*PARP16*	*TMEM207*
USP49	*PARP3*	*TMEM209*
USP5	*PARP4*	*TMEM211*
USP51	*PARP9*	*TMEM212*
USP53	*PARPBP*	*TMEM214*
USP54	*PARVA*	*TMEM215*
USP6	*PASK*	*TMEM220*
USP6NL	*PATE1*	*TMEM229A*
USP9X	*PATE2*	*TMEM229B*
USP9Y	*PATJ*	*TMEM230*
UST	*PATL1*	*TMEM231*
UTP14C	*PATZ1*	*TMEM233*
UTP15	*PAWR*	*TMEM235*
UTP25	*PAX1*	*TMEM236*
UTP6	*PAX3*	*TMEM237*
UTS2B	*PAX6*	*TMEM241*
UTY	*PAXBP1*	*TMEM245*
VAMP3	*PBK*	*TMEM246*
VAMP4	*PBOV1*	*TMEM248*
VAMP8	*PBRM1*	*TMEM25*
VANGL1	*PBX1*	*TMEM251*
VANGL2	*PBX3*	*TMEM253*
VAPA	*PCBP1*	*TMEM258*
VAPB	*PCCB*	*TMEM26*
VASH1	*PCDH10*	*TMEM263*
VASH2	*PCDH11X*	*TMEM33*
VAT1L	*PCDH11Y*	*TMEM39A*
VAV2	*PCDH12*	*TMEM41A*

VAV3	*PCDH15*	*TMEM43*
VAX1	*PCDH17*	*TMEM44*
VCAM1	*PCDH18*	*TMEM52B*
VCAN	*PCDH19*	*TMEM53*
VCL	*PCDH20*	*TMEM62*
VCP	*PCDH7*	*TMEM63C*
VCPIP1	*PCDH8*	*TMEM64*
VDAC3	*PCDH9*	*TMEM65*
VEZF1	*PCDHA9*	*TMEM74B*
VEZT	*PCDHAC1*	*TMEM86A*
VGLL3	*PCDHB10*	*TMEM87A*
VGLL4	*PCDHB11*	*TMEM87B*
VHL	*PCDHB12*	*TMEM92*
VHLL	*PCDHB15*	*TMEM9B*
VKORC1L1	*PCDHB17P*	*TMLHE*
VLDLR	*PCDHB3*	*TMOD2*
VMA21	*PCDHB4*	*TMOD3*
VMAC	*PCDHB5*	*TMPO*
VN1R1	*PCDHB7*	*TMPPE*
VPS13A	*PCDHGA1*	*TMPRSS4*
VPS13C	*PCDHGA10*	*TMSB15B*
VPS26C	*PCDHGA11*	*TMTC1*
VPS36	*PCDHGA12*	*TMTC2*
VPS37A	*PCDHGA2*	*TMTC3*
VPS50	*PCDHGA3*	*TMX4*
VPS52	*PCDHGA4*	*TNF*
VPS53	*PCDHGA5*	*TNFAIP1*
VPS54	*PCDHGA6*	*TNFAIP2*
VPS9D1	*PCDHGA7*	*TNFAIP8*
VSIR	*PCDHGA8*	*TNFAIP8L1*
VSTM4	*PCDHGA9*	*TNFRSF10C*
VSTM5	*PCDHGB1*	*TNFRSF11B*
VSX1	*PCDHGB2*	*TNFRSF13C*
VTA1	*PCDHGB3*	*TNFRSF14*
VTI1A	*PCDHGB4*	*TNFRSF19*
VTI1B	*PCDHGB6*	*TNFRSF1B*
VTN	*PCDHGB7*	*TNFRSF8*
VWA1	*PCDHGC3*	*TNFSF10*
VWA3B	*PCDHGC4*	*TNFSF12-TNFSF13*
VWC2	*PCDHGC5*	*TNFSF13*
VWC2L	*PCF11*	*TNFSF13B*
VWDE	*PCGF3*	*TNFSF14*
WAC	*PCGF5*	*TNFSF15*
WARS2	*PCGF6*	*TNFSF4*

WASF2	*PCID2*	*TNFSF8*
WASF3	*PCLO*	*TNIK*
WASL	*PCMTD2*	*TNIP1*
WBP1L	*PCNA*	*TNKS*
WDCP	*PCNP*	*TNKS2*
WDFY2	*PCNX1*	*TNN*
WDPCP	*PCNX2*	*TNNI1*
WDR12	*PCSK1*	*TNPO1*
WDR20	*PCSK2*	*TNR*
WDR33	*PCSK5*	*TNRC6A*
WDR35	*PCSK7*	*TNRC6B*
WDR4	*PCSK9*	*TNRC6C*
WDR41	*PCTP*	*TNS3*
WDR47	*PCYOX1*	*TNS4*
WDR48	*PCYT1B*	*TOB2*
WDR55	*PCYT2*	*TOGARAM2*
WDR59	*PDAP1*	*TOLLIP*
WDR6	*PDCD10*	*TOM1L1*
WDR61	*PDCD2*	*TOMM20*
WDR7	*PDCD4*	*TOMM34*
WDR70	*PDCD6*	*TOMM7*
WDR75	*PDCD6IP*	*TOMM70*
WDR77	*PDCD7*	*TOPBP1*
WDR82	*PDCL*	*TOR1AIP1*
WDR88	*PDE10A*	*TOR1AIP2*
WDR97	*PDE11A*	*TOR1B*
WDTC1	*PDE12*	*TOX*
WEE1	*PDE1A*	*TOX3*
WFDC1	*PDE1C*	*TP53BP2*
WFDC13	*PDE3A*	*TP53INP1*
WFS1	*PDE3B*	*TP53INP2*
WIF1	*PDE4B*	*TP63*
WIPF1	*PDE4D*	*TPCN2*
WIPF2	*PDE4DIP*	*TPD52*
WIPI2	*PDE5A*	*TPD52L1*
WLS	*PDE6A*	*TPD52L2*
WNK3	*PDE6D*	*TPGS2*
WNT1	*PDE6H*	*TPH1*
WNT10B	*PDE7A*	*TPK1*
WNT2	*PDE7B*	*TPM1*
WNT2B	*PDE8B*	*TPM3*
WNT7A	*PDE9A*	*TPPP3*
WNT8B	*PDF*	*TPRA1*
WNT9B	*PDGFC*	*TPRG1*

WRN	*PDGFD*	*TPRG1L*
WSB1	*PDGFRA*	*TPT1*
WSB2	*PDHA1*	*TRA2B*
WSCD1	*PDHB*	*TRABD2A*
WT1	*PDHX*	*TRABD2B*
WWC2	*PDIK1L*	*TRAF2*
WWC3	*PDK1*	*TRAF3IP1*
WWP2	*PDK4*	*TRAF3IP2*
WWTR1	*PDLIM3*	*TRAF4*
XBP1	*PDLIM5*	*TRAF5*
XCR1	*PDLIM7*	*TRAF6*
XIAP	*PDP1*	*TRAK1*
XIRP2	*PDP2*	*TRAM1*
XK	*PDPK1*	*TRAM2*
XKR4	*PDPR*	*TRAPPC10*
XKR7	*PDS5B*	*TRAPPC6B*
XPO5	*PDSS2*	*TRAPPC9*
XPO6	*PDX1*	*TRARG1*
XPR1	*PDXDC1*	*TRAT1*
XRCC3	*PDZD2*	*TRDMT1*
XRCC6	*PDZD8*	*TREM1*
XRN1	*PDZRN3*	*TREML2*
XYLT1	*PEAK1*	*TRIM14*
XYLT2	*PEAR1*	*TRIM2*
YAE1	*PEG10*	*TRIM24*
YAF2	*PELI1*	*TRIM28*
YAP1	*PELI2*	*TRIM35*
YARS2	*PELO*	*TRIM36*
YEATS2	*PER2*	*TRIM4*
YIPF3	*PER3*	*TRIM44*
YIPF4	*PERM1*	*TRIM48*
YIPF5	*PERP*	*TRIM58*
YIPF6	*PEX10*	*TRIM62*
YLPM1	*PEX11A*	*TRIM64B*
YOD1	*PEX11B*	*TRIM67*
YPEL1	*PEX13*	*TRIM71*
YPEL3	*PEX19*	*TRIM72*
YTHDC1	*PEX2*	*TRIM74*
YTHDF3	*PEX26*	*TRIM9*
YWHAE	*PEX3*	*TRIOBP*
YWHAG	*PEX5L*	*TRIP11*
YY1AP1	*PEX7*	*TRIP13*
ZADH2	*PF4*	*TRIP4*
ZBED1	*PF4V1*	*TRIQK*

ZBED4	*PFAS*	*TRMO*
ZBED6CL	*PFDN4*	*TRMT10B*
ZBTB16	*PFKFB2*	*TRMT2A*
ZBTB20	*PFKM*	*TRMT5*
ZBTB21	*PFN4*	*TRMT6*
ZBTB24	*PGAP1*	*TRMT9B*
ZBTB25	*PGAP2*	*TRNP1*
ZBTB3	*PGBD2*	*TROAP*
ZBTB33	*PGBD3*	*TRPA1*
ZBTB34	*PGBD4*	*TRPC1*
ZBTB37	*PGBD5*	*TRPC3*
ZBTB39	*PGGT1B*	*TRPC4*
ZBTB40	*PGK1*	*TRPC4AP*
ZBTB41	*PGLYRP2*	*TRPC5*
ZBTB43	*PGM1*	*TRPM2*
ZBTB49	*PGM2*	*TRPM3*
ZBTB5	*PGM2L1*	*TRPS1*
ZBTB6	*PGM3*	*TRPV2*
ZBTB7A	*PGM5*	*TRUB1*
ZBTB7C	*PGPEP1*	*TRUB2*
ZBTB8B	*PGPEP1L*	*TSC1*
ZBTB8OS	*PGR*	*TSC22D2*
ZBTB9	*PGRMC1*	*TSC22D3*
ZC2HC1C	*PHACTR1*	*TSEN2*
ZC3H11A	*PHACTR2*	*TSEN54*
ZC3H12B	*PHACTR4*	*TSFM*
ZC3H12C	*PHB*	*TSHZ2*
ZC3H13	*PHB2*	*TSKU*
ZC3H14	*PHC1*	*TSN*
ZC3H7B	*PHC3*	*TSNARE1*
ZC3H8	*PHEX*	*TSPAN11*
ZC3HAV1L	*PHF12*	*TSPAN12*
ZCCHC14	*PHF14*	*TSPAN13*
ZCCHC24	*PHF19*	*TSPAN14*
ZCCHC4	*PHF20*	*TSPAN15*
ZCRB1	*PHF20L1*	*TSPAN17*
ZDHHC14	*PHF21B*	*TSPAN2*
ZDHHC15	*PHF23*	*TSPAN3*
ZDHHC17	*PHF24*	*TSPAN5*
ZDHHC18	*PHF3*	*TSPAN7*
ZDHHC21	*PHF6*	*TSPYL1*
ZDHHC22	*PHF7*	*TSPYL5*
ZDHHC23	*PHF8*	*TSSK1B*
ZDHHC24	*PHIP*	*TSTD2*

ZDHHC3	*PHKA1*	*TTBK1*
ZDHHC8	*PHKA2*	*TTBK2*
ZDHHC9	*PHKG2*	*TTC13*
ZEB2	*PHLDA3*	*TTC14*
ZFAND1	*PHLDB1*	*TTC26*
ZFAND2A	*PHLDB2*	*TTC28*
ZFAND3	*PHLPP2*	*TTC30B*
ZFAND5	*PHOX2B*	*TTC31*
ZFHX2	*PHTF1*	*TTC37*
ZFHX3	*PHTF2*	*TTC39A*
ZFP1	*PHYH*	*TTC39B*
ZFP28	*PHYHIPL*	*TTC39C*
ZFP30	*PI15*	*TTC4*
ZFP36L2	*PI4K2A*	*TTC7A*
ZFP37	*PI4K2B*	*TTC7B*
ZFP62	*PI4KA*	*TTC8*
ZFP90	*PIAS1*	*TTC9*
ZFP91	*PIAS2*	*TTF2*
ZFPM2	*PIAS4*	*TTI2*
ZFYVE16	*PIBF1*	*TTL*
ZFYVE9	*PICALM*	*TTLL11*
ZHX1	*PID1*	*TTLL12*
ZIC1	*PIFO*	*TTLL4*
ZIC5	*PIGF*	*TTLL7*
ZIK1	*PIGG*	*TTPAL*
ZIM3	*PIGH*	*TTR*
ZKSCAN1	*PIGK*	*TUB*
ZKSCAN3	*PIGM*	*TUBA1A*
ZKSCAN4	*PIGO*	*TUBA1C*
ZKSCAN5	*PIGP*	*TUBB6*
ZKSCAN7	*PIGR*	*TUBGCP3*
ZKSCAN8	*PIGV*	*TUBGCP4*
ZMAT4	*PIH1D3*	*TULP4*
ZMIZ1	*PIK3AP1*	*TWF1*
ZMIZ2	*PIK3C2A*	*TWISTNB*
ZMPSTE24	*PIK3C3*	*TWNK*
ZMYM4	*PIK3CA*	*TXK*
ZMYND11	*PIK3CB*	*TXLNA*
ZNF100	*PIK3CG*	*TXLNB*
ZNF107	*PIK3R1*	*TXLNG*
ZNF114	*PIK3R2*	*TXNDC12*
ZNF124	*PIK3R3*	*TXNDC16*
ZNF131	*PIK3R5*	*TXNDC17*
ZNF132	*PIKFYVE*	*TXNRD1*

ZNF140	*PIM3*	*TXNRD2*
ZNF141	*PIN4*	*TYR*
ZNF146	*PINK1*	*TYRP1*
ZNF148	*PIP4K2A*	*TYW3*
ZNF154	*PIP4K2C*	*TYW5*
ZNF160	*PIP4P2*	*UAP1*
ZNF169	*PIP5K1A*	*UBA2*
ZNF174	*PIP5K1B*	*UBA5*
ZNF180	*PIP5K1C*	*UBA6*
ZNF181	*PITPNA*	*UBAC1*
ZNF185	*PITPNB*	*UBAP2L*
ZNF19	*PITPNC1*	*UBASH3B*
ZNF207	*PITPNM3*	*UBC*
ZNF211	*PITX2*	*UBD*
ZNF22	*PIWIL1*	*UBE2D1*
ZNF230	*PIWIL2*	*UBE2D3*
ZNF234	*PJA2*	*UBE2E1*
ZNF236	*PKD2*	*UBE2E2*
ZNF24	*PKHD1*	*UBE2E3*
ZNF25	*PKHD1L1*	*UBE2F*
ZNF253	*PKIA*	*UBE2G1*
ZNF256	*PKLR*	*UBE2G2*
ZNF260	*PKN2*	*UBE2H*
ZNF264	*PKNOX1*	*UBE2I*
ZNF268	*PKP4*	*UBE2J1*
ZNF274	*PLA2G12A*	*UBE2J2*
ZNF275	*PLA2G2C*	*UBE2K*
ZNF277	*PLA2G4A*	*UBE2N*
ZNF280C	*PLA2G4D*	*UBE2O*
ZNF280D	*PLA2G4E*	*UBE2Q1*
ZNF284	*PLA2G4F*	*UBE2QL1*
ZNF287	*PLA2G5*	*UBE2S*
ZNF3	*PLA2G7*	*UBE2T*
ZNF302	*PLA2R1*	*UBE2V2*
ZNF317	*PLAA*	*UBE2W*
ZNF326	*PLAAT3*	*UBE3B*
ZNF330	*PLAAT5*	*UBE4A*
ZNF333	*PLAC8*	*UBIAD1*
ZNF337	*PLAG1*	*UBN2*
ZNF341	*PLAGL2*	*UBOX5*
ZNF343	*PLAU*	*UBP1*
ZNF35	*PLB1*	*UBQLN4*
ZNF350	*PLBD2*	*UBR5*
ZNF354C	*PLCB1*	*UBR7*

ZNF362	*PLCB4*	*UBTD2*
ZNF367	*PLCD4*	*UBXN10*
ZNF37A	*PLCE1*	*UBXN2A*
ZNF385A	*PLCG1*	*UBXN2B*
ZNF385B	*PLCH1*	*UBXN7*
ZNF391	*PLCL1*	*UCHL5*
ZNF395	*PLCXD1*	*UCMA*
ZNF396	*PLCXD2*	*UCP2*
ZNF397	*PLCXD3*	*UCP3*
ZNF407	*PLD1*	*UEVLD*
ZNF410	*PLD2*	*UFL1*
ZNF417	*PLD5*	*UFM1*
ZNF426	*PLD6*	*UGCG*
ZNF429	*PLEKHA1*	*UGT2B15*
ZNF431	*PLEKHA3*	*UGT8*
ZNF436	*PLEKHA5*	*UHMK1*
ZNF436-AS1	*PLEKHA6*	*UHRF1*
ZNF446	*PLEKHA8*	*UHRF1BP1L*
ZNF451	*PLEKHB2*	*ULK2*
ZNF460	*PLEKHF2*	*ULK3*
ZNF461	*PLEKHG2*	*UMPS*
ZNF462	*PLEKHG3*	*UNC13A*
ZNF471	*PLEKHG4B*	*UNC13B*
ZNF483	*PLEKHG5*	*UNC5B*
ZNF490	*PLEKHH1*	*UNC5C*
ZNF496	*PLEKHH2*	*UNC5D*
ZNF500	*PLEKHM1*	*UNC79*
ZNF501	*PLEKHM3*	*UNC93A*
ZNF507	*PLEKHO1*	*UNKL*
ZNF512	*PLEKHS1*	*UPF1*
ZNF514	*PLG*	*UPF3B*
ZNF518B	*PLGLB1*	*UPP2*
ZNF530	*PLGLB2*	*UQCC3*
ZNF536	*PLIN3*	*UQCR10*
ZNF543	*PLIN5*	*UQCR11*
ZNF546	*PLK2*	*UQCRB*
ZNF547	*PLLP*	*UQCRFS1*
ZNF548	*PLN*	*URB1*
ZNF549	*PLP2*	*URGCP-MRPS24*
ZNF550	*PLPBP*	*URI1*
ZNF551	*PLPP3*	*UROS*
ZNF552	*PLPP4*	*USB1*
ZNF555	*PLPP5*	*USF3*
ZNF556	*PLPP6*	*USP1*

ZNF557	*PLPP7*	*USP13*
ZNF559-ZNF177	*PLPPR4*	*USP14*
ZNF560	*PLPPR5*	*USP15*
ZNF561	*PLRG1*	*USP18*
ZNF562	*PLS1*	*USP21*
ZNF572	*PLSCR1*	*USP22*
ZNF574	*PLSCR2*	*USP28*
ZNF576	*PLSCR5*	*USP3*
ZNF583	*PLXDC2*	*USP30*
ZNF584	*PLXNA2*	*USP31*
ZNF585A	*PLXNA4*	*USP32*
ZNF587B	*PLXNB1*	*USP35*
ZNF592	*PLXNC1*	*USP36*
ZNF593	*PM20D1*	*USP39*
ZNF605	*PM20D2*	*USP4*
ZNF607	*PMAIP1*	*USP40*
ZNF613	*PMEPA1*	*USP46*
ZNF614	*PMP2*	*USP47*
ZNF616	*PMPCA*	*USP48*
ZNF618	*PMPCB*	*USP49*
ZNF620	*PMVK*	*USP5*
ZNF621	*PNISR*	*USP51*
ZNF623	*PNLIPRP3*	*USP53*
ZNF624	*PNMA1*	*USP54*
ZNF625	*PNMA2*	*USP6*
ZNF626	*PNMA6A*	*USP6NL*
ZNF629	*PNMA8A*	*USP9X*
ZNF641	*PNN*	*USP9Y*
ZNF652	*PNO1*	*UST*
ZNF655	*PNOC*	*UTP14A*
ZNF660	*PNP*	*UTP14C*
ZNF668	*PNPLA3*	*UTP15*
ZNF670	*PNPLA4*	*UTP25*
ZNF680	*PNPLA8*	*UTP6*
ZNF695	*PNRC1*	*UTS2B*
ZNF697	*PNRC2*	*UTY*
ZNF699	*POC1B*	*VAMP3*
ZNF70	*POC1B-GALNT4*	*VAMP4*
ZNF701	*POF1B*	*VAMP7*
ZNF703	*POFUT1*	*VANGL1*
ZNF704	*POFUT2*	*VANGL2*
ZNF705A	*POGK*	*VAPB*
ZNF705D	*POGLUT1*	*VASH1*
ZNF705G	*POGLUT3*	*VASH2*

ZNF706	*POLA1*	*VAT1*
ZNF708	*POLA2*	*VAT1L*
ZNF709	*POLE*	*VAV2*
ZNF71	*POLE4*	*VAV3*
ZNF711	*POLG*	*VAX1*
ZNF714	*POLH*	*VCAM1*
ZNF736	*POLI*	*VCAN*
ZNF74	*POLK*	*VCL*
ZNF746	*POLM*	*VCPIP1*
ZNF747	*POLQ*	*VDAC3*
ZNF749	*POLR1A*	*VEZF1*
ZNF75A	*POLR1B*	*VEZT*
ZNF764	*POLR1C*	*VGLL3*
ZNF765	*POLR1E*	*VGLL4*
ZNF770	*POLR2C*	*VHL*
ZNF772	*POLR2D*	*VHLL*
ZNF773	*POLR2E*	*VIL1*
ZNF776	*POLR2F*	*VIT*
ZNF778	*POLR2M*	*VKORC1L1*
ZNF785	*POLR3A*	*VLDLR*
ZNF789	*POLR3B*	*VMA21*
ZNF791	*POLR3E*	*VMAC*
ZNF793	*POLR3F*	*VN1R1*
ZNF799	*POLR3G*	*VNN1*
ZNF805	*POLR3K*	*VPS13A*
ZNF814	*POM121*	*VPS13B*
ZNF831	*POM121L12*	*VPS13C*
ZNF844	*POMC*	*VPS26A*
ZNF85	*POMP*	*VPS26C*
ZNF852	*POMT1*	*VPS36*
ZNF875	*PON1*	*VPS37A*
ZNF879	*PON3*	*VPS50*
ZNF880	*POP1*	*VPS52*
ZNF891	*POP4*	*VPS53*
ZNF91	*POPDC2*	*VPS54*
ZNF93	*POSTN*	*VPS9D1*
ZNHIT6	*POT1*	*VRTN*
ZNRD2	*POTED*	*VSIR*
ZNRF1	*POTEE*	*VSTM4*
ZNRF2	*POTEI*	*VSTM5*
ZNRF3	*POU1F1*	*VSX1*
ZRANB1	*POU2AF1*	*VSX2*
ZSCAN12	*POU2F1*	*VTA1*
ZSCAN22	*POU2F2*	*VTI1A*

ZSCAN23	*POU3F2*	*VTI1B*
ZSCAN31	*POU3F3*	*VTN*
ZSWIM4	*POU4F1*	*VWA1*
ZSWIM7	*POU4F2*	*VWA3B*
ZW10	*POU6F2*	*VWC2*
ZXDA	*PP7080*	*VWC2L*
ZXDB	*PPA2*	*VWDE*
ZXDC	*PPARD*	*WAC*
ZYX	*PPARG*	*WARS*
ZZEF1	*PPARGC1A*	*WARS2*
ZZZ3	*PPARGC1B*	*WASF2*
	PPAT	*WASF3*
	PPBP	*WASL*
	PPDPF	*WBP1L*
	PPEF2	*WDCP*
	PPFIA1	*WDFY2*
	PPFIBP1	*WDHD1*
	PPHLN1	*WDPCP*
	PPIE	*WDR12*
	PPIF	*WDR20*
	PPIG	*WDR3*
	PPIL1	*WDR33*
	PPIL4	*WDR34*
	PPIL6	*WDR35*
	PPIP5K2	*WDR4*
	PPL	*WDR41*
	PPM1A	*WDR47*
	PPM1B	*WDR48*
	PPM1D	*WDR55*
	PPM1E	*WDR59*
	PPM1H	*WDR60*
	PPM1K	*WDR61*
	PPM1L	*WDR7*
	PPME1	*WDR70*
	PPP1CB	*WDR73*
	PPP1CC	*WDR75*
	PPP1R12A	*WDR76*
	PPP1R12B	*WDR77*
	PPP1R14C	*WDR82*
	PPP1R15A	*WDR89*
	PPP1R15B	*WDR97*
	PPP1R1A	*WDTC1*
	PPP1R1C	*WEE1*
	PPP1R21	*WFDC1*

PPP1R3A
PPP1R3B
PPP1R3C
PPP1R3D
PPP1R3E
PPP1R3G
PPP1R9A
PPP2CA
PPP2R1A
PPP2R1B
PPP2R2A
PPP2R2B
PPP2R2C
PPP2R3A
PPP2R5C
PPP2R5E
PPP3CB
PPP3CC
PPP3R1
PPP3R2
PPP4R1
PPP4R2
PPP4R3A
PPP4R4
PPP5D1
PPP6C
PPP6R1
PPP6R3
PPTC7
PPWD1
PQLC2
PQLC2L
PRAF2
PRAMEF1
PRAMEF15
PRAMEF2
PRAMEF27
PRAMEF4
PRAMEF6
PRAMEF7
PRAMEF8
PRC1
PRCP
PRDM1
WFDC13
WFS1
WIF1
WIPF1
WIPF2
WIPI2
WLS
WNK3
WNT1
WNT10B
WNT2
WNT2B
WNT8B
WNT9B
WRN
WSB1
WSB2
WSCD1
WT1
WWC2
WWC3
WWP2
WWTR1
XAF1
XBP1
XCR1
XIAP
XIRP2
XK
XKR4
XKR7
XPO5
XPR1
XRCC3
XRCC6
XRN1
XYLT2
YAE1
YAF2
YAP1
YARS2
YEATS2
YIPF3
YIPF4

PRDM10	*YIPF5*
PRDM13	*YIPF6*
PRDM15	*YME1L1*
PRDM16	*YOD1*
PRDM2	*YPEL1*
PRDM4	*YPEL2*
PRDM5	*YPEL3*
PRDM6	*YTHDC1*
PRDM7	*YTHDF2*
PRELID2	*YTHDF3*
PRELID3B	*YWHAB*
PRELP	*YWHAE*
PREPL	*YWHAG*
PREX1	*YWHAZ*
PREX2	*YY1*
PRH2	*YY2*
PRICKLE2	*ZADH2*
PRIM1	*ZBED1*
PRIMPOL	*ZBED4*
PRKAA1	*ZBED6CL*
PRKAA2	*ZBTB16*
PRKAB1	*ZBTB2*
PRKAB2	*ZBTB20*
PRKACA	*ZBTB21*
PRKACB	*ZBTB24*
PRKACG	*ZBTB25*
PRKAG2	*ZBTB26*
PRKAG3	*ZBTB3*
PRKAR1A	*ZBTB33*
PRKAR2A	*ZBTB34*
PRKAR2B	*ZBTB37*
PRKCA	*ZBTB39*
PRKCE	*ZBTB40*
PRKCI	*ZBTB41*
PRKCQ	*ZBTB42*
PRKDC	*ZBTB43*
PRKG1	*ZBTB49*
PRKG2	*ZBTB5*
PRKN	*ZBTB6*
PRKRA	*ZBTB7B*
PRKX	*ZBTB7C*
PRL	*ZBTB8B*
PRLHR	*ZBTB8OS*
PRMT2	*ZBTB9*

PRMT3	*ZC2HC1C*
PRMT5	*ZC3H10*
PRMT6	*ZC3H11A*
PRMT9	*ZC3H12B*
PRND	*ZC3H12C*
PRNP	*ZC3H13*
PROK2	*ZC3H14*
PROM2	*ZC3H15*
PROP1	*ZC3H4*
PRORP	*ZC3H7B*
PROS1	*ZC3H8*
PROSER1	*ZC3HAV1L*
PROSER2	*ZCCHC14*
PROSER3	*ZCCHC24*
PROX1	*ZCCHC4*
PROX2	*ZCRB1*
PRPF18	*ZDHHC14*
PRPF19	*ZDHHC15*
PRPF38A	*ZDHHC17*
PRPF38B	*ZDHHC18*
PRPF39	*ZDHHC2*
PRPF4	*ZDHHC20*
PRPF40A	*ZDHHC21*
PRPF4B	*ZDHHC22*
PRPH2	*ZDHHC23*
PRPS1	*ZDHHC24*
PRPS2	*ZDHHC3*
PRPSAP2	*ZDHHC8*
PRR14L	*ZDHHC9*
PRR15	*ZEB1*
PRR16	*ZEB2*
PRR20A	*ZFAND1*
PRR20B	*ZFAND2A*
PRR20C	*ZFAND3*
PRR20D	*ZFAND5*
PRR20E	*ZFHX2*
PRR23A	*ZFHX3*
PRR23B	*ZFP1*
PRR23D1	*ZFP14*
PRR23D2	*ZFP2*
PRR27	*ZFP28*
PRR29	*ZFP30*
PRR3	*ZFP36L2*
PRR5L	*ZFP37*

PRR9	*ZFP62*
PRRC1	*ZFP90*
PRRG4	*ZFP91*
PRRX1	*ZFPM2*
PRSS12	*ZFYVE16*
PRSS22	*ZFYVE27*
PRSS23	*ZHX1*
PRSS33	*ZIC1*
PRSS35	*ZIC5*
PRTG	*ZIK1*
PRUNE2	*ZIM3*
PRXL2B	*ZKSCAN1*
PRXL2C	*ZKSCAN3*
PSAPL1	*ZKSCAN4*
PSAT1	*ZKSCAN5*
PSD3	*ZKSCAN7*
PSD4	*ZKSCAN8*
PSEN1	*ZMAT4*
PSG1	*ZMIZ1*
PSG11	*ZMIZ2*
PSG2	*ZMPSTE24*
PSG3	*ZMYM4*
PSG4	*ZMYM6*
PSG5	*ZNF100*
PSG8	*ZNF107*
PSIP1	*ZNF112*
PSKH2	*ZNF117*
PSMA2	*ZNF124*
PSMA4	*ZNF132*
PSMA5	*ZNF134*
PSMA8	*ZNF136*
PSMB2	*ZNF138*
PSMC3IP	*ZNF140*
PSMD1	*ZNF141*
PSMD10	*ZNF146*
PSMD11	*ZNF148*
PSMD12	*ZNF154*
PSMD5	*ZNF160*
PSME4	*ZNF169*
PSMF1	*ZNF174*
PSMG1	*ZNF180*
PSMG4	*ZNF181*
PSPC1	*ZNF185*
PSPH	*ZNF19*

PSRC1	*ZNF195*
PTAFR	*ZNF207*
PTAR1	*ZNF208*
PTBP2	*ZNF211*
PTBP3	*ZNF22*
PTCD2	*ZNF224*
PTCD3	*ZNF233*
PTCH1	*ZNF234*
PTCHD1	*ZNF236*
PTCHD3	*ZNF24*
PTCHD4	*ZNF25*
PTDSS1	*ZNF253*
PTDSS2	*ZNF256*
PTEN	*ZNF260*
PTER	*ZNF264*
PTGDR	*ZNF268*
PTGER2	*ZNF274*
PTGER3	*ZNF275*
PTGER4	*ZNF277*
PTGES	*ZNF280C*
PTGES3	*ZNF280D*
PTGFR	*ZNF284*
PTGFRN	*ZNF287*
PTGIR	*ZNF3*
PTGIS	*ZNF302*
PTGR2	*ZNF317*
PTGS1	*ZNF319*
PTGS2	*ZNF326*
PTH2R	*ZNF330*
PTHLH	*ZNF331*
PTK2	*ZNF333*
PTPDC1	*ZNF337*
PTPMT1	*ZNF341*
PTPN1	*ZNF343*
PTPN11	*ZNF35*
PTPN14	*ZNF350*
PTPN2	*ZNF354C*
PTPN20	*ZNF362*
PTPN3	*ZNF366*
PTPN4	*ZNF367*
PTPRB	*ZNF37A*
PTPRC	*ZNF382*
PTPRD	*ZNF385A*
PTPRE	*ZNF385B*

PTPRG	*ZNF391*
PTPRJ	*ZNF395*
PTPRK	*ZNF396*
PTPRM	*ZNF397*
PTPRN2	*ZNF407*
PTPRO	*ZNF417*
PTPRT	*ZNF419*
PTPRZ1	*ZNF426*
PTRH2	*ZNF429*
PTRHD1	*ZNF431*
PTS	*ZNF436*
PTTG1IP	*ZNF436-AS1*
PUDP	*ZNF439*
PUM2	*ZNF440*
PURA	*ZNF446*
PURB	*ZNF451*
PURG	*ZNF460*
PUS10	*ZNF461*
PUS3	*ZNF471*
PUS7	*ZNF479*
PUS7L	*ZNF483*
PVRIG	*ZNF484*
PWP1	*ZNF490*
PWWP2A	*ZNF493*
PWWP3B	*ZNF496*
PXDN	*ZNF500*
PXK	*ZNF501*
PXMP4	*ZNF503*
PXN	*ZNF507*
PXT1	*ZNF514*
PXYLP1	*ZNF518B*
PYGO1	*ZNF525*
PYHIN1	*ZNF530*
PYROXD1	*ZNF536*
PYROXD2	*ZNF543*
QDPR	*ZNF546*
QKI	*ZNF547*
QPCT	*ZNF548*
QPCTL	*ZNF549*
QRFPR	*ZNF550*
QRSL1	*ZNF551*
QSER1	*ZNF552*
QSOX2	*ZNF555*
QTRT2	*ZNF556*

R3HDM1	*ZNF557*
RAB10	*ZNF559-ZNF177*
RAB11A	*ZNF560*
RAB11B	*ZNF561*
RAB11FIP1	*ZNF562*
RAB11FIP2	*ZNF571*
RAB12	*ZNF572*
RAB14	*ZNF576*
RAB15	*ZNF579*
RAB17	*ZNF583*
RAB18	*ZNF584*
RAB1A	*ZNF585A*
RAB21	*ZNF587*
RAB22A	*ZNF587B*
RAB23	*ZNF592*
RAB27A	*ZNF593*
RAB27B	*ZNF605*
RAB28	*ZNF607*
RAB29	*ZNF613*
RAB2A	*ZNF614*
RAB2B	*ZNF616*
RAB30	*ZNF618*
RAB31	*ZNF620*
RAB32	*ZNF621*
RAB33B	*ZNF623*
RAB38	*ZNF624*
RAB39B	*ZNF625*
RAB3B	*ZNF626*
RAB3C	*ZNF629*
RAB3GAP1	*ZNF641*
RAB3GAP2	*ZNF652*
RAB3IL1	*ZNF655*
RAB3IP	*ZNF660*
RAB41	*ZNF670*
RAB42	*ZNF672*
RAB43	*ZNF676*
RAB4A	*ZNF680*
RAB5IF	*ZNF695*
RAB6A	*ZNF697*
RAB6B	*ZNF699*
RAB6C	*ZNF70*
RAB7A	*ZNF701*
RAB8B	*ZNF703*
RAB9B	*ZNF704*

RABGAP1	*ZNF705A*
RABGAP1L	*ZNF705D*
RABGEF1	*ZNF705G*
RABIF	*ZNF706*
RABL2A	*ZNF708*
RABL2B	*ZNF709*
RABL3	*ZNF71*
RABL6	*ZNF716*
RAC1	*ZNF726*
RACGAP1	*ZNF728*
RAD1	*ZNF736*
RAD17	*ZNF737*
RAD18	*ZNF74*
RAD21	*ZNF746*
RAD21L1	*ZNF747*
RAD50	*ZNF749*
RAD51	*ZNF75A*
RAD51AP1	*ZNF764*
RAD51AP2	*ZNF765*
RAD51B	*ZNF772*
RAD51C	*ZNF773*
RAD52	*ZNF776*
RAD54B	*ZNF778*
RAD54L2	*ZNF785*
RAD9B	*ZNF789*
RADX	*ZNF791*
RAE1	*ZNF793*
RAG1	*ZNF799*
RAI14	*ZNF805*
RAI2	*ZNF814*
RALA	*ZNF827*
RALB	*ZNF831*
RALBP1	*ZNF844*
RALGAPA1	*ZNF85*
RALGAPA2	*ZNF850*
RALGPS1	*ZNF852*
RALGPS2	*ZNF875*
RALY	*ZNF879*
RALYL	*ZNF880*
RAMAC	*ZNF891*
RAN	*ZNF90*
RANBP1	*ZNF91*
RANBP2	*ZNF92*
RANBP3L	*ZNF93*

RANBP6
RANBP9
RANGAP1
RAP1A
RAP1GAP
RAP2A
RAP2B
RAP2C
RAPGEF1
RAPGEF2
RAPGEF3
RAPGEF5
RAPGEF6
RAPH1
RARB
RARRES1
RARS2
RASA1
RASA2
RASAL2
RASAL3
RASD2
RASEF
RASGEF1A
RASGRF1
RASGRF2
RASGRP3
RASSF1
RASSF2
RASSF3
RASSF4
RASSF6
RASSF8
RASSF9
RAVER2
RB1
RB1CC1
RBAK
RBBP4
RBBP5
RBBP6
RBBP9
RBFOX1
RBFOX2
ZNF99
ZNHIT6
ZNRF1
ZNRF2
ZNRF3
ZRANB1
ZSCAN12
ZSCAN16
ZSCAN18
ZSCAN22
ZSCAN31
ZSWIM4
ZSWIM7
ZXDA
ZXDB
ZXDC
ZYG11B
ZYX
ZZEF1
ZZZ3

RBL1
RBL2
RBM11
RBM12
RBM12B
RBM14
RBM17
RBM18
RBM19
RBM20
RBM22
RBM24
RBM25
RBM26
RBM27
RBM28
RBM3
RBM38
RBM39
RBM4
RBM41
RBM43
RBM44
RBM46
RBM48
RBM7
RBMS1
RBMS2
RBMXL1
RBMXL2
RBMY1A1
RBMY1B
RBMY1D
RBMY1E
RBMY1F
RBP2
RBPMS
RBPMS2
RBSN
RC3H2
RCAN2
RCAN3
RCBTB1
RCBTB2

RCC2
RCCD1
RCHY1
RCL1
RCN1
RCN2
RCOR1
RCOR3
RCSD1
RD3
RDH10
RDH12
RDH13
RDH14
RDX
RECK
RECQL
REEP1
REEP3
REEP5
REG3A
REG3G
REG4
REL
RELCH
RELL1
RELT
REP15
REPS1
REPS2
RERE
RERG
RESF1
REST
RET
RETREG1
RETREG2
REV1
RFC1
RFC3
RFLNB
RFNG
RFPL3S
RFPL4B

RFTN2
RFX2
RFX3
RFX5
RFX6
RGL1
RGL2
RGMA
RGMB
RGN
RGPD4
RGPD5
RGPD6
RGPD8
RGR
RGS13
RGS16
RGS17
RGS18
RGS2
RGS21
RGS4
RGS5
RGS6
RGS7
RGS7BP
RGS9BP
RHBDD1
RHEB
RHOBTB1
RHOBTB2
RHOBTB3
RHOH
RHOJ
RHOQ
RHOT1
RHPN2
RIBC1
RIC1
RIC8B
RICTOR
RIDA
RILPL2
RIMBP2

RIMKLA
RIMKLB
RIMS2
RIMS3
RIMS4
RIN2
RINT1
RIOK1
RIOK2
RIOK3
RIOX2
RIPK2
RIPK4
RIPOR2
RIPOR3
RIPPLY3
RIT1
RITA1
RLBP1
RLIM
RLN1
RLN2
RMDN2
RMI1
RMI2
RMND5A
RNASE13
RNASE4
RNASEH1
RNASEH2B
RNASEH2C
RNASET2
RND3
RNF103-CHMP3
RNF11
RNF111
RNF114
RNF125
RNF126
RNF13
RNF130
RNF138
RNF141
RNF144A

RNF144B
RNF146
RNF148
RNF149
RNF150
RNF152
RNF157
RNF165
RNF168
RNF169
RNF17
RNF170
RNF180
RNF182
RNF185
RNF187
RNF19A
RNF19B
RNF2
RNF20
RNF207
RNF212
RNF212B
RNF213
RNF216
RNF217
RNF219
RNF26
RNF32
RNF34
RNF38
RNF4
RNF41
RNF44
RNGTT
RNLS
RNMT
RO60
ROBO1
ROBO2
ROBO3
ROBO4
ROCK1
ROCK2

ROMO1
ROPN1
ROR1
ROR2
RORA
RORB
RORC
ROS1
RP2
RPA1
RPA3
RPAIN
RPAP2
RPAP3
RPE
RPE65
RPEL1
RPF1
RPF2
RPGR
RPGRIP1L
RPH3AL
RPL10L
RPL12
RPL13
RPL13A
RPL14
RPL17
RPL22
RPL22L1
RPL23
RPL23A
RPL26L1
RPL27A
RPL31
RPL36AL
RPL37
RPL37A
RPL7
RPL7L1
RPL8
RPL9
RPP14
RPP30

RPP40
RPRD1A
RPRD2
RPS12
RPS15A
RPS19
RPS2
RPS20
RPS23
RPS27A
RPS27L
RPS29
RPS4Y1
RPS6
RPS6KA2
RPS6KA3
RPS6KA5
RPS6KA6
RPS6KB1
RPS6KC1
RPS6KL1
RPTN
RPUSD2
RRAGC
RRAGD
RRAS2
RREB1
RRH
RRM1
RRM2B
RRN3
RRP15
RRP1B
RRP36
RRP7A
RRP8
RRS1
RS1
RSAD2
RSBN1
RSBN1L
RSF1
RSKR
RSL1D1

RSPH3
RSPH4A
RSPO1
RSPO3
RSPRY1
RTBDN
RTCA
RTF2
RTKN2
RTL6
RTL8B
RTL9
RTN3
RTN4
RTN4RL1
RTP4
RUBCN
RUBCNL
RUFY1
RUFY2
RUFY3
RUNDC1
RUNDC3B
RUNX1
RUNX1T1
RUNX2
RWDD1
RWDD2A
RWDD2B
RWDD4
RXFP1
RXYLT1
RYBP
RYR2
S100A10
S100A16
S100A2
S100A7A
S100PBP
S1PR1
S1PR3
S1PR5
SAA2
SACM1L

SAE1
SALL1
SALL4
SAMD1
SAMD12
SAMD14
SAMD15
SAMD3
SAMD4A
SAMD5
SAMD8
SAMD9L
SAP30
SAP30BP
SAP30L
SAR1A
SAR1B
SARAF
SART3
SASH1
SASS6
SATB1
SATB2
SAV1
SAXO2
SAYSD1
SBF2
SBSPON
SC5D
SCAF1
SCAF4
SCAF8
SCAI
SCAMP5
SCAPER
SCARA5
SCARB1
SCARB2
SCCPDH
SCD5
SCEL
SCFD1
SCFD2
SCG5

SCGB2A1
SCIMP
SCIN
SCLT1
SCML1
SCML2
SCML4
SCN11A
SCN1A
SCN2A
SCN2B
SCN3A
SCN8A
SCN9A
SCNM1
SCO1
SCP2
SCRG1
SCRIB
SCRN1
SCRN2
SCRN3
SCUBE3
SCYL1
SCYL2
SCYL3
SDAD1
SDC1
SDC2
SDCBP
SDE2
SDF2
SDHAF1
SDHAF3
SDHD
SDK1
SDR16C5
SEC11A
SEC14L3
SEC14L4
SEC14L5
SEC14L6
SEC16B
SEC22C

SEC23IP
SEC24A
SEC24B
SEC24D
SEC31B
SEC61A2
SEC61G
SEC62
SEC63
SECISBP2L
SEH1L
SEL1L
SEL1L3
SELENOI
SELENOK
SELENON
SELENOP
SELENOS
SELENOT
SEM1
SEMA3A
SEMA3C
SEMA3D
SEMA4D
SEMA4G
SEMA5A
SEMA6A
SEMA6D
SEMA7A
SENP2
SENP5
SENP7
SEPHS1
SEPTIN10
SEPTIN11
SEPTIN14
SEPTIN2
SEPTIN3
SEPTIN4
SEPTIN6
SEPTIN7
SERAC1
SERBP1
SERF1A

SERF1B
SERINC1
SERINC3
SERP1
SERPINA5
SERPINB10
SERPINB11
SERPINB13
SERPINB2
SERPINB7
SERPINB8
SERPINB9
SERPINC1
SERPINE1
SERPING1
SERTAD2
SERTAD3
SERTAD4
SERTM1
SESN1
SESN2
SESN3
SESTD1
SETBP1
SETD3
SETD4
SETD5
SETD7
SETD9
SETDB2
SETX
SEZ6L
SF3A1
SF3B1
SF3B3
SF3B5
SFI1
SFMBT1
SFMBT2
SFPQ
SFRP1
SFRP2
SFRP4
SFT2D2

SFT2D3
SFTPA1
SFTPA2
SFXN1
SFXN2
SFXN5
SGCB
SGCD
SGMS2
SGO1
SGPL1
SGPP2
SGSM2
SGTA
SGTB
SH2B3
SH2D1A
SH2D1B
SH2D4A
SH2D4B
SH2D7
SH3BGR
SH3BGRL
SH3BGRL2
SH3BP2
SH3BP4
SH3BP5
SH3BP5L
SH3D19
SH3GL2
SH3GLB1
SH3GLB2
SH3KBP1
SH3PXD2B
SH3RF1
SH3RF2
SH3RF3
SH3YL1
SHC1
SHC3
SHC4
SHCBP1
SHCBP1L
SHE

SHH
SHISA2
SHISA3
SHISA6
SHISA7
SHISA9
SHISAL1
SHLD1
SHLD2
SHMT1
SHOC2
SHPRH
SHQ1
SHROOM2
SHROOM3
SHROOM4
SHTN1
SIAH2
SIAH3
SIDT1
SIDT2
SIGLEC11
SIGLEC12
SIGLEC15
SIGLEC8
SIGLEC9
SIK1
SIK2
SIKE1
SIM1
SIM2
SIN3A
SIPA1
SIPA1L1
SIRPA
SIRPB1
SIRPB2
SIRT1
SIRT3
SIRT5
SIRT7
SIT1
SIX1
SIX3

SIX4
SIX6
SKA1
SKA2
SKA3
SKAP2
SKI
SKIDA1
SKIL
SKP1
SKP2
SLA
SLA2
SLAIN1
SLAIN2
SLAMF6
SLBP
SLC10A1
SLC10A2
SLC10A3
SLC10A6
SLC10A7
SLC11A1
SLC12A2
SLC12A3
SLC12A5
SLC12A6
SLC12A7
SLC13A1
SLC13A3
SLC14A1
SLC15A2
SLC15A4
SLC15A5
SLC16A1
SLC16A13
SLC16A14
SLC16A2
SLC16A6
SLC16A7
SLC16A9
SLC17A2
SLC17A4
SLC17A6

SLC17A8
SLC17A9
SLC18A1
SLC18A2
SLC18B1
SLC19A2
SLC1A1
SLC1A2
SLC1A3
SLC1A4
SLC1A5
SLC1A6
SLC1A7
SLC20A2
SLC22A1
SLC22A10
SLC22A15
SLC22A2
SLC22A9
SLC23A1
SLC23A2
SLC24A1
SLC24A2
SLC24A4
SLC24A5
SLC25A10
SLC25A12
SLC25A15
SLC25A16
SLC25A17
SLC25A18
SLC25A21
SLC25A21-AS1
SLC25A23
SLC25A24
SLC25A25
SLC25A26
SLC25A27
SLC25A30
SLC25A31
SLC25A32
SLC25A36
SLC25A37
SLC25A38

SLC25A4
SLC25A40
SLC25A44
SLC25A46
SLC25A5
SLC26A1
SLC26A10
SLC26A2
SLC26A4
SLC26A7
SLC26A8
SLC26A9
SLC27A4
SLC28A1
SLC28A3
SLC29A4
SLC2A1
SLC2A10
SLC2A12
SLC2A13
SLC2A14
SLC2A2
SLC2A3
SLC2A9
SLC30A1
SLC30A10
SLC30A2
SLC30A4
SLC30A5
SLC30A6
SLC30A7
SLC30A8
SLC30A9
SLC31A1
SLC31A2
SLC32A1
SLC34A1
SLC35A1
SLC35A5
SLC35B3
SLC35B4
SLC35C2
SLC35D1
SLC35D2

SLC35E1
SLC35E3
SLC35F1
SLC35F2
SLC35F3
SLC35F5
SLC35F6
SLC35G1
SLC35G3
SLC36A1
SLC36A2
SLC36A4
SLC37A3
SLC38A1
SLC38A11
SLC38A2
SLC38A4
SLC38A7
SLC38A9
SLC39A1
SLC39A11
SLC39A14
SLC39A2
SLC39A6
SLC39A8
SLC39A9
SLC3A1
SLC41A2
SLC41A3
SLC43A2
SLC43A3
SLC44A1
SLC44A5
SLC45A4
SLC46A3
SLC49A4
SLC4A1
SLC4A10
SLC4A4
SLC4A5
SLC4A7
SLC4A8
SLC5A1
SLC5A10

SLC5A12
SLC5A3
SLC5A6
SLC5A7
SLC5A8
SLC6A1
SLC6A11
SLC6A15
SLC6A17
SLC6A20
SLC6A5
SLC6A8
SLC7A1
SLC7A11
SLC7A14
SLC7A2
SLC7A6
SLC7A6OS
SLC8A1
SLC8B1
SLC9A4
SLC9A6
SLC9A7
SLC9A8
SLC9A9
SLC9B1
SLC9B2
SLC9C1
SLCO1B1
SLCO1C1
SLCO2B1
SLCO3A1
SLCO4C1
SLCO5A1
SLF1
SLF2
SLFN11
SLFN12L
SLFN13
SLFN5
SLIT1
SLIT2
SLITRK1
SLITRK3

SLK
SLMAP
SLU7
SLX4IP
SMAD1
SMAD2
SMAD3
SMAD4
SMAD5
SMAD7
SMAD9
SMAP1
SMAP2
SMARCA1
SMARCA2
SMARCA5
SMARCAD1
SMARCC1
SMARCE1
SMC1B
SMC2
SMC3
SMC4
SMC5
SMC6
SMCHD1
SMCO3
SMCR8
SMDT1
SMG1
SMG5
SMG7
SMIM12
SMIM13
SMIM14
SMIM15
SMIM18
SMIM19
SMIM21
SMIM30
SMIM6
SMIM7
SMIM8
SMKR1

SMLR1
SMN1
SMN2
SMNDC1
SMO
SMR3A
SMS
SMTNL2
SMU1
SMUG1
SMURF1
SMURF2
SMYD2
SNAI1
SNAI2
SNAP23
SNAP25
SNAP29
SNAP47
SNAP91
SNAPC1
SNAPC3
SNAPC5
SNAPIN
SNCAIP
SND1
SNED1
SNF8
SNIP1
SNN
SNRK
SNRNP27
SNRNP40
SNRNP48
SNRPD1
SNRPD3
SNRPF
SNRPG
SNTB2
SNX1
SNX10
SNX13
SNX16
SNX18

SNX19
SNX2
SNX22
SNX24
SNX25
SNX27
SNX30
SNX4
SNX9
SOAT1
SOBP
SOCS2
SOCS3
SOCS4
SOCS5
SOCS6
SOD2
SOGA1
SOHLH1
SOHLH2
SON
SORBS1
SORBS2
SORCS1
SORCS2
SORD
SORL1
SORT1
SOS2
SOST
SOSTDC1
SOWAHA
SOWAHB
SOWAHC
SOX1
SOX11
SOX12
SOX13
SOX21
SOX30
SOX4
SOX5
SOX7
SP1

SP100
SP110
SP140L
SP2
SP3
SP4
SP8
SP9
SPA17
SPACA1
SPAG1
SPAG11A
SPAG16
SPAG6
SPAG9
SPAM1
SPANXN1
SPARC
SPARCL1
SPATA13
SPATA17
SPATA18
SPATA2
SPATA22
SPATA33
SPATA4
SPATA5
SPATA6
SPATA9
SPATS2
SPATS2L
SPC24
SPC25
SPCS1
SPCS2
SPCS3
SPDYE1
SPDYE3
SPECC1
SPEF2
SPEN
SPESP1
SPG21
SPG7

SPHAR
SPHKAP
SPIC
SPICE1
SPIDR
SPIN1
SPIN3
SPIN4
SPINT2
SPIRE1
SPOCK1
SPOCK2
SPOCK3
SPOPL
SPOUT1
SPPL2A
SPPL2C
SPRED1
SPRED2
SPRY1
SPRY3
SPRY4
SPRYD4
SPRYD7
SPTBN1
SPTBN2
SPTLC1
SPTLC2
SPTLC3
SPTSSA
SPTY2D1
SPX
SRA1
SRC
SRCAP
SRD5A1
SRD5A3
SREK1
SREK1IP1
SRFBP1
SRGAP1
SRGAP3
SRGN
SRI

SRL
SRP19
SRP9
SRPK2
SRPRA
SRPRB
SRPX2
SRR
SRRD
SRRM1
SRRM4
SRSF1
SRSF10
SRSF11
SRSF12
SRSF2
SRSF3
SRSF4
SRSF6
SRSF7
SRSF9
SRXN1
SRY
SS18
SS18L1
SSB
SSBP2
SSBP3
SSH2
SSPN
SSR1
SSR3
SSR4P1
SSTR2
SSX1
SSX2
SSX2IP
SSX3
ST13
ST18
ST3GAL6
ST6GAL1
ST6GAL2
ST6GALNAC3

ST6GALNAC5
ST7L
ST8SIA1
ST8SIA2
ST8SIA3
ST8SIA4
ST8SIA6
STAC
STAC2
STAG1
STAG2
STAM
STAM2
STAMBP
STAMBPL1
STAP1
STARD13
STARD3NL
STARD4
STARD7
STARD9
STAT1
STAT2
STAT3
STAT5B
STATH
STAU1
STAU2
STC1
STC2
STEAP1
STEAP2
STEAP3
STEAP4
STIM1
STIM2
STK10
STK16
STK17B
STK24
STK26
STK3
STK32A
STK32B

STK32C
STK35
STK38L
STK39
STK40
STKLD1
STMN2
STMND1
STMP1
STN1
STOM
STOML1
STOML3
STON2
STOX2
STRADA
STRADB
STRIP1
STRIP2
STRN
STRN3
STRN4
STS
STT3A
STT3B
STUM
STX11
STX16
STX17
STX1A
STX1B
STX2
STX3
STX6
STX7
STXBP1
STXBP3
STXBP4
STXBP5
STXBP5L
STXBP6
STYK1
STYX
STYXL1

SUB1
SUCLG1
SUCLG2
SUCNR1
SUCO
SUFU
SUGCT
SUGT1
SULF1
SULF2
SULT1B1
SULT1C2
SULT1C4
SULT1E1
SULT4A1
SUMO1
SUMO2
SUMO3
SUOX
SUPT3H
SUPT7L
SURF1
SURF4
SURF6
SUSD1
SUSD5
SUSD6
SUV39H2
SUZ12
SV2A
SV2B
SV2C
SVIP
SWAP70
SWT1
SYAP1
SYCP2
SYF2
SYK
SYN1
SYN3
SYNC
SYNCRIP
SYNDIG1

SYNE3
SYNGR3
SYNJ1
SYNJ2
SYNJ2BP
SYNM
SYNPO
SYNPO2
SYNPO2L
SYNPR
SYPL1
SYS1
SYT1
SYT10
SYT11
SYT13
SYT15
SYT2
SYT4
SYT5
SYT6
SYT7
SYT9
SYTL3
SZRD1
TAB1
TAB3
TAC1
TACO1
TACR1
TACR3
TADA1
TADA2B
TAF11
TAF1A
TAF1B
TAF1D
TAF2
TAF3
TAF4B
TAF5
TAF5L
TAF7
TAF8

TAF9B
TAFA1
TAFA2
TAFA3
TAFA4
TAGAP
TAL1
TAL2
TANC1
TANC2
TANGO6
TANK
TAOK1
TAOK3
TAP2
TAPT1
TARDBP
TARSL2
TAS2R20
TAS2R30
TAS2R4
TAS2R5
TASOR
TASOR2
TASP1
TAT
TATDN1
TATDN2
TATDN3
TAX1BP1
TBC1D1
TBC1D12
TBC1D14
TBC1D15
TBC1D16
TBC1D19
TBC1D20
TBC1D22A
TBC1D23
TBC1D24
TBC1D25
TBC1D30
TBC1D32
TBC1D4

TBC1D5
TBC1D8
TBC1D8B
TBC1D9
TBCA
TBCC
TBCEL
TBK1
TBL1X
TBL1XR1
TBPL1
TBPL2
TBRG1
TBX15
TBX18
TBX20
TBX3
TBX5
TBX6
TC2N
TCAF1
TCAF2
TCEA1
TCEAL4
TCEANC
TCEANC2
TCF12
TCF15
TCF21
TCF23
TCF24
TCF4
TCF7
TCF7L2
TCFL5
TCHHL1
TCHP
TCIM
TCIRG1
TCL1A
TCN2
TCOF1
TCP1
TCP11L2

TCTA
TCTE1
TCTEX1D1
TCTN1
TDG
TDGF1
TDO2
TDP2
TDRD15
TDRD6
TDRP
TEC
TECPR1
TECPR2
TECRL
TECTB
TEDDM1
TEF
TEFM
TEKT1
TENM1
TENM2
TENM3
TENM4
TENT2
TENT4A
TENT5C
TENT5D
TEP1
TEPP
TEPSIN
TERB2
TERF1
TERF2
TERF2IP
TES
TESK1
TESMIN
TESPA1
TET1
TET2
TET3
TEX19
TEX2

TEX22
TEX35
TEX36
TF
TFAM
TFAP2B
TFAP2C
TFB2M
TFCP2L1
TFDP1
TFDP2
TFE3
TFEC
TFF3
TFG
TFIP11
TFPI
TFPI2
TGFA
TGFB2
TGFB3
TGFBI
TGFBR1
TGFBR2
TGFBR3
TGIF1
TGIF2LX
TGIF2LY
TGIF2-RAB5IF
TGM2
TGM4
TGM5
TGOLN2
TGS1
THADA
THAP1
THAP12
THAP2
THAP3
THAP6
THAP9
THBD
THBS1
THBS2

THBS4
THEM4
THEMIS2
THG1L
THNSL1
THOC3
THOC5
THOC7
THRAP3
THRB
THSD4
THSD7A
THSD7B
THTPA
THUMPD2
THUMPD3
TIA1
TIAL1
TIFA
TIFAB
TIGAR
TIGD2
TIGD4
TIGD6
TIGIT
TIMD4
TIMELESS
TIMM10
TIMM17A
TIMM29
TIMM8A
TIMM9
TIMP3
TIMP4
TIPRL
TIRAP
TJP2
TK2
TKFC
TKTL1
TLCD2
TLCD4
TLCD5
TLE3

TLE4
TLK1
TLK2
TLL1
TLL2
TLN2
TLNRD1
TLR3
TLR6
TLR7
TLX1
TLX3
TM2D2
TM2D3
TM4SF1
TM4SF18
TM4SF20
TM4SF5
TM6SF1
TM9SF2
TM9SF3
TMA16
TMA7
TMBIM4
TMBIM6
TMC2
TMC6
TMC7
TMCC1
TMCC3
TMCO1
TMCO3
TMED10
TMED2
TMED4
TMED5
TMED7
TMED8
TMED9
TMEFF1
TMEFF2
TMEM100
TMEM104
TMEM106B

TMEM106C
TMEM108
TMEM11
TMEM116
TMEM117
TMEM120B
TMEM121B
TMEM127
TMEM128
TMEM130
TMEM132A
TMEM132B
TMEM134
TMEM135
TMEM144
TMEM14B
TMEM151A
TMEM151B
TMEM154
TMEM156
TMEM161B
TMEM163
TMEM164
TMEM165
TMEM167A
TMEM167B
TMEM168
TMEM169
TMEM170A
TMEM170B
TMEM174
TMEM175
TMEM178A
TMEM178B
TMEM181
TMEM182
TMEM183A
TMEM184A
TMEM184B
TMEM184C
TMEM185A
TMEM185B
TMEM186
TMEM189

TMEM19
TMEM192
TMEM196
TMEM200A
TMEM200C
TMEM207
TMEM209
TMEM213
TMEM215
TMEM220
TMEM221
TMEM229A
TMEM230
TMEM231
TMEM232
TMEM233
TMEM234
TMEM236
TMEM237
TMEM239
TMEM241
TMEM242
TMEM243
TMEM245
TMEM246
TMEM248
TMEM25
TMEM251
TMEM252
TMEM254
TMEM255A
TMEM26
TMEM260
TMEM263
TMEM267
TMEM273
TMEM30A
TMEM30B
TMEM33
TMEM35A
TMEM37
TMEM38A
TMEM39A
TMEM40

TMEM41A
TMEM43
TMEM44
TMEM47
TMEM50A
TMEM50B
TMEM53
TMEM62
TMEM63C
TMEM64
TMEM65
TMEM67
TMEM68
TMEM69
TMEM70
TMEM72
TMEM74
TMEM86A
TMEM87A
TMEM87B
TMEM97
TMEM98
TMEM9B
TMF1
TMIE
TMLHE
TMOD1
TMOD2
TMOD3
TMPO
TMPPE
TMPRSS11A
TMPRSS11B
TMPRSS11D
TMPRSS11E
TMPRSS12
TMPRSS13
TMPRSS15
TMPRSS2
TMPRSS4
TMPRSS5
TMSB15B
TMTC1
TMTC2

TMTC3
TMTC4
TMX1
TMX3
TMX4
TNF
TNFAIP6
TNFAIP8
TNFAIP8L1
TNFAIP8L3
TNFRSF10A
TNFRSF10B
TNFRSF10C
TNFRSF11A
TNFRSF11B
TNFRSF13C
TNFRSF14
TNFRSF19
TNFRSF1B
TNFRSF25
TNFRSF8
TNFRSF9
TNFSF10
TNFSF13B
TNFSF14
TNFSF15
TNFSF4
TNFSF8
TNIK
TNIP2
TNIP3
TNKS
TNKS1BP1
TNKS2
TNN
TNNI1
TNPO1
TNR
TNRC6A
TNRC6B
TNRC6C
TNS3
TNS4
TOGARAM1

TOGARAM2
TOM1L1
TOMM20
TOMM20L
TOMM22
TOMM40
TOMM6
TOP1
TOP2A
TOPORS
TOR1A
TOR1AIP1
TOR1AIP2
TOR1B
TOX
TOX2
TOX3
TP53
TP53BP2
TP53I11
TP53INP1
TP53INP2
TP53TG5
TP63
TPBGL
TPCN1
TPCN2
TPD52
TPD52L1
TPD52L3
TPGS2
TPH1
TPH2
TPK1
TPM3
TPMT
TPP1
TPPP
TPR
TPRG1
TPRG1L
TPRX1
TPRXL
TPST1

TPST2
TPT1
TRA2B
TRABD
TRABD2A
TRABD2B
TRAF1
TRAF2
TRAF3
TRAF3IP1
TRAF3IP3
TRAF4
TRAF5
TRAF6
TRAK1
TRAK2
TRAM2
TRAPPC10
TRAPPC13
TRAPPC2B
TRAPPC4
TRAPPC6B
TRAPPC8
TRAPPC9
TRAT1
TRDMT1
TRDN
TREM1
TREML2
TRERF1
TRHDE
TRIAP1
TRIB1
TRIM13
TRIM14
TRIM2
TRIM22
TRIM23
TRIM24
TRIM25
TRIM29
TRIM33
TRIM35
TRIM36

TRIM37
TRIM38
TRIM4
TRIM40
TRIM41
TRIM44
TRIM45
TRIM5
TRIM58
TRIM6
TRIM63
TRIM64B
TRIM65
TRIM66
TRIM67
TRIM7
TRIM71
TRIM72
TRIML2
TRIO
TRIP11
TRIP12
TRIP13
TRIP4
TRIQK
TRMO
TRMT10A
TRMT10B
TRMT10C
TRMT12
TRMT13
TRMT1L
TRMT44
TRMT5
TRMT9B
TRMU
TRNT1
TRPA1
TRPC3
TRPC4
TRPC5
TRPC6
TRPM1
TRPM3

TRPM4
TRPM6
TRPM7
TRPS1
TRPV1
TRPV3
TRUB1
TSC22D1
TSC22D2
TSC22D3
TSEN2
TSFM
TSHR
TSHZ1
TSHZ2
TSHZ3
TSKU
TSLP
TSN
TSNAX
TSPAN11
TSPAN12
TSPAN14
TSPAN15
TSPAN2
TSPAN3
TSPAN5
TSPAN6
TSPAN9
TSPYL1
TSPYL6
TSR1
TSTD2
TTBK2
TTC14
TTC17
TTC19
TTC21B
TTC22
TTC23
TTC26
TTC28
TTC29
TTC30A

TTC30B
TTC31
TTC32
TTC33
TTC37
TTC39A
TTC39B
TTC4
TTC5
TTC7A
TTC8
TTC9
TTF1
TTF2
TTK
TTL
TTLL11
TTLL5
TTLL7
TTLL9
TTN
TTPA
TTPAL
TTR
TUB
TUBA1B
TUBB1
TUBB6
TUBE1
TUBGCP3
TUFT1
TULP3
TULP4
TUSC1
TUSC2
TUSC3
TUT4
TUT7
TVP23A
TVP23C
TWF1
TWIST1
TWISTNB
TWSG1

TXK
TXLNA
TXLNG
TXNDC15
TXNDC16
TXNDC2
TXNDC8
TXNDC9
TXNL1
TXNL4B
TXNRD1
TXNRD2
TYMS
TYRO3
TYRP1
TYW1
TYW3
TYW5
UACA
UAP1
UBA2
UBA5
UBA6
UBAP2L
UBASH3B
UBB
UBD
UBE2A
UBE2B
UBE2D1
UBE2D2
UBE2D3
UBE2D4
UBE2E3
UBE2G1
UBE2H
UBE2I
UBE2J1
UBE2K
UBE2L3
UBE2N
UBE2Q1
UBE2Q2
UBE2QL1

UBE2R2
UBE2S
UBE2T
UBE2U
UBE2V2
UBE2W
UBE3A
UBE3B
UBE3D
UBE4A
UBE4B
UBFD1
UBIAD1
UBL3
UBL4A
UBLCP1
UBN2
UBP1
UBQLN1
UBQLN4
UBR1
UBR2
UBR5
UBR7
UBTD2
UBXN10
UBXN2A
UBXN2B
UBXN4
UBXN7
UCHL3
UCHL5
UCK1
UCK2
UCKL1
UCP1
UCP3
UEVLD
UFD1
UFL1
UFM1
UGCG
UGDH
UGGT1

UGP2
UGT2A1
UGT2A3
UGT2B10
UGT2B11
UGT2B15
UGT2B4
UGT3A1
UGT8
UHMK1
UHRF1BP1
UHRF1BP1L
UIMC1
ULBP1
ULBP3
ULK2
UMODL1
UMODL1-AS1
UMPS
UNC119
UNC119B
UNC13A
UNC13B
UNC5C
UNC5D
UNC80
UNC93A
UNKL
UPF3B
UPK1B
UPP1
UPP2
UPRT
UQCR11
UQCRB
UQCRFS1
URB1
URI1
URM1
USB1
USF3
USH2A
USHBP1
USP1

USP10
USP12
USP13
USP14
USP15
USP18
USP22
USP24
USP25
USP3
USP31
USP32
USP37
USP38
USP42
USP44
USP46
USP47
USP48
USP49
USP51
USP53
USP6NL
USP7
USP8
USP9X
USPL1
UST
UTP14C
UTP15
UTP23
UTP25
UTP3
UTP4
UTRN
UTS2
UTY
UVRAG
UVSSA
UXS1
VAMP1
VAMP4
VAMP7
VANGL1

VANGL2
VAPA
VAPB
VASH2
VASP
VAT1
VAT1L
VAV2
VAV3
VAX1
VBP1
VCAM1
VCAN
VCP
VCPIP1
VDAC3
VEGFA
VEZF1
VEZT
VGLL3
VGLL4
VHL
VHLL
VIL1
VIP
VIPAS39
VIPR1
VIRMA
VKORC1L1
VLDLR
VMA21
VMP1
VN1R1
VNN1
VNN2
VPS13A
VPS13B
VPS13C
VPS18
VPS26A
VPS26B
VPS26C
VPS29
VPS36

VPS37A
VPS41
VPS4A
VPS4B
VPS50
VPS52
VPS53
VRK1
VRK2
VSIG1
VSIG10
VSIG10L
VSIG4
VSTM2A
VSTM4
VSTM5
VSX1
VTA1
VTI1A
VWA1
VWA2
VWA3A
VWA3B
VWA5A
VWA5B1
VWA8
VWC2
VWC2L
VWDE
VXN
WAC
WAPL
WARS
WARS2
WASF1
WASF3
WASHC2A
WASHC4
WASL
WBP11
WBP1L
WBP2NL
WBP4
WDCP

WDFY2
WDHD1
WDPCP
WDR12
WDR13
WDR20
WDR25
WDR3
WDR31
WDR33
WDR35
WDR4
WDR41
WDR44
WDR45B
WDR47
WDR48
WDR59
WDR5B
WDR7
WDR70
WDR73
WDR75
WDR76
WDR77
WDR78
WDR82
WDR89
WDR92
WDR93
WDTC1
WDYHV1
WFDC13
WFDC5
WHAMM
WIF1
WIPF1
WIPF2
WIPI2
WLS
WNK1
WNK2
WNK3
WNT16

WNT2
WNT2B
WNT3
WNT4
WNT8A
WNT8B
WNT9B
WRN
WSB1
WSB2
WSCD1
WT1
WTAP
WTIP
WWC2
WWC3
WWP1
WWP2
WWTR1
XAF1
XBP1
XCL1
XCL2
XCR1
XIAP
XK
XKR4
XKR7
XKR9
XPNPEP2
XPNPEP3
XPO1
XPOT
XPR1
XRCC2
XRCC4
XRN1
XRN2
XRRA1
XXYLT1
XYLB
XYLT1
XYLT2
YAE1

YAF2
YAP1
YARS
YARS2
YEATS2
YEATS4
YES1
YIPF4
YIPF6
YKT6
YME1L1
YOD1
YPEL1
YPEL2
YPEL5
YTHDC2
YTHDF1
YTHDF2
YTHDF3
YWHAG
YWHAH
YY1
ZADH2
ZBBX
ZBED1
ZBED3
ZBED4
ZBED6
ZBED8
ZBED9
ZBTB1
ZBTB11
ZBTB12
ZBTB14
ZBTB16
ZBTB2
ZBTB20
ZBTB21
ZBTB24
ZBTB25
ZBTB26
ZBTB3
ZBTB33
ZBTB34

ZBTB37
ZBTB38
ZBTB39
ZBTB40
ZBTB41
ZBTB42
ZBTB43
ZBTB44
ZBTB46
ZBTB5
ZBTB6
ZBTB7B
ZBTB7C
ZBTB8A
ZBTB8B
ZC2HC1A
ZC2HC1C
ZC3H12B
ZC3H12C
ZC3H13
ZC3H14
ZC3H15
ZC3H4
ZC3H7B
ZC3H8
ZC3HAV1
ZC3HAV1L
ZC3HC1
ZC4H2
ZCCHC10
ZCCHC14
ZCCHC17
ZCCHC2
ZCCHC24
ZCCHC4
ZCCHC7
ZCRB1
ZCWPW2
ZDBF2
ZDHHC13
ZDHHC14
ZDHHC15
ZDHHC16
ZDHHC17

ZDHHC2
ZDHHC20
ZDHHC21
ZDHHC23
ZDHHC3
ZDHHC6
ZDHHC7
ZDHHC9
ZEB1
ZEB2
ZER1
ZFAND2A
ZFAND3
ZFAND4
ZFAND5
ZFAND6
ZFAT
ZFC3H1
ZFHX2
ZFHX3
ZFP1
ZFP14
ZFP28
ZFP3
ZFP30
ZFP36L1
ZFP36L2
ZFP37
ZFP42
ZFP62
ZFP64
ZFP69
ZFP69B
ZFP82
ZFP90
ZFP91
ZFPM2
ZFR
ZFX
ZFY
ZFYVE1
ZFYVE16
ZFYVE21
ZFYVE28

ZG16
ZGPAT
ZHX1
ZIC1
ZIC2
ZIC3
ZIK1
ZIM3
ZKSCAN1
ZKSCAN2
ZKSCAN3
ZKSCAN4
ZKSCAN7
ZKSCAN8
ZMAT2
ZMAT3
ZMAT4
ZMIZ1
ZMPSTE24
ZMYM1
ZMYM2
ZMYM4
ZMYM6
ZMYND11
ZMYND8
ZNF10
ZNF100
ZNF106
ZNF112
ZNF117
ZNF132
ZNF134
ZNF136
ZNF138
ZNF14
ZNF140
ZNF141
ZNF143
ZNF146
ZNF148
ZNF154
ZNF157
ZNF160
ZNF169

ZNF174
ZNF175
ZNF180
ZNF182
ZNF184
ZNF185
ZNF189
ZNF19
ZNF197
ZNF20
ZNF200
ZNF207
ZNF208
ZNF212
ZNF214
ZNF215
ZNF217
ZNF222
ZNF223
ZNF224
ZNF226
ZNF230
ZNF234
ZNF235
ZNF236
ZNF236-DT
ZNF24
ZNF248
ZNF25
ZNF250
ZNF251
ZNF253
ZNF256
ZNF260
ZNF264
ZNF267
ZNF268
ZNF273
ZNF274
ZNF275
ZNF277
ZNF28
ZNF280C
ZNF280D

ZNF281
ZNF282
ZNF284
ZNF287
ZNF292
ZNF3
ZNF302
ZNF317
ZNF318
ZNF319
ZNF320
ZNF322
ZNF324B
ZNF326
ZNF329
ZNF330
ZNF331
ZNF333
ZNF334
ZNF337
ZNF33A
ZNF33B
ZNF345
ZNF346
ZNF347
ZNF35
ZNF354A
ZNF354B
ZNF354C
ZNF365
ZNF366
ZNF367
ZNF37A
ZNF382
ZNF383
ZNF384
ZNF385B
ZNF385D
ZNF391
ZNF394
ZNF395
ZNF396
ZNF397
ZNF404

ZNF407
ZNF410
ZNF415
ZNF417
ZNF419
ZNF420
ZNF426
ZNF429
ZNF43
ZNF430
ZNF431
ZNF432
ZNF436
ZNF436-AS1
ZNF439
ZNF440
ZNF441
ZNF442
ZNF443
ZNF444
ZNF446
ZNF449
ZNF45
ZNF451
ZNF460
ZNF461
ZNF462
ZNF468
ZNF470
ZNF471
ZNF480
ZNF483
ZNF484
ZNF485
ZNF486
ZNF487
ZNF490
ZNF491
ZNF493
ZNF497
ZNF501
ZNF503
ZNF507
ZNF510

ZNF512
ZNF513
ZNF516
ZNF518B
ZNF519
ZNF521
ZNF525
ZNF526
ZNF527
ZNF529
ZNF532
ZNF536
ZNF540
ZNF546
ZNF547
ZNF548
ZNF549
ZNF550
ZNF551
ZNF554
ZNF555
ZNF556
ZNF557
ZNF558
ZNF559-ZNF177
ZNF560
ZNF561
ZNF561-AS1
ZNF562
ZNF563
ZNF567
ZNF572
ZNF574
ZNF576
ZNF577
ZNF578
ZNF583
ZNF584
ZNF585A
ZNF585B
ZNF586
ZNF587
ZNF592
ZNF597

ZNF605
ZNF607
ZNF608
ZNF609
ZNF610
ZNF611
ZNF613
ZNF614
ZNF616
ZNF618
ZNF619
ZNF620
ZNF621
ZNF623
ZNF624
ZNF626
ZNF627
ZNF639
ZNF641
ZNF644
ZNF648
ZNF649
ZNF652
ZNF654
ZNF655
ZNF660
ZNF667
ZNF669
ZNF670
ZNF671
ZNF674
ZNF675
ZNF680
ZNF681
ZNF684
ZNF688
ZNF689
ZNF695
ZNF696
ZNF697
ZNF699
ZNF70
ZNF701
ZNF703

ZNF704
ZNF705A
ZNF705D
ZNF706
ZNF708
ZNF709
ZNF711
ZNF713
ZNF716
ZNF717
ZNF720
ZNF724
ZNF726
ZNF736
ZNF737
ZNF738
ZNF740
ZNF749
ZNF75A
ZNF763
ZNF765
ZNF766
ZNF770
ZNF772
ZNF773
ZNF774
ZNF776
ZNF778
ZNF780A
ZNF782
ZNF785
ZNF789
ZNF79
ZNF790
ZNF791
ZNF793
ZNF799
ZNF80
ZNF804B
ZNF81
ZNF813
ZNF814
ZNF823
ZNF827

ZNF829
ZNF830
ZNF831
ZNF84
ZNF843
ZNF844
ZNF845
ZNF850
ZNF852
ZNF853
ZNF860
ZNF862
ZNF875
ZNF879
ZNF891
ZNF90
ZNF92
ZNF93
ZNF99
ZNHIT1
ZNHIT6
ZNRF1
ZNRF2
ZNRF3
ZP1
ZPLD1
ZPR1
ZRANB1
ZRANB2
ZRANB3
ZSCAN1
ZSCAN12
ZSCAN16
ZSCAN18
ZSCAN2
ZSCAN22
ZSCAN23
ZSCAN25
ZSCAN29
ZSCAN30
ZSCAN31
ZSWIM4
ZSWIM6
ZSWIM7

ZUP1
ZW10
ZWILCH
ZWINT
ZXDA
ZXDB
ZXDC
ZYG11B
ZZEF1
ZZZ3

miRPathDB V2.0 database.

Table S2. Differentially Expressed genes identified in GSE62452 and GSE28735

		GSE62452		
**ID**	**P.Value**	**logFC**	**Gene.symbol**	**Gene.title**
7917455	1.34E-02	1.34E-01	*sep-15*	15 kDa selenoprotein
7930498	6.85E-12	1.42	*ACSL5*	acyl-CoA synthetase long-chain family member 5
7934906	1.69E-09	1.23	*ACTA2*	actin, alpha 2, smooth muscle, aorta
8145293	2.25E-11	1.23	*ADAM28*	ADAM metallopeptidase domain 28
8146000	5.52E-12	1.2	*ADAM9*	ADAM metallopeptidase domain 9
8111387	2.27E-12	1.14	*ADAMTS12*	ADAM metallopeptidase with thrombospondin type 1 motif 12
8126820	3.62E-12	1.38	*ADGRF1*	adhesion G protein-coupled receptor F1
8132557	4.33E-11	1.07	*AEBP1*	AE binding protein 1
8138381	5.39E-14	1.86	*AGR2*	anterior gradient 2, protein disulphide isomerase family member
7981514	7.88E-19	1.51	*AHNAK2*	AHNAK nucleoprotein 2
7934898	2.78E-08	1.14	*ANKRD22*	ankyrin repeat domain 22
8132318	5.39E-13	1.47	*ANLN*	anillin actin binding protein
7942135	7.97E-15	1.2	*ANO1*	anoctamin 1
8042439	3.30E-15	1.55	*ANTXR1*	anthrax toxin receptor 1
8101260	4.53E-12	1.03	*ANTXR2*	anthrax toxin receptor 2
8098246	2.82E-11	1.94	*ANXA10*	annexin A10
8072735	5.72E-15	1.43	*APOL1*	apolipoprotein L1
8095744	1.88E-05	1.08	*AREG*	amphiregulin
7954527	3.90E-12	1.08	*ARNTL2*	aryl hydrocarbon receptor nuclear translocator like 2
8040113	2.89E-18	1.03	*ASAP2*	ArfGAP with SH3 domain, ankyrin repeat and PH domain 2
7923086	5.15E-10	1.03	*ASPM*	abnormal spindle microtubule assembly
8162394	1.59E-08	1.16	*ASPN*	asporin
8067125	3.73E-13	1.21	*BCAS1*	breast carcinoma amplified sequence 1
8170648	7.58E-11	1.23	*BGN*	biglycan
8053417	1.90E-14	1.22	*CAPG*	capping actin protein, gelsolin like
8048864	1.01E-09	1.26	*CCL20*	C-C motif chemokine ligand 20
8120719	4.00E-12	1.25	*CD109*	CD109 molecule
8086517	9.70E-13	1.01	*CDCP1*	CUB domain containing protein 1
8001800	1.72E-13	1.51	*CDH11*	cadherin 11
7996819	2.25E-17	1.43	*CDH3*	cadherin 3

8037205	4.41E-12	1.1	*CEACAM1*	carcinoembryonic antigen related cell adhesion molecule 1
8029086	1.62E-15	2.79	*CEACAM5*	carcinoembryonic antigen related cell adhesion molecule 5
8029098	2.64E-15	2.42	*CEACAM6*	carcinoembryonic antigen related cell adhesion molecule 6
7985317	6.13E-14	1.39	*CEMIP*	cell migration inducing hyaluronan binding protein
7909708	9.65E-12	1.12	*CENPF*	centromere protein F
8082928	1.57E-10	1.72	*CLDN18*	claudin 18
8129082	1.42E-16	1.42	*COL10A1*	collagen type X alpha 1 chain
7918064	1.46E-15	1.85	*COL11A1*	collagen type XI alpha 1 chain
8127563	2.53E-11	1.65	*COL12A1*	collagen type XII alpha 1 chain
7936144	7.85E-18	1.65	*COL17A1*	collagen type XVII alpha 1 chain
8016646	2.75E-09	1.53	*COL1A1*	collagen type I alpha 1 chain
8046922	1.33E-08	1.42	*COL3A1*	collagen type III alpha 1 chain
8057620	3.35E-10	1.36	*COL5A2*	collagen type V alpha 2 chain
8059905	1.33E-08	1.27	*COL6A3*	collagen type VI alpha 3 chain
8081235	3.69E-12	1.37	*COL8A1*	collagen type VIII alpha 1 chain
8035517	4.25E-09	1.2	*COMP*	cartilage oligomeric matrix protein
8091385	9.59E-08	1.67	*CP*	ceruloplasmin (ferroxidase)
8065412	2.60E-11	1.65	*CST1*	cystatin SN
8065416	3.03E-12	1.04	*CST2*	cystatin SA
7909164	6.32E-18	2.55	*CTSE*	cathepsin E
7919815	3.20E-08	1.19	*CTSK*	cathepsin K
8100977	4.36E-10	1.23	*CXCL5*	C-X-C motif chemokine ligand 5
7929466	4.28E-09	1.26	*CYP2C18*	cytochrome P450 family 2 subfamily C member 18
8089082	1.72E-12	1.1	*DCBLD2*	discoidin, CUB and LCCL domain containing 2
8103563	8.80E-13	1.13	*DDX60*	DEXD/H-box helicase 60
7968800	3.46E-11	1.02	*DGKH*	diacylglycerol kinase eta
8046124	1.43E-09	1.25	*DHRS9*	dehydrogenase/reductase 9
8118061	3.67E-09	1.81	*DPCR1*	diffuse panbronchiolitis critical region 1
8114920	2.24E-09	1.1	*DPYSL3*	dihydropyrimidinase like 3
8020762	1.97E-06	1.04	*DSG3*	desmoglein 3
7988350	2.29E-04	1.03	*DUOX2*	dual oxidase 2
8083941	1.02E-14	1.19	*ECT2*	epithelial cell transforming 2
8112980	6.86E-13	1.61	*EDIL3*	EGF like repeats and discoidin domains 3

8097692	2.43E-13	1.32	*EDNRA*	endothelin receptor type A
7978544	2.28E-14	1.39	*EGLN3*	egl-9 family hypoxia inducible factor 3
8059279	8.50E-12	1.02	*EPHA4*	EPH receptor A4
7979179	1.62E-12	1.09	*ERO1A*	endoplasmic reticulum oxidoreductase 1 alpha
8138289	1.70E-11	1.02	*ETV1*	ETS variant 1
8056257	1.48E-10	1.61	*FAP*	fibroblast activation protein alpha
7988467	2.02E-07	1.19	*FBN1*	fibrillin 1
8152703	1.26E-16	1.45	*FBXO32*	F-box protein 32
8148220	9.66E-08	1.07	*FER1L6*	fer-1 like family member 6
8064904	2.27E-15	1.34	*FERMT1*	fermitin family member 1
7965541	1.38E-15	1.18	*FGD6*	FYVE, RhoGEF and PH domain containing 6
8058765	3.32E-15	2.19	*FN1*	fibronectin 1
8123104	1.08E-09	1.31	*FNDC1*	fibronectin type III domain containing 1
8027748	2.23E-14	1.32	*FXYD3*	FXYD domain containing ion transport regulator 3
8109926	9.35E-11	1.86	*GABRP*	gamma-aminobutyric acid type A receptor pi subunit
7984001	2.77E-09	1.34	*GCNT3*	glucosaminyl (N-acetyl) transferase 3, mucin type
7954065	8.40E-14	1.01	*GPRC5A*	G protein-coupled receptor class C group 5 member A
7979658	1.00E-08	1.07	*GPX2*	glutathione peroxidase 2
8105348	8.85E-11	1.08	*GPX8*	glutathione peroxidase 8 (putative)
7982377	7.03E-11	1.03	*GREM1*	gremlin 1, DAN family BMP antagonist
8167973	2.21E-12	1.07	*HEPH*	hephaestin
8042942	2.73E-13	1.16	*HK2*	hexokinase 2
8103769	6.90E-07	1.06	*HPGD*	hydroxyprostaglandin dehydrogenase 15-(NAD)
7976443	4.29E-14	1.23	*IFI27*	interferon alpha inducible protein 27
7902553	1.27E-09	1.09	*IFI44*	interferon induced protein 44
7902541	7.29E-10	1.42	*IFI44L*	interferon induced protein 44 like
8058857	1.23E-10	1.49	*IGFBP5*	insulin like growth factor binding protein 5
8029773	4.13E-12	1.08	*IGFL2*	IGF like family member 2
8084794	1.20E-13	1.08	*IL1RAP*	interleukin 1 receptor accessory protein
8102950	2.20E-14	1.18	*INPP4B*	inositol polyphosphate-4-phosphatase type II B
7989985	1.01E-14	1.09	*ITGA11*	integrin subunit alpha 11
8105267	1.33E-16	2.14	*ITGA2*	integrin subunit alpha 2
8008237	6.48E-14	1.43	*ITGA3*	integrin subunit alpha 3

8009951	9.48E-18	1.23	*ITGB4*	integrin subunit beta 4
8015387	1.04E-12	1.45	*KRT17*	keratin 17
8015349	1.87E-16	1.83	*KRT19*	keratin 19
7963421	2.82E-06	1.02	*KRT6A*	keratin 6A
7955613	3.49E-10	1.49	*KRT7*	keratin 7
8045539	1.89E-10	1.17	*KYNU*	kynureninase
7924029	4.02E-20	2.08	*LAMB3*	laminin subunit beta 3
7908072	4.10E-21	2.65	*LAMC2*	laminin subunit gamma 2
8060940	2.46E-09	1.16	*LAMP5*	lysosomal associated membrane protein family member 5
8158167	2.41E-07	1.12	*LCN2*	lipocalin 2
8102232	3.80E-15	1.23	*LEF1*	lymphoid enhancer binding factor 1
8092541	1.61E-11	1.47	*LIPH*	lipase H
7969438	7.51E-14	1.17	*LMO7*	LIM domain 7
8041383	3.42E-12	1.1	*LTBP1*	latent transforming growth factor beta binding protein 1
7957023	1.19E-08	1.05	*LYZ*	lysozyme
8050537	2.86E-10	1.23	*MATN3*	matrilin 3
8050160	5.54E-19	1.55	*MBOAT2*	membrane bound O-acyltransferase domain containing 2
8135601	2.14E-13	1.35	*MET*	MET proto-oncogene, receptor tyrosine kinase
7960919	1.26E-05	1.06	*MFAP5*	microfibrillar associated protein 5
7938485	5.47E-16	1.13	*MICAL2*	microtubule associated monooxygenase, calponin and LIM domain containing 2
8049487	3.71E-18	1.24	*MLPH*	melanophilin
7951271	9.75E-06	1.19	*MMP1*	matrix metallopeptidase 1
8071758	7.16E-13	1.23	*MMP11*	matrix metallopeptidase 11
7951297	1.94E-10	1.41	*MMP12*	matrix metallopeptidase 12
7973336	2.18E-13	1.19	*MMP14*	matrix metallopeptidase 14
7951217	3.58E-05	1.2	*MMP7*	matrix metallopeptidase 7
8090180	1.56E-08	1.51	*MUC13*	mucin 13, cell surface associated
8033674	8.10E-11	1.19	*MUC16*	mucin 16, cell surface associated
8135048	1.21E-07	1.3	*MUC17*	mucin 17, cell surface associated
7935058	1.29E-14	1.39	*MYOF*	myoferlin
7950933	6.10E-16	1.41	*NOX4*	NADPH oxidase 4
8104758	2.01E-10	1.27	*NPR3*	natriuretic peptide receptor 3
8002303	2.18E-14	1.31	*NQO1*	NAD(P)H quinone dehydrogenase 1

8047763	2.10E-10	1.16	*NRP2*	neuropilin 2
8120967	1.76E-10	1.24	*NT5E*	5'-nucleotidase ecto
7958913	1.00E-12	1.04	*OAS2*	2'-5'-oligoadenylate synthetase 2
7961142	3.79E-07	1.24	*OLR1*	oxidized low density lipoprotein receptor 1
8138613	1.99E-14	1.16	*OSBPL3*	oxysterol binding protein like 3
7898413	1.04E-11	1.14	*PADI1*	peptidyl arginine deiminase 1
8056151	2.61E-13	1.11	*PLA2R1*	phospholipase A2 receptor 1
8101429	4.58E-13	1.76	*PLAC8*	placenta specific 8
8150509	2.92E-11	1.41	*PLAT*	plasminogen activator, tissue type
7928429	1.67E-14	1.29	*PLAU*	plasminogen activator, urokinase
7979710	1.41E-14	1.09	*PLEK2*	pleckstrin 2
7971077	3.76E-15	2.5	*POSTN*	periostin
7964907	2.54E-13	1.01	*PTPRR*	protein tyrosine phosphatase, receptor type R
8120043	4.31E-15	1.03	*RUNX2*	runt related transcription factor 2
7920297	1.97E-10	1.08	*S100A14*	S100 calcium binding protein A14
7969493	9.91E-15	1.55	*SCEL*	sciellin
8150889	5.14E-15	1.2	*SDR16C5*	short chain dehydrogenase/reductase family 16C, member 5
8140534	2.57E-08	1.26	*SEMA3C*	semaphorin 3C
8023696	1.11E-05	1.1	*SERPINB3*	serpin family B member 3
8021584	1.54E-16	1.97	*SERPINB5*	serpin family B member 5
8123246	3.88E-11	1.13	*SLC22A3*	solute carrier family 22 member 3
7915472	9.61E-19	1.67	*SLC2A1*	solute carrier family 2 member 1
8125149	2.91E-09	1.08	*SLC44A4*	solute carrier family 44 member 4
8169504	1.08E-16	2.66	*SLC6A14*	solute carrier family 6 member 14
8078014	7.80E-14	1.2	*SLC6A6*	solute carrier family 6 member 6
8066493	7.27E-14	1.73	*SLPI*	secretory leukocyte peptidase inhibitor
8115327	5.79E-08	1.03	*SPARC*	secreted protein acidic and cysteine rich
8168749	1.87E-08	1.07	*SRPX2*	sushi repeat containing protein, X-linked 2
7961215	4.25E-17	1.03	*STYK1*	serine/threonine/tyrosine kinase 1
8146863	3.54E-15	1.98	*SULF1*	sulfatase 1
8066822	2.14E-09	1.09	*SULF2*	sulfatase 2
7950810	1.82E-13	1.06	*SYTL2*	synaptotagmin like 2
7948444	2.51E-07	1.59	*TCN1*	transcobalamin 1

8070579	6.08E-09	1.51	*TFF1*	trefoil factor 1
8108217	6.23E-09	1.02	*TGFBI*	transforming growth factor beta induced
8066214	1.38E-11	1.26	*TGM2*	transglutaminase 2
8130867	3.13E-12	1.53	*THBS2*	thrombospondin 2
7993638	3.51E-10	1.55	*TMC5*	transmembrane channel like 5
7993588	1.31E-17	1.14	*TMC7*	transmembrane channel like 7
7945169	2.66E-09	1.12	*TMEM45B*	transmembrane protein 45B
7944164	1.38E-18	2.06	*TMPRSS4*	transmembrane protease, serine 4
8045688	1.22E-06	1.15	*TNFAIP6*	TNF alpha induced protein 6
8015016	4.18E-14	1.31	*TNS4*	tensin 4
8014974	4.39E-11	1.16	*TOP2A*	topoisomerase (DNA) II alpha
7952290	6.26E-16	1.28	*TRIM29*	tripartite motif containing 29
8179617	7.88E-11	1.35	*TRIM31*	tripartite motif containing 31
7901175	1.30E-19	2.38	*TSPAN1*	tetraspanin 1
7964927	1.28E-07	1.39	*TSPAN8*	tetraspanin 8
8106743	9.57E-12	1.76	*VCAN*	versican
8169263	1.16E-09	1.6	*VSIG1*	V-set and immunoglobulin domain containing 1
8058591	2.01E-10	-1.45	*ACADL*	acyl-CoA dehydrogenase, long chain
8101881	8.64E-06	-1.11	*ADH1B*	alcohol dehydrogenase 1B (class I), beta polypeptide
8095628	6.14E-10	-2.51	*ALB*	albumin
8074201	3.98E-08	-1.2	*ANKRD62*	ankyrin repeat domain 62
7991335	1.40E-09	-1.78	*ANPEP*	alanyl aminopeptidase, membrane
8047300	4.56E-13	-1.77	*AOX1*	aldehyde oxidase 1
7994252	4.52E-08	-1.46	*AQP8*	aquaporin 8
7951910	7.03E-09	-1.14	*BACE1*	beta-secretase 1
8174201	7.50E-10	-1.07	*BEX1*	brain expressed X-linked 1
7937079	9.88E-08	-1.13	*BNIP3*	BCL2 interacting protein 3
7908917	1.93E-12	-1.12	*BTG2*	BTG anti-proliferation factor 2
7989473	7.82E-09	-1.12	*C2CD4B*	C2 calcium dependent domain containing 4B
8163839	6.42E-10	-1.13	*C5*	complement component 5
8111864	2.87E-07	-1.18	*C6*	complement component 6
8158976	2.54E-06	-2.46	*CEL*	carboxyl ester lipase
7898176	2.00E-06	-2.14	*CELA2A*	chymotrypsin like elastase family member 2A

7898184	1.26E-06	-2.09	*CELA2B*	chymotrypsin like elastase family member 2B
7898725	4.89E-06	-2.12	*CELA3A*	chymotrypsin like elastase family member 3A
7898713	2.05E-06	-1.96	*CELA3B*	chymotrypsin like elastase family member 3B
8158987	6.37E-08	-1.45	*CELP*	carboxyl ester lipase pseudogene
8135661	1.55E-03	-1.28	*CFTR*	cystic fibrosis transmembrane conductance regulator
7910915	2.14E-07	-1.02	*CHRM3*	cholinergic receptor muscarinic 3
8125936	2.10E-06	-2.35	*CLPS*	colipase
8136235	3.14E-05	-1.9	*CPA1*	carboxypeptidase A1
8136187	4.02E-06	-2.5	*CPA2*	carboxypeptidase A2
8083246	9.24E-05	-1.71	*CPB1*	carboxypeptidase B1
8110990	8.07E-10	-1.19	*CTNND2*	catenin delta 2
7898167	5.05E-07	-2.57	*CTRC*	chymotrypsin C
8002121	7.07E-08	-2.06	*CTRL*	chymotrypsin like
8124196	8.77E-07	-1.18	*DCDC2*	doublecortin domain containing 2
8044700	8.95E-09	-1.1	*DPP10*	dipeptidyl peptidase like 10
8096845	1.76E-07	-1.82	*EGF*	epidermal growth factor
8145532	3.54E-12	-1.05	*EPHX2*	epoxide hydrolase 2
7925342	1.18E-11	-1.62	*ERO1B*	endoplasmic reticulum oxidoreductase 1 beta
7961524	8.66E-08	-2.42	*ERP27*	endoplasmic reticulum protein 27
8098671	7.89E-09	-1.67	*F11*	coagulation factor XI
8176193	3.08E-08	-1.07	*F8*	coagulation factor VIII
8151532	1.57E-04	-1.25	*FABP4*	fatty acid binding protein 4
8068684	1.96E-05	-1.03	*FAM3B*	family with sequence similarity 3 member B
8149521	7.69E-08	-1.67	*FGL1*	fibrinogen like 1
8046116	6.36E-07	-1.09	*G6PC2*	glucose-6-phosphatase catalytic subunit 2
7988414	1.78E-05	-1.54	*GATM*	glycine amidinotransferase
8119620	7.59E-07	-1.28	*GNMT*	glycine N-methyltransferase
7999920	1.06E-06	-2.6	*GP2*	glycoprotein 2
7949320	1.96E-07	-1.05	*GPHA2*	glycoprotein hormone alpha 2
8056327	1.15E-08	-1.04	*GRB14*	growth factor receptor bound protein 14
8166202	2.77E-07	-1.2	*GRPR*	gastrin releasing peptide receptor
8127065	5.66E-09	-1.38	*GSTA2*	glutathione S-transferase alpha 2
8089402	1.70E-08	-1.17	*GUCA1C*	guanylate cyclase activator 1C

7991034	3.55E-08	-1.15	*HOMER2*	homer scaffolding protein 2
7954377	2.90E-10	-2.31	*IAPP*	islet amyloid polypeptide
7913768	4.46E-06	-1.04	*IL22RA1*	interleukin 22 receptor subunit alpha 1
8009493	2.49E-06	-1.35	*KCNJ16*	potassium voltage-gated channel subfamily J member 16
7903592	7.50E-09	-1.78	*KIAA1324*	KIAA1324
8038633	1.13E-07	-1.79	*KLK1*	kallikrein 1
7961580	1.30E-11	-1.12	*LMO3*	LIM domain only 3
8052372	5.28E-08	-1.3	*MIR217*	microRNA 217
8001531	3.95E-08	-1.22	*MT1G*	metallothionein 1G
7908597	3.29E-09	-1.85	*NR5A2*	nuclear receptor subfamily 5 group A member 2
8142270	1.12E-09	-1.33	*NRCAM*	neuronal cell adhesion molecule
7990555	1.31E-07	-1.53	*NRG4*	neuregulin 4
7938687	2.11E-08	-1.02	*NUCB2*	nucleobindin 2
7988987	1.00E-05	-1.02	*ONECUT1*	one cut homeobox 1
7944793	1.56E-08	-1.31	*OR8D4*	olfactory receptor family 8 subfamily D member 4
8169389	2.99E-08	-1.19	*PAK3*	p21 (RAC1) activated kinase 3
7991815	2.27E-08	-1.94	*PDIA2*	protein disulfide isomerase family A member 2
8141094	5.08E-11	-1.52	*PDK4*	pyruvate dehydrogenase kinase 4
8126954	4.13E-04	-1.03	*PKHD1*	polycystic kidney and hepatic disease 1 (autosomal recessive)
7967034	2.19E-06	-2.22	*PLA2G1B*	phospholipase A2 group IB
7923837	5.02E-08	-1.35	*PM20D1*	peptidase M20 domain containing 1
7930790	1.51E-07	-2.84	*PNLIPRP1*	pancreatic lipase related protein 1
7930804	1.10E-06	-2.6	*PNLIPRP2*	pancreatic lipase related protein 2 (gene/pseudogene)
8136795	8.74E-05	-1.64	*PRSS1*	protease, serine 1
8154848	4.34E-06	-1.25	*PRSS3*	protease, serine 3
8136801	3.24E-05	-1.5	*PRSS3P2*	protease, serine 3 pseudogene 2
8062948	2.77E-08	-1.52	*RBPJL*	recombination signal binding protein for immunoglobulin kappa J region like
8042986	1.89E-04	-1.58	*REG1A*	regenerating family member 1 alpha
8053330	1.41E-04	-1.96	*REG1B*	regenerating family member 1 beta
8053337	6.00E-05	-1.98	*REG1CP*	regenerating family member 1 gamma, pseudogene
8053341	7.73E-04	-1.49	*REG3A*	regenerating family member 3 alpha
8042978	1.49E-04	-1.27	*REG3G*	regenerating family member 3 gamma
8167027	3.63E-09	-1.03	*RGN*	regucalcin

8117288	2.25E-06	-1.28	*SCGN*	secretagogin, EF-hand calcium binding protein
8091910	3.15E-08	-2.3	*SERPINI2*	serpin family I member 2
7934936	3.80E-10	-1.52	*SLC16A12*	solute carrier family 16 member 12
8117304	2.03E-06	-1.15	*SLC17A4*	solute carrier family 17 member 4
7956229	2.67E-08	-1.29	*SLC39A5*	solute carrier family 39 member 5
7948249	2.57E-09	-1.12	*SLC43A1*	solute carrier family 43 member 1
8095585	4.87E-05	-1.39	*SLC4A4*	solute carrier family 4 member 4
8144786	1.34E-13	-1.39	*SLC7A2*	solute carrier family 7 member 2
8114964	1.33E-03	-1.3	*SPINK1*	serine peptidase inhibitor, Kazal type 1
8092682	1.94E-06	-1.03	*SST*	somatostatin
8036699	9.75E-07	-1.25	*SYCN*	syncollin
8141016	2.75E-07	-1.11	*TFPI2*	tissue factor pathway inhibitor 2
8002283	4.20E-10	-1.73	*TMED6*	transmembrane p24 trafficking protein 6
7957221	1.77E-08	-1.61	*TRHDE*	thyrotropin releasing hormone degrading enzyme
8057056	1.89E-12	-1.2	*TTN*	titin
8020795	1.18E-02	-1.04	*TTR*	transthyretin

GSE28735

ID	P.Value	logFC	Gene.symbol	Gene.title
7985310	1.83E-15	1.128362	*ABHD17C*	abhydrolase domain containing 17C
7930498	1.19E-09	1.4634847	*ACSL5*	acyl-CoA synthetase long-chain family member 5
7934906	8.51E-07	1.1454927	*ACTA2*	actin, alpha 2, smooth muscle, aorta
7936968	8.37E-05	1.0717007	*ADAM12*	ADAM metallopeptidase domain 12
8145293	1.87E-07	1.141082	*ADAM28*	ADAM metallopeptidase domain 28
8146000	2.77E-09	1.1530387	*ADAM9*	ADAM metallopeptidase domain 9
8111387	2.08E-11	1.4163264	*ADAMTS12*	ADAM metallopeptidase with thrombospondin type 1 motif 12
8112342	2.85E-10	1.038462	*ADAMTS6*	ADAM metallopeptidase with thrombospondin type 1 motif 6
8126820	9.84E-11	1.6035156	*ADGRF1*	adhesion G protein-coupled receptor F1
8132557	5.02E-08	1.1198558	*AEBP1*	AE binding protein 1
8094028	1.10E-09	1.2469322	*AFAP1-AS1*	AFAP1 antisense RNA 1
8138381	5.69E-13	2.1373789	*AGR2*	anterior gradient 2, protein disulphide isomerase family member
7981514	1.40E-16	1.7455516	*AHNAK2*	AHNAK nucleoprotein 2
7962183	8.82E-10	1.062622	*AK4*	adenylate kinase 4

8149071	3.01E-07	1.0255767	*ANGPT2*	angiopoietin 2
7934898	1.10E-10	1.5765009	*ANKRD22*	ankyrin repeat domain 22
8132318	4.63E-11	1.6620173	*ANLN*	anillin actin binding protein
7942135	1.27E-11	1.2739089	*ANO1*	anoctamin 1
8042439	5.32E-11	1.5904338	*ANTXR1*	anthrax toxin receptor 1
8098246	5.23E-11	2.3579367	*ANXA10*	annexin A10
8095986	1.60E-07	1.0462073	*ANXA3*	annexin A3
8072735	2.49E-12	1.5050227	*APOL1*	apolipoprotein L1
8095744	5.40E-04	1.0105213	*AREG*	amphiregulin
7954527	9.10E-11	1.2634424	*ARNTL2*	aryl hydrocarbon receptor nuclear translocator like 2
8040113	1.48E-14	1.0555331	*ASAP2*	ArfGAP with SH3 domain, ankyrin repeat and PH domain 2
7923086	5.33E-09	1.2180584	*ASPM*	abnormal spindle microtubule assembly
7997593	3.79E-09	1.0605813	*ATP2C2*	ATPase secretory pathway Ca2+ transporting 2
8141206	9.69E-10	1.2480627	*BAIAP2L1*	BAI1 associated protein 2 like 1
8067125	5.07E-13	1.5367902	*BCAS1*	breast carcinoma amplified sequence 1
8170648	1.88E-07	1.2178456	*BGN*	biglycan
8053417	3.20E-12	1.409224	*CAPG*	capping actin protein, gelsolin like
8006594	1.67E-04	1.1312298	*CCL18*	C-C motif chemokine ligand 18
8048864	3.22E-10	1.3648089	*CCL20*	C-C motif chemokine ligand 20
8120719	3.63E-09	1.2498704	*CD109*	CD109 molecule
8086517	4.12E-12	1.1710249	*CDCP1*	CUB domain containing protein 1
8001800	4.02E-09	1.4890444	*CDH11*	cadherin 11
7996819	8.76E-16	1.6362931	*CDH3*	cadherin 3
8037205	1.81E-11	1.292694	*CEACAM1*	carcinoembryonic antigen related cell adhesion molecule 1
8029086	2.16E-13	3.1812869	*CEACAM5*	carcinoembryonic antigen related cell adhesion molecule 5
8029098	1.06E-12	2.6271224	*CEACAM6*	carcinoembryonic antigen related cell adhesion molecule 6
7985317	1.68E-13	1.8033796	*CEMIP*	cell migration inducing hyaluronan binding protein
7909708	1.11E-10	1.3075462	*CENPF*	centromere protein F
8082928	2.26E-10	2.1265416	*CLDN18*	claudin 18
8129082	3.23E-14	1.5692449	*COL10A1*	collagen type X alpha 1 chain
7918064	3.05E-13	2.1946749	*COL11A1*	collagen type XI alpha 1 chain
8127563	7.18E-09	1.732026	*COL12A1*	collagen type XII alpha 1 chain
7936144	2.22E-16	1.8936258	*COL17A1*	collagen type XVII alpha 1 chain

8016646	8.94E-07	1.5020929	*COL1A1*	collagen type I alpha 1 chain
8046922	5.70E-06	1.3701904	*COL3A1*	collagen type III alpha 1 chain
8057620	3.70E-07	1.3867727	*COL5A2*	collagen type V alpha 2 chain
8059905	6.07E-06	1.2233471	*COL6A3*	collagen type VI alpha 3 chain
8081235	4.54E-09	1.4414827	*COL8A1*	collagen type VIII alpha 1 chain
8035517	5.99E-08	1.4673376	*COMP*	cartilage oligomeric matrix protein
8100154	1.61E-08	1.0800867	*CORIN*	corin, serine peptidase
8091385	1.94E-06	1.767232	*CP*	ceruloplasmin (ferroxidase)
8065412	2.10E-10	2.0248356	*CST1*	cystatin SN
8065416	7.25E-11	1.2321847	*CST2*	cystatin SA
8147777	5.55E-11	1.0049256	*CTHRC1*	collagen triple helix repeat containing 1
7909164	6.00E-15	2.6841011	*CTSE*	cathepsin E
7919815	2.32E-05	1.0460687	*CTSK*	cathepsin K
8100977	2.77E-09	1.5615738	*CXCL5*	C-X-C motif chemokine ligand 5
7929466	8.67E-08	1.4042131	*CYP2C18*	cytochrome P450 family 2 subfamily C member 18
8089082	6.57E-09	1.0321687	*DCBLD2*	discoidin, CUB and LCCL domain containing 2
8103563	1.29E-08	1.0535862	*DDX60*	DEXD/H-box helicase 60
7968800	1.59E-08	1.0501716	*DGKH*	diacylglycerol kinase eta
8046124	8.43E-08	1.2735449	*DHRS9*	dehydrogenase/reductase 9
7927631	3.61E-09	1.2162724	*DKK1*	dickkopf WNT signaling pathway inhibitor 1
7979307	1.49E-08	1.1127349	*DLGAP5*	DLG associated protein 5
8118061	6.51E-10	2.1386271	*DPCR1*	diffuse panbronchiolitis critical region 1
8114920	1.11E-06	1.1362667	*DPYSL3*	dihydropyrimidinase like 3
8020779	2.73E-06	1.0892258	*DSG2*	desmoglein 2
8020762	1.17E-04	1.0104942	*DSG3*	desmoglein 3
8083941	1.39E-12	1.3293738	*ECT2*	epithelial cell transforming 2
8112980	2.04E-08	1.5138422	*EDIL3*	EGF like repeats and discoidin domains 3
8097692	4.42E-09	1.3127658	*EDNRA*	endothelin receptor type A
8113433	2.78E-12	1.1553482	*EFNA5*	ephrin A5
7972713	1.22E-11	1.0293451	*EFNB2*	ephrin B2
7978544	5.13E-13	1.6243329	*EGLN3*	egl-9 family hypoxia inducible factor 3
7953532	1.80E-09	1.1134007	*ENO2*	enolase 2
8059279	3.01E-09	1.1711916	*EPHA4*	EPH receptor A4

7965390	1.27E-04	1.0430022	*EPYC*	epiphycan
7979179	4.80E-12	1.219264	*ERO1A*	endoplasmic reticulum oxidoreductase 1 alpha
8112045	6.30E-13	1.0108476	*ESM1*	endothelial cell specific molecule 1
8138289	7.65E-08	1.0579796	*ETV1*	ETS variant 1
8056257	6.13E-07	1.4972633	*FAP*	fibroblast activation protein alpha
7988467	1.49E-04	1.0233667	*FBN1*	fibrillin 1
8152703	2.43E-14	1.4711818	*FBXO32*	F-box protein 32
8065948	2.05E-13	1.089496	*FER1L4*	fer-1 like family member 4, pseudogene
8148220	4.80E-08	1.3372369	*FER1L6*	fer-1 like family member 6
8064904	2.45E-12	1.5010753	*FERMT1*	fermitin family member 1
7965541	2.57E-12	1.2339911	*FGD6*	FYVE, RhoGEF and PH domain containing 6
8058765	3.25E-12	2.2111	*FN1*	fibronectin 1
8123104	4.97E-08	1.4763207	*FNDC1*	fibronectin type III domain containing 1
8116579	3.69E-11	1.1159918	*FOXQ1*	forkhead box Q1
8033054	3.99E-08	1.0387011	*FUT3*	fucosyltransferase 3 (Lewis blood group)
8027748	1.66E-13	1.5452418	*FXYD3*	FXYD domain containing ion transport regulator 3
8109926	9.38E-08	2.0308344	*GABRP*	gamma-aminobutyric acid type A receptor pi subunit
7984001	1.02E-07	1.4315447	*GCNT3*	glucosaminyl (N-acetyl) transferase 3, mucin type
7970441	1.15E-15	1.0717644	*GJB2*	gap junction protein beta 2
7954065	2.89E-12	1.1751238	*GPRC5A*	G protein-coupled receptor class C group 5 member A
7979658	6.73E-07	1.1878256	*GPX2*	glutathione peroxidase 2
8105348	2.15E-07	1.0094489	*GPX8*	glutathione peroxidase 8 (putative)
7982377	4.57E-09	1.0603713	*GREM1*	gremlin 1, DAN family BMP antagonist
8167973	4.92E-09	1.2034709	*HEPH*	hephaestin
8117589	7.94E-11	1.1253782	*HIST1H3H*	histone cluster 1, H3h
8124531	1.04E-09	1.0039847	*HIST1H3I*	histone cluster 1, H3i
8042942	5.71E-11	1.3526864	*HK2*	hexokinase 2
8016457	3.11E-14	1.0872489	*HOXB5*	homeobox B5
8103769	1.76E-05	1.1121756	*HPGD*	hydroxyprostaglandin dehydrogenase 15-(NAD)
7976443	1.65E-11	1.3512633	*IFI27*	interferon alpha inducible protein 27
7902541	1.27E-05	1.2289118	*IFI44L*	interferon induced protein 44 like
8138566	2.82E-09	1.1658791	*IGF2BP3*	insulin like growth factor 2 mRNA binding protein 3
8058857	1.19E-06	1.3557069	*IGFBP5*	insulin like growth factor binding protein 5

8029773	7.32E-12	1.4258422	*IGFL2*	IGF like family member 2
8043981	2.67E-08	1.0359142	*IL1R2*	interleukin 1 receptor type 2
8084794	8.95E-11	1.0983738	*IL1RAP*	interleukin 1 receptor accessory protein
8173444	4.46E-08	1.0835424	*IL2RG*	interleukin 2 receptor subunit gamma
8102950	3.31E-11	1.12227	*INPP4B*	inositol polyphosphate-4-phosphatase type II B
7989985	2.48E-13	1.3723916	*ITGA11*	integrin subunit alpha 11
8105267	5.63E-14	2.2658244	*ITGA2*	integrin subunit alpha 2
8008237	2.38E-12	1.5188551	*ITGA3*	integrin subunit alpha 3
8009951	2.71E-14	1.303354	*ITGB4*	integrin subunit beta 4
8037408	1.65E-18	1.0487336	*KCNN4*	potassium calcium-activated channel subfamily N member 4
7984540	6.91E-09	1.0248458	*KIF23*	kinesin family member 23
8005449	1.19E-10	1.1537338	*KRT17*	keratin 17
8015349	1.25E-14	2.0580204	*KRT19*	keratin 19
7963421	3.52E-05	1.0361116	*KRT6A*	keratin 6A
7955613	1.27E-08	1.6835824	*KRT7*	keratin 7
8045539	4.37E-08	1.2622391	*KYNU*	kynureninase
8020551	7.66E-15	1.3547947	*LAMA3*	laminin subunit alpha 3
7924029	1.19E-18	2.3442298	*LAMB3*	laminin subunit beta 3
7908072	7.03E-19	2.9016536	*LAMC2*	laminin subunit gamma 2
8060940	6.15E-07	1.2891922	*LAMP5*	lysosomal associated membrane protein family member 5
8158167	4.06E-06	1.1424984	*LCN2*	lipocalin 2
8102232	4.12E-11	1.3104127	*LEF1*	lymphoid enhancer binding factor 1
8092541	1.05E-10	1.6364271	*LIPH*	lipase H
7969438	7.10E-12	1.177314	*LMO7*	LIM domain 7
8149774	1.02E-10	1.2380353	*LOXL2*	lysyl oxidase like 2
8077366	1.05E-07	1.1022207	*LRRN1*	leucine rich repeat neuronal 1
8041383	1.39E-08	1.0574087	*LTBP1*	latent transforming growth factor beta binding protein 1
8138466	2.44E-05	1.0055842	*MACC1*	metastasis associated in colon cancer 1
8054479	6.50E-10	1.0471758	*MALL*	mal, T-cell differentiation protein like
8050537	1.98E-08	1.3906947	*MATN3*	matrilin 3
8050160	1.95E-14	1.5977576	*MBOAT2*	membrane bound O-acyltransferase domain containing 2
8155214	2.08E-09	1.1532836	*MELK*	maternal embryonic leucine zipper kinase
8135601	5.01E-12	1.4803842	*MET*	MET proto-oncogene, receptor tyrosine kinase

7960919	1.47E-04	1.0858004	*MFAP5*	microfibrillar associated protein 5
7938485	1.33E-13	1.2684082	*MICAL2*	microtubule associated monooxygenase, calponin and LIM domain containing 2
7937020	3.11E-09	1.1643182	*MKI67*	marker of proliferation Ki-67
8049487	2.82E-18	1.4629327	*MLPH*	melanophilin
7951271	1.96E-04	1.2251593	*MMP1*	matrix metallopeptidase 1
8071758	1.08E-11	1.5014524	*MMP11*	matrix metallopeptidase 11
7951297	4.19E-09	1.7443562	*MMP12*	matrix metallopeptidase 12
7973336	7.61E-11	1.325874	*MMP14*	matrix metallopeptidase 14
7951217	2.34E-04	1.2129338	*MMP7*	matrix metallopeptidase 7
8063115	5.12E-09	1.1166764	*MMP9*	matrix metallopeptidase 9
8087547	5.22E-16	1.1476047	*MST1R*	macrophage stimulating 1 receptor
7919669	1.87E-13	1.1160298	*MTMR11*	myotubularin related protein 11
7920642	4.51E-06	1.1236976	*MUC1*	mucin 1, cell surface associated
8090180	1.44E-06	1.6196007	*MUC13*	mucin 13, cell surface associated
8033674	2.46E-09	1.2668127	*MUC16*	mucin 16, cell surface associated
8135048	9.06E-07	1.7016829	*MUC17*	mucin 17, cell surface associated
8171172	4.22E-08	1.1398482	*MXRA5*	matrix remodeling associated 5
7935058	4.50E-11	1.3488062	*MYOF*	myoferlin
8100464	2.02E-10	1.0295189	*NMU*	neuromedin U
7950933	5.05E-13	1.6264947	*NOX4*	NADPH oxidase 4
8104746	2.75E-08	1.2844418	*NPR3*	natriuretic peptide receptor 3
8002303	3.20E-13	1.4664629	*NQO1*	NAD(P)H quinone dehydrogenase 1
8047763	2.66E-08	1.2613624	*NRP2*	neuropilin 2
8120967	2.80E-07	1.208268	*NT5E*	5'-nucleotidase ecto
7945245	1.21E-11	1.0137033	*NTM*	neurotrimin
7958884	3.86E-10	1.130268	*OAS1*	2'-5'-oligoadenylate synthetase 1
7958913	5.84E-09	1.0571731	*OAS2*	2'-5'-oligoadenylate synthetase 2
8100298	5.13E-10	1.1130531	*OCIAD2*	OCIA domain containing 2
7961142	1.72E-05	1.2763264	*OLR1*	oxidized low density lipoprotein receptor 1
8138613	3.71E-10	1.1831684	*OSBPL3*	oxysterol binding protein like 3
7898413	2.37E-10	1.3742273	*PADI1*	peptidyl arginine deiminase 1
8094520	3.29E-12	1.0136969	*PCDH7*	protocadherin 7
7950391	1.75E-11	1.0538702	*PGM2L1*	phosphoglucomutase 2 like 1

7990151	1.10E-11	1.021846	*PKM*	pyruvate kinase, muscle
8056151	4.39E-09	1.1070027	*PLA2R1*	phospholipase A2 receptor 1
8101429	8.34E-10	1.8359027	*PLAC8*	placenta specific 8
8150509	2.00E-08	1.3462227	*PLAT*	plasminogen activator, tissue type
7928429	2.54E-10	1.3743673	*PLAU*	plasminogen activator, urokinase
7979710	1.11E-14	1.2965764	*PLEK2*	pleckstrin 2
7930980	5.44E-15	1.1414518	*PLPP4*	phospholipid phosphatase 4
8083146	1.05E-05	1.0651547	*PLS1*	plastin 1
7971077	6.76E-13	2.6298373	*POSTN*	periostin
7964907	2.94E-12	1.2630898	*PTPRR*	protein tyrosine phosphatase, receptor type R
8050007	4.54E-07	1.1653407	*PXDN*	peroxidasin
8104788	2.46E-09	1.0131847	*RAI14*	retinoic acid induced 14
7915184	1.90E-13	1.1258644	*RHBDL2*	rhomboid like 2
7933855	3.56E-11	1.0185636	*RTKN2*	rhotekin 2
8120043	4.49E-13	1.2121193	*RUNX2*	runt related transcription factor 2
7920297	3.91E-10	1.2504989	*S100A14*	S100 calcium binding protein A14
7920291	1.04E-12	1.1656542	*S100A16*	S100 calcium binding protein A16
8093950	1.43E-17	1.2260793	*S100P*	S100 calcium binding protein P
7969493	9.21E-14	1.6301907	*SCEL*	sciellin
7960529	2.41E-07	1.0022056	*SCNN1A*	sodium channel epithelial 1 alpha subunit
8150889	2.50E-15	1.5155709	*SDR16C5*	short chain dehydrogenase/reductase family 16C, member 5
8140534	4.29E-05	1.0999293	*SEMA3C*	semaphorin 3C
7990345	2.29E-12	1.0218449	*SEMA7A*	semaphorin 7A (John Milton Hagen blood group)
8023696	7.48E-05	1.2747167	*SERPINB3*	serpin family B member 3
8021584	1.26E-14	2.1830693	*SERPINB5*	serpin family B member 5
7902127	1.52E-11	1.0548758	*SGIP1*	SH3 domain GRB2 like endophilin interacting protein 1
8010770	1.32E-13	1.0000809	*SLC16A3*	solute carrier family 16 member 3
8123246	1.57E-07	1.14787	*SLC22A3*	solute carrier family 22 member 3
7923850	2.85E-07	1.0173151	*SLC26A9*	solute carrier family 26 member 9
7915472	2.13E-15	1.8381029	*SLC2A1*	solute carrier family 2 member 1
8125149	1.29E-09	1.2232882	*SLC44A4*	solute carrier family 44 member 4
8169504	2.65E-15	3.075768	*SLC6A14*	solute carrier family 6 member 14
8078014	4.11E-10	1.2712856	*SLC6A6*	solute carrier family 6 member 6

8044080	1.44E-10	1.0730247	*SLC9A2*	solute carrier family 9 member A2
7954330	3.35E-05	1.1014516	*SLCO1B3*	solute carrier organic anion transporter family member 1B3
8066493	6.36E-11	1.7899024	*SLPI*	secretory leukocyte peptidase inhibitor
8168749	2.01E-06	1.1052856	*SRPX2*	sushi repeat containing protein, X-linked 2
8018774	1.77E-09	1.028552	*ST6GALNAC1*	ST6 N-acetylgalactosaminide alpha-2,6-sialyltransferase 1
7961215	2.27E-16	1.2155107	*STYK1*	serine/threonine/tyrosine kinase 1
8146863	1.31E-10	1.9594916	*SULF1*	sulfatase 1
8066822	1.82E-06	1.0889349	*SULF2*	sulfatase 2
8100798	1.85E-05	1.0806951	*SULT1B1*	sulfotransferase family 1B member 1
8044212	4.43E-05	1.0128167	*SULT1C2*	sulfotransferase family 1C member 2
7950810	1.15E-10	1.0928962	*SYTL2*	synaptotagmin like 2
7948444	1.75E-05	1.5896369	*TCN1*	transcobalamin 1
8070579	9.91E-09	1.9432884	*TFF1*	trefoil factor 1
8070574	2.96E-05	1.1430847	*TFF2*	trefoil factor 2
8108217	5.41E-08	1.1341551	*TGFBI*	transforming growth factor beta induced
8066214	8.35E-08	1.205276	*TGM2*	transglutaminase 2
8130867	4.55E-09	1.6264449	*THBS2*	thrombospondin 2
7993638	6.43E-09	1.7178244	*TMC5*	transmembrane channel like 5
7993588	8.57E-15	1.285476	*TMC7*	transmembrane channel like 7
7945169	1.21E-08	1.3963273	*TMEM45B*	transmembrane protein 45B
7944164	2.85E-16	2.2573618	*TMPRSS4*	transmembrane protease, serine 4
8045688	1.54E-05	1.30502	*TNFAIP6*	TNF alpha induced protein 6
8015016	2.19E-12	1.4127467	*TNS4*	tensin 4
8014974	1.86E-09	1.3128398	*TOP2A*	topoisomerase (DNA) II alpha
8061579	1.48E-09	1.1088969	*TPX2*	TPX2, microtubule nucleation factor
8012953	1.65E-13	1.0200102	*TRIM16*	tripartite motif containing 16
7952290	7.15E-14	1.4077371	*TRIM29*	tripartite motif containing 29
8124707	4.49E-09	1.3998011	*TRIM31*	tripartite motif containing 31
7901175	2.49E-18	2.7266844	*TSPAN1*	tetraspanin 1
7964927	9.05E-06	1.4074942	*TSPAN8*	tetraspanin 8
8106743	4.80E-08	1.7040513	*VCAN*	versican
8169263	1.03E-09	1.9952567	*VSIG1*	V-set and immunoglobulin domain containing 1
8051322	4.34E-10	1.1333331	*XDH*	xanthine dehydrogenase

8163116	6.30E-07	-1.0014458	*EPB41L4B*	erythrocyte membrane protein band 4.1 like 4B
7922846	5.04E-07	-1.0048831	*FAM129A*	family with sequence similarity 129 member A
7947496	6.83E-06	-1.0052447	*SLC1A2*	solute carrier family 1 member 2
8098654	1.25E-10	-1.0101842	*KLKB1*	kallikrein B1
8161755	1.73E-08	-1.0122549	*ALDH1A1*	aldehyde dehydrogenase 1 family member A1
7965884	3.44E-06	-1.0185951	*PAH*	phenylalanine hydroxylase
7976322	2.99E-05	-1.02353	*CHGA*	chromogranin A
8022666	3.28E-09	-1.0283231	*CHST9*	carbohydrate sulfotransferase 9
8113666	5.34E-10	-1.0293164	*SEMA6A*	semaphorin 6A
7976488	8.47E-05	-1.0333527	*SERPINA5*	serpin family A member 5
8060134	9.80E-10	-1.0356758	*KIF1A*	kinesin family member 1A
8174513	6.56E-05	-1.0388273	*CHRDL1*	chordin like 1
8121515	5.26E-05	-1.0443833	*SLC16A10*	solute carrier family 16 member 10
8149927	3.58E-11	-1.0502238	*CLU*	clusterin
8135378	7.42E-10	-1.0549731	*PRKAR2B*	protein kinase cAMP-dependent type II regulatory subunit beta
8068684	5.66E-04	-1.0574936	*FAM3B*	family with sequence similarity 3 member B
8146687	3.89E-10	-1.0653827	*ADHFE1*	alcohol dehydrogenase, iron containing 1
8054281	4.54E-12	-1.0700796	*LONRF2*	LON peptidase N-terminal domain and ring finger 2
7922130	1.37E-06	-1.0727789	*DPT*	dermatopontin
7910915	1.69E-05	-1.0729804	*CHRM3*	cholinergic receptor muscarinic 3
8041727	1.42E-03	-1.073476	*SLC3A1*	solute carrier family 3 member 1
7993126	1.13E-08	-1.0759596	*ABAT*	4-aminobutyrate aminotransferase
8015884	1.36E-02	-1.0792696	*PPY*	pancreatic polypeptide
7937079	6.24E-06	-1.093632	*BNIP3*	BCL2 interacting protein 3
7938687	5.50E-07	-1.0996724	*NUCB2*	nucleobindin 2
7949320	5.08E-06	-1.10197	*GPHA2*	glycoprotein hormone alpha 2
7930714	1.30E-08	-1.1040269	*ATRNL1*	attractin like 1
7924309	1.15E-09	-1.1041791	*ESRRG*	estrogen related receptor gamma
8017885	1.29E-06	-1.104388	*ABCA8*	ATP binding cassette subfamily A member 8
8049990	1.60E-08	-1.1093278	*FAM150B*	family with sequence similarity 150 member B
7988987	1.52E-04	-1.1098869	*ONECUT1*	one cut homeobox 1
7944795	6.30E-07	-1.1183533	*OR4D5*	olfactory receptor family 4 subfamily D member 5
7933194	3.74E-12	-1.1287498	*CXCL12*	C-X-C motif chemokine ligand 12

7913768	7.64E-05	-1.1288124	*IL22RA1*	interleukin 22 receptor subunit alpha 1
8166784	3.70E-11	-1.1334431	*TSPAN7*	tetraspanin 7
8176193	6.97E-07	-1.1382302	*F8*	coagulation factor VIII
8056250	2.42E-02	-1.13885	*GCG*	glucagon
8106827	2.44E-09	-1.1424538	*ADGRV1*	adhesion G protein-coupled receptor V1
7986195	1.21E-08	-1.1493667	*SV2B*	synaptic vesicle glycoprotein 2B
8114964	1.26E-02	-1.1548584	*SPINK1*	serine peptidase inhibitor, Kazal type 1
7909681	1.77E-10	-1.1566953	*PROX1*	prospero homeobox 1
7922598	1.40E-11	-1.1579211	*ANGPTL1*	angiopoietin like 1
7976350	3.71E-10	-1.1597007	*UNC79*	unc-79 homolog (C. elegans)
7917304	2.28E-09	-1.1641287	*MCOLN3*	mucolipin 3
8105607	1.61E-06	-1.1697213	*FAM159B*	family with sequence similarity 159 member B
8029693	7.21E-06	-1.1699062	*FOSB*	FosB proto-oncogene, AP-1 transcription factor subunit
7908917	1.26E-10	-1.1710536	*BTG2*	BTG anti-proliferation factor 2
7948249	5.52E-07	-1.1801271	*SLC43A1*	solute carrier family 43 member 1
7976073	5.50E-10	-1.1848582	*FLRT2*	fibronectin leucine rich transmembrane protein 2
8127072	4.43E-06	-1.1862356	*GSTA1*	glutathione S-transferase alpha 1
8163839	1.30E-07	-1.1974638	*C5*	complement component 5
7983718	2.92E-07	-1.2012936	*SCG3*	secretogranin III
8057377	3.89E-11	-1.2015218	*CCDC141*	coiled-coil domain containing 141
8044700	2.52E-06	-1.204828	*DPP10*	dipeptidyl peptidase like 10
8126954	1.03E-03	-1.2130362	*PKHD1*	polycystic kidney and hepatic disease 1 (autosomal recessive)
8154848	1.02E-04	-1.2143664	*PRSS3*	protease, serine 3
7989473	2.64E-07	-1.2225682	*C2CD4B*	C2 calcium dependent domain containing 4B
8001531	5.08E-06	-1.2238896	*MT1G*	metallothionein 1G
7991034	2.00E-06	-1.248792	*HOMER2*	homer scaffolding protein 2
8056327	1.63E-07	-1.2659649	*GRB14*	growth factor receptor bound protein 14
7946579	1.42E-05	-1.2742767	*LYVE1*	lymphatic vessel endothelial hyaluronan receptor 1
7951910	1.16E-07	-1.2745662	*BACE1*	beta-secretase 1
7956229	1.41E-06	-1.2896791	*SLC39A5*	solute carrier family 39 member 5
8133876	1.18E-07	-1.2904853	*CD36*	CD36 molecule
8167027	7.44E-09	-1.2929591	*RGN*	regucalcin
8036699	2.95E-05	-1.2944747	*SYCN*	syncollin

8100853	5.86E-05	-1.3070631	*GC*	GC, vitamin D binding protein
8145532	1.13E-11	-1.309602	*EPHX2*	epoxide hydrolase 2
8042978	1.40E-03	-1.3113611	*REG3G*	regenerating family member 3 gamma
8124196	1.33E-05	-1.3135133	*DCDC2*	doublecortin domain containing 2
8089402	1.17E-06	-1.321844	*GUCA1C*	guanylate cyclase activator 1C
8092682	1.64E-06	-1.3220569	*SST*	somatostatin
8113234	1.93E-09	-1.3241064	*PCSK1*	proprotein convertase subtilisin/kexin type 1
8174201	2.54E-09	-1.3355184	*BEX1*	brain expressed X-linked 1
8111677	5.47E-11	-1.3452642	*LIFR*	leukemia inhibitory factor receptor alpha
8166202	4.45E-06	-1.3544622	*GRPR*	gastrin releasing peptide receptor
8148003	3.28E-05	-1.3557504	*SLC30A8*	solute carrier family 30 member 8
8052372	8.15E-06	-1.3568427	*MIR217*	microRNA 217
7961580	9.74E-12	-1.3596111	*LMO3*	LIM domain only 3
8074201	5.00E-07	-1.3759078	*ANKRD62*	ankyrin repeat domain 62
8110990	4.60E-08	-1.3781816	*CTNND2*	catenin delta 2
7923837	5.61E-06	-1.380062	*PM20D1*	peptidase M20 domain containing 1
8135661	5.01E-03	-1.3874427	*CFTR*	cystic fibrosis transmembrane conductance regulator
8119620	1.70E-05	-1.3901202	*GNMT*	glycine N-methyltransferase
8141016	4.15E-07	-1.4106651	*TFPI2*	tissue factor pathway inhibitor 2
8117304	1.00E-05	-1.4346631	*SLC17A4*	solute carrier family 17 member 4
8169389	5.30E-08	-1.4388473	*PAK3*	p21 (RAC1) activated kinase 3
8127065	6.38E-07	-1.4493653	*GSTA2*	glutathione S-transferase alpha 2
8105084	7.84E-06	-1.4541744	*C7*	complement component 7
8046116	1.49E-07	-1.4543064	*G6PC2*	glucose-6-phosphatase catalytic subunit 2
8057056	1.78E-11	-1.4561933	*TTN*	titin
8158987	2.16E-06	-1.4747756	*CELP*	carboxyl ester lipase pseudogene
8095585	1.26E-04	-1.5267887	*SLC4A4*	solute carrier family 4 member 4
8136801	2.13E-04	-1.5307853	*PRSS3P2*	protease, serine 3 pseudogene 2
8111864	4.60E-08	-1.5355449	*C6*	complement component 6
8020795	2.20E-03	-1.5423422	*TTR*	transthyretin
7994252	1.40E-06	-1.5447562	*AQP8*	aquaporin 8
8144786	3.48E-11	-1.5674787	*SLC7A2*	solute carrier family 7 member 2
8009493	5.72E-06	-1.5883822	*KCNJ16*	potassium voltage-gated channel subfamily J member 16

8141094	1.33E-08	-1.61166	*PDK4*	pyruvate dehydrogenase kinase 4
7944793	3.26E-07	-1.6139998	*OR8D4*	olfactory receptor family 8 subfamily D member 4
8053341	2.80E-03	-1.6238604	*REG3A*	regenerating family member 3 alpha
8151532	8.18E-06	-1.6241164	*FABP4*	fatty acid binding protein 4
8142270	1.12E-08	-1.6280118	*NRCAM*	neuronal cell adhesion molecule
7990555	8.69E-06	-1.6338384	*NRG4*	neuregulin 4
7988414	7.45E-05	-1.6627798	*GATM*	glycine amidinotransferase
8149521	1.94E-05	-1.6669633	*FGL1*	fibrinogen like 1
8058591	2.09E-08	-1.6686389	*ACADL*	acyl-CoA dehydrogenase, long chain
8101881	4.00E-08	-1.6765011	*ADH1B*	alcohol dehydrogenase 1B (class I), beta polypeptide
7957221	5.25E-07	-1.680468	*TRHDE*	thyrotropin releasing hormone degrading enzyme
8062948	8.62E-07	-1.7077773	*RBPJL*	recombination signal binding protein for immunoglobulin kappa J region like
8117288	3.72E-07	-1.7441982	*SCGN*	secretagogin, EF-hand calcium binding protein
8042986	6.69E-04	-1.7448384	*REG1A*	regenerating family member 1 alpha
8136795	2.30E-04	-1.7786876	*PRSS1*	protease, serine 1
8083246	3.07E-04	-1.8422931	*CPB1*	carboxypeptidase B1
7934936	1.94E-09	-1.8661882	*SLC16A12*	solute carrier family 16 member 12
7991335	1.81E-07	-1.8945569	*ANPEP*	alanyl aminopeptidase, membrane
8002283	6.04E-08	-1.8966689	*TMED6*	transmembrane p24 trafficking protein 6
8038633	4.35E-06	-1.9187229	*KLK1*	kallikrein 1
7925342	5.57E-11	-1.9621349	*ERO1B*	endoplasmic reticulum oxidoreductase 1 beta
8098671	4.77E-08	-2.006714	*F11*	coagulation factor XI
8047300	6.71E-12	-2.0361547	*AOX1*	aldehyde oxidase 1
7908597	1.63E-07	-2.0610724	*NR5A2*	nuclear receptor subfamily 5 group A member 2
7903592	1.35E-08	-2.0721882	*KIAA1324*	KIAA1324
7898713	2.25E-05	-2.0730927	*CELA3B*	chymotrypsin like elastase family member 3B
8096845	2.96E-06	-2.0791816	*EGF*	epidermal growth factor
8136235	1.22E-04	-2.0943071	*CPA1*	carboxypeptidase A1
7991815	4.02E-07	-2.1084087	*PDIA2*	protein disulfide isomerase family A member 2
8053337	2.53E-04	-2.1295849	*REG1CP*	regenerating family member 1 gamma, pseudogene
8053330	4.80E-04	-2.1335769	*REG1B*	regenerating family member 1 beta
8002121	1.73E-06	-2.2352664	*CTRL*	chymotrypsin like
7898725	2.93E-05	-2.2463916	*CELA3A*	chymotrypsin like elastase family member 3A

7898184	1.02E-05	-2.2770016	*CELA2B*	chymotrypsin like elastase family member 2B
7898176	1.43E-05	-2.2827218	*CELA2A*	chymotrypsin like elastase family member 2A
7967034	2.51E-05	-2.3100656	*PLA2G1B*	phospholipase A2 group IB
8091910	1.28E-06	-2.4767376	*SERPINI2*	serpin family I member 2
8125936	1.29E-05	-2.5449413	*CLPS*	colipase
7961524	1.64E-06	-2.5885804	*ERP27*	endoplasmic reticulum protein 27
8095628	1.43E-07	-2.6310982	*ALB*	albumin
8136187	2.38E-05	-2.6502338	*CPA2*	carboxypeptidase A2
8158976	1.14E-05	-2.71677	*CEL*	carboxyl ester lipase
7999920	8.79E-06	-2.793808	*GP2*	glycoprotein 2
7898167	3.47E-06	-2.7982229	*CTRC*	chymotrypsin C
7954377	2.36E-09	-2.809762	*IAPP*	islet amyloid polypeptide
7930790	2.37E-06	-3.0266571	*PNLIPRP1*	pancreatic lipase related protein 1

Table S3. The 4 DEmiRNAs' targets for 47 of the 98 downregulated genes were found in the two GEO databases

miR-222-3p	miR-345-5p	miR-100-5p	miR-221-3p
ACADL	*ACADL*	*ADH1B*	*ACADL*
ADH1B	*ADH1B*	*EPHX2*	*ADH1B*
BNIP3	*AOX1*	*G6PC2*	*BNIP3*
C6	*BACE1*	*IL22RA1*	*C6*
CHRM3	*BNIP3*	*LMO3*	*CHRM3*
DPP10	*C5*	*SLC17A4*	*DPP10*
EGF	*CHRM3*	*SLC4A4*	*EGF*
EPHX2	*CTNND2*		*EPHX2*
FAM3B	*DPP10*		*FAM3B*
KIAA1324	*EGF*		*FGL1*
LMO3	*EPHX2*		*KIAA1324*
NR5A2	*ERO1B*		*LMO3*
NRG4	*ERP27*		*NR5A2*
ONECUT1	*F11*		*NRG4*
PAK3	*F8*		*ONECUT1*
PKHD1	*FABP4*		*PAK3*
SLC17A4	*G6PC2*		*PKHD1*
SLC4A4	*GATM*		*SLC17A4*
SLC7A2	*GRB14*		*SLC4A4*
TTR	*GRPR*		*SLC7A2*
	GUCA1C		*TTR*
	IAPP		
	IL22RA1		
	KIAA1324		
	LMO3		
	NR5A2		
	NRCAM		
	NRG4		
	NUCB2		
	ONECUT1		
	PAK3		
	PDK4		
	PKHD1		
	PM20D1		
	REG3A		
	REG3G		
	RGN		
	SLC17A4		
	SLC4A4		
	SLC7A2		
	TFPI2		

TRHDE
TTN
TTR

Table S4. Clinicopathological features of samples used in this study.

Type of samples	Cohort	Assay	Sample	Gender	Age	Alcohol	Smoking	Ethnicity	Tumor size	TNM	Differentiation grade	Survival
Tissue	First Cohort Controls (13)	Microarrays Identification=3	Control 1	F	36	-	-	Mexican-Mestizo	-	-	-	-
			Control 2	F	49	-	-	Mexican-Mestizo	-	-	-	-
			Control 3	M	52	-	-	Mexican-Mestizo	-	-	-	-
		Validation (pool) RT-qPCR=10	Control 4	M	50	-	-	Mexican-Mestizo	-	-	-	-
			Control 5	M	50	-	-	Mexican-Mestizo	-	-	-	-
			Control 6	F	47	-	-	Mexican-Mestizo	-	-	-	-
			Control 7	F	58	-	-	Mexican-Mestizo	-	-	-	-
			Control 8	F	64	-	-	Mexican-Mestizo	-	-	-	-
			Control 9	M	59	-	-	Mexican-Mestizo	-	-	-	-
			Control 10	M	78	-	-	Mexican-Mestizo	-	-	-	-
			Control 11	M	73	-	-	Mexican-Mestizo	-	-	-	-
			Control 12	M	70	-	-	Mexican-Mestizo	-	-	-	-
			Control 13	M	66	-	-	Mexican-Mestizo	-	-	-	-
	Second Cohort Controls (15)	Validation (independent) RT-qPCR=15	Control 14	M	31	-	-	Mexican-Mestizo	-	-	-	-
			Control 15	M	49	-	-	Mexican-Mestizo	-	-	-	-
			Control 16	F	28	-	-	Mexican-Mestizo	-	-	-	-
			Control 17	F	38	-	-	Mexican-Mestizo	-	-	-	-
			Control 18	M	26	-	-	Mexican-Mestizo	-	-	-	-

			Control 19	F	33	-	-	Mexican-Mestizo	-	-	-	-
			Control 20	M	74	-	-	Mexican-Mestizo	-	-	-	-
			Control 21	F	26	-	-	Mexican-Mestizo	-	-	-	-
			Control 22	M	60	-	-	Mexican-Mestizo	-	-	-	-
			Control 23	M	40	-	-	Mexican-Mestizo	-	-	-	-
			Control 24	M	27	-	-	Mexican-Mestizo	-	-	-	-
			Control 25	M	65	-	-	Mexican-Mestizo	-	-	-	-
			Control 26	M	27	-	-	Mexican-Mestizo	-	-	-	-
			Control 27	F	64	-	-	Mexican-Mestizo	-	-	-	-
			Control 28	F	52	-	-	Mexican-Mestizo	-	-	-	-
	First Cohort Patients (25)	Microarrays Identification=12	Patient 1	M	66	No data	No data	Mexican-Mestizo	5.5	IIA	G2	-
			Patient 2	F	66	No data	No data	Mexican-Mestizo	-	IIA	G2	13
			Patient 3	F	57	No data	No data	Mexican-Mestizo	5.5	IIA	G2	-
			Patient 4	M	48	No data	No data	Mexican-Mestizo	5.1	IIB	G2	18
			Patient 5	M	75	No data	No data	Mexican-Mestizo	-	IIB	G2	12
			Patient 6	F	47	No data	No data	Mexican-Mestizo	9	IIB	G2	11
			Patient 7	M	78	No data	No data	Mexican-Mestizo	7.4	III	G1	4
			Patient 8	M	40	No data	No data	Mexican-Mestizo	2.59	III	G2	20
			Patient 9	M	61	No data	No data	Mexican-Mestizo	6.8	III	G3	4

			**Patient 10**	**F**	**66**	**No data**	**No data**	**Mexican-Mestizo**	**40**	**IV**	**G2**	**1**
			**Patient 11**	**F**	**64**	**No data**	**No data**	**Mexican-Mestizo**	**-**	**IV**	**G2**	**-**
			**Patient 12**	**M**	**54**	**No data**	**No data**	**Mexican-Mestizo**	**-**	**IV**	**G3**	**10**
		**Validation (pool) RT-qPCR=13**	**Patient 13**	**F**	**81**	**No data**	**No data**	**Mexican-Mestizo**	**5**	**IIB**	**G2**	**14**
			**Patient 14**	**M**	**66**	**No data**	**No data**	**Mexican-Mestizo**	**5**	**IIB**	**G2**	**14**
			**Patient 15**	**M**	**52**	**No data**	**No data**	**Mexican-Mestizo**	**3**	**IIB**	**G2**	**5**
			**Patient 16**	**M**	**72**	**No data**	**No data**	**Mexican-Mestizo**	**3.96**	**III**	**G3**	**9**
			**Patient 17**	**M**	**70**	**No data**	**No data**	**Mexican-Mestizo**	**2.1**	**III**	**G2**	**9**
			**Patient 18**	**M**	**47**	**No data**	**No data**	**Mexican-Mestizo**	**2.1**	**III**	**G2**	**-**
			**Patient 19**	**F**	**55**	**No data**	**No data**	**Mexican-Mestizo**	**16**	**III**	**G2**	**16**
			**Patient 20**	**F**	**42**	**No data**	**No data**	**Mexican-Mestizo**	**4.1**	**IV**	**G2**	**20**
			**Patient 21**	**F**	**56**	**No data**	**No data**	**Mexican-Mestizo**	**5**	**IV**	**G2**	**3**
			**Patient 22**	**F**	**62**	**No data**	**No data**	**Mexican-Mestizo**	**-**	**IV**	**G2**	**1**
			**Patient 23**	**F**	**68**	**No data**	**No data**	**Mexican-Mestizo**	**5**	**IV**	**G3**	**10**
			**Patient 24**	**F**	**54**	**No data**	**No data**	**Mexican-Mestizo**	**-**	**IV**	**G3**	**-**
			**Patient 25**	**F**	**69**	**No data**	**No data**	**Mexican-Mestizo**	**6**	**IV**	**G3**	**-**
	**Second Cohort Patients (15)**	**Validation (independent) RT-qPCR=15**	**Patient 26**	**F**	**86**	**No data**	**No data**	**Mexican-Mestizo**	**2.1**	**IIB**	**G2**	**-**
			**Patient 27**	**M**	**60**	**No data**	**No data**	**Mexican-Mestizo**	**6**	**IIB**	**G2**	**-**
			**Patient 28**	**F**	**75**	**No data**	**No data**	**Mexican-Mestizo**	**-**	**-**	**G2**	**-**

			Patient 29	F	77	No data	No data	Mexican-Mestizo	4	IIB	G2	-
			Patient 30	F	62	No data	No data	Mexican-Mestizo	-	-	G2	-
			Patient 31	M	65	No data	No data	Mexican-Mestizo	-	-	G2	-
			Patient 32	M	64	No data	No data	Mexican-Mestizo	3.2	IIA	G2	-
			Patient 33	F	77	No data	No data	Mexican-Mestizo	4	IIB	G2	-
			Patient 34	M	68	No data	No data	Mexican-Mestizo	-	IIA	-	-
			Patient 35	F	58	No data	No data	Mexican-Mestizo	-	IIA	G2	-
			Patient 36	F	66	No data	No data	Mexican-Mestizo	-	IIA	G2	132
			Patient 37	F	47	No data	No data	Mexican-Mestizo	9	IIB	G2	11
			Patient 38	M	78	No data	No data	Mexican-Mestizo	7.4	III	G1	46
			Patient 39	F	64	No data	No data	Mexican-Mestizo	-	IV	G2	-
			Patient 40	F	68	No data	No data	Mexican-Mestizo	5	IV	G3	10
Plasma	First Cohort Controls (20)	Validation (independent) RT-qPCR=20	Control 1	F	43	-	-	Mexican-Mestizo	-	-	-	-
			Control 2	F	47	-	-	Mexican-Mestizo	-	-	-	-
			Control 3	F	51	-	-	Mexican-Mestizo	-	-	-	-
			Control 4	M	54	-	-	Mexican-Mestizo	-	-	-	-
			Control 5	F	47	-	-	Mexican-Mestizo	-	-	-	-
			Control 6	F	39	-	-	Mexican-Mestizo	-	-	-	-
			Control 7	F	56	-	-	Mexican-Mestizo	-	-	-	-

			Control 8	F	45	-	-	Mexican-Mestizo	-	-	-	-
			Control 9	F	62	-	-	Mexican-Mestizo	-	-	-	-
			Control 10	M	49	-	-	Mexican-Mestizo	-	-	-	-
			Control 11	M	47	-	-	Mexican-Mestizo	-	-	-	-
			Control 12	M	44	-	-	Mexican-Mestizo	-	-	-	-
			Control 13	M	30	-	-	Mexican-Mestizo	-	-	-	-
			Control 14	M	30	-	-	Mexican-Mestizo	-	-	-	-
			Control 15	F	57	-	-	Mexican-Mestizo	-	-	-	-
			Control 16	M	27	-	-	Mexican-Mestizo	-	-	-	-
			Control 17	M	31	-	-	Mexican-Mestizo	-	-	-	-
			Control 18	M	27	-	-	Mexican-Mestizo	-	-	-	-
			Control 19	F	27	-	-	Mexican-Mestizo	-	-	-	-
			Control 20	F	50	-	-	Mexican-Mestizo	-	-	-	-
	First Cohort Patients (46)	Validation (independent) RT-qPCR=46	Patient 1	F	63	No	No	Mexican-Mestizo	-	IV	-	-
			Patient 2	M	44	No	Yes	Mexican-Mestizo	-	III	-	-
			Patient 3	F	70	Yes	Yes	Mexican-Mestizo	-	IV	-	-
			Patient 4	F	69	Yes	No	Mexican-Mestizo	-	III	-	-
			Patient 5	F	56	No	No	Mexican-Mestizo	-	III	-	-
			Patient 6	M	78	No	No	Mexican-Mestizo	-	III	-	-

			Patient 7	F	79	No	No	Mexican-Mestizo	-	IV	-	-
			Patient 8	F	82	No	Yes	Mexican-Mestizo	-	IV	-	-
			Patient 9	F	51	Yes	Yes	Mexican-Mestizo	-	III	-	-
			Patient 10	F	70	No	No	Mexican-Mestizo	-	IV	-	-
			Patient 11	F	41	No	No	Mexican-Mestizo	-	IV	-	-
			Patient 12	M	48	No	No	Mexican-Mestizo	-	III	-	-
			Patient 13	F	83	Yes	Yes	Mexican-Mestizo	-	IV	-	-
			Patient 14	M	63	No	No	Mexican-Mestizo	-	IV	-	-
			Patient 15	M	46	No data	No data	Mexican-Mestizo	-	IV	-	-
			Patient 16	F	63	No	Yes	Mexican-Mestizo	-	III	-	-
			Patient 17	F	75	Yes	Yes	Mexican-Mestizo	-	IV	-	-
			Patient 18	F	65	No	Yes	Mexican-Mestizo	-	III	-	-
			Patient 19	M	75	No	No	Mexican-Mestizo	-	IV	-	-
			Patient 20	M	56	No data	No data	Mexican-Mestizo	-	III	-	-
			Patient 21	M	62	No	No	Mexican-Mestizo	-	IV	-	-
			Patient 22	F	68	Yes	Yes	Mexican-Mestizo	-	I	-	-
			Patient 23	F	51	No	Yes	Mexican-Mestizo	-	III	-	-
			Patient 24	M	48	No	Yes	Mexican-Mestizo	-	IV	-	-
			Patient 25	M	56	No	No	Mexican-Mestizo	-	IV	-	-

			Patient 26	F	71	No	Yes	Mexican-Mestizo	-	IV	-	-
			Patient 27	M	48	No	No	Mexican-Mestizo	-	III	-	-
			Patient 28	M	72	No	No	Mexican-Mestizo	-	III	-	-
			Patient 29	M	46	No	No	Mexican-Mestizo	-	IV	-	-
			Patient 30	F	63	No	No	Mexican-Mestizo	-	IV	-	-
			Patient 31	M	69	No	No	Mexican-Mestizo	-	III	-	-
			Patient 32	F	51	Yes	No data	Mexican-Mestizo	-	II	-	-
			Patient 33	F	50	No	No data	Mexican-Mestizo	-	II	-	-
			Patient 34	M	62	No	No data	Mexican-Mestizo	-	IV	-	-
			Patient 35	F	60	No	Yes	Mexican-Mestizo	-	III	-	-
			Patient 36	M	57	No data	No data	Mexican-Mestizo	-	II	-	-
			Patient 37	M	65	No data	No data	Mexican-Mestizo	-	IV	-	-
			Patient 38	M	60	No data	No data	Mexican-Mestizo	-	III	-	-
			Patient 39	M	60	No data	No data	Mexican-Mestizo	-	III	-	-
			Patient 40	M	44	No data	No data	Mexican-Mestizo	-	III	-	-
			Patient 41	F	82	No data	No	Mexican-Mestizo	-	IV	-	-
			Patient 42	F	71	Yes	Yes	Mexican-Mestizo	-	III	-	-
			Patient 43	F	68	No	Yes	Mexican-Mestizo	-	-	-	-
			Patient 44	M	-	No	No	Mexican-Mestizo	-	-	-	-

			**Patient 45**	**F**	**63**	**No**	**No**	**Mexican-Mestizo**	**-**	**II**	**-**	**-**
			**Patient 46**	**F**	**71**	**Yes**	**Yes**	**Mexican-Mestizo**	**2.9**	**II**	**-**	**-**

Blue letters correspond to plasma samples and dark letters indicate tissue samples.
